# Supplementary material for: Asymmetric C–H Dehydrogenative Alkenylation via a Photo-induced Chiral α‑Imino Radical Intermediate
Source: Nat Commun. 2024 May 14;15:4044. doi: 10.1038/s41467-024-48350-w (PMC11094157; doi:10.1038/s41467-024-48350-w)
Supplement: Supplementary file 1 — Supplementary Information [file 41467_2024_48350_MOESM1_ESM.pdf]

Supplementary Information

Asymmetric C–H Dehydrogenative Alkenylation *via* a Photo-induced Chiral α-Imino Radical Intermediate

Zongbin Jia[1], Liang Cheng[1], Long Zhang[1], Sanzhong Luo*[1]

[1]Center of Basic Molecular Science, Department of Chemistry, Tsinghua University, Beijing, China, 100084.

E-mail: luosz@tsinghua.edu.cn

Table of Contents

1. **General information** S3

2. **Optimization of reaction condition** S4

3. **Experimental section**..S14

   3.1. **General experimental procedure**..S14

   3.2. **Synthesis of substrates**..S15

   3.3. **Determination of absolute configuration**...S23

   3.4. **Emission quenching experiments**..S24

   3.5. **DFT calculations** S27

4. **Characterization of compounds**..S28

5. **NMR spectra**..S49

6. **HPLC spectra**..S108

7. **References**..S155

General information: Commercial reagents were used as received, unless otherwise indicated. Nuclear magnetic resonance (NMR) spectra were recorded using Bruker AV-400 spectrometers (400 MHz for ^{1}H NMR, 100 MHz for ^{13}C NMR and 376 MHz for ^{19}F NMR). Solvent resonance as the internal standard (^{1}H NMR: $CDCl_3$ at 7.26 ppm; ^{13}C NMR: $CDCl_3$ at 77.16 ppm). The following abbreviations were used to express the multiplicities: s = singlet; d = doublet; t = triplet; q = quartet; m = multiplet; dd = doublet; dt = doublet of triplet; ddd = doublet of doublets of doublets; br = broad. Silica gel (100-200 mesh) was used for column chromatography. Analytical gas chromatography (GC) was carried out on a Shimadzu GC-2010 plus and a RT-Msieve 5A column (30 m, 0.32 mm ID, 30 µm, cat. # 19722). Infrared Spectroscopy was conducted on Thermo Fisher Nicolet 6700. High resolution mass spectra were obtained using electrospray ionization (ESI) mass spectrometer. HPLC analysis was performed using Chiralcel columns purchased (Chiral Daicel Chiralpak IC, IA, IE, AD-H, AS-H, OJ-H, OD-H columns). Optical rotations were measured using a 1 mL cell with a 1 cm path length on a Perkin-Elmer 341 digital polarimeter and reported as follows: $[\alpha]_D^{20}$ (c in g per 100 mL of solvent).

2. Optimization of Reaction Conditions

Supplementary Table 1. Screening of the hydrogen acceptors.[a]

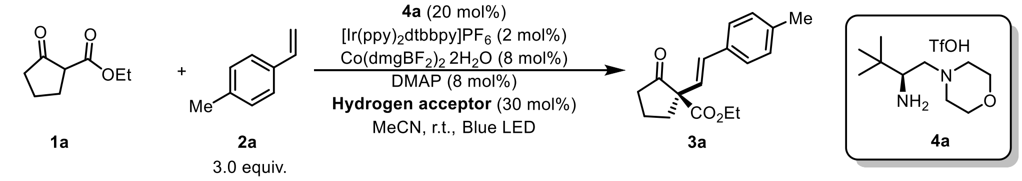

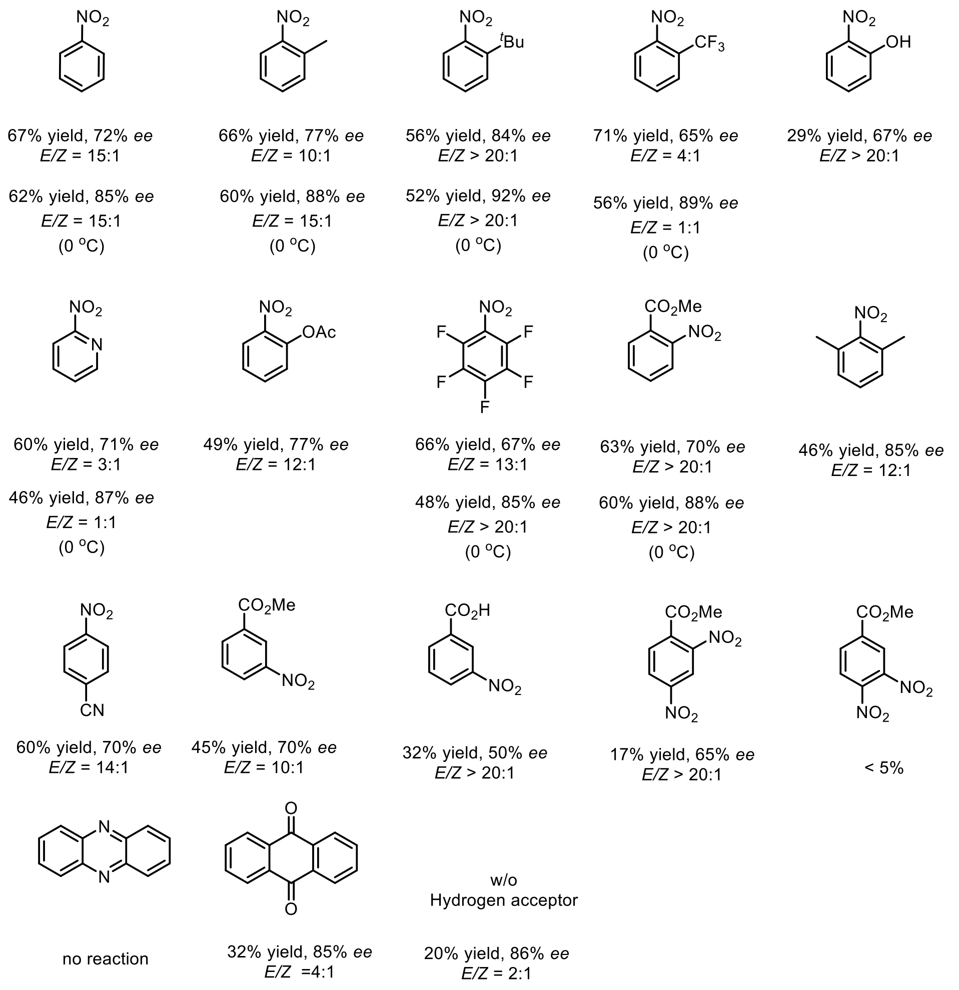

[a]Reactions were performed with **1a** (0.1 mmol), **2a** (0.3 mmol), amine catalyst **4a** (20 mol%), $Co(dmgBF_2)_2(H_2O)_2$ (8 mol%), $[Ir(ppy)_2dtbbpy]PF_6$ (2 mol%), DMAP (8 mol%), hydrogen acceptor (30 mol%), 0.3 mL MeCN, deaerated and irradiated for 24 h by a 30 W blue LED under room temperature. The yield and *E/Z* ratio were determined by GC analysis using 1,3,5-trimethoxybenzene as an internal standard. Enantioselectivity was determined by chiral HPLC analysis. w/o = without.

The importance of hydrogen acceptor:

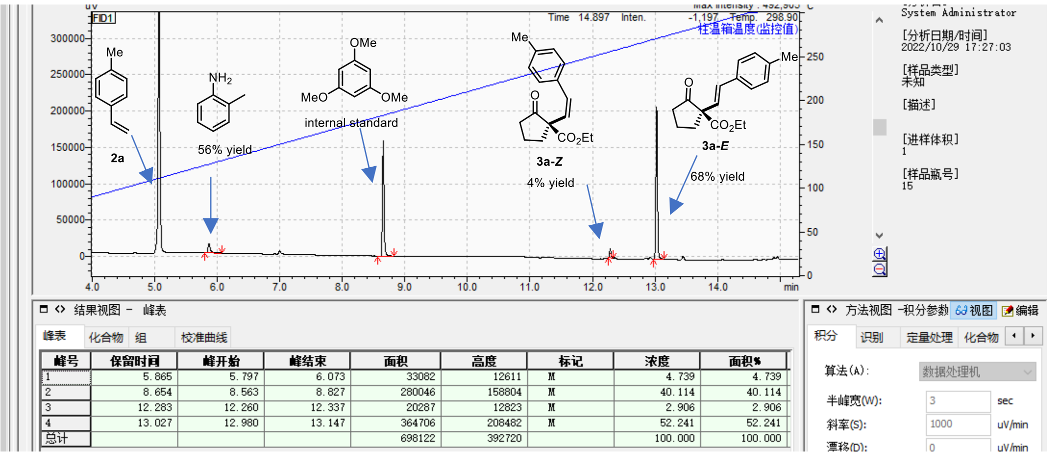

峰号	保留时间	峰开始	峰结束	面积	高度	标记	浓度	面积%
1	5.865	5.797	6.073	33082	12611	M	4.739	4.739
2	8.654	8.563	8.827	280046	158804	M	40.114	40.114
3	12.283	12.260	12.337	20287	12823	M	2.906	2.906
4	13.027	12.980	13.147	364706	208482	M	52.241	52.241
总计				698122	392720		100.000	100.000

Supplementary Fig. 1. GC spectrum of the reaction mixture under the standard condition. With *o*-Me-$C_6H_4NO_2$ (25 mol%) as hydrogen acceptor. The reaction mixture was detected by GC analysis with an internal standard.

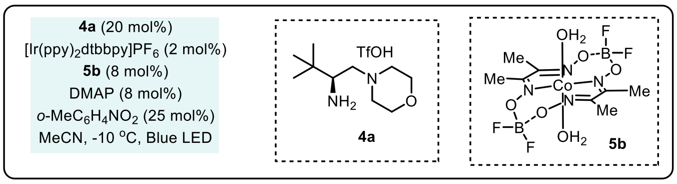

Standard condition

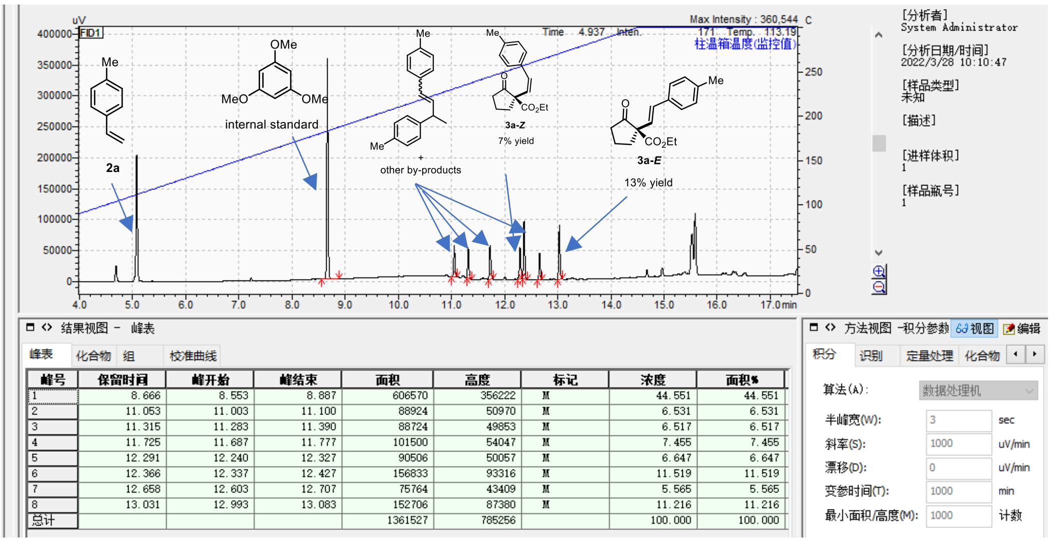

峰号	保留时间	峰开始	峰结束	面积	高度	标记	浓度	面积%
1	8.666	8.553	8.887	606570	356222	M	44.551	44.551
2	11.053	11.003	11.100	88924	50970	M	6.531	6.531
3	11.315	11.283	11.390	88724	49853	M	6.517	6.517
4	11.725	11.687	11.777	101500	54047	M	7.455	7.455
5	12.291	12.240	12.327	90506	50057	M	6.647	6.647
6	12.366	12.337	12.427	156833	93316	M	11.519	11.519
7	12.658	12.603	12.707	75764	43409	M	5.565	5.565
8	13.031	12.993	13.083	152706	87380	M	11.216	11.216
总计				1361527	785256		100.000	100.000

Supplementary Fig. 2. GC spectrum of the reaction mixture *w/o o*-Me-$C_6H_4NO_2$. The reaction mixture was detected by GC analysis with an internal standard,

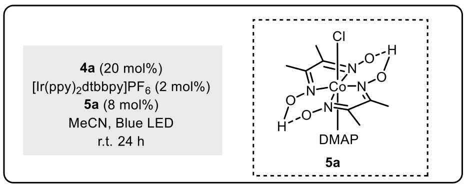

Our previous condition

Supplementary Table 2. Screening of the temperature and amount of hydrogen acceptor.[a]

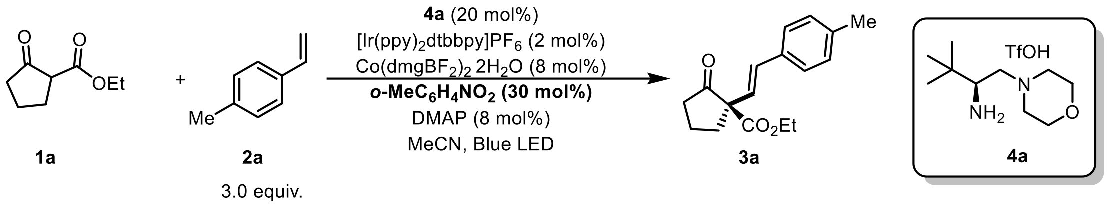

Entry	Conditions	Yield **3a** (%)	*E/Z*	*ee.*
1	Room temperature	66	10:1	77
2	10 °C	62	14:1	83
3	0 °C	60	15:1	88
4	-5 °C	60	11:1	89
5	-10 °C	57	10:1	91
6	-20 °C	39	> 20:1	95
7	-10 °C; H-acceptor 20 mol%	43	11:1	91
8	-10 °C; H-acceptor 25 mol%	56	12:1	91
9	-10 °C; H-acceptor 50 mol%	36	> 20:1	91
10	-10 °C; H-acceptor 1.0 equiv.	< 10	--	--

[a]Reactions were performed with **1a** (0.1 mmol), **2a** (0.3 mmol), amine catalyst **4a** (20 mol%), $Co(dmgBF_2)_2(H_2O)_2$ (8 mol%), $[Ir(ppy)_2dtbbpy]PF_6$ (2 mol%), DMAP (8 mol%), *o*-Me-$C_6H_4NO_2$ (30 mol%), 0.3 mL MeCN, deaerated and irradiated for 48 h by a 30 W blue LED under corresponding temperature. The yield and *E/Z* ratio were determined by GC analysis using 1,3,5-trimethoxybenzene as an internal standard. Enantioselectivity was determined by chiral HPLC analysis.

Supplementary Table 3. Screening of the base additives.[a]

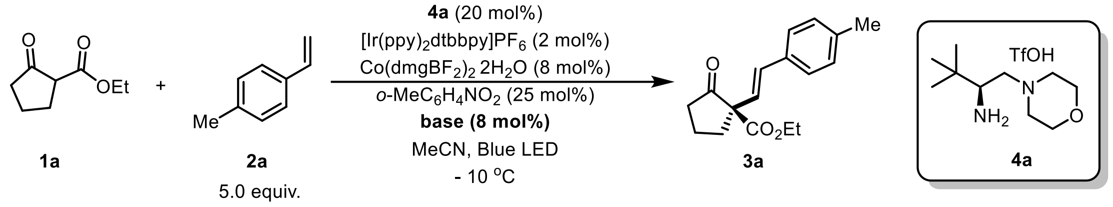

Entry	Base (8 mol%)	Yield **3a** (%)	*E/Z*	*ee.*
**1**	**DMAP**	**73(71)**	**18:1**	**93**
2	DABCO	38	> 20:1	95
3	DBU	26	> 20:1	93
4	MeONa	66	14:1	91
5	*o*-Phenanthroline	< 5	--	--
6	4,4'-Di-tertbutyl-2,2'-bipyridine (dtbbpy)	< 5	--	--
7	2,6-lutidine	65	15:1	91
8	pyridine	68	12:1	92
9	K_2CO_3	54	> 20:1	90
8	DMAP 2 mol%	23	6:1	91
9	DMAP 12 mol%	36	14:1	87
10	DMAP 50 mol%	< 10	--	--
11	w/o DMAP	8	4:1	43

[a]Reactions were performed with **1a** (0.1 mmol), **2a** (0.5 mmol), amine catalyst (20 mol%), $Co(dmgBF_2)_2(H_2O)_2$ (8 mol%), $[Ir(ppy)_2dtbbpy]PF_6$ (2 mol%), DMAP (8 mol%), *o*-$MeC_6H_4NO_2$ (25 mol%), 0.3 mL MeCN, deaerated and irradiated for 48 h by a 30 W blue LED under corresponding temperature. The yield and *E/Z* ratio were determined by GC analysis using 1,3,5-trimethoxybenzene as an internal standard. Enantioselectivity was determined by chiral HPLC analysis. w/o = without.

Supplementary Table 4. Screening of the cobalt catalysts.[a]

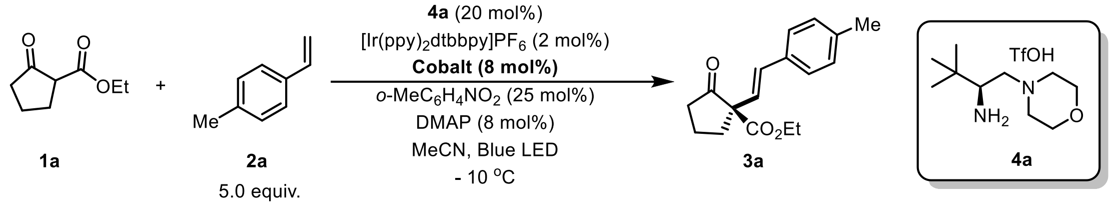

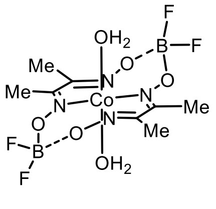

$Co(dmgBF_2)_2(H_2O)_2$
5b
73% yield, 93% *ee*
E/Z = 18:1

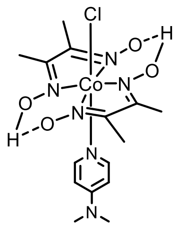

5a
6% yield, 70% *ee*
E/Z > 20:1

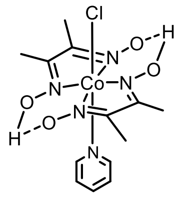

5c
11% yield, 83% *ee*
E/Z > 20:1

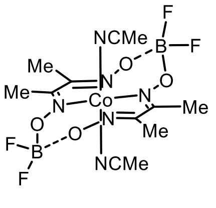

$Co(dmgBF_2)_2(MeCN)_2$
5d
70% yield, 93% *ee*
E/Z = 13:1

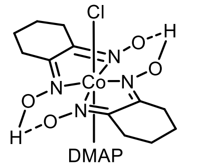

5e
8% yield, 72% *ee*
E/Z > 20:1

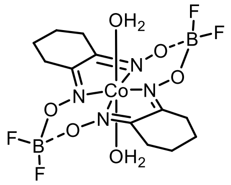

5f
45% yield, 91% *ee*
E/Z > 20:1

[a]Reactions were performed with **1a** (0.1 mmol), **2a** (0.5 mmol), amine catalyst **4a** (20 mol%), cobalt (8 mol%), $[Ir(ppy)_2dtbbpy]PF_6$ (2 mol%), DMAP (8 mol%), *o*-$MeC_6H_4NO_2$ (25 mol%), 0.3 mL MeCN, deaerated and irradiated for 48 h by a 30 W blue LED under corresponding temperature. The yield and *E/Z* ratio were determined by GC analysis using 1,3,5-trimethoxybenzene as an internal standard. Enantioselectivity was determined by chiral HPLC analysis.

Supplementary Table 5. Screening for Co(dmgH$_2$)$_2$DMAPCl.[a]

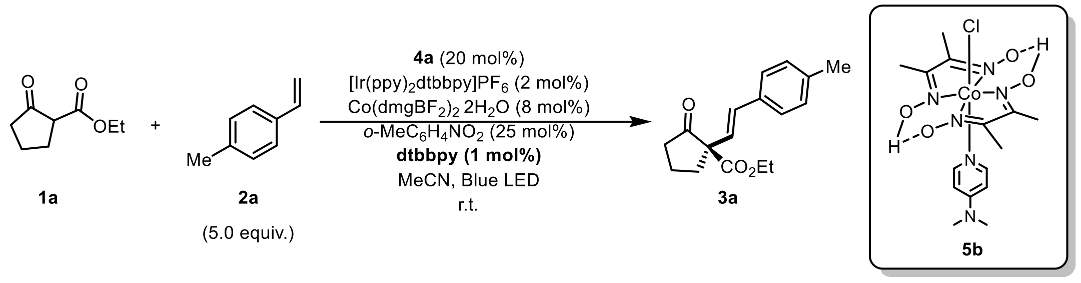

Entry	Variation from condition	Yield **3a** (%)	*E/Z*	*ee.*
1	none	35	2:1	71
2	-10 ºC	10	8:1	74
4	*m*-NO_2-$C_6H_4CO_2Me$ (30 mol%)	40	3:1	68
5	$PhNO_2$ (30 mol%)	42	2:1	73
6	$C_6F_5NO_2$ (30 mol%)	37	1:1	72
7[b]	w/o dtbbpy	18	4:1	80
8[b]	*o*-Phenanthroline (1 mol%) as base additive	24	>20:1	81
9	DMAP (8 mol%) as base additive	11	>20:1	76

[a]Reactions were performed with **1a** (0.1 mmol), **2a** (0.5 mmol), amine catalyst **4a** (20 mol%), Co(dmgH$_2$)$_2$DMAPCl (8 mol%), [Ir(ppy)$_2$dtbbpy]PF$_6$ (2 mol%), dtbbpy (1 mol%), *o*-$MeC_6H_4NO_2$ (30 mol%), 0.3 mL MeCN, deaerated and irradiated for 24 h by a 30 W blue LED under corresponding temperature. The yield and *E/Z* ratio were determined by GC analysis using 1,3,5-trimethoxybenzene as an internal standard. Enantioselectivity was determined by chiral HPLC analysis. [b]Without hydrogen acceptor,

Supplementary Table 6. Screening of the amine catalysts.[a]

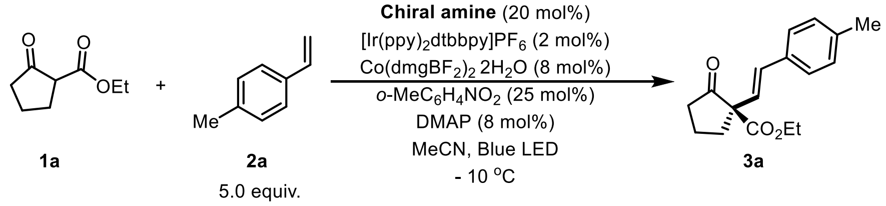

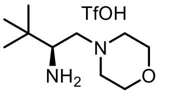

4a
73% yield, 93% *ee*
E/Z = 18:1

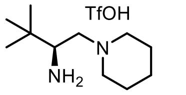

4b
20% yield, 81% *ee*
E/Z = 19:1

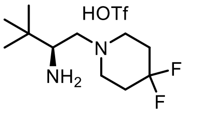

4c
55% yield, 85% *ee*
E/Z > 20:1

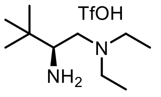

4d
13% yield, 72% *ee*
E/Z > 20:1

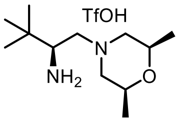

4e
66% yield, 91% *ee*
E/Z = 15:1

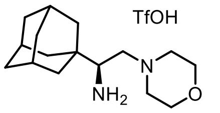

4f
70% yield, 93% *ee*
E/Z = 17:1

w/o chiral amine
< 1%

[a]Reactions were performed with **1a** (0.1 mmol), **2a** (0.5 mmol), amine catalyst (20 mol%), $Co(dmgBF_2)_2(H_2O)_2$ (8 mol%), $[Ir(ppy)_2dtbbpy]PF_6$ (2 mol%), DMAP (8 mol%), *o*-$MeC_6H_4NO_2$ (25 mol%), 0.3 mL MeCN, deaerated and irradiated for 48 h by a 30 W blue LED under corresponding temperature. The yield and *E/Z* ratio were determined by GC analysis using 1,3,5-trimethoxybenzene as an internal standard. Enantioselectivity was determined by chiral HPLC analysis. w/o = without.

Supplementary Table 7. Screening of the photoredox catalysts.[a]

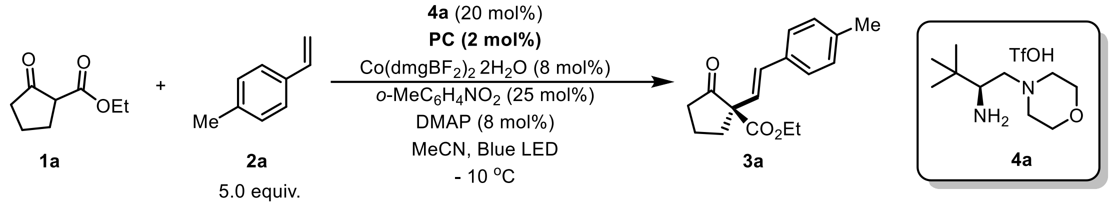

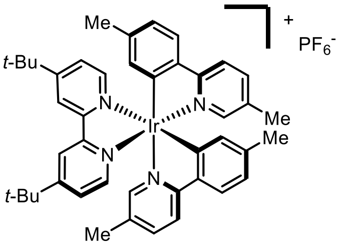

< 10% yield

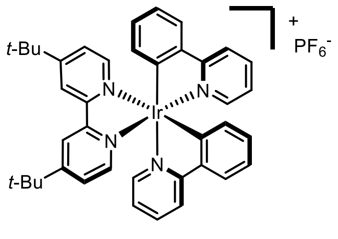

$[Ir(ppy)_2dtbbpy]PF_6$
[Ir]
73% yield, 93% *ee*
E/Z = 18:1

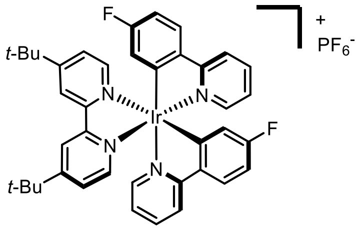

$[Ir(F\text{-}ppy)_2dtbbpy]PF_6$
[Ir]-F
72% yield, 92% *ee*
E/Z = 2:1

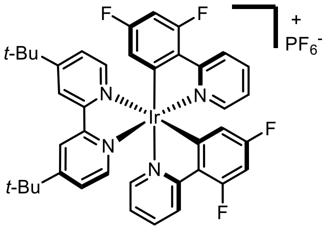

$[Ir(dF\text{-}ppy)_2dtbbpy]PF_6$
[Ir]-dF
75% yield, 92% *ee*
E/Z = 1:1

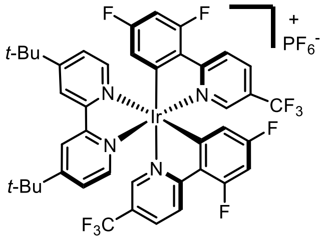

< 10% yield

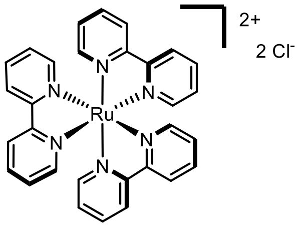

< 5% yield

[a]Reactions were performed with **1a** (0.1 mmol), **2a** (0.5 mmol), amine catalyst **4a** (20 mol%), $Co(dmgBF_2)_2(H_2O)_2$ (8 mol%), photoredox catalyst (2 mol%), DMAP (8 mol%), *o*-$MeC_6H_4NO_2$ (25 mol%), 0.3 mL MeCN, deaerated and irradiated for 48 h by a 30 W blue LED under corresponding temperature. The yield and *E/Z* ratio were determined by GC analysis using 1,3,5-trimethoxybenzene as an internal standard. Enantioselectivity was determined by chiral HPLC analysis.

Supplementary Table 8. Screening of the ratio of substrates and solvents.[a]

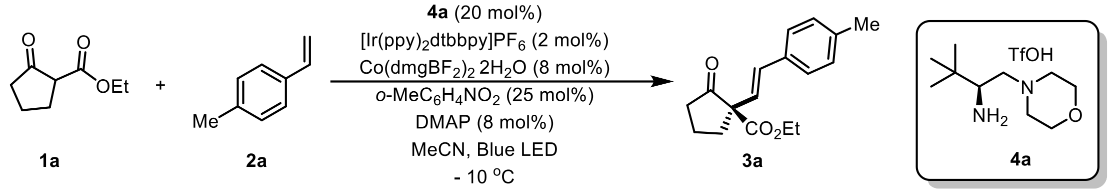

Entry	Variation from condition	Yield **3a** (%)	*E/Z*	*ee.*
1	**1a** : **2a** = 1 : 3	62	15:1	91
2	**1a** : **2a** = 2 : 1	40	11:1	85
3	MeCN 0.1 mL	46	10:1	87
4	MeCN 0.5 mL	65	15:1	93
5	MeCN 1.0 mL	52	> 20:1	95
6[b]	*t*Bu-CN	38	1:1	76
7[b]	PhCN	56	6:1	65
8[b]	Toluene	20	5:1	54
9[b]	HFIP	7	2:1	23
10[b]	EtOH	31	7:1	54
11[b]	THF	31	4:1	59
12[b]	DCM	33	1:4	60
13[b]	DMF	14	1:1	77
14[b]	Et_2O	16	1:2	50
15[b]	Hexane	22	1:4	26
16[b]	DMSO	11	1:2	73
17[b]	Acetone	24	3:1	73

[a]Reactions were performed with **1a** (0.1 mmol), **2a** (0.5 mmol), amine catalyst **4a** (20 mol%), $Co(dmgBF_2)_2(H_2O)_2$ (8 mol%), $[Ir(ppy)_2dtbbpy]PF_6$ (2 mol%), DMAP (8 mol%), *o*-$MeC_6H_4NO_2$ (25 mol%), 0.3 mL solvent, deaerated and irradiated for 24-48 h by a 30 W blue LED under corresponding temperature. The yield and *E/Z* ratio were determined by GC analysis using 1,3,5-trimethoxybenzene as an internal standard. Enantioselectivity was determined by chiral HPLC analysis. [b]Reaction under room temperature.

$$\Delta\Delta G = 0.1010 \times \delta_p - 0.4656 \times SA + 0.5037$$

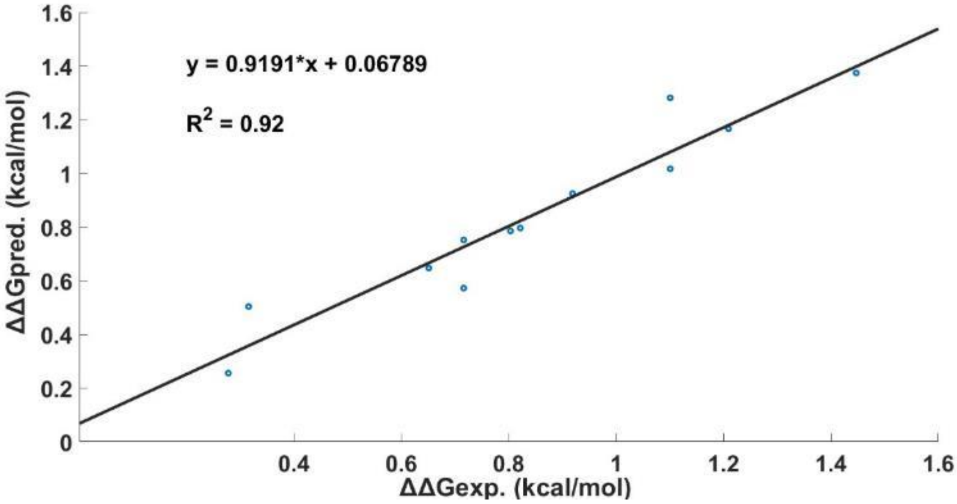

Supplementary Fig. 3. Stepwise multivariant linear free energy correlation analysis.

3. Experimental section

3.1 General experimental procedure

General procedure for the asymmetric alkenylation of β-ketocarbonyls

In an oven-dried 5 mL pyrex tube equipped with a magnetic stir bar, β-ketocarbonyls **1** (0.1 mmol), $[Ir(ppy)_2dtbbpy]PF_6$ (1.86 mg, 2 mol%), $Co(dmgBF_2)_2$ $2H_2O$ (3.36 mg, 8 mol%), chiral primary amine **4a** (6.72 mg, 20 mol%), DMAP (0.98 mg, 8mol%), alkene (0.5 mmol) and MeCN (0.3 mL) were added. The mixture was equipped with a rubber septum and bubbled with a stream of argon for 15 min. The sample was then irradiated by a 30 W blue LED under -10 ^oC condition for 48 h until completion as indicated by TLC. The reaction mixture was directly loaded onto silica gel column and eluted with diethyl ether /hexane to give the target product.

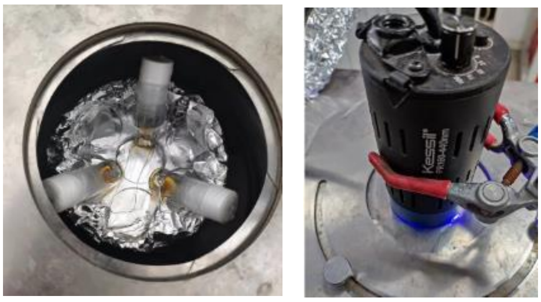

Supplementary Fig. 4. The reaction set-up of the asymmetric alkenylation reaction. Left: put the reaction vials into cooler. Right: irradiation the vials with a 30 W 440 nm LED.

Gram scale reaction:

An oven-dried 100 mL round-bottom flask equipped with a magnetic stir bar, ethyl 2-oxocyclopentanecarboxylate **1a** (1.56 g, 10 mmol), 4-methylstyrene **2a** (5.90 g, 50 mmol), $[Ir(ppy)_2dtbbpy]PF_6$ (63.7 mg, 0.7 mol%), amine catalyst **4a** (504 mg, 15 mol%), **5b** (336 mg, 8 mol%), DMAP (98.0 mg, 8 mol%) and 30 mL MeCN were added. After degassing 3 times by a standard freeze-thaw operation, the flask was placed approximately 5 cm from a 30 W blue LED at -10 ^oC until completion as indicated by TLC (96h). After removal of the solvent, saturated Na_2CO_3 (30 mL) solution and DCM (30 mL) was added to the mixture and the organic phases were separated. The aqueous phase was extracted with DCM (10 mL × 3) and the combined organic layers were dry with anhydrous sodium sulfate, concentrated under reduced pressure. The crude residue was directly loaded onto silica gel

column and eluted with PE: EA = 5:1 to give the desired product **3a** (1.65 g, 61% yield, 91% *ee*, *E/Z* > 20:1).

3.2 Synthesis of substrates

General procedure for enamine synthesis:

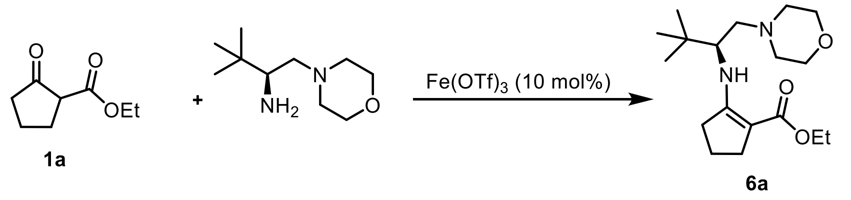

The secondary enamine **6a** was synthesized according to procedure reported in the literature.[3] $Fe(OTf)_3$ (0.2 mmol, 41 mg) was added to a mixture of primary amines (2 mmol) and β-ketocarbonyl compounds **1a** (2 mmol) in a 25 mL round-bottom flask (neat) and the mixtures were allowed to stir at ambient temperature. The completion of the reaction was monitored by TLC. The pure enamine **6a** was obtained by passing them directly through alkaline alumina column chromatography using hexane/ethyl acetate = 5:1 as eluants.

All of *β*-ketoesters and *β*-ketoamides were commercially available and used directly without further purification except the following substrates. *β*-Ketoamides **1ad**, **1af** and *β*-Ketoester **1am** were prepared according to literature precedent.[1]

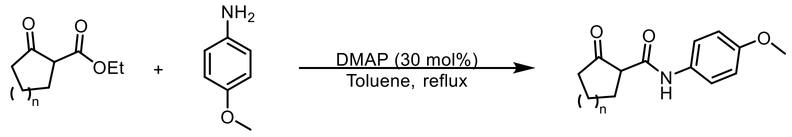

A mixture of corresponding amine/alcohol (20 mmol, 2 equiv), ethyl 2-oxocyclopentanecarboxylate (1.56 g, 10 mmol, 1 equiv), and DMAP (0.36 g, 0.3 mmol, 0.3 equiv) in toluene (20 mL) was heated to reflux for 20 h under argon. The solvent is then removed under reduced pressure and the residue is purified by flash chromatography on a silica gel column using EtOAc/Hexane (1:5) as eluent to offered the desired product.

1ad: 1.5 g, 64% yield, known compound. NMR data: ^1H NMR (400 MHz, Chloroform-*d*) δ 8.62 (s, 1H), 7.45 (d, J = 8.8 Hz, 2H), 6.86 (d, J = 8.7 Hz, 2H), 3.79 (s, 3H), 3.14 (t, J = 9.3 Hz, 1H), 2.51-2.29 (m, 4H), 2.14-2.07 (m, 1H), 1.93-1.84 (m, 1H).

1af: 1.8 g, 72% yield, known compound. NMR data: ^{1}H NMR (400 MHz, Chloroform-*d*) δ 9.21 (s, 1H), 7.42 (dd, *J* = 36.9, 8.7 Hz, 2H), 6.87 (t, *J* = 8.7 Hz, 2H), 3.79 (d, *J* = 3.4 Hz, 3H), 3.31 (dd, *J* = 10.8, 5.5 Hz, 1H), 2.68-2.38 (m, 2H), 2.37-2.20 (m, 2H), 2.18-1.91 (m, 2H), 1.84-1.74 (m, 2H).

1am: 2.4 g, 65% yield, ^{1}H NMR (400 MHz, Chloroform-*d*) δ 4.61-4.57 (m, 1H), 4.37-4.27 (m, 1H), 4.23-4.20 (m, 1H), 4.20-4.13 (m 1H), 3.90 (d, *J* = 13.0 Hz, 1H), 3.77-3.71 (m, 1H), 3.70-3.59 (m, 1H), 3.17 (t, *J* = 9.2 Hz, 1H), 2.40-2.20 (m, 3H), 2.17-2.11 (m, 1H), 1.93-1.76 (m, 1H), 1.52 (d, *J* = 2.6 Hz, 3H), 1.45 (d, *J* = 6.2 Hz, 3H), 1.41 (d, *J* = 12.2 Hz, 1H), 1.38 (d, *J* = 2.2 Hz, 2H), 1.35-1.28 (m, 3H). ^{13}C NMR (100 MHz, Chloroform-*d*) δ 211.7, 169.0, 168.6, 109.2, 109.2, 109.1, 109.0, 108.6, 103.2, 101.5, 101.4, 71.1, 70.9, 70.4, 70.3, 70.1, 66.0, 65.6, 65.6, 61.4, 61.3, 54.7, 54.6, 38.2, 38.1, 27.8, 27.2, 26.7, 26.6, 26.5, 26.0, 25.9, 25.4, 25.4, 25.4, 24.1, 24.1, 21.1, 20.9.

Cobaloximes **5a** and **5b** were prepared according to literature precedent.[2]

To a 100 mL round-bottom flask with stir bar, $CoCl_2$ hydrate (2.3 mmol, 0.54 g) and acetone (15 mL) were added and stirred resulting in a blue homogeneous solution. To this solution, the respective oxime ligand (7.5 mmol) was added and the color changed from blue to purple/pink. Air was bubbled to the mixture. After 1hour, green precipitate formed. The reaction mixture cooled to 0 °C and the green solid was filtered via vacuum filtration, then washed with 10 mL diethyl ether. This solid was then added to a 100 mL round-bottom flask with stir bar and taken up in MeOH (20 mL). To the stirring solution, DMAP was added (4.05mmol) and the solution turned brown immediately. The reaction mixture was allowed to stir for 30 min. After, stirringis stopped and the mixture was cooled to 0 °C. The resulting brown solid was collected via vacuum filtration and washed with water (10 mL) then diethyl ether (10 mL). The product was dried under vacuum to yield the desired cobaloxime **5a** (695 mg, 78% yield). **^{1}H NMR** (400 MHz, Acetonitrile-d_3) δ 18.57 (s, 2H), 7.47 (d, *J* = 7.8 Hz, 2H), 6.43 (d, *J* = 7.6 Hz, 2H), 2.93 (s, 6H), 2.33 (s, 12H).

In a 250 mL flask, 150 mL of degassed diethyl ether was added to a flask containing $Co(OAc)_2$•$4H_2O$ (2.0 g, 8 mmol) and dimethylglyoxime (1.9 g, 16 mmol), followed by freshly distilled BF_3•Et_2O (10 mL, excess). The mixture was stirred for 6 h under argon. The resulting solid was filtered under argon, washed with ice–cold water (3 × 10 mL, degassed) and air-dried. A brownish-red solid product **5b** was obtained (2.2 g) with 65% yield.

The aminocatalysts was synthesized according to the procedure reported previously by our group.[1,3]

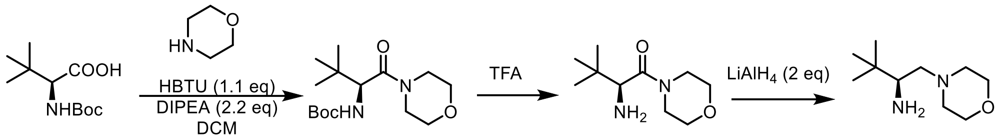

To a solution of N-Boc-L-tert-leucine (23.15 g, 100 mmol) in anhydrous CH_2Cl_2 (200 mL) at 0 °C, was added N,N-Diisopropylethylamine (DIPEA, 28.43 g, 220 mmol) followed by HBTU (41.72 g, 110 mmol). After stirring for 30 min at room temperature, morpholine (8.71 g, 100 mmol) in anhydrous CH_2Cl_2 (20 mL) was added under ice bath, and the solution was stirred at room temperature for an additional 12 h. The reaction mixture was washed with HCl aqueous solution (0.1 M), saturated $NaHCO_3$ and brine, then dried with anhydrous Na_2SO_4. After removal of the solvent, the residue was purified by chromatography with petroleum ether and ethyl acetate = 5:1 to obtain the desired product (29.1g, 97% yield).

To the obtained product in the last step was added 200 mL CH_2Cl_2, then 35 mL TFA at 0 °C and stirred 2h. The solvent was removed to 30 mL, and H_2O (20 mL) and CH_2Cl_2 (100 mL) were added. The pH value of the aqueous solution was adjusted to 14 by the addition of 3M NaOH solution under ice bath. The aqueous phase was extracted with CH_2Cl_2 (100 mL), and the organic layers were combined and dried with anhydrous Na_2SO_4. After removal of the solvent, the intermediate compound (15.1 g, 77% yield) was directly used for the next step without purification.

To the above-obtained intermediate (15.1 g) in anhydrous THF (150 mL) at 0 °C was added $LiAlH_4$ (5.70 g, 150 mmol) in portions, then the solution was stirred at room temperature for 10 minutes and heated to reflux for 12 h. After cooling to room temperature, 10 mL water was added at 0 °C, followed by 15% aqueous NaOH (10 mL), and anhydrous Na_2SO_4 after 10 minutes. The solid formed was filtered and washed with ethyl acetate several times. The solvent was removed, and pure catalyst (10.0 g, 54% overall yield) was obtained as a colorless oil by distillation under reduced pressure (80°C, 1 mm Hg). The spectroscopic data match those reported in the literature. **^{1}H NMR** (400 MHz, Chloroform-*d*) δ 3.77-3.61 (m, 4H), 2.70-2.48 (m, 3H), 2.36-2.21 (m, 3H), 2.18-2.04 (m, 1H), 1.43 (br., 2H), 0.86 (s, 9H). **^{13}C NMR** (100 MHz, Chloroform-*d*) δ 67.3, 61.1, 55.8, 54.2, 33.1, 26.3.

The synthesis of **4b** and **4c** were identical to this method.

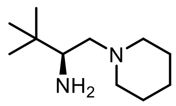

(S)-3,3-dimethyl-1-(piperidin-1-yl) butan-2-amine: 8.1g, 44% overall yield, known compound, **^{1}H NMR** (400 MHz, Chloroform-*d*) δ 2.59 (dd, *J* = 11.0, 2.5 Hz, 1H), 2.49 (d, *J* = 9.7 Hz, 2H), 2.25 – 2.13 (m, 3H), 2.03 (t, *J* = 11.5 Hz, 1H), 1.64-1.44 (m, 6H), 1.43-1.36 (m, 2H), 0.85 (s, 9H).

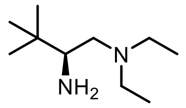

(S)-N1,N1-diethyl-3,3-dimethylbutane-1,2-diamine: 7.2g, 41% overall yield. known compound, ^{1}H NMR (400 MHz, Chloroform-*d*) δ 2.69-2.54 (m, 3H), 2.47-2.39 (m, 3H), 2.19-2.10 (m, 1H), 1.74 (s, 2H), 1.02 (t, *J* = 7.1 Hz, 6H), 0.91 (d, *J* = 1.0 Hz, 9H).

Preparation of **4a**: An oven-dried 100 mL flask was equipped with a magnetic stir bar, (S)-3,3-dimethyl-1-morpholinobutan-2-amine (930 mg, 5.0 mmol, 1.0 equiv) was dissolved in 40 mL anhydrous DCM and added to the flask. To anther flask was equipped with TfOH (750 mg, 5.0 mmol, 1.0 equiv) and 10 mL anhydrous DCM. The acid solution was added to the amine solution drop wise within 5 min under 0 ^{o}C condition. The mixture was stirred for 60 min under room temperature and then concentrated in vacuum to afford **4a** as white solid which was used without further purification.

4b and **4c** were produced using the same procedure.

[Ir(ppy)$_2$dtbbpy]PF$_6$ were purchased from commercial source and used without further purification. Alkenes were commercially available and used directly without further purification except the following substrates.

The following starting materials were prepared according to reported literature procedures:

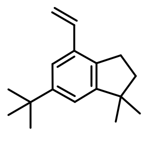

2ag

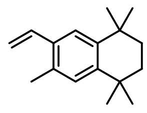

2ah

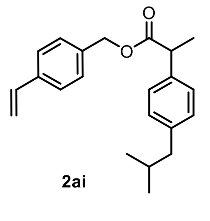

2ai

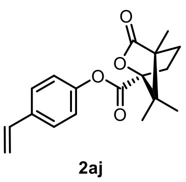

2aj

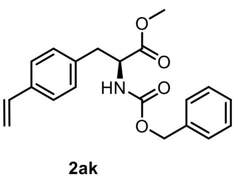

2ak

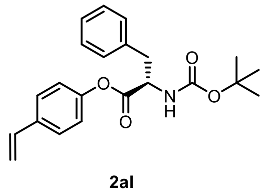

2al

Synthesis of 2ag and 2ah:[4,5]

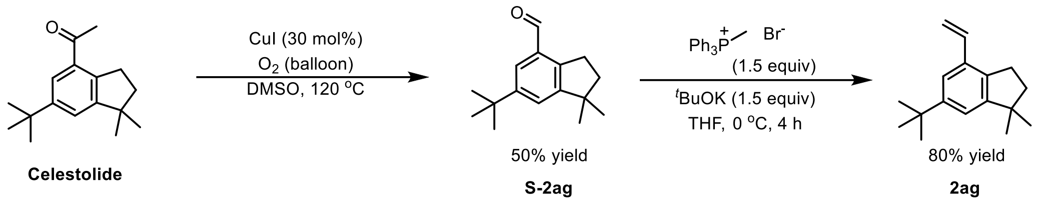

To a solution of Celestolide (20.0 mmol, 4.88 g) and CuI (6 mmol, 1.14 g) in DMSO (50 mL) under oxygen atmosphere (oxygen balloon). The reaction mixture was heated to 120 °C and stirred 72h (monitored by TLC). Upon cooling to room temperature, the reaction mixture was diluted with 100 mL of dichloromethane and 50 mL of water. After filtered through a pad of Celite, the organic and aqueous layers were separated, and the aqueous layer was extracted with dichloromethane (2×30 mL). The combined organic layers were reextracted with water (50 mL). Collected the organic layer, dried over $MgSO_4$ and filtered. The filtrate was concentrated in vacuum, and then the residue was purified by silica gel column chromatography (PE:EA = 20:1) to afford **S-2ag** (2.3 g, 50% yield).

Methyltriphenylphosphonium bromide (5.36 g, 15 mmol, 1.5 equiv.) was suspended in anhydrous THF (100 mL) and the mixture was cooled to 0 °C. Potassium *tert*-butoxide (1.68 g, 15 mmol, 1.5 equiv.) was slowly added to the reaction mixture under Ar atmosphere. The mixture was allowed to stir at 0 °C for another 30 min to obtain a deep red solution. The corresponding substituted aldehydes **S-2ai** (10 mmol) in anhydrous THF (10 mL) was added dropwise. After stirring under Ar conditions for 4 h, the reaction mixture was quenched by H_2O (50 mL). The organic layer was separated and the aqueous phase was extracted with DCM (2 × 50 mL). The combined organic solutions were washed with brine (60 mL), dried over Na_2SO_4, filtered, and concentrated. The crude product was purified by chromatography (silica gel, petroleum ether/EtOAc = 50:1) to afford the alkenes **2ag** as colorless oil (1.82 g, 80% yield). The synthesis of **2ah** was similar to this method.

Synthesis of 2ak:[6]

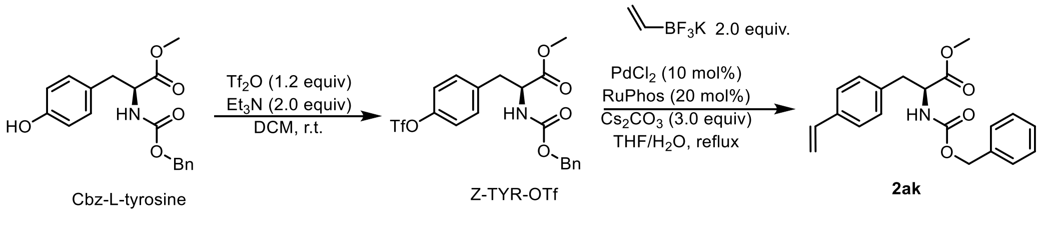

To a solution of Cbz-L-tyrosine derivative (1.58 g, 5.00 mmol) in anhydrous DCM (30 mL), triethylamine (1.3 mL, 10.0 mmol) and trifluoromethanesulfonic anhydride (1.0 mL, 6.0 mmol) were added dropwise at 0 °C. The resulting brown mixture was stirred for 5 hours while warming to room temperature gradually. The reaction terminated by adding aq. $NaHCO_3$ and the crude product was extracted with DCM. The combined organic layer was dried over $MgSO_4$. After evaporation, the concentrate was purified by silica gel column chromatography (PE:EA = 2:1) to obtain the desired product Z-TYR-OTf as a light-yellow solid (1.84 g, 80%).

The mixture of Z-TYR-OTf (1.84 g, 4.00 mmol), potassium trifluoroborate (1.07 g, 8.00 mmol), palladium (II) chloride (70.9 g, 0.4 mmol), RuPhos (373 mg, 0.8 mmol) and cesium carbonate (3.91 g, 12.0 mmol) was charged in round-bottom flask and it was vacuumed and filled with argon three times. 11.4 mL of THF/H_2O (8:1) was added and the resulting dark brown mixture was stirred at 85 °C for 24 hours. After adding More H_2O, the crude product was extracted with Et_2O. The combined organic layer was dried over $MgSO_4$. After evaporation, the crude product was purified by silica gel column chromatography (PE:EA = 4:1) to obtain the desired product **2ak** as white solid (1.0 g, 75%).

Synthesis of 2al and 2aj:[3]

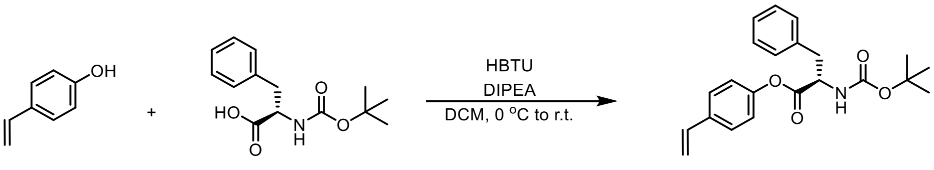

N-Boc-L-phenylalanine

2al

The **2al** were synthesized according to procedure reported in the literature.[3] To a solution of N-(tert-Butoxycarbonyl)-L-phenylalanine (30 mmol, 7.95 g) in DCM (100 mL) was added DIPEA (66 mmol, 1.0 equiv, 11.5 mL) and HBTU (33 mmol, 1.1 equiv, 12.51 g) at 0 °C. The reaction mixture was allowed to stir for 30 min followed by addition 4-hydroxystyrene (30 mmol, 1.0 equiv, 3.60 g). After stirring at room temperature overnight, the organic phase was washed with 0.1M HCl (50 mL), H_2O (50 mL), and sat. aq. $NaHCO_3$ (50 mL). The organic layer was dried over anhydrous Na_2SO_4 and concentrated in *vacuo*. The crude product was purified by flash chromatography (petroleum ether/EtOAc = 5:1) to afford the coupling ester of **2al** as white solid (3.60 g, 32% yield). The synthesis of **2aj** was similar to this method.

Synthesis of 2ai:[7,8]

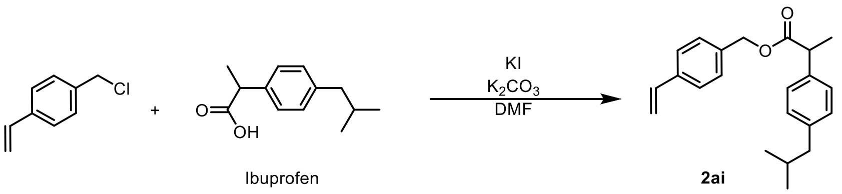

In a dry round-bottomed flask, the corresponding ibuprofen (10 mmol) was dissolved in DMF (50 mL). Then, K_2CO_3 (2.07 g, 15 mmol) and KI (2.49 g, 15 mmol) were added and stirred. To this stirring suspension, 4-vinylbenzyl chloride (1.68 g, 11 mmol) was added, and the reaction was stirred at room temperature for overnight. After the reaction was completed, 100 mL of ethyl acetate were added, followed by 10 mL of H_2O. The reaction mixture was then extracted with ethyl acetate (3 x50 mL), the combined organic phase was washed by H_2O (3 x 20 mL). Then the organic layer was dried with Na_2SO_4. After filtration and the solvent was removed under reduced pressure, the residue was purified by flash chromatography (eluent: PE/EA = 10:1) to afford the desired product **2ai** (2.8 g, 87% yield).

The difference of reactivity between Co(dmgBF$_2$)$_2$·2H$_2$O 5b and Co(dmgH$_2$)$_2$DMAPCl 5a.

Supplementary Table 9. Reversible addition to alkene:[11]

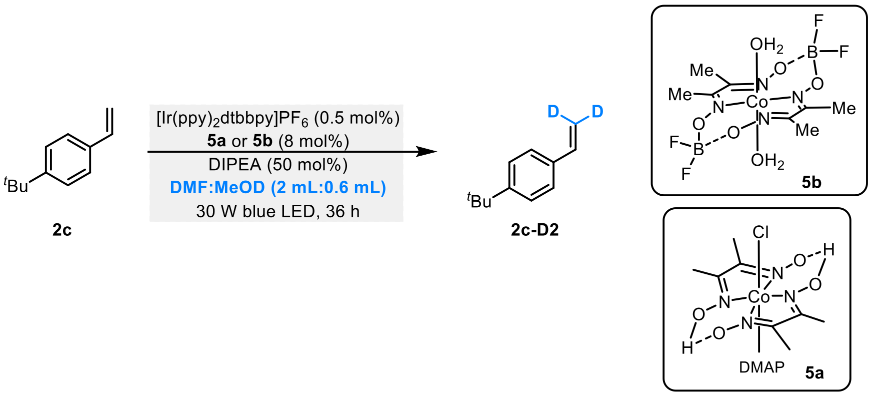

Entry	Cobalt	Yield **2c/2c-D2** (%)	**D** (%)
1	**5b**	91	0
15	**5a**	88	91

[a]Reactions were performed with **2c** (0.2 mmol), DIPEA (50 mol%), cobalt **5a** or **5b** (8 mol%), [Ir(ppy)$_2$dtbbpy]PF$_6$ (0.5 mol%), 0.6 mL of MeOD and 2.0 mL DMF, deaerated and irradiated for 36 h by a 30 W blue LED under room temperature. Yield with isolated product. Deuterium incorporation percentages was detected by ^{1}H NMR analysis.

Supplementary Table 10. Reduction to hydrogen acceptor:

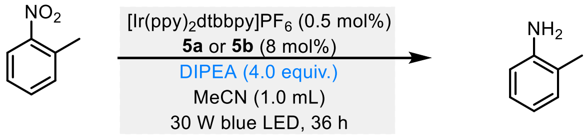

Entry	Cobalt	Conversion of substrate (%)	Yield of *o*-Toluidine (%)
1	**5b**	86	55
2	**5a**	95	32

[a]Reactions were performed with *o*-Me-$C_6H_4NO_2$ (0.1 mmol), DIPEA (4.0 equivalent), cobalt **5a** or **5b** (8 mol%), [Ir(ppy)$_2$dtbbpy]PF_6 (0.5 mol%), 1.0 mL MeCN, deaerated and irradiated for 36 h by a 30 W blue LED under room temperature. The yield and conversion were determined by GC analysis using 1,3,5-trimethoxybenzene as an internal standard.

3.3 Determination of Absolute Configuration:[9]

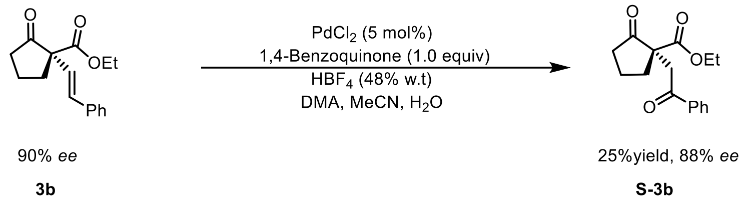

Palladium acetate (1.15 mg, 5 mol%) and benzoquinone (10.8 mg, 0.10 mmol) were charged in a resealable 10 mL vial under air. A mixture of DMA (0.22 mL), MeCN (0.22 mL) and water (63 µL) was added, followed by the addition of aqueous HBF_4 (18 µL, 48% in water, 0.14 mmol). After the addition of the **3b** (0.10 mmol, 90% *ee*), the homogenous reaction mixture was stirred for 16 h at room temperature. The crude reaction mixture was then diluted with brine (3 mL) and ether (3 mL), the phases were separated and the aqueous phase was further extracted with ether. The combined organic phases were then dried over Na_2SO_4, filtered, and evaporated in vacuo. The crude product was then further purified by column chromatography on silica gel using pentane/ether as eluent S-**3b** (25% yield, 88% ee) as a white solid. HPLC analysis: Daicel Chiralpak OJ-H, hexane/iso-propanol = 90:10, flow rate = 1.0 mL/min, λ= 240 nm, retention time: 16.12 (major) and 18.96 min (minor). The absolute configuration of product **3b** was determined according to the optical rotation of S-**3b** ($[\alpha]_D^{20}$ = -23.3 (c = 0.52, $CHCl_3$) compared with the α-alkylation product reported by our group.[10]

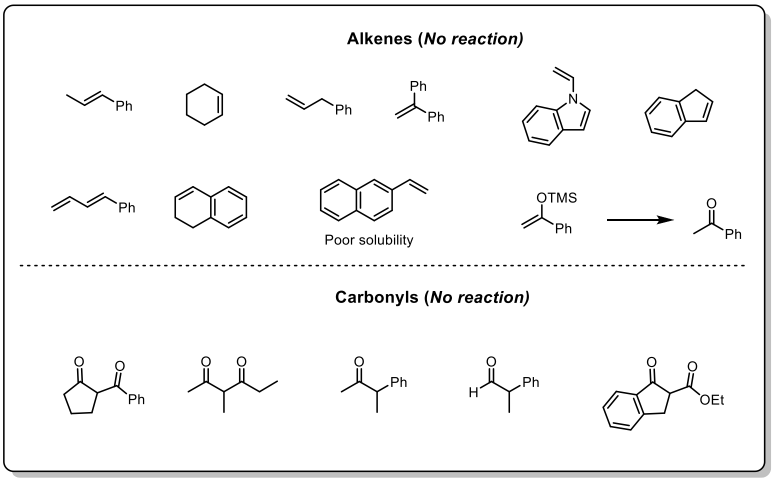

Supplementary Fig. 5. Unsuccessful examples. Upper: unreactive alkenes in the current reaction condition. Lower: unreactive carbonyls in the current reaction condition.

3.4 Emission quenching experiments

The fluorescence quenching experiments were carried out in degassed CH_3CN at room temperature upon excitation by 400 nm light.

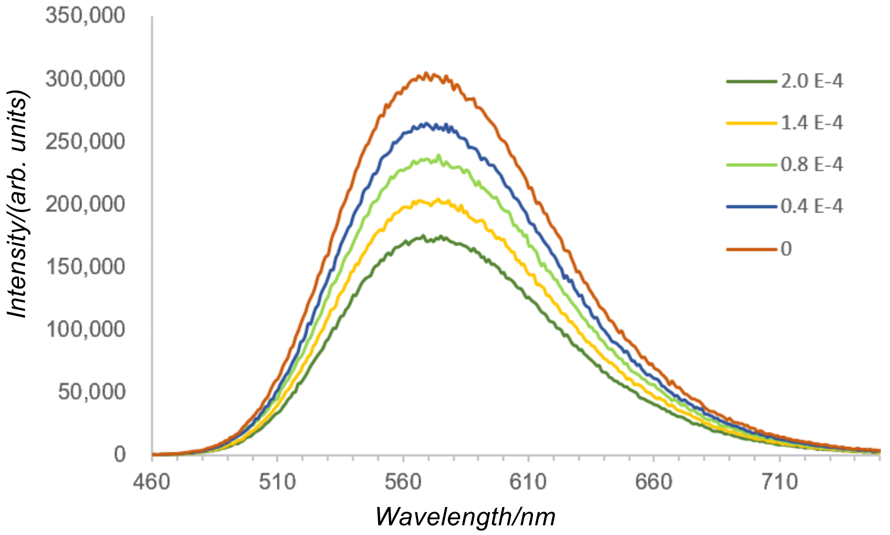

Supplementary Fig. 6. Fluorescence quenching experiments with cobalt 5a. The emission quenching of $[Ir(ppy)_2dtbbpy]PF_6$ (1.0×10^{-5} M) as a function of concentration of cobaloxime **5a** in deaerated CH_3CN with excitation at 400 nm.

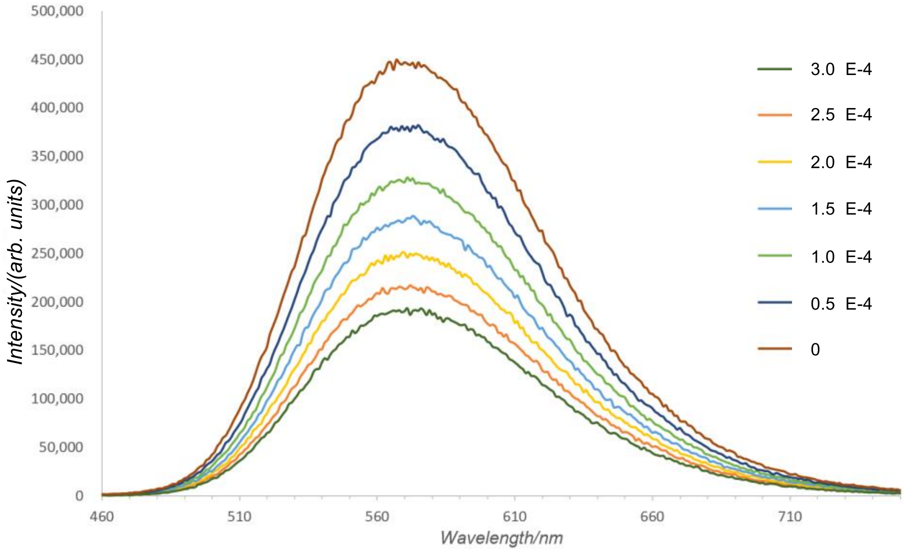

Supplementary Fig. 7. Fluorescence quenching experiments with cobalt 5b. The emission quenching of $[Ir(ppy)_2dtbbpy]PF_6$ (1.0×10^{-5} M) as a function of concentration of cobaloxime **5b** in deaerated CH_3CN with excitation at 400 nm.

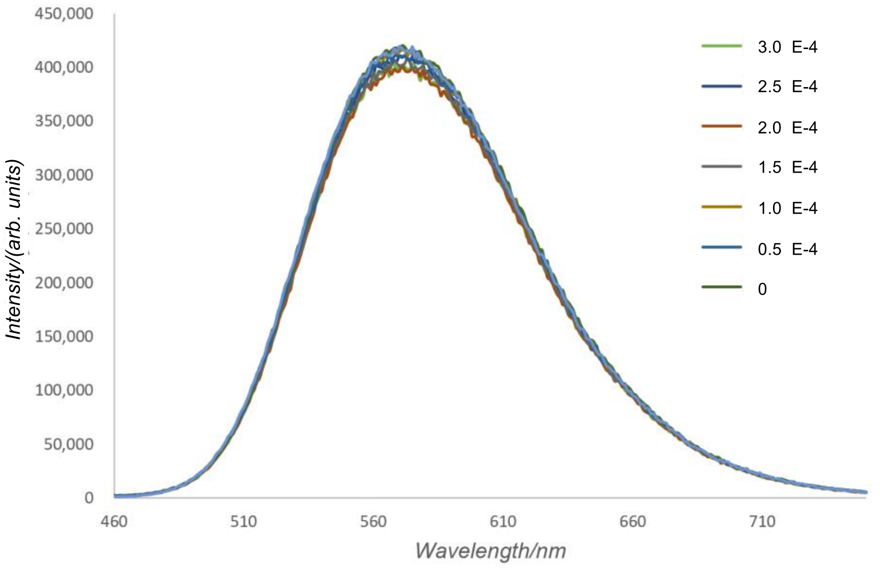

Supplementary Fig. 8. Fluorescence quenching experiments with enamine 6a. The emission quenching of [Ir(ppy)$_2$dtbbpy]PF$_6$ (1.0 × 10^{-5} M) as a function of concentration of enamine **6a** in deaerated CH_3CN with excitation at 400 nm.

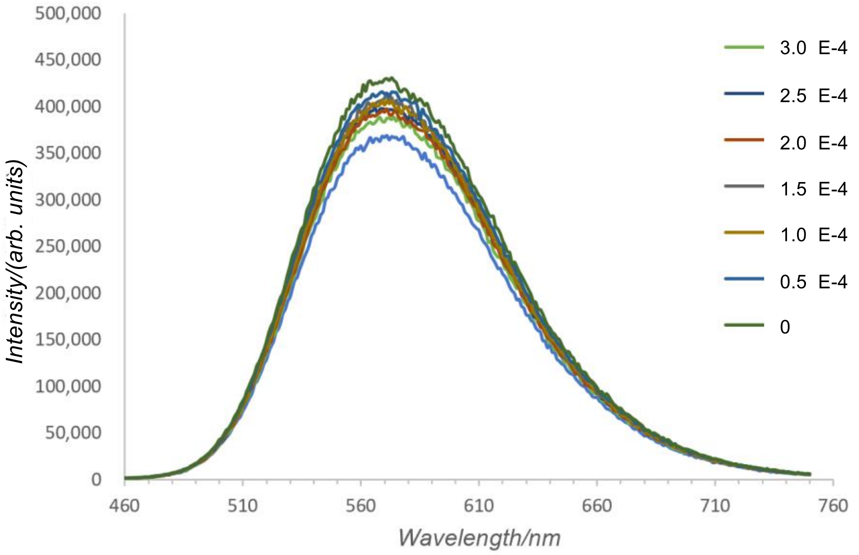

Supplementary Fig. 9. Fluorescence quenching experiments with *o*-Me-$C_6H_4NO_2$. The emission quenching of [Ir(ppy)$_2$dtbbpy]PF$_6$ (1.0 × 10^{-5} M) as a function of concentration of *o*-Me-$C_6H_4NO_2$ in deaerated CH_3CN with excitation at 400 nm.

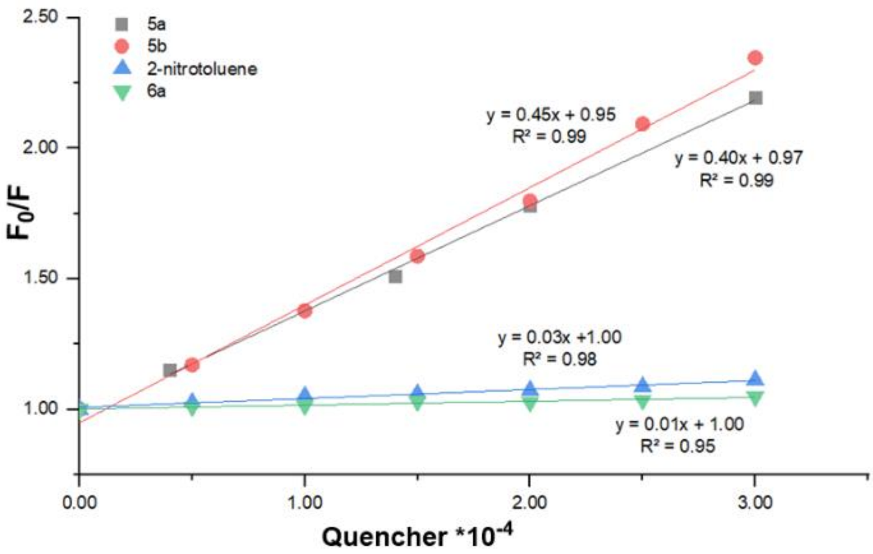

Supplementary Fig. 10. Stern-Volmer quenching experiments. Stern-Volmer plots of [Ir(ppy)$_2$dtbbpy]PF$_6$ using cobalt **5a**/**5b**, 2-nitrotoluene and enamine **6a** as quenchers in CH_3CN.

3.5 DFT calculations:

The computational details:

The pK_as of enamine **6a** and its radical cation were calculated based on our previous methods.[12] Geometry optimization were performed by using Gaussian 16.[13] As Truhlar et al.'s M06-2X hybrid functional was shown to provide accurate predictions of thermodynamic properties of organic molecules,[14,15] geometry optimizations and frequency computations were performed at the M06-2X/6-311G(d,p) level of theory. IEF-PCM model was used to account for the solvation effects in DMSO.[16-18] Thermal free energy corrections were obtained at 298.15 K to calculate pK_a values. Low frequencies (<100 cm^{-1}) contribution for vibrational entropy was corrected according to the quasi-harmonic approximation method of Grimme using the GoodVibes.[19]

The proton exchange method[17] was used to determine the acidities of enamines and enamine radical cations (Supplementary equation 2). The acidities of aniline (pK_a = 30.6) and its radical cation (pK_a = 6.5) in DMSO reported by Bordwell and Cheng were used as reference (Supplementary Fig. 11).[20]

$$pK_a(A-H^{+\cdot}) = pK_a(A-H) + F(E(A^-)-E(HA))/ln(10)RT \ (1)$$

$$HA(aq,1M) + PhNH^-(aq,1M) \rightarrow A^-(aq,1\,M) + PhNH_2(aq,1M)$$

$$\Delta G_{sol} = G_{sol}(PhNH_2) + G_{sol}(A^-) - G_{sol}(HA) - G_{sol}(PhNH^-)$$

$$pK_a(HA) = \frac{\Delta G_{sol}}{RTln10} + pK_a(PhNH_2) \ (2)$$

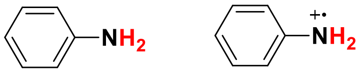

pK_a in DMSO: 30.6 6.5

Supplementary Fig. 11. Experimentally determined Radical cations' pK_a in DMSO. According to Bordwell-Cheng equation

Supplementary Table 11. The energies of the intermediates.

	E	ZPE	H	qh-H	T.S	T.qh-S	G(T)	qh-G(T)
EnH+	-1040.65	0.510524	-1040.12	-1040.12	0.079865	0.075294	-1040.2	-1040.2
En	-1040.2	0.494007	-1039.68	-1039.69	0.080794	0.075753	-1039.76	-1039.76
EnA	-1039.69	0.479097	-1039.18	-1039.19	0.079961	0.075354	-1039.26	-1039.26
EnRC	-1040	0.494273	-1039.48	-1039.49	0.081298	0.076489	-1039.56	-1039.56
EnR	-1039.55	0.48	-1039.04	-1039.04	0.080463	0.076052	-1039.12	-1039.12
PAR	-286.898	0.104257	-286.788	-286.788	0.035809	0.035814	-286.824	-286.824
PARC	-287.348	0.117716	-287.224	-287.224	0.036665	0.036658	-287.261	-287.261
PA	-287.556	0.117809	-287.431	-287.431	0.03572	0.035728	-287.467	-287.467
PAA	-287.034	0.103656	-286.924	-286.924	0.035205	0.035207	-286.959	-286.959
PAHC	-287.989	0.132861	-287.849	-287.849	0.036711	0.036634	-287.886	-287.886

4. Characterization of Compounds:

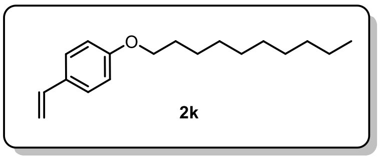

2k

2k: colorless oil, 1.43 g, 55% yield. ^{1}H NMR (400 MHz, Chloroform-*d*) δ 7.33 (d, *J* = 8.7 Hz, 2H), 6.85 (d, *J* = 8.7 Hz, 2H), 6.66 (dd, *J* = 17.6, 10.9 Hz, 1H), 5.60 (d, *J* = 17.6 Hz, 1H), 5.11 (d, *J* = 10.8 Hz, 1H), 3.95 (t, *J* = 6.6 Hz, 2H), 1.84-1.72 (m, 2H), 1.48-1.41 (m, 2H), 1.36-1.27 (m, 12H), 0.88 (t, *J* = 6.7 Hz, 3H).^{13}C NMR (100 MHz, Chloroform-*d*) δ 159.1, 136.4, 130.3, 127.5, 114.6, 111.5, 68.2, 32.0, 29.7, 29.7, 29.6, 29.5, 29.4, 26.2, 22.8, 14.3. IR (thin film, cm^{-1}): 3086, 3041, 2919, 2851, 1606, 1510, 1248, 1175, 837. HRMS (ESI) calcd. for $C_{18}H_{28}OH^+$: 261.2213, found: 261.2206.

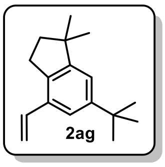

2ag

2ag: white solid, 1.82 g, 80% yield. ^{1}H NMR (400 MHz, Chloroform-*d*) δ 7.40 (s, 1H), 7.15 (s, 1H), 6.86 (dd, *J* = 17.6, 11.1 Hz, 1H), 5.74 (d, *J* = 17.7 Hz, 1H), 5.33 (d, *J* = 11.1 Hz, 1H), 2.95 (t, *J* = 7.2 Hz, 2H), 1.99 (t, *J* = 7.2 Hz, 2H), 1.39 (s, 9H), 1.31 (s, 6H).^{13}C NMR (100 MHz, Chloroform-*d*) δ 153.1, 150.1, 138.1, 135.7, 133.1, 120.4, 118.6, 114.6, 44.1, 41.5, 34.9, 31.8, 28.9, 28.6. IR (thin film, cm^{-1}): 3085, 3060, 2952, 2862, 1460, 1393, 990, 903, 875, 693. HRMS (ESI) calcd. for $C_{17}H_{24}H^+$: 229.1951, found: 229.1988.

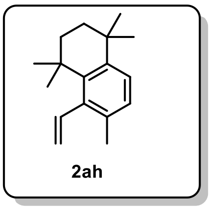

2ah

2ah: white solid, 1.48 g, 65% yield. ^{1}H NMR (400 MHz, Chloroform-d) δ 7.42 (s, 1H), 7.14 (s, 1H), 6.91 (dd, *J* = 17.4, 11.0 Hz, 1H), 5.62 (dd, *J* = 17.5, 1.4 Hz, 1H), 5.26 (dd, *J* = 11.0, 1.5 Hz, 1H), 2.33 (s, 3H), 1.96-1.82 (m, 1H), 1.65 (t, *J* = 13.2 Hz, 1H), 1.44-1.24 (m, 12H), 1.08 (s, 3H), 1.00 (d, *J* = 6.8 Hz, 3H). ^{13}C NMR (100 MHz, Chloroform-d) δ 145.9, 142.6, 135.2, 134.3, 132.7, 128.8, 123.6, 114.5, 43.9, 37.7, 34.7, 34.2, 32.6, 32.2, 28.7, 25.1, 19.7, 17.0. IR (thin film, cm^{-1}): 3088, 3055, 2960, 2871, 1470, 975, 900, 865, HRMS (ESI) calcd. for $C_{17}H_{24}H^+$: 229.1951, found: 229.1948.

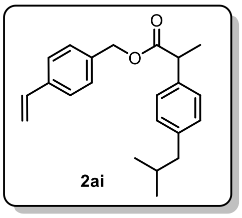

2ai

2ai: white solid, 2.8 g, 87% yield. ^{1}H NMR (400 MHz, Chloroform-*d*) δ 7.36 (d, *J* = 8.2 Hz, 2H), 7.23 (q, *J* = 9.6, 9.1 Hz, 4H), 7.11 (d, *J* = 8.0 Hz, 2H), 6.71 (dd, *J* = 17.6, 10.9 Hz, 1H), 5.76 (d, *J* = 17.6 Hz, 1H), 5.27 (d, *J* = 10.9 Hz, 1H), 5.11 (d, *J* = 2.0 Hz, 2H), 3.77 (q, *J* = 7.2 Hz, 1H), 2.48 (d, *J* = 7.2 Hz,

2H), 1.91-1.84 (m, 1H), 1.53 (d, *J* = 7.1 Hz, 3H), 0.93 (d, *J* = 6.6 Hz, 6H).^{13}C NMR (100 MHz, Chloroform-*d*) δ 174.6, 140.7, 137.7, 137.5, 136.5, 135.7, 129.4, 128.1, 127.3, 126.4, 114.3, 66.1, 45.3, 45.1, 30.3, 22.5, 18.5. IR (thin film, cm^{-1}): 3088, 3052, 3008, 2953, 1732, 1513, 1154, 908, 825, 549, 443. HRMS (ESI) calcd. for $C_{22}H_{26}O_2H^+$: 323.2006, found: 323.2011.

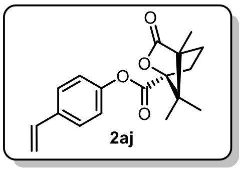

2aj: white solid, 2.25 g, 25% yield, $[\alpha]_D^{20}$ = -21.9 (c = 1.00, $CHCl_3$). ^{1}H NMR (400 MHz, Chloroform-*d*) δ 7.43 (d, *J* = 8.6 Hz, 2H), 7.09 (d, *J* = 8.6 Hz, 2H), 6.70 (dd, *J* = 17.6, 10.9 Hz, 1H), 5.77-5.68 (m, 1H), 5.32-5.23 (m, 1H), 2.60-2.53 (m, 1H), 2.23-2.16 (m, 1H), 2.03-1.96 (m, 1H), 1.80-1.73 (m, 1H), 1.19-1.08 (m, 9H).^{13}C NMR (100 MHz, Chloroform-*d*) δ 178.0, 166.2, 149.6, 136.1, 135.8, 127.4, 121.5, 114.6, 91.0, 55.0, 54.8, 30.9, 29.1, 17.0, 9.9. IR (thin film, cm^{-1}): 3056, 2973, 2935, 2877, 1788, 1505, 1264, 1166, 1048, 732. HRMS (ESI) calcd. for $C_{18}H_{20}O_4Na^+$: 323.1254, found: 323.1245.

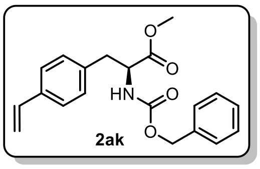

2ak: white solid, 1.0 g, 75% yield, $[\alpha]_D^{20}$ = +26.4 (c = 1.00, $CHCl_3$). ^{1}H NMR (400 MHz, Chloroform-*d*) δ 7.44-7.28 (m, 7H), 7.06 (d, *J* = 8.1 Hz, 2H), 6.68 (dd, *J* = 17.6, 10.9 Hz, 1H), 5.72 (d, *J* = 17.6 Hz, 1H), 5.24 (d, *J* = 11.0 Hz, 1H), 5.14-5.05 (m, 2H), 4.67 (q, *J* = 6.0 Hz, 1H), 3.73 (s, 3H), 3.16-3.05 (m, 2H).^{13}C NMR (100 MHz, Chloroform-*d*) δ 172.0, 155.7, 136.6, 136.5, 136.3, 135.4, 129.6, 128.6, 128.3, 128.2, 126.6, 113.9, 67.1, 54.9, 52.5, 38.1. IR (thin film, cm^{-1}): 3333, 3064, 3033, 2952, 1715, 1511, 1209, 1055, 908, 737, 697. HRMS (ESI) calcd. for $C_{20}H_{21}NO_4Na^+$: 362.1363, found: 362.1352.

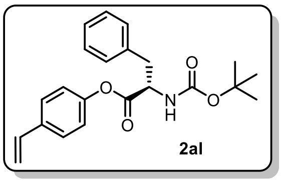

2al: white solid, 3.6 g, 32% yield, $[\alpha]_D^{20}$ = -14.8 (c = 1.00, $CHCl_3$). ^{1}H NMR (400 MHz, Chloroform-*d*) δ 7.40 (d, *J* = 8.3 Hz, 2H), 7.32 (dd, *J* = 15.1, 7.3 Hz, 3H), 7.25 (d, *J* = 10.1 Hz, 2H), 6.99-6.92 (m, 2H), 6.69 (dd, *J* = 17.6, 10.9 Hz, 1H), 5.71 (d, *J* = 17.6 Hz, 1H), 5.25 (d, *J* = 10.9 Hz, 1H), 5.07 (d, *J* = 8.3 Hz, 1H), 4.82 (q, *J* = 6.7 Hz, 1H), 3.24 (d, *J* = 6.1 Hz, 2H), 1.45 (s, 9H).^{13}C NMR (100 MHz, Chloroform-*d*) δ 170.7, 155.3, 150.0, 135.9, 135.8, 129.6, 128.9, 127.4, 127.3, 121.5, 114.4, 80.3, 54.8, 38.5, 28.4. IR (thin film, cm^{-1}): 3428, 3351, 3004, 2931, 1761, 1709, 1504, 1365, 1162, 699. HRMS (ESI) calcd. for $C_{22}H_{25}NO_4Na^+$: 390.1676, found: 390.1665.

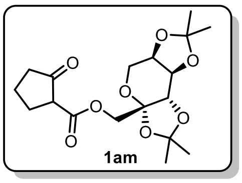

1am: prepared according to previous reported,[3] ropy solid, 2.4 g, 65% yield, $[\alpha]_D^{20}$ = -30.4 (c = 1.00, $CHCl_3$). ^{1}H NMR (400 MHz, Chloroform-*d*) δ 4.61-4.57 (m, 1H), 4.37-4.27 (m, 1H), 4.23-4.20 (m, 1H), 4.20-4.13 (m 1H), 3.90 (d, *J* = 13.0 Hz, 1H), 3.77-3.71 (m, 1H), 3.70-3.59 (m, 1H), 3.17 (t, *J* = 9.2 Hz, 1H), 2.40-2.20 (m, 3H), 2.17-2.11 (m, 1H), 1.93-1.76 (m, 1H), 1.52 (d, *J* = 2.6 Hz, 3H), 1.45 (d, *J* = 6.2 Hz, 3H), 1.41 (d, *J* = 12.2 Hz,, 1H), 1.38 (d, *J* = 2.2 Hz, 2H), 1.35-1.28 (m, 3H).^{13}C NMR (100 MHz, Chloroform-*d*) δ 211.7, 169.0, 168.6, 109.2, 109.2, 109.1, 109.0, 108.6, 103.2, 101.5, 101.4, 71.1, 70.9, 70.4, 70.3, 70.1, 66.0, 65.6, 65.6, 61.4, 61.3, 54.7, 54.6, 38.2, 38.1, 27.8, 27.2, 26.7, 26.6, 26.5, 26.0, 25.9, 25.4, 25.4, 25.4, 24.1, 24.1, 21.1, 20.9. IR (thin film, cm^{-1}): 3501, 2988, 2938, 1731, 1377, 1251, 1209, 1068, 865, 757. HRMS (ESI) calcd. for $C_{18}H_{26}O_8Na^+$: 393.1520, found: 393.1514.

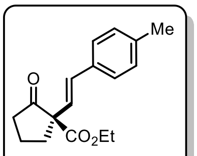

3a-*E*

3a-*E*: The compound was synthesized according to the general procedure, using ethyl 2-oxocyclopentanecarboxylate (15.6 mg, 0.1 mmol) and corresponding alkene (59 mg, 0.5 mmol). The product was purified by column chromatography (PE/EA=5:1), colorless oil, 18.2 mg, 67% yield, 93% *ee*, $[\alpha]_D^{20}$ = -38.9 (c = 2.50, $CHCl_3$). HPLC analysis: Daicel Chiralpak IC, hexane/iso-propanol = 98:2, flow rate = 1.0 mL/min, λ = 254 nm, retention time: 29.90 min (major) and 31.91 min (minor). ^{1}H NMR (400 MHz, Chloroform-*d*) δ 7.33-7.24 (m, 2H), 7.12 (d, *J* = 7.8 Hz, 2H), 6.48 (d, *J* = 16.3 Hz, 1H), 6.35 (d, *J* = 16.3 Hz, 1H), 4.20 (q, *J* = 7.2 Hz, 2H), 2.75-2.68 (m, 1H), 2.51-2.33 (m, 3H), 2.33 (s, 2H), 2.30-2.25 (m, 1H), 2.13-1.91 (m, 2H), 1.26 (t, *J* = 7.1 Hz, 3H).^{13}C NMR (100 MHz, Chloroform-*d*) δ 212.5, 170.5, 138.0, 133.8, 131.7, 129.4, 126.6, 124.9, 63.0, 62.0, 37.7, 33.6, 21.3, 19.7, 14.2. IR (thin film, cm^{-1}): 3086, 2976, 2923, 1720, 1230, 1025, 970, 795, 721, 507. HRMS (ESI) calcd. for $C_{17}H_{20}O_3H^+$: 273.1485, found: 273.1477.

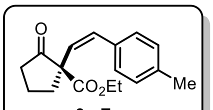

3a-*Z*

3a-*Z*: The compound was synthesized according to the general procedure with $[Ir(dF\text{-}ppy)_2dtbbpy]PF_6$ as catalyst, using ethyl 2-oxocyclopentanecarboxylate (15.6 mg, 0.1 mmol) and corresponding alkene (59 mg, 0.5 mmol). The product was purified by column chromatography (PE/EA=5:1), colorless oil, 10.4 mg, 37% yield, 92% *ee*, $[\alpha]_D^{20}$ = -38.9 (c = 2.50, $CHCl_3$). HPLC analysis: Daicel Chiralpak IC, hexane/iso-propanol = 98:2, flow rate = 1.0 mL/min, λ = 248 nm, retention time: 21.84 min (major) and 23.41 min (minor). ^{1}H NMR (400 MHz, Chloroform-*d*) δ 7.17-7.08 (m, 4H), 6.68 (d, *J* = 12.0 Hz, 1H), 5.80 (d, *J* = 12.0 Hz, 1H), 3.98 (q, *J* = 7.3 Hz, 2H), 2.66-2.53 (m, 1H), 2.51-2.35 (m, 1H), 2.33 (s, 3H), 2.28-2.14 (m, 1H), 1.97-1.78 (m, 3H), 1.15 (t, *J* = 7.1 Hz, 3H). ^{13}C NMR (100 MHz, Chloroform-*d*) δ 213.3, 170.0, 137.3, 134.0, 133.1, 128.8, 128.6, 128.2, 62.7, 61.9, 37.3, 35.6, 21.4, 19.7, 14.0. IR (thin film, cm^{-1}): 3086, 2976, 2923, 1720, 1230, 1025, 970, 795, 721, 507. HRMS (ESI) calcd. for $C_{17}H_{20}O_3H^+$: 273.1485, found: 273.1477.

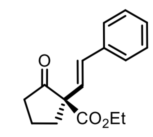

3b

3b: The compound was synthesized according to the general procedure, using ethyl 2-oxocyclopentanecarboxylate (15.6 mg, 0.1 mmol) and corresponding alkene (52 mg, 0.5 mmol). The product was purified by column chromatography (PE/EA=5:1), colorless oil, 18.1 mg, 70% yield, 90% *ee*, $[\alpha]_D^{20}$ = -38.0 (c = 1.80, $CHCl_3$). HPLC analysis: Daicel Chiralpak OJ-H, hexane/iso-propanol = 95:5, flow rate = 1.0 mL/min, λ = 254 nm, retention time: 20.92 min (minor) and 23.82 min (major). ^1H NMR (400 MHz, Chloroform-*d*) δ 7.41 (d, *J* = 8.2 Hz, 2H), 7.33 (t, *J* = 7.8 Hz, 2H), 7.27 (d, *J* = 6.6 Hz, 1H), 6.54 (d, *J* = 16.3 Hz, 1H), 6.43 (d, *J* = 16.4 Hz, 1H), 4.22 (q, *J* = 7.2 Hz, 2H), 2.78-2.71 (m, 1H), 2.55-2.25 (m, 3H), 2.18-1.96 (m, 2H), 1.28 (t, *J* = 7.1 Hz, 3H).^{13}C NMR (100 MHz, Chloroform-*d*) δ 212.4, 170.4, 136.6, 131.8, 128.7, 128.1, 126.7, 126.0, 63.0, 62.0, 37.7, 33.6, 19.7, 14.2. IR (thin film, cm^{-1}): 3436, 3063, 3034, 2976, 1725, 1096, 1012, 748, 699, 505. HRMS (ESI) calcd. for $C_{16}H_{18}O_3Na^+$: 281.1148, found: 281.1140.

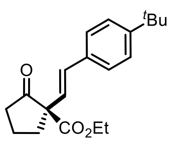

3c

3c: The compound was synthesized according to the general procedure, using ethyl 2-oxocyclopentanecarboxylate (15.6 mg, 0.1 mmol) and corresponding alkene (80 mg, 0.5 mmol). The product was purified by column chromatography (PE/EA=5:1), colorless oil, 23.2 mg, 74% yield, 93% *ee*, $[\alpha]_D^{20}$ = -19.7 (c = 1.75, $CHCl_3$). HPLC analysis: Daicel Chiralpak AD-H, hexane/iso-propanol = 98:2, flow rate = 1.0 mL/min, λ = 254 nm, retention time: 11.69 min (minor) and 13.28 min (major). ^1H NMR (400 MHz, Chloroform-*d*) δ 7.34 (s, 4H), 6.49 (d, *J* = 16.3 Hz, 1H), 6.37 (d, *J* = 16.4 Hz, 1H), 4.19 (q, *J* = 7.7, 7.1 Hz, 2H), 2.75-2.68 (m, 1H), 2.51-2.22 (m, 3H), 2.09-1.91 (m, 2H), 1.31 (s, 9H), 1.26 (t, *J* = 7.1 Hz, 3H).^{13}C NMR (100 MHz, Chloroform-*d*) δ 212.5, 170.5, 151.3, 133.8, 131.5, 126.4, 125.6, 125.1, 63.0, 62.0, 37.6, 34.7, 33.5, 31.4, 19.7, 14.2. IR (thin film, cm^{-1}): 2962, 2905, 2869, 1719, 1219, 1109, 970, 735, 557. HRMS (ESI) calcd. for $C_{20}H_{26}O_3Na^+$: 337.1774, found: 337.1766.

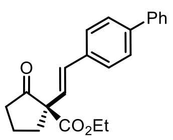

3d

3d: The compound was synthesized according to the general procedure, using ethyl 2-oxocyclopentanecarboxylate (15.6 mg, 0.1 mmol) and corresponding alkene (90 mg, 0.5 mmol). The product was purified by column chromatography (PE/EA=5:1), white solid, 23.0 mg, 67% yield, 92% *ee*, $[\alpha]_D^{20}$ = +74.5 (c = 2.20, $CHCl_3$). HPLC analysis: Daicel Chiralpak AD-H, hexane/iso-propanol = 98:2, flow rate = 1.0 mL/min, λ = 254 nm, retention time: 13.68 min (major) and 14.84 min (minor). ^1H NMR (400 MHz, Chloroform-*d*) δ 7.64-7.49 (m, 4H), 7.49-7.42 (m, 2H), 7.35 (t, *J* = 8.2 Hz, 3H), 6.75 (d, *J* = 12.1 Hz, 1H), 5.84 (s, 1H), 4.01-3.94 (m, 2H), 2.72-2.58 (m, 1H), 2.54-2.41 (m, 1H), 2.31-2.17 (m, 1H), 2.03-1.82 (m, 3H), 1.14 (t, *J* = 7.1 Hz, 3H).^{13}C NMR (100 MHz, Chloroform-*d*) δ 213.2, 169.9,

140.7, 140.2, 136.0, 132.8, 129.1, 128.9, 128.9, 127.5, 127.1, 126.7, 62.7, 62.0, 37.3, 35.8, 19.7, 14.0. IR (thin film, cm^{-1}): 3057, 3014, 2977, 1752, 1715, 1487, 1221, 772, 749, 698. HRMS (ESI) calcd. for $C_{22}H_{22}O_3Na^+$: 357.1461, found: 357.1452.

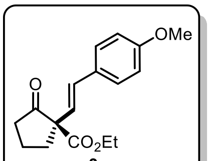

3e

3e: The compound was synthesized according to the general procedure, using ethyl 2-oxocyclopentanecarboxylate (15.6 mg, 0.1 mmol) and corresponding alkene (67 mg, 0.5 mmol). The product was purified by column chromatography (PE/EA=2:1), colorless oil, 10.1 mg, 35% yield, 94% *ee*, $[\alpha]_D^{20}$ = -5.5 (c = 2.00, $CHCl_3$). HPLC analysis: Daicel Chiralpak OD-H, hexane/iso-propanol = 95:5, flow rate = 1.0 mL/min, λ = 287 nm, retention time: 9.73 min (major) and 11.03 min (minor). ^{1}H NMR (400 MHz, Chloroform-*d*) δ 7.36-7.30 (m, 2H), 6.93-6.79 (m, 2H), 6.45 (d, *J* = 16.3 Hz, 1H), 6.26 (d, *J* = 16.3 Hz, 1H), 4.23-4.16 (m, 2H), 3.80 (s, 3H), 2.74-2.67 (m, 1H), 2.50-2.34 (m, 2H), 2.35-2.23 (m, 1H), 2.10-1.94 (m, 2H), 1.26 (t, *J* = 7.1 Hz, 3H).^{13}C NMR (100 MHz, Chloroform-*d*) δ 212.6, 170.6, 159.6, 131.3, 129.4, 127.9, 123.6, 114.1, 63.0, 62.0, 55.4, 37.7, 33.6, 19.7, 14.2. IR (thin film, cm^{-1}): 3035, 2965, 2906, 2837, 1721, 1511, 1246, 1173, 1029, 523. HRMS (ESI) calcd. for $C_{17}H_{20}O_4Na^+$: 311.1254, found: 311.1247.

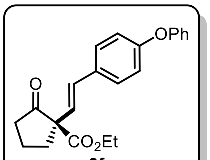

3f

3f: The compound was synthesized according to the general procedure, using ethyl 2-oxocyclopentanecarboxylate (15.6 mg, 0.1 mmol) and corresponding alkene (99 mg, 0.5 mmol). The product was purified by column chromatography (PE/EA=4:1), colorless oil, 18.2 mg, 52% yield, 91% *ee*, $[\alpha]_D^{20}$ = -16.3 (c = 1.30, $CHCl_3$). HPLC analysis: Daicel Chiralpak AS-H, hexane/iso-propanol = 95:5, flow rate = 1.0 mL/min, λ = 267 nm, retention time: 8.29 min (minor) and 10.02 min (major). ^{1}H NMR (400 MHz, Chloroform-*d*) δ 7.34 (dd, *J* = 14.3, 8.4 Hz, 4H), 7.14-7.08 (m, 1H), 6.98 (dd, *J* = 20.4, 8.1 Hz, 4H), 6.49 (d, *J* = 16.3 Hz, 1H), 6.32 (d, *J* = 16.3 Hz, 1H), 4.24-4.17 (m, 2H), 2.75-2.69 (dt, *J* = 13.6, 7.0 Hz, 1H), 2.52-2.41 (m, 1H), 2.41-2.23 (m, 3H), 2.15-1.92 (m, 2H), 1.27 (t, *J* = 7.1 Hz, 3H).^{13}C NMR (100 MHz, Chloroform-*d*) δ 212.5, 170.5, 157.3, 157.1, 131.7, 131.0, 129.9, 128.1, 125.0, 123.6, 119.1, 119.0, 63.0, 62.0, 37.7, 33.6, 19.7, 14.2. IR (thin film, cm^{-1}): 3065, 3040, 2979, 1726, 1588, 1507, 1488, 1237, 751, 693. HRMS (ESI) calcd. for $C_{22}H_{22}O_4Na^+$: 373.1410, found: 373.1402.

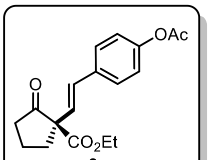

3g

3g: The compound was synthesized according to the general procedure, using ethyl 2-oxocyclopentanecarboxylate (15.6 mg, 0.1 mmol) and corresponding alkene (81 mg, 0.5 mmol). The product was purified by column chromatography (PE/EA=2:1), colorless oil, 9,5 mg, 30% yield, 92% *ee*, $[\alpha]_D^{20}$ = -17.8 (c = 1.40, $CHCl_3$). HPLC analysis: Daicel Chiralpak OD-H, hexane/iso-propanol = 95:5,

flow rate = 1.0 mL/min, λ = 254 nm, retention time: 14.48 min (major) and 15.89 min (minor). ^{1}H NMR (400 MHz, Chloroform-*d*) δ 7.39 (d, *J* = 8.5 Hz, 2H), 7.04 (d, *J* = 8.5 Hz, 2H), 6.49 (d, *J* = 16.3 Hz, 1H), 6.36 (d, *J* = 16.4 Hz, 1H), 4.19 (q, *J* = 7.0 Hz, 2H), 2.75-2.68 (m, 1H), 2.49-2.20 (m, 6H), 2.15-1.91 (m, 2H), 1.26 (t, *J* = 7.1 Hz, 3H). ^{13}C NMR (100 MHz, Chloroform-*d*) δ 212.3, 150.5, 134.4, 130.8, 127.7, 126.3, 121.8, 63.0, 62.0, 37.6, 33.6, 21.2, 19.7, 14.2. IR (thin film, cm^{-1}): 3038, 2979, 2941, 2906, 1722, 1506, 1369, 1191, 911. HRMS (ESI) calcd. for $C_{18}H_{20}O_5H^+$: 317.1384, found: 317.1375.

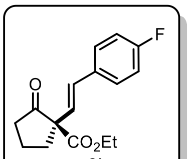

3h

3h: The compound was synthesized according to the general procedure, using ethyl 2-oxocyclopentanecarboxylate (15.6 mg, 0.1 mmol) and corresponding alkene (61 mg, 0.5 mmol). The product was purified by column chromatography (PE/EA=5:1), colorless oil, 16.8 mg, 60% yield, 90% *ee*, $[\alpha]_D^{20}$ = -33.8 (c = 0.66, $CHCl_3$). HPLC analysis: Daicel Chiralpak OJ-H, hexane/iso-propanol = 95:5, flow rate = 1.0 mL/min, λ = 254 nm, retention time: 19.45 min (minor) and 21.41 min (major). ^{1}H NMR (400 MHz, Chloroform-*d*) δ 7.36 (dd, *J* = 8.6, 5.5 Hz, 2H), 7.00 (t, *J* = 8.6 Hz, 2H), 6.47 (d, *J* = 16.3 Hz, 1H), 6.32 (d, *J* = 16.3 Hz, 1H), 4.20 (q, *J* = 7.2 Hz, 2H), 2.75-2.68 (m, 1H), 2.55-2.19 (m, 3H), 2.15-1.92 (m, 2H), 1.26 (t, *J* = 7.1 Hz, 3H). ^{19}F NMR (376 MHz, $CDCl_3$) δ -113.92. ^{13}C NMR (100 MHz, Chloroform-*d*) δ 212.4, 170.4, 163.9, 161.4, 132.7, 132.7, 130.7, 128.3, 128.2, 125.8, 125.7, 115.7, 115.5, 62.9, 62.1, 37.7, 33.6, 19.7, 14.2. IR (thin film, cm^{-1}): 3437, 2978, 2942, 2909, 1725, 1510, 1228, 833, 518. HRMS (ESI) calcd. for $C_{16}H_{17}FO_3Na^+$: 299.1054, found: 299.1046.

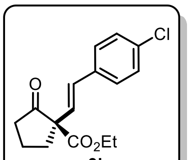

3i

3i: The compound was synthesized according to the general procedure, using ethyl 2-oxocyclopentanecarboxylate (15.6 mg, 0.1 mmol) and corresponding alkene (69 mg, 0.5 mmol). The product was purified by column chromatography (PE/EA=5:1), colorless oil, 12.3 mg, 42% yield, 87% *ee*, $[\alpha]_D^{20}$ = -34.4 (c = 1.10, $CHCl_3$). HPLC analysis: Daicel Chiralpak OJ-H, hexane/iso-propanol = 95:5, flow rate = 1.0 mL/min, λ = 260 nm, retention time: 17.37 min (minor) and 20.16 min (major). ^{1}H NMR (400 MHz, Chloroform-*d*) δ 7.29 (q, *J* = 8.5 Hz, 4H), 6.46 (d, *J* = 16.4 Hz, 1H), 6.38 (d, *J* = 16.4 Hz, 1H), 4.20 (q, *J* = 7.1 Hz, 2H), 2.75-2.68 (m, 1H), 2.53-2.21 (m, 3H), 2.14-1.93 (m, 2H), 1.26 (t, *J* = 7.2 Hz, 3H).^{13}C NMR (100 MHz, Chloroform-*d*) δ 212.3, 170.3, 135.1, 133.8, 130.6, 128.9, 127.9, 126.7, 63.0, 62.1, 37.7, 33.6, 19.7, 14.2. IR (thin film, cm^{-1}): 2978, 2940, 2905, 1722, 1491, 1231, 1092, 803, 506. HRMS (ESI) calcd. for $C_{16}H_{17}ClO_3H^+$: 293.0939, found: 293.0931.

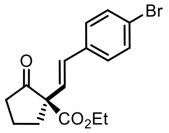

3j

3j: The compound was synthesized according to the general procedure, using ethyl 2-oxocyclopentanecarboxylate (15.6 mg, 0.1 mmol) and corresponding alkene (91 mg, 0.5 mmol). The product was purified by column chromatography (PE/EA=5:1), white solid, 14.4 mf, 43% yield, 89% *ee*, $[\alpha]_D^{20}$ = -26.3 (c = 1.45, $CHCl_3$). HPLC analysis: Daicel Chiralpak IC, hexane/iso-propanol = 98:2, flow rate = 1.0 mL/min, λ = 254 nm, retention time: 19.86 min (major) and 22.82 min (minor). ^{1}H NMR (400 MHz, Chloroform-*d*) δ 7.43 (d, *J* = 8.5 Hz, 2H), 7.25 (d, *J* = 7.0 Hz, 2H), 6.43 (s, 1H), 6.42 (s, 1H), 4.24-4.16 (m, 2H), 2.75-2.69 (m, 1H), 2.51-2.35 (m, 2H), 2.33-2.22 (m, 1H), 2.13-1.93 (m, 2H), 1.26 (t, *J* = 7.1 Hz, 3H).^{13}C NMR (100 MHz, Chloroform-*d*) δ 212.3, 170.2, 135.5, 131.8, 130.7, 128.2, 126.8, 121.9, 63.0, 62.1, 37.7, 33.6, 19.7, 14.2. IR (thin film, cm^{-1}): 2977, 2928, 2854, 1750, 1722, 1488, 1401, 1231, 971, 503. HRMS (ESI) calcd. for $C_{16}H_{17}BrO_3H^+$: 337.0434, found: 337.0430.

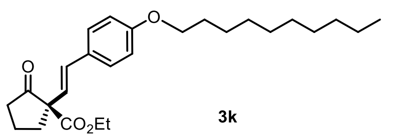

3k

3k: The compound was synthesized according to the general procedure, using ethyl 2-oxocyclopentanecarboxylate (15.6 mg, 0.1 mmol) and corresponding alkene (130 mg, 0.5 mmol). The product was purified by column chromatography (PE/EA=4:1), colorless oil, 17.4 mg, 42% yield, 90% *ee*, $[\alpha]_D^{20}$ = +3.1 (c = 1.73, $CHCl_3$). HPLC analysis: Daicel Chiralpak IC, hexane/iso-propanol = 98:2, flow rate = 1.0 mL/min, λ = 254 nm, retention time: 19.07 min (major) and 20.37 min (minor). ^{1}H NMR (400 MHz, Chloroform-*d*) δ 7.31 (d, *J* = 8.7 Hz, 2H), 6.87-6.78 (m, 2H), 6.45 (d, *J* = 16.3 Hz, 1H), 6.25 (d, *J* = 16.3 Hz, 1H), 4.22-4.17 (m, 2H), 4.04-3.90 (m, 2H), 2.76-2.58 (m, 1H), 2.52-2.17 (m, 3H), 2.11-1.82 (m, 2H), 1.80-1.73 (m, 2H), 1.48-1.41 (m, 2H), 1.40-1.20 (m, 15H), 1.14 (t, *J* = 7.2 Hz, 1H), 0.88 (t, *J* = 6.7 Hz, 3H).^{13}C NMR (100 MHz, Chloroform-*d*) δ 212.6, 170.6, 159.2, 132.9, 131.3, 129.9, 129.1, 127.9, 127.4, 123.4, 114.7, 114.1, 68.2, 63.0, 61.9, 37.6, 37.3, 35.7, 33.6, 32.0, 29.7, 29.5, 29.5, 29.4, 26.2, 22.8, 19.7, 14.3, 14.2, 14.0. IR (thin film, cm^{-1}): 2924, 2854, 1752, 1722, 1607, 1510, 1244, 1173, 1026, 826, 522. HRMS (ESI) calcd. for $C_{26}H_{38}O_4Na^+$: 437.2662, found: 437.2650.

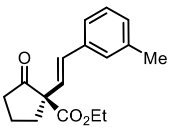

3l

3l: The compound was synthesized according to the general procedure, using ethyl 2-oxocyclopentanecarboxylate (15.6 mg, 0.1 mmol) and corresponding alkene (59 mg, 0.5 mmol). The product was purified by column chromatography (PE/EA=5:1), colorless oil, 18.2 mg, 67% yield, 91% *ee*, $[\alpha]_D^{20}$ = -31.9 (c = 1.80, $CHCl_3$). HPLC analysis: Daicel Chiralpak OJ-H, hexane/iso-propanol = 95:5, flow rate = 1.0 mL/min, λ = 254 nm, retention time: 20.50 min (minor) and 23.02 min (major). ^{1}H NMR (400 MHz, Chloroform-*d*) δ 7.24-7.17 (m, 3H), 7.06 (d, *J* = 6.0 Hz, 1H), 6.49 (d, *J* = 16.3 Hz, 1H), 6.40 (d, *J* = 16.4 Hz, 1H), 4.20 (q, *J* = 7.2 Hz, 2H), 2.76-2.69 (m, 1H), 2.49-2.39 (m, 1H), 2.38-2.18 (m, 5H), 2.11-1.96 (m, 2H), 1.27 (t, *J* = 7.1 Hz, 3H).^{13}C NMR (100 MHz, Chloroform-*d*) δ 212.5, 170.4, 138.3, 136.5, 131.9, 128.9, 128.6, 127.3, 125.7, 123.9, 63.0, 62.0, 37.6, 33.5, 21.5, 19.7, 14.2. IR (thin film, cm^{-1}): 3032, 2978, 2927, 1751, 1724, 1227, 1172, 772, 694. HRMS (ESI) calcd. for $C_{17}H_{20}O_3H^+$: 273.1485, found: 273.1479.

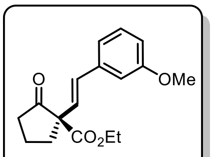

3m

3m: The compound was synthesized according to the general procedure, using ethyl 2-oxocyclopentanecarboxylate (15.6 mg, 0.1 mmol) and corresponding alkene (67 mg, 0.5 mmol). The product was purified by column chromatography (PE/EA=3:1), colorless oil, 16.4 mg, 57% yield, 88% *ee*, $[\alpha]_D^{20}$ = -41.1 (c = 1.63, $CHCl_3$). HPLC analysis: Daicel Chiralpak OD-H, hexane/iso-propanol = 98:2, flow rate = 1.0 mL/min, λ = 250 nm, retention time: 16.22 min (major) and 22.23 min (minor). ^{1}H NMR (400 MHz, Chloroform-*d*) δ 7.29-7.18 (m, 1H), 7.02-6.90 (m, 2H), 6.81 (dd, *J* = 8.2, 2.6 Hz, 1H), 6.49 (d, *J* = 16.3 Hz, 1H), 6.40 (dd, *J* = 16.3, 1.0 Hz, 1H), 4.20 (q, *J* = 7.2 Hz, 2H), 3.81 (d, *J* = 1.0 Hz, 3H), 2.76-2.69 (m, 1H), 2.52-2.23 (m, 3H), 2.12-1.94 (m, 2H), 1.26 (t, *J* = 7.1 Hz, 3H).^{13}C NMR (100 MHz, Chloroform-*d*) δ 212.4, 170.3, 159.9, 138.0, 131.7, 129.7, 126.2, 119.4, 114.0, 111.7, 63.0, 62.0, 55.4, 37.7, 33.5, 19.7, 14.2. IR (thin film, cm^{-1}): 2964, 2921, 2837, 1750, 1723, 1579, 1229, 1158, 776, 690. HRMS (ESI) calcd. for $C_{17}H_{20}O_4Na^+$: 311.1254, found: 311.1247.

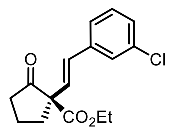

3n

3n: The compound was synthesized according to the general procedure, using ethyl 2-oxocyclopentanecarboxylate (15.6 mg, 0.1 mmol) and corresponding alkene (69 mg, 0.5 mmol). The product was purified by column chromatography (PE/EA=5:1), colorless oil, 9.0 mg, 31% yield, 90% *ee*, $[\alpha]_D^{20}$ = -54.9 (c = 0.90, $CHCl_3$). HPLC analysis: Daicel Chiralpak OD-H, hexane/iso-propanol = 98:2, flow rate = 1.0 mL/min, λ = 254 nm, retention time: 9.66 min (major) and 11.34 min (minor). ^{1}H NMR (400 MHz, Chloroform-*d*) δ 7.38 (s, 1H), 7.27-7.22 (m, 3H), 6.44 (s, 1H), 6.44 (s, 1H), 4.24-4.18 (m, 2H), 2.76-2.69 (m, 1H), 2.53-2.21 (m, 3H), 2.13-1.94 (m, 2H), 1.27 (t, *J* = 7.1 Hz, 3H). ^{13}C NMR (100 MHz, Chloroform-*d*) δ 212.2, 170.2, 138.4, 134.7, 130.6, 129.9, 128.0, 127.6, 126.6, 124.9, 63.0, 62.2, 37.7, 33.6, 19.7, 14.2. IR (thin film, cm^{-1}): 2967, 2931, 2856, 1751, 1724, 1231, 1167, 777, 684. HRMS (ESI) calcd. for $C_{16}H_{17}ClO_3Na^+$: 315.0758, found: 315.0750.

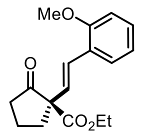

3o

3o: The compound was synthesized according to the general procedure, using ethyl 2-oxocyclopentanecarboxylate (15.6 mg, 0.1 mmol) and corresponding alkene (67 mg, 0.5 mmol). The product was purified by column chromatography (PE/EA=3:1), colorless oil, 14.4 mg, 50% yield, 93% *ee*, $[\alpha]_D^{20}$ = +17.5 (c = 1.40, $CHCl_3$). HPLC analysis: Daicel Chiralpak OD-H, hexane/iso-propanol = 95:5, flow rate = 1.0 mL/min, λ = 254 nm, retention time: 11.15 min (major) and 13.65 min (minor). ^{1}H NMR (400 MHz, Chloroform-*d*) δ 7.47 (dd, *J* = 7.7, 1.7 Hz, 1H), 7.26-7.18 (m, 1H), 6.95-6.85 (m, 2H), 6.84 (d, *J* = 1.5 Hz, 1H), 6.43 (d, *J* = 16.5 Hz, 1H), 4.20 (q, *J* = 7.1, 6.6 Hz, 2H), 3.83 (s, 3H), 2.76-2.69 (m, 1H), 2.51-2.27 (m, 3H), 2.10-1.97 (m, 2H), 1.27 (t, *J* = 7.1 Hz, 3H). ^{13}C NMR (100 MHz,

Chloroform-*d*) δ 212.6, 170.6, 156.8, 129.1, 126.9, 126.4, 126.2, 125.6, 120.8, 110.9, 63.5, 61.9, 55.6, 37.7, 33.3, 19.7, 14.2. IR (thin film, cm^{-1}): 3057, 2964, 2838, 1749, 1718, 1488, 1463, 1242, 1025, 751. HRMS (ESI) calcd. for $C_{17}H_{20}O_4Na^+$: 311.1254, found: 311.1244.

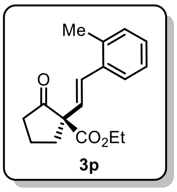

3p: The compound was synthesized according to the general procedure, using ethyl 2-oxocyclopentanecarboxylate (15.6 mg, 0.1 mmol) and corresponding alkene (59 mg, 0.5 mmol). The product was purified by column chromatography (PE/EA=5:1), colorless oil, 16.6 mg, 61% yield, 90% *ee*, $[\alpha]_D^{20}$ = -54.7 (c = 1.70, $CHCl_3$). HPLC analysis: Daicel Chiralpak AD-H, hexane/iso-propanol = 98:2, flow rate = 1.0 mL/min, λ = 254 nm, retention time: 8.89 min (major) and 9.48 min (minor). ^{1}H NMR (400 MHz, Chloroform-*d*) δ 7.49-7.39 (m, 1H), 7.18-7.12 (m, 3H), 6.74 (d, *J* = 16.2 Hz, 1H), 6.27 (d, *J* = 16.2 Hz, 1H), 4.21 (q, *J* = 7.1 Hz, 2H), 2.77-2.70 (m, 1H), 2.52-2.21 (m, 6H), 2.16-1.93 (m, 2H), 1.28 (t, *J* = 7.1 Hz, 3H).^{13}C NMR (100 MHz, Chloroform-*d*) δ 212.4, 170.5, 135.8, 135.7, 130.3, 129.9, 127.9, 127.4, 126.3, 126.0, 63.3, 62.0, 37.6, 33.7, 19.9, 19.7, 19.7, 14.2. IR (thin film, cm^{-1}): 3049, 3017, 2977, 1750, 1722, 1231, 1170, 1027, 970, 750. HRMS (ESI) calcd. for $C_{17}H_{20}O_3H^+$: 273.1485, found: 273.1477.

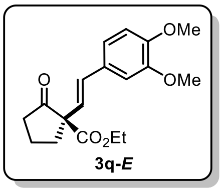

3q-*E*: The compound was synthesized according to the general procedure, using ethyl 2-oxocyclopentanecarboxylate (15.6 mg, 0.1 mmol) and corresponding alkene (82 mg, 0.5 mmol). The product was purified by column chromatography (PE/EA=4:1), colorless oil, 10.1 mg, 32% yield, 93% *ee*, $[\alpha]_D^{20}$ = +107.9 (c = 0.59, $CHCl_3$). HPLC analysis: Daicel Chiralpak OJ-H, hexane/iso-propanol = 80:20, flow rate = 1.0 mL/min, λ = 270 nm, retention time: 22.42 min (major) and 26.62 min (minor). ^{1}H NMR (400 MHz, Chloroform-*d*) δ 7.26 (s, 1H), 6.97-6.89 (m, 2H), 6.81 (d, *J* = 8.2 Hz, 1H), 6.45 (d, *J* = 16.3 Hz, 1H), 6.27 (d, *J* = 16.3 Hz, 1H), 4.23-4.16 (m, 2H), 3.89 (d, *J* = 9.5 Hz, 6H), 2.75-2.69 (m, 1H), 2.46-2.23 (m, 4H), 1.26 (t, *J* = 7.1 Hz, 3H).^{13}C NMR (100 MHz, Chloroform-*d*) δ 212.6, 170.4, 149.1, 149.0, 131.5, 129.5, 123.6, 120.0, 111.0, 108.7, 62.8, 61.9, 55.9, 55.9, 37.5, 33.4, 19.6, 14.1. IR (thin film, cm^{-1}): 2960, 2936, 2836, 1748, 1721, 1513, 1262, 1232, 1139, 1025. HRMS (ESI) calcd. for $C_{18}H_{22}O_5Na^+$: 341.1359, found: 341.1359.

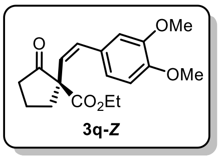

3q-*Z*: The compound was synthesized according to the general procedure, using ethyl 2-oxocyclopentanecarboxylate (15.6 mg, 0.1 mmol) and corresponding alkene (82 mg, 0.5 mmol). The product was purified by column chromatography (PE/EA=4:1), colorless oil, 5.1 mg, 16% yield, 93% *ee*, $[\alpha]_D^{20}$ = +107.9 (c = 0.59, $CHCl_3$). HPLC analysis: Daicel Chiralpak OJ-H, hexane/iso-propanol =80:20, flow rate = 1.0 mL/min, λ = 254 nm, retention time: 13.40 min (major) and 25.39 min (minor). ^{1}H NMR (400 MHz, Chloroform-*d*) δ 6.96 (s, 1H), 6.80 (d, *J* = 1.1 Hz, 2H), 6.67 (d, *J* = 12.0 Hz, 1H), 5.68 (d, *J*

= 12.0 Hz, 1H), 3.93-3.83 (m, 8H), 2.66-2.61 (m, 1H), 2.51-2.43 (m, 1H), 2.30-2.18 (m, 1H), 2.03-1.83 (m, 3H), 1.09 (t, *J* = 7.1 Hz, 3H). ^{13}C NMR (100 MHz, Chloroform-*d*) δ 213.3, 170.3, 148.5, 148.4, 133.3, 129.7, 127.4, 121.3, 111.7, 110.7, 62.5, 61.9, 56.0, 55.9, 37.5, 36.3, 19.6, 14.0. IR (thin film, cm^{-1}): 2960, 2936, 2836, 1750, 1711, 1512, 1236, 1138, 1024. HRMS (ESI) calcd. for $C_{18}H_{22}O_5Na^+$: 341.1359, found: 341.1359.

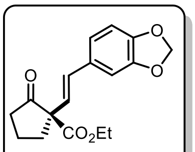

3r-*E*

3r-*E*: The compound was synthesized according to the general procedure, using ethyl 2-oxocyclopentanecarboxylate (31.2 mg, 0.2 mmol) and corresponding alkene (148 mg, 1.0 mmol). The product was purified by column chromatography (PE/EA=5:1), colorless oil, 15.4 mg, 24% yield, 91% *ee*, $[\alpha]_D^{20}$ = +116.0 (c = 1.60, $CHCl_3$). HPLC analysis: Daicel Chiralpak AD-H, hexane/iso-propanol = 98:2, flow rate = 1.0 mL/min, λ = 254 nm, retention time: 14.81 min (major) and 15.60 min (minor). ^{1}H NMR (400 MHz, Chloroform-*d*) δ 6.95 (s, 1H), 6.81 (d, *J* = 8.0 Hz, 1H), 6.74 (d, *J* = 8.0 Hz, 1H), 6.42 (d, *J* = 16.3 Hz, 1H), 6.22 (d, *J* = 16.3 Hz, 1H), 5.95 (s, 2H), 4.19 (q, *J* = 7.0 Hz, 2H), 2.74-2.67 (m, 1H), 2.45-2.22 (m, 3H), 2.09-1.95 (m, 2H), 1.26 (t, *J* = 7.1 Hz, 3H). ^{13}C NMR (100 MHz, Chloroform-*d*) δ 212.5, 170.5, 148.2, 147.6, 131.4, 131.0, 124.1, 121.6, 108.4, 105.9, 101.3, 62.9, 62.0, 37.7, 33.6, 19.7, 14.2. IR (thin film, cm^{-1}): 2977, 2891, 2778, 1751, 1714, 1487, 1439, 1219, 1035, 730. HRMS (ESI) calcd. for $C_{17}H_{18}O_5Na^+$: 325.1046, found: 325.1038.

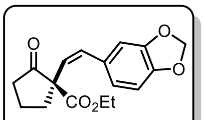

3r-*Z*

3r-*Z*: The compound was synthesized according to the general procedure, using ethyl 2-oxocyclopentanecarboxylate (31.2 mg, 0.2 mmol) and corresponding alkene (148 mg, 1.0 mmol). The product was purified by column chromatography (PE/EA=5:1), colorless oil, 4.8 mg, 8% yield, 91% *ee*, $[\alpha]_D^{20}$ = +25.4 (c = 2.80, $CHCl_3$). HPLC analysis: Daicel Chiralpak AD-H, hexane/iso-propanol = 98:2, flow rate = 1.0 mL/min, λ = 254 nm, retention time: 30.89 min (major) and 36.53 min (minor). ^{1}H NMR (400 MHz, Chloroform-*d*) δ 6.76 (s, 1H), 6.74 (s, 2H), 6.61 (d, *J* = 12.0 Hz, 1H), 5.94 (s, 2H), 5.73 (d, *J* = 12.0 Hz, 1H), 4.01 (q, *J* = 7.1 Hz, 2H), 2.65-2.53 (m, 1H), 2.51-2.39 (m, 1H), 2.29-2.13 (m, 1H), 2.01-1.81 (m, 3H), 1.17 (t, *J* = 7.1 Hz, 3H).^{13}C NMR (100 MHz, Chloroform-*d*) δ 213.1, 170.0, 147.4, 147.0, 132.8, 130.9, 128.1, 122.4, 109.0, 108.0, 101.1, 62.6, 61.9, 37.3, 35.8, 19.6, 14.0. IR (thin film, cm^{-1}): 2976, 2897, 2779, 1750, 1724, 1504, 1489, 1445, 1249, 1038. HRMS (ESI) calcd. for $C_{17}H_{18}O_5Na^+$: 325.1046, found: 325.1041.

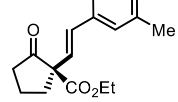

3s

3s: The compound was synthesized according to the general procedure, using ethyl 2-oxocyclopentanecarboxylate (15.6 mg, 0.1 mmol) and corresponding alkene (66 mg, 0.5 mmol). The product was purified by column chromatography (PE/EA=5:1), colorless oil, 14.6 mg, 51% yield, 80%

ee, $[\alpha]_D^{20}$ = -41.1 (c = 1.45, $CHCl_3$). HPLC analysis: Daicel Chiralpak AD-H, hexane/iso-propanol = 98:2, flow rate = 1.0 mL/min, λ = 254 nm, retention time: 7.15 min (major) and 7.65 min (minor). ^{1}H NMR (400 MHz, Chloroform-*d*) δ 7.26 (d, *J* = 2.6 Hz, 1H), 7.06-6.91 (m, 2H), 6.71 (d, *J* = 16.2 Hz, 1H), 6.26 (d, *J* = 16.2 Hz, 1H), 4.29-4.16 (m, 2H), 2.77-2.70 (m, 1H), 2.54-2.37 (m, 2H), 2.29 (d, *J* = 9.0 Hz, 7H), 2.13-1.97 (m, 2H), 1.27 (t, *J* = 7.1 Hz, 3H).^{13}C NMR (100 MHz, Chloroform-*d*) δ 212.5, 170.5, 135.7, 135.5, 132.6, 130.3, 129.9, 128.7, 127.0, 126.6, 63.3, 62.0, 37.7, 33.6, 21.1, 19.7, 19.4, 14.2. IR (thin film, cm^{-1}): 2975, 2921, 1751, 1723, 1228, 1163, 1028, 971, 809, 750. HRMS (ESI) calcd. for $C_{18}H_{22}O_3Na^+$: 309.1461, found: 309.1454.

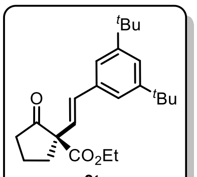

3t

3t: The compound was synthesized according to the general procedure, using ethyl 2-oxocyclopentanecarboxylate (15.6 mg, 0.1 mmol) and corresponding alkene (108 mg, 0.5 mmol). The product was purified by column chromatography (PE/EA=5:1), colorless oil, 24.2 mg, 65% yield, 93% *ee*, $[\alpha]_D^{20}$ = -14.6 (c = 4.02, $CHCl_3$). HPLC analysis: Daicel Chiralpak AD-H; IC, hexane/iso-propanol = 98:2, flow rate = 1.0 mL/min, λ = 254 nm, retention time: 11.66 min (minor) and 13.20 min (major). ^{1}H NMR (400 MHz, Chloroform-*d*) δ 7.34 (s, 1H), 7.24 (d, *J* = 1.8 Hz, 2H), 6.55 (d, *J* = 16.3 Hz, 1H), 6.39 (d, *J* = 16.3 Hz, 1H), 4.21 (q, *J* = 7.1 Hz, 2H), 2.77-2.71 (m, 1H), 2.54-2.27 (m, 3H), 2.14-1.93 (m, 2H), 1.33 (s, 18H), 1.27 (t, *J* = 7.1 Hz, 3H). ^{13}C NMR (100 MHz, Chloroform-*d*) δ 212.6, 170.5, 151.1, 135.7, 133.0, 124.9, 122.5, 121.0, 63.1, 62.0, 37.7, 35.0, 33.6, 31.6, 19.7, 14.2. IR (thin film, cm^{-1}): 3032, 2978, 2954, 2826, 1750, 1721, 1484, 1223, 1144, 1012. HRMS (ESI) calcd. for $C_{24}H_{34}O_3H^+$: 371.2581, found: 371.2526.

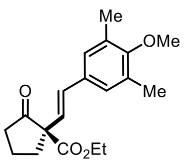

3u

3u: The compound was synthesized according to the general procedure, using ethyl 2-oxocyclopentanecarboxylate (15.6 mg, 0.1 mmol) and corresponding alkene (81 mg, 0.5 mmol). The product was purified by column chromatography (PE/EA=3:1), colorless oil, 24.0 mg, 76% yield, 94% *ee*, $[\alpha]_D^{20}$ = -26.1 (c = 2.41, $CHCl_3$). HPLC analysis: Daicel Chiralpak IC, hexane/iso-propanol = 98:2, flow rate = 1.0 mL/min, λ = 265 nm, retention time: 32.74 min (major) and 35.70 min (minor). ^{1}H NMR (400 MHz, Chloroform-*d*) δ 7.06 (s, 2H), 6.41 (d, *J* = 16.3 Hz, 1H), 6.28 (d, *J* = 16.3 Hz, 1H), 4.23-4.15 (m, 2H), 3.70 (s, 3H), 2.74-2.67 (m, 1H), 2.50-2.19 (m, 9H), 2.12-1.87 (m, 2H), 1.26 (t, *J* = 7.1 Hz, 3H).^{13}C NMR (100 MHz, Chloroform-*d*) δ 212.5, 170.5, 157.1, 132.1, 131.3, 131.1, 127.2, 124.7, 63.0, 62.0, 59.9, 37.6, 33.5, 19.7, 16.2, 14.2. IR (thin film, cm^{-1}): 2961, 2904, 2868, 1752, 1724, 1594, 1464, 1393, 1229, 707. HRMS (ESI) calcd. for $C_{19}H_{24}O_4Na^+$: 339.1567, found: 339.1565.

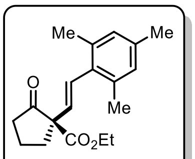

3v

3v: The compound was synthesized according to the general procedure, using ethyl 2-oxocyclopentanecarboxylate (15.6 mg, 0.1 mmol) and corresponding alkene (73 mg, 0.5 mmol). The product was purified by column chromatography (PE/EA=5:1), colorless oil, 12.0 mg, 40% yield, 86% *ee*, $[\alpha]_D^{20}$ = -38.2 (c = 1.20, $CHCl_3$). HPLC analysis: Daicel Chiralpak IC, hexane/iso-propanol = 98:2, flow rate = 1.0 mL/min, λ = 254 nm, retention time: 16.46 min (minor) and 17.54 min (major). ^{1}H NMR (400 MHz, Chloroform-*d*) δ 6.86 (s, 2H), 6.47 (d, *J* = 16.7 Hz, 1H), 5.82 (d, *J* = 16.7 Hz, 1H), 4.21 (q, *J* = 7.1 Hz, 2H), 2.74-2.67 (m, 1H), 2.47-2.37 (m, 2H), 2.35-2.30 (m, 1H), 2.26 (s, 3H), 2.24 (s, 6H), 2.12-2.00 (m, 2H), 1.28 (t, *J* = 7.2 Hz, 3H). ^{13}C NMR (100 MHz, Chloroform-*d*) δ 212.5, 170.7, 136.6, 136.0, 133.5, 130.6, 130.2, 128.6, 63.4, 61.9, 37.6, 33.7, 21.1, 20.8, 19.8, 14.2. IR (thin film, cm^{-1}): 2972, 2918, 1750, 1720, 1448, 1231, 1027, 851. HRMS (ESI) calcd. for $C_{19}H_{24}O_3Na^+$: 323.1618, found: 323.1607.

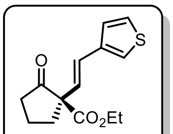

3w

3w: The compound was synthesized according to the general procedure, using ethyl 2-oxocyclopentanecarboxylate (15.6 mg, 0.1 mmol) and corresponding alkene (55 mg, 0.5 mmol). The product was purified by column chromatography (PE/EA=5:1), colorless oil, 7.9 mg, 30% yield, 96% *ee*, $[\alpha]_D^{20}$ = +13.9 (c = 0.30, $CHCl_3$). HPLC analysis: Daicel Chiralpak IC, hexane/iso-propanol = 98:2, flow rate = 1.0 mL/min, λ = 254 nm, retention time: 30.42 min (major) and 32.14 min (minor). ^{1}H NMR (400 MHz, Chloroform-*d*) δ 7.28-7.26 (m, 1H), 7.23 (d, *J* = 5.1 Hz, 1H), 7.18 (s, 1H), 6.53 (d, *J* = 16.3 Hz, 1H), 6.25 (d, *J* = 16.3 Hz, 1H), 4.20 (q, *J* = 7.2 Hz, 2H), 2.73-2.67 (m, 1H), 2.49-2.36 (m, 2H), 2.33-2.23 (m, 1H), 2.09-1.95 (m, 2H), 1.26 (t, *J* = 7.1 Hz, 3H).^{13}C NMR (100 MHz, Chloroform-*d*) δ 212.4, 170.4, 139.2, 126.3, 126.1, 125.7, 125.0, 122.9, 62.9, 62.0, 37.6, 33.6, 19.7, 14.2. IR (thin film, cm^{-1}): 3103, 2974, 2933, 1749, 1717, 1227, 1113, 1026, 967, 861, 772. HRMS (ESI) calcd. for $C_{14}H_{16}O_3SH^+$: 265.0893, found: 265.0886.

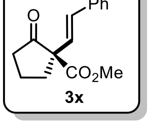

3x

3x: The compound was synthesized according to the general procedure, using corresponding *β*-ketoester (14.2 mg, 0.1 mmol) and styrene (52 mg, 0.5 mmol). The product was purified by column chromatography (PE/EA=5:1), colorless oil, 15.6 mg, 64% yield, 90% *ee*, $[\alpha]_D^{20}$ = -50.3 (c = 1.50, $CHCl_3$). HPLC analysis: Daicel Chiralpak OJ-H, hexane/iso-propanol = 95:5, flow rate = 1.0 mL/min, λ = 254 nm, retention time: 30.28 min (minor) and 34.55 min (major). ^{1}H NMR (400 MHz, Chloroform-*d*) δ 7.44-7.37 (m, 2H), 7.32 (t, *J* = 7.4 Hz, 2H), 7.27-7.22 (m, 1H), 6.51 (d, *J* = 16.3 Hz, 1H), 6.40 (d, *J* = 16.3 Hz, 1H), 3.75 (s, 3H), 2.74 (dt, *J* = 13.7, 7.1 Hz, 1H), 2.51-2.35 (m, 2H), 2.31 (dt, *J* = 13.1, 6.7 Hz, 1H), 2.11-1.94 (m, 2H). ^{13}C NMR (100 MHz, Chloroform-*d*) δ 212.1, 170.7, 136.3, 131.8, 128.6, 128.0, 126.6, 125.7, 62.9, 52.9, 37.5, 33.4, 19.6. IR (thin film, cm^{-1}): 3468, 2954, 1728, 1235, 1118, 968, 749,

697, 503. HRMS (ESI) calcd. for $C_{15}H_{16}O_3Na^+$: 267.0992, found: 267.0984.

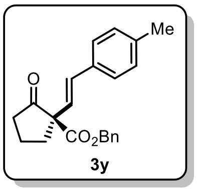

3y: The compound was synthesized according to the general procedure, using corresponding *β*-ketoester (21.8 mg, 0.1 mmol) and alkene (59 mg, 0.5 mmol). The product was purified by column chromatography (PE/EA=5:1), colorless oil, 16.4mg, 49% yield, 90% *ee*, $[\alpha]_D^{20}$ = -61.3 (c = 1.55, $CHCl_3$). HPLC analysis: Daicel Chiralpak IC, hexane/iso-propanol = 98:2, flow rate = 1.0 mL/min, λ = 254 nm, retention time: 34.78 min (major) and 46.08 min (minor). ^1H NMR (400 MHz, Chloroform-*d*) δ 7.37-7.30 (m, 5H), 7.28 (d, *J* = 7.9 Hz, 2H), 7.12 (d, *J* = 7.9 Hz, 2H), 6.50 (d, *J* = 16.3 Hz, 1H), 6.36 (d, *J* = 16.4 Hz, 1H), 5.19 (q, *J* = 14.8 Hz, 2H), 2.72 (dt, *J* = 13.7, 7.1 Hz, 1H), 2.48-2.35 (m, 2H), 2.33 (s, 3H), 2.32-2.26 (m, 1H), 2.07-1.96 (m, 2H). ^{13}C NMR (100 MHz, Chloroform-*d*) δ 212.2, 170.3, 138.0, 135.7, 133.7, 132.0, 129.4, 128.7, 128.3, 127.9, 126.6, 124.6, 67.4, 63.0, 37.6, 33.6, 21.3, 19.7. IR (thin film, cm^{-1}): 3032, 2959, 2921, 2887, 1724, 1212, 967, 798, 697, 507. HRMS (ESI) calcd. for $C_{22}H_{22}O_3Na^+$: 357.1461, found: 357.1450.

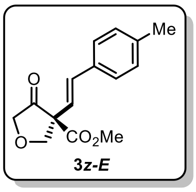

3z-*E*: The compound was synthesized according to the general procedure, using corresponding *β*-ketoester (144 mg, 0.1 mmol) and alkene (59 mg, 0.5 mmol). The product was purified by column chromatography (PE/EA=3:1), colorless oil, 3.4 mg, 13% yield, 90% *ee*, $[\alpha]_D^{20}$ = -27.0 (c = 0.35, $CHCl_3$). HPLC analysis: Daicel Chiralpak AD-H, hexane/iso-propanol = 98:2, flow rate = 1.0 mL/min, λ = 254 nm, retention time: 18.05 min (minor) and 21.43 min (major). ^1H NMR (400 MHz, Chloroform-*d*) δ 7.29 (d, *J* = 7.9 Hz, 2H), 7.13 (d, *J* = 7.8 Hz, 2H), 6.63 (d, *J* = 16.4 Hz, 1H), 6.34 (d, *J* = 16.4 Hz, 1H), 4.82 (d, *J* = 9.7 Hz, 1H), 4.37 (d, *J* = 9.8 Hz, 1H), 4.19-4.05 (m, 2H), 3.80 (s, 3H), 2.34 (s, 3H).^{13}C NMR (100 MHz, Chloroform-*d*) δ 207.5, 168.7, 138.5, 133.6, 133.2, 129.5, 126.7, 121.1, 75.1, 70.7, 61.8, 53.5, 21.4. IR (thin film, cm^{-1}): 2954, 2922, 2853, 1773, 1734, 1434, 1237, 808, 507. HRMS (ESI) calcd. for $C_{16}H_{18}O_4H^+$: 275.1278, found: 275.1252.

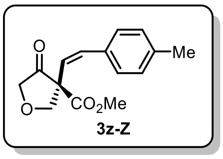

3z-*Z*: The compound was synthesized according to the general procedure, using corresponding *β*-ketoester (14.4 mg, 0.1 mmol) and alkene (59 mg, 0.5 mmol). The product was purified by column chromatography (PE/EA=3:1), colorless oil, 3.4 mg, 13% yield, 90% *ee*, $[\alpha]_D^{20}$ = -27.0 (c = 0.35, $CHCl_3$). HPLC analysis: Daicel Chiralpak AD-H, hexane/iso-propanol = 98:2, flow rate = 1.0 mL/min, λ = 254 nm, retention time: 10.46 min (minor) and 14.47 min (major). ^1H NMR (400 MHz, Chloroform-*d*) δ 7.16-7.07 (m, 4H), 6.79 (d, *J* = 11.8 Hz, 1H), 5.81 (d, *J* = 11.8 Hz, 1H), 4.62 (d, *J* = 10.0 Hz, 1H), 4.18 (d, *J* = 17.2 Hz, 1H), 3.85-3.73 (m, 2H), 3.63 (s, 3H), 2.34 (s, 3H). ^{13}C NMR (100 MHz, Chloroform-*d*)

δ 208.41, 168.45, 137.91, 135.07, 133.44, 129.11, 128.23, 124.11, 75.73, 70.52, 61.68, 53.42, 21.39. HRMS (ESI) calcd. for $C_{16}H_{18}O_4H^+$: 275.1278, found: 275.1240.

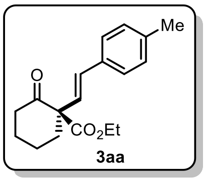

3aa: The compound was synthesized according to the general procedure, using corresponding *β*-ketoester (17.0 mg, 0.1 mmol) and alkene (59 mg, 0.5 mmol). The product was purified by column chromatography (PE/EA=5:1), colorless oil, 8.6 mg, 30% yield, 70% *ee*, $[\alpha]_D^{20}$ = +22.4 (c = 0.86, $CHCl_3$). HPLC analysis: Daicel Chiralpak IC, hexane/iso-propanol = 98:2, flow rate = 1.0 mL/min, λ = 254 nm, retention time: 20.72 min (minor) and 26.25 min (major). ^{1}H NMR (400 MHz, Chloroform-*d*) δ 7.30 (d, *J* = 7.7 Hz, 2H), 7.11 (d, *J* = 7.8 Hz, 2H), 6.64 (d, *J* = 16.5 Hz, 1H), 6.41 (d, *J* = 16.5 Hz, 1H), 4.29-4.16 (m, 2H), 2.76-2.72 (m, 1H), 2.60-2.40 (m, 2H), 2.33 (s, 3H), 2.04-2.00 (m, 1H), 1.89-1.67 (m, 4H), 1.26 (t, *J* = 7.1 Hz, 3H).^{13}C NMR (100 MHz, Chloroform-*d*) δ 206.3, 170.5, 137.8, 134.0, 131.3, 129.4, 126.6, 126.1, 62.6, 61.9, 41.0, 36.5, 27.4, 22.7, 21.4, 14.2. IR (thin film, cm^{-1}): 2939, 2866, 1713, 1513, 1449, 1233, 1199, 969, 506. HRMS (ESI) calcd. for $C_{18}H_{22}O_3H^+$: 287.1642, found: 287.1634.

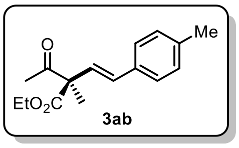

3ab: The compound was synthesized according to the general procedure, using corresponding *β*-ketoester (14.4 mg, 0.1 mmol) and alkene (59 mg, 0.5 mmol). The product was purified by column chromatography (PE/EA=3:1), colorless oil, 5.2 mg, 20% yield, 74% *ee*, $[\alpha]_D^{20}$ = -81.8 (c = 0.35, $CHCl_3$). HPLC analysis: Daicel Chiralpak IC, hexane/iso-propanol = 98:2, flow rate = 1.0 mL/min, λ = 254 nm, retention time: 12.58 min (major) and 13.74 min (minor). ^{1}H NMR (400 MHz, Chloroform-*d*) δ 7.30 (d, *J* = 7.8 Hz, 2H), 7.14 (d, *J* = 7.8 Hz, 2H), 6.68 (d, *J* = 16.5 Hz, 1H), 6.42 (d, *J* = 16.4 Hz, 1H), 4.25 (q, *J* = 7.1 Hz, 2H), 2.34 (s, 3H), 2.20 (s, 3H), 1.58 (s, 3H), 1.29 (t, *J* = 7.1 Hz, 3H).^{13}C NMR (100 MHz, Chloroform-*d*) δ 203.5, 172.1, 138.1, 133.8, 131.7, 129.5, 126.6, 126.4, 62.0, 61.9, 26.8, 21.4, 20.0, 14.2. IR (thin film, cm^{-1}): 2984, 2937, 2873, 1714, 1355, 1238, 1106, 973, 798. HRMS (ESI) calcd. for $C_{16}H_{20}O_3H^+$: 261.1485, found: 261.1480.

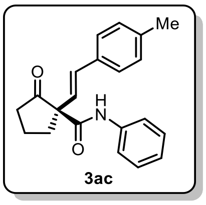

3ac: The compound was synthesized according to the general procedure, using corresponding *β*-ketoester (20.3 mg, 0.1 mmol) and alkene (59 mg, 0.5 mmol). The product was purified by column chromatography (PE/EA=3:1), colorless oil, 23.3 mg, 73% yield, 83% *ee*, $[\alpha]_D^{20}$ = -51.3 (c = 2.36, $CHCl_3$). HPLC analysis: Daicel Chiralpak IC, hexane/iso-propanol = 98:2, flow rate = 1.0 mL/min, λ = 254 nm, retention time: 32.60 min (minor) and 36.83 min (major). ^{1}H NMR (400 MHz, Chloroform-*d*) δ 8.65 (s, 1H), 7.54 (dd, *J* = 19.3, 7.7 Hz, 2H), 7.38-7.23 (m, 4H), 7.11 (dd, *J* = 16.8, 7.8 Hz, 3H), 6.56 (d, *J* = 16.2 Hz, 1H), 6.23 (d, *J* = 16.1 Hz, 1H), 2.88-2.74 (m, 1H), 2.59-2.41 (m, 2H), 2.41-2.35 (m, 1H), 2.33 (s, 3H), 2.07-1.88 (m, 2H). ^{13}C NMR (100 MHz, Chloroform-*d*) δ 217.9, 166.5, 138.5, 137.8, 133.1, 132.7, 129.5, 129.1,

126.6, 126.1, 124.5, 119.9, 63.8, 38.9, 33.3, 21.4, 18.9. IR (thin film, cm^{-1}): 3331, 3027, 2923, 1724, 1684, 1599, 1536, 1441, 754, 508. HRMS (ESI) calcd. for $C_{16}H_{20}O_3H^+$: 320.1645, found: 320.1637.

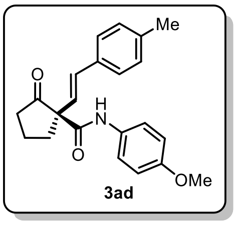

3ad: The compound was synthesized according to the general procedure, using corresponding *β*-ketoester (230 mg, 0.1 mmol) and alkene (59 mg, 0.5 mmol). The product was purified by column chromatography (PE/EA=3:1), colorless oil, 19.9 mg, 57% yield, 83% *ee*, $[\alpha]_D^{20}$ = -83.0 (c = 0.90, $CHCl_3$). HPLC analysis: Daicel Chiralpak IC, hexane/iso-propanol = 95:5, flow rate = 1.0 mL/min, λ = 262 nm, retention time: 39.58 min (minor) and 46.20 min (major). ^{1}H NMR (400 MHz, Chloroform-*d*) δ 8.52 (s, 1H), 7.44 (d, *J* = 9.0 Hz, 2H), 7.27 (d, *J* = 7.5 Hz, 2H), 7.13 (d, *J* = 7.9 Hz, 2H), 6.84 (d, *J* = 9.1 Hz, 2H), 6.55 (d, *J* = 16.2 Hz, 1H), 6.23 (d, *J* = 16.2 Hz, 1H), 3.78 (s, 3H), 2.84-2.76 (m, 1H), 2.57-2.36 (m, 3H), 2.33 (s, 3H), 2.05-1.88 (m, 2H).^{13}C NMR (100 MHz, Chloroform-*d*) δ 217.9, 166.3, 156.5, 138.5, 133.2, 132.6, 131.0, 129.5, 126.6, 126.2, 121.6, 114.2, 63.7, 55.6, 38.9, 33.3, 21.4, 18.9. IR (thin film, cm^{-1}): 3337, 2997, 2954, 2931, 1724, 1510, 1235, 1034, 828, 508. HRMS (ESI) calcd. for $C_{22}H_{23}NO_3Na^+$: 372.1570, found: 372.1560.

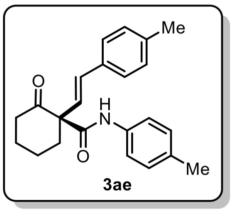

3ae: The compound was synthesized according to the general procedure, using corresponding *β*-ketoester (22.8 mg, 0.1 mmol) and alkene (59 mg, 0.5 mmol). The product was purified by column chromatography (PE/EA=3:1), colorless oil, 10.8 mg, 31% yield, 67% *ee*, $[\alpha]_D^{20}$ = -29.9 (c = 0.90, $CHCl_3$). HPLC analysis: Daicel Chiralpak AD-H, hexane/iso-propanol = 95:5, flow rate = 1.0 mL/min, λ = 254 nm, retention time: 48.26 min (major) and 61.68 min (minor). ^{1}H NMR (400 MHz, Chloroform-*d*) δ 8.25 (s, 1H), 7.36 (d, *J* = 8.4 Hz, 2H), 7.30-7.22 (m, 2H), 7.10 (dd, *J* = 12.9, 8.0 Hz, 4H), 6.52 (d, *J* = 16.6 Hz, 1H), 6.42 (d, *J* = 16.6 Hz, 1H), 2.66-2.47 (m, 3H), 2.30 (d, *J* = 16.3 Hz, 6H), 2.24-2.13 (m, 1H), 1.98-1.76 (m, 4H).^{13}C NMR (100 MHz, Chloroform-*d*) δ 211.1, 167.6, 138.4, 135.2, 134.2, 133.4, 132.8, 129.6, 129.5, 128.2, 126.6, 120.2, 63.3, 40.7, 35.6, 26.2, 21.7, 21.4, 21.0. IR (thin film, cm^{-1}): 3337, 2925, 2361, 1597, 1515, 1313, 811, 751, 511. HRMS (ESI) calcd. for $C_{23}H_{25}NO_2Na^+$: 370.1778, found: 370.1766.

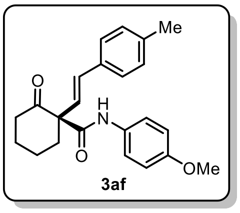

3af: The compound was synthesized according to the general procedure, using corresponding *β*-ketoester (24.4 mg, 0.1 mmol) and alkene (59 mg, 0.5 mmol). The product was purified by column chromatography (PE/EA=3:1), colorless oil, 9.4 mg, 26% yield, 70% *ee*, $[\alpha]_D^{20}$ = -29.9 (c = 0.90, $CHCl_3$). HPLC analysis:

Daicel Chiralpak OD-H, hexane/iso-propanol = 90:10, flow rate = 1.0 mL/min, λ = 254 nm, retention time: 12.57 min (major) and 15.65 min (minor). ^{1}H NMR (400 MHz, Chloroform-*d*) δ 8.22 (s, 1H), 7.38 (d, *J* = 9.0 Hz, 2H), 7.29-7.22 (m, 2H), 7.11 (d, *J* = 7.9 Hz, 2H), 6.81 (d, *J* = 9.0 Hz, 2H), 6.51 (d, *J* = 16.6 Hz, 1H), 6.42 (d, *J* = 16.6 Hz, 1H), 3.75 (s, 3H), 2.66-2.46 (m, 3H), 2.32 (s, 3H), 2.23-2.14 (m, 1H), 1.95-1.77 (m, 4H). ^{13}C NMR (100 MHz, Chloroform-*d*) δ 211.2, 167.6, 156.6, 138.4, 133.4, 132.9, 130.9, 129.5, 128.3, 126.6, 121.9, 114.2, 63.2, 55.6, 40.7, 35.6, 26.2, 21.7, 21.4. IR (thin film, cm^{-1}): 3338, 2931, 2863, 2836, 1688, 1510, 1235, 1035, 827, 736. HRMS (ESI) calcd. for $C_{23}H_{25}NO_3Na^+$: 386.1727, found: 386.1715.

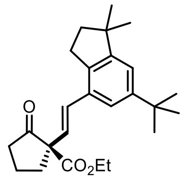

3ag

3ag: The compound was synthesized according to the general procedure, using corresponding *β*-ketoester (15.6 mg, 0.1 mmol) and alkene (68 mg, 0.3 mmol). The product was purified by column chromatography (PE/EA=5:1), colorless oil, 17.2 mg, 45% yield, 94% *ee*, $[\alpha]_D^{20}$ = -40.0 (c = 1.70, $CHCl_3$). HPLC analysis: Daicel Chiralpak OD-H*2, hexane/iso-propanol = 98:2, flow rate = 0.5 mL/min, λ = 254 nm, retention time: 18.81 min (major) and 20.75 min (minor). ^{1}H NMR (400 MHz, Chloroform-*d*) δ 7.29 (s, 1H), 7.09 (s, 1H), 6.60 (d, *J* = 16.4 Hz, 1H), 6.33 (d, *J* = 16.4 Hz, 1H), 4.21 (q, *J* = 7.1 Hz, 2H), 2.90 (t, *J* = 7.2 Hz, 2H), 2.77-2.70 (m, 1H), 2.53-2.27 (m, 3H), 2.10-1.97 (m, 2H), 1.93 (t, *J* = 7.2 Hz, 2H), 1.33 (s, 9H), 1.31-1.21 (m, 9H). ^{13}C NMR (100 MHz, Chloroform-*d*) δ 212.6, 170.5, 153.2, 150.2, 138.2, 131.9, 130.7, 126.5, 121.0, 118.7, 63.3, 62.0, 44.1, 41.4, 37.6, 34.9, 33.5, 31.7, 31.6, 28.8, 19.7, 14.3. IR (thin film, cm^{-1}): 2953, 2863, 1752, 1725, 1461, 1362, 1228, 970, 877. HRMS (ESI) calcd. for $C_{25}H_{34}O_3Na^+$: 405.2400, found: 405.2393.

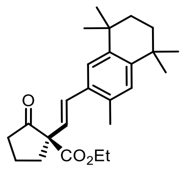

3ah

3ah: The compound was synthesized according to the general procedure, using corresponding *β*-ketoester (15.6 mg, 0.1 mmol) and alkene (68 mg, 0.3 mmol). The product was purified by column chromatography (PE/EA=5:1), colorless oil, 19.8 mg, 52% yield, 88% *ee*, $[\alpha]_D^{20}$ = -12.6 (c = 1.65, $CHCl_3$). HPLC analysis: Daicel Chiralpak IE, hexane/iso-propanol = 98:2, flow rate = 1.0 mL/min, λ = 254 nm, retention time: 5.86 min (major) and 6.19 min (minor). ^{1}H NMR (400 MHz, Chloroform-*d*) δ 7.34 (s, 1H), 7.12 (s, 1H), 6.69 (d, *J* = 16.2 Hz, 1H), 6.21 (d, *J* = 16.2 Hz, 1H), 4.20 (q, *J* = 7.1 Hz, 2H), 2.77-2.70 (q, 1H), 2.47-2.36 (m, 2H), 2.29 (s, 3H), 2.11-1.96 (m, 2H), 1.89-1.82 (q, 1H), 1.62 (t, *J* = 13.2 Hz, 1H), 1.36 (dd, *J* = 13.4, 2.6 Hz, 1H), 1.31 (d, *J* = 2.7 Hz, 5H), 1.29 (s, 1H), 1.28-1.25 (m, 5H), 1.05 (s, 3H), 0.98 (d, *J* = 6.8 Hz, 3H). ^{13}C NMR (100 MHz, Chloroform-*d*) δ 212.5, 170.6, 146.2, 142.8, 133.1, 132.8, 130.1, 128.9, 126.4, 124.0, 63.3, 61.9, 43.9, 37.7, 37.6, 34.7, 34.2, 33.7, 32.6, 32.2, 28.7, 25.0, 19.8, 19.7, 17.0, 14.2. IR (thin film, cm^{-1}): 2961, 2924, 2854, 1753, 1725, 1455, 1231, 1115, 1028, 806. HRMS (ESI) calcd. for $C_{27}H_{34}O_8H^+$: 383.2581, found: 383.2556.

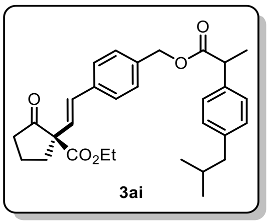
3ai

3ai: The compound was synthesized according to the general procedure, using*β*-ketoester (15.6 mg, 0.1 mmol) and corresponding alkene (97 mg, 0.3 mmol). The product was purified by column chromatography (PE/EA=3:1), white solid, 24.8 mg, 52% yield, 1:1 d.r., 89% *ee*, $[\alpha]_D^{20}$ = -27.0 (c = 2.48, $CHCl_3$). HPLC analysis: Daicel Chiralpak AD-H*2, hexane/iso-propanol = 95:5, flow rate = 0.8 mL/min, λ = 254 nm, retention time: 42.81 min (minor), 44.18 min (major), 45.74 min (minor) and 48.59 min (major). ^{1}H NMR (400 MHz, Chloroform-*d*) δ 7.32 (d, *J* = 8.0 Hz, 2H), 7.18 (t, *J* = 8.7 Hz, 4H), 7.09 (d, *J* = 7.8 Hz, 2H), 6.49 (d, *J* = 16.4 Hz, 1H), 6.39 (d, *J* = 16.3 Hz, 1H), 5.07 (d, *J* = 2.5 Hz, 2H), 4.20 (q, *J* = 7.1 Hz, 2H), 3.74 (q, *J* = 7.1 Hz, 1H), 2.75-2.69 (m, 1H), 2.50-2.35 (m, 4H), 2.33-2.25 (m, 1H), 2.14-1.93 (m, 2H), 1.88-1.80 (m, 1H), 1.50 (d, *J* = 7.2 Hz, 3H), 1.26 (t, *J* = 7.1 Hz, 3H), 0.90 (d, *J* = 6.6 Hz, 6H).^{13}C NMR (100 MHz, Chloroform-*d*) δ 212.4, 174.6, 170.3, 140.7, 137.7, 136.3, 135.9, 131.3, 129.5, 128.2, 127.3, 126.7, 126.3, 66.1, 63.0, 62.1, 45.3, 45.2, 37.7, 33.6, 30.3, 22.5, 19.7, 18.5, 14.2. IR (thin film, cm^{-1}): 2956, 2933, 2869, 1729, 1457, 1231, 1158, 750, 549. HRMS (ESI) calcd. for $C_{30}H_{36}O_5Na^+$: 499.2455, found: 499.2444.

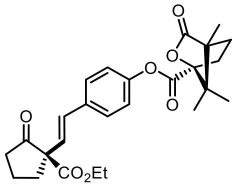
3aj

3aj: The compound was synthesized according to the general procedure, using corresponding *β*-ketoester (15.6 mg, 0.1 mmol) and alkene (90 mg, 0.3 mmol). The product was purified by column chromatography (PE/EA=3:1), white solid, 20.4 mg, 45% yield, 94% *de*, $[\alpha]_D^{20}$ = -6.3 (c = 1.80, $CHCl_3$). HPLC analysis: Daicel Chiralpak IA*2, hexane/iso-propanol = 85:15, flow rate = 0.8 mL/min, λ = 254 nm, retention time: 71.04 min (minor) and 74.35 min (major). ^{1}H NMR (400 MHz, Chloroform-*d*) δ 7.42 (d, *J* = 8.3 Hz, 2H), 7.08 (d, *J* = 8.3 Hz, 2H), 6.50 (d, *J* = 16.4 Hz, 1H), 6.39 (d, *J* = 16.3 Hz, 1H), 4.20 (q, *J* = 7.1 Hz, 2H), 2.76-2.69 (m, 1H), 2.61-2.15 (m, 5H), 2.11-1.95 (m, 3H), 1.80-1.73 (m, 1H), 1.26 (t, *J* = 7.0 Hz, 3H), 1.15 (d, *J* = 8.6 Hz, 6H), 1.10 (s, 3H).^{13}C NMR (100 MHz, Chloroform-*d*) δ 212.3, 177.9, 170.3, 166.2, 149.7, 135.0, 130.6, 127.8, 126.8, 121.6, 91.0, 63.0, 62.1, 55.0, 54.9, 37.7, 33.6, 30.9, 29.1, 19.7, 17.0, 17.0, 14.2, 9.9. IR (thin film, cm^{-1}): 2970, 2922, 1789, 1751, 1726, 1260, 1198, 1167, 1047, 750. HRMS (ESI) calcd. for $C_{26}H_{30}O_7Na^+$: 477.1884, found: 477.1872.

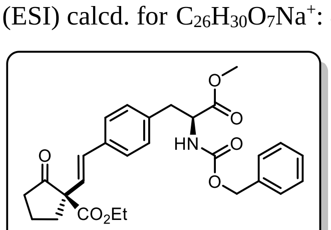
3ak

3ak: The compound was synthesized according to the general procedure, using corresponding *β*-ketoester (15.6 mg, 0.1 mmol) and alkene (101 mg, 0.3 mmol). The product was purified by column

chromatography (PE/EA=3:1), white solid, 27.1 mg, 55% yield, 94% *de*, $[\alpha]_D^{20}$ = +18.5 (c = 2.00, $CHCl_3$). HPLC analysis: Daicel Chiralpak OD-H, hexane/iso-propanol = 90:10, flow rate = 1.0 mL/min, λ = 260 nm, retention time: 50.38 min (major) and 66.17 min (minor). ^{1}H NMR (400 MHz, Chloroform-*d*) δ 7.39-7.27 (m, 7H), 7.04 (d, *J* = 7.8 Hz, 2H), 6.47 (d, *J* = 16.3 Hz, 1H), 6.37 (d, *J* = 16.4 Hz, 1H), 5.21 (d, *J* = 8.2 Hz, 1H), 5.09 (d, *J* = 3.9 Hz, 2H), 4.65 (q, *J* = 6.5 Hz, 1H), 4.20 (q, *J* = 7.3 Hz, 2H), 3.72 (s, 3H), 3.15-3.03 (m, 2H), 2.75-2.68 (m, 1H), 2.49-2.25 (m, 3H), 2.10-1.95 (m, 2H), 1.26 (t, *J* = 7.1 Hz, 3H). ^{13}C NMR (100 MHz, Chloroform-*d*) δ 212.4, 172.0, 170.4, 155.7, 136.3, 135.6, 135.5, 131.4, 129.6, 128.7, 128.3, 128.2, 126.9, 125.9, 67.1, 63.0, 62.0, 54.9, 52.5, 38.1, 37.7, 33.6, 19.7, 14.2. IR (thin film, cm^{-1}): 3356, 3031, 2954, 1746, 1717, 1513, 1213, 1026, 753, 699. HRMS (ESI) calcd. for $C_{28}H_{31}NO_7Na^+$: 516.1993, found: 516.1988.

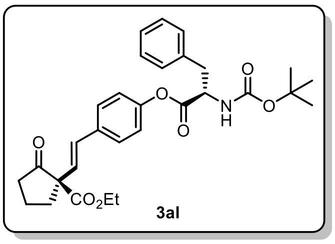

3al: The compound was synthesized according to the general procedure, using corresponding *β*-ketoester (15.6 mg, 0.1 mmol) and alkene (110 mg, 0.3 mmol). The product was purified by column chromatography (PE/EA=3:1), white solid, 21.3 mg, 41% yield, 92% *de*, $[\alpha]_D^{20}$ = -18.6 (c = 1.80, $CHCl_3$). HPLC analysis: Daicel Chiralpak OD-H*2, hexane/iso-propanol = 98:2, flow rate = 0.8 mL/min, λ = 254 nm, retention time: 53.75 min (major) and 63.45 min (minor). ^{1}H NMR (400 MHz, Chloroform-*d*) δ 7.38 (d, *J* = 8.4 Hz, 2H), 7.31 (dd, *J* = 14.5, 7.1 Hz, 3H), 7.23 (d, *J* = 6.4 Hz, 2H), 6.94 (d, *J* = 8.6 Hz, 2H), 6.49 (d, *J* = 16.4 Hz, 1H), 6.36 (d, *J* = 16.4 Hz, 1H), 5.06 (d, *J* = 8.2 Hz, 1H), 4.80 (q, *J* = 6.7 Hz, 1H), 4.20 (q, *J* = 7.1 Hz, 2H), 3.23 (d, *J* = 6.1 Hz, 2H), 2.75-2.69 (m, 1H), 2.52-2.35 (m, 2H), 2.33-2.24 (m, 1H), 2.11-1.94 (m, 2H), 1.44 (s, 9H), 1.26 (t, *J* = 7.1 Hz, 3H). ^{13}C NMR (100 MHz, Chloroform-*d*) δ 212.3, 170.7, 170.3, 155.3, 150.1, 135.8, 134.6, 130.7, 129.6, 128.9, 127.7, 127.4, 126.5, 121.6, 80.3, 63.0, 62.1, 54.8, 38.5, 37.7, 33.6, 28.4, 19.7, 14.2. IR (thin film, cm^{-1}): 2977, 2933, 1753, 1713, 1508, 1366, 1164, 749, 701. HRMS (ESI) calcd. for $C_{30}H_{35}NO_7Na^+$: 544.2306, found: 544.2301.

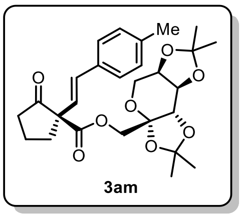

3am: The compound was synthesized according to the general procedure, using corresponding *β*-ketoester (36.9 mg, 0.1 mmol) and alkene (59 mg, 0.5 mmol). The product was purified by column chromatography (PE/EA=3:1), white solid, 10.2 mg, 21% yield, 91% *de*, $[\alpha]_D^{20}$ = +25.6 (c = 1.00, $CHCl_3$). HPLC analysis: Daicel Chiralpak IC, hexane/iso-propanol = 95:5, flow rate = 1.0 mL/min, λ = 254 nm, retention time: 22.60 min (major) and 26.90 min (minor). ^{1}H NMR (400 MHz, Chloroform-*d*) δ 7.10 (s, 4H), 6.68 (d, *J* = 12.0 Hz, 1H), 5.88 (d, *J* = 12.0 Hz, 1H), 4.55 (dd, *J* = 7.8, 2.6 Hz, 1H), 4.24-4.19 (m, 2H), 4.10 (d, *J* = 3.4 Hz, 2H), 3.92 (d, *J* = 13.0 Hz, 1H), 3.73 (d, *J* = 13.0 Hz, 1H), 2.64-2.55 (m, 1H), 2.46-2.38 (m, 1H), 2.33 (s, 3H), 2.30-2.16 (m, 2H), 1.99-1.92 (m, 1H), 1.87-1.79 (m, 1H), 1.53 (s, 3H), 1.47 (d, *J* = 3.9 Hz, 6H), 1.32 (s, 3H). ^{13}C NMR (100 MHz, Chloroform-*d*) δ 212.3, 169.4, 137.3, 133.9, 133.3, 128.9, 128.6, 128.3, 109.2, 109.2, 101.2, 71.0, 70.2, 70.1, 66.5, 62.5, 61.4, 37.1, 35.3, 26.8, 26.1,

25.7, 24.2, 21.4, 19.5. IR (thin film, cm^{-1}): 2988, 2935, 1754, 1725, 1376, 1206, 1069, 864, 736, 522. HRMS (ESI) calcd. for $C_{27}H_{34}O_8Na^+$: 509.2146, found: 509.2134.

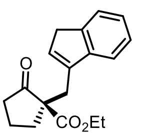
3an

3an: The compound was synthesized according to the general procedure, using corresponding *β*-ketoester (15.6 mg, 0.1 mmol) and alkene (65 mg, 0.5 mmol). The product was purified by column chromatography (PE/EA=5:1), colorless oil, 23.3 mg, 82% yield, 81% *ee*, $[\alpha]_D^{20}$ = -22.4 (c = 2.33, $CHCl_3$). HPLC analysis: Daicel Chiralpak IC, hexane/iso-propanol = 98:2, flow rate = 1.0 mL/min, λ = 254 nm, retention time: 23.77 min (major) and 27.38 min (minor). ^{1}H NMR (400 MHz, Chloroform-*d*) δ 7.44 (d, *J* = 7.3 Hz, 1H), 7.37 (d, *J* = 7.5 Hz, 1H), 7.29 (dd, *J* = 15.9, 8.4 Hz, 1H), 7.21 (t, *J* = 7.3 Hz, 1H), 6.23 (s, 1H), 4.17 (q, *J* = 7.1 Hz, 2H), 3.33 (s, 2H), 3.26 (d, *J* = 15.0 Hz, 1H), 2.98 (dd, *J* = 15.2, 1.5 Hz, 1H), 2.65-2.50 (m, 1H), 2.49-2.34 (m, 1H), 2.21-2.09 (m, 1H), 2.06-1.86 (m, 2H), 1.86-1.71 (m, 1H), 1.23 (t, *J* = 7.1 Hz, 3H).^{13}C NMR (100 MHz, Chloroform-*d*) δ 215.0, 171.1, 145.8, 144.0, 139.7, 131.8, 126.3, 124.9, 123.8, 119.3, 61.7, 60.8, 38.3, 38.2, 32.6, 30.8, 19.7, 14.2. IR (thin film, cm^{-1}): 3067, 3041, 2977, 2888, 1749, 1716, 1220, 1143, 770, 720. HRMS (ESI) calcd. for $C_{18}H_{20}O_3H^+$: 285.1485, found: 285.1478.

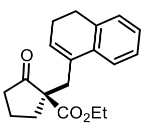
3ao

3ao: The compound was synthesized according to the general procedure, using corresponding *β*-ketoester (15.6 mg, 0.1 mmol) and alkene (72 mg, 0.5 mmol). The product was purified by column chromatography (PE/EA=5:1), colorless oil, 19.4 mg, 65% yield, 86% *ee*, $[\alpha]_D^{20}$ = -30.3 (c = 1.50, $CHCl_3$). HPLC analysis: Daicel Chiralpak OD-H, hexane/iso-propanol = 98:2, flow rate = 1.0 mL/min, λ = 263 nm, retention time: 8.68 min (major) and 9.39 min (minor). ^{1}H NMR (400 MHz, Chloroform-*d*) δ 7.26 (s, 1H), 7.21-7.09 (m, 3H), 5.87 (t, *J* = 4.6 Hz, 1H), 4.10 (q, *J* = 7.1 Hz, 2H), 3.23 (d, *J* = 14.7 Hz, 1H), 2.88 (d, *J* = 14.7 Hz, 1H), 2.74-2.62 (m, 2H), 2.46-2.33 (m, 2H), 2.22-2.17 (m, 2H), 2.12-2.03 (m, 1H), 1.93-1.71 (m, 3H), 1.22 (t, *J* = 7.1 Hz, 3H).^{13}C NMR (100 MHz, Chloroform-*d*) δ 215.2, 171.2, 136.6, 135.2, 132.6, 129.4, 127.7, 127.0, 126.4, 123.2, 61.6, 61.1, 38.3, 35.1, 32.5, 28.5, 23.3, 19.7, 14.2. IR (thin film, cm^{-1}): 3016, 2976, 2934, 2830, 1719, 1447, 1221, 1142, 1024, 739. HRMS (ESI) calcd. for $C_{19}H_{22}O_3Na^+$: 321.1461, found: 321.1453.

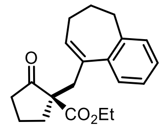
3ap

3ap: The compound was synthesized according to the general procedure, using corresponding *β*-ketoester (15.6 mg, 0.1 mmol) and alkene (79 mg, 0.5 mmol). The product was purified by column chromatography (PE/EA=5:1), colorless oil, 12.8 mg, 41% yield, 71% *ee*, $[\alpha]_D^{20}$ = +23.0 (c = 0.66, $CHCl_3$). HPLC analysis: Daicel Chiralpak IC, hexane/iso-propanol = 98:2, flow rate = 1.0 mL/min, λ = 254 nm, retention time: 19.27 min (minor) and 20.75 min (major). ^{1}H NMR (400 MHz, Chloroform-*d*) δ 7.25-7.10 (m, 4H), 6.01

(t, J = 7.2 Hz, 1H), 3.93-3.75 (m, 2H), 3.34 (d, J = 14.1 Hz, 1H), 2.65 (d, J = 14.1 Hz, 1H), 2.54 (t, J = 7.3 Hz, 2H), 2.38-2.26 (m, 2H), 2.17-2.01 (m, 3H), 1.85-1.63 (m, 5H), 1.15 (t, J = 7.2 Hz, 3H). ^{13}C NMR (100 MHz, Chloroform-*d*) δ 214.3, 169.9, 141.5, 140.5, 137.3, 130.8, 129.2, 127.2, 127.0, 126.1, 62.0, 61.7, 41.1, 37.9, 35.0, 32.5, 32.5, 24.9, 19.6, 14.3. IR (thin film, cm^{-1}): 2929, 2855, 2360, 1752, 1720, 1448, 1199, 754, 633. HRMS (ESI) calcd. for $C_{20}H_{24}O_3Na^+$: 335.1617, found: 335.1617.

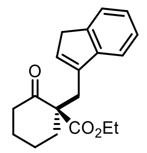

3aq

3aq: The compound was synthesized according to the general procedure, using corresponding *β*-ketoester (17.0 mg, 0.1 mmol) and alkene (65 mg, 0.5 mmol). The product was purified by column chromatography (PE/EA=5:1), colorless oil, 8.9 mg, 30% yield, 81% *ee*, $[\alpha]_D^{20}$ = +78.4 (c = 0.70, $CHCl_3$). HPLC analysis: Daicel Chiralpak OJ, hexane/iso-propanol = 95:5, flow rate = 1.0 mL/min, λ = 254 nm, retention time: 11.77 min (major) and 16.51 min (minor). ^{1}H NMR (400 MHz, Chloroform-*d*) δ 7.42 (d, J = 7.3 Hz, 1H), 7.32 (d, J = 7.9 Hz, 1H), 7.29-7.24 (m, 1H), 7.17 (t, J = 7.3 Hz, 1H), 6.25 (s, 1H), 4.09-4.01 (m, 2H), 3.32 (s, 2H), 3.23 (d, J = 14.7 Hz, 1H), 2.90 (d, J = 14.7 Hz, 1H), 2.60-2.53 (m, 1H), 2.51-2.41 (m, 2H), 2.06-1.99 (m, 1H), 1.77-1.60 (m, 3H), 1.55-1.51 (m, 1H), 1.13 (t, J = 7.1 Hz, 3H).^{13}C NMR (100 MHz, Chloroform-*d*) δ 207.7, 171.7, 146.2, 144.2, 139.5, 132.6, 126.2, 124.8, 124.0, 119.5, 61.7, 41.5, 38.4, 36.6, 31.8, 28.0, 22.9, 14.2. IR (thin film, cm^{-1}): 2937, 2866, 2360, 2341, 1713, 1453, 1189, 1022, 771. HRMS (ESI) calcd. for $C_{19}H_{22}O_3H^+$: 299.1642, found: 299.1638.

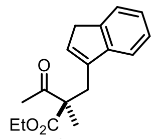

3ar

3ar: The compound was synthesized according to the general procedure, using corresponding *β*-ketoester (14.4 mg, 0.1 mmol) and alkene (65 mg, 0.5 mmol). The product was purified by column chromatography (PE/EA=5:1), colorless oil, 9.5 mg, 35% yield, 79% *ee*, $[\alpha]_D^{20}$ = +29.0 (c = 0.80, $CHCl_3$). HPLC analysis: Daicel Chiralpak OJ, hexane/iso-propanol = 99:1, flow rate = 1.0 mL/min, λ = 254 nm, retention time: 25.65 min (minor) and 27.23 min (major). ^{1}H NMR (400 MHz, Chloroform-*d*) δ 7.44 (d, J = 7.3 Hz, 1H), 7.35 (d, J = 7.5 Hz, 1H), 7.29 (t, J = 7.5 Hz, 1H), 7.19 (t, J = 7.3 Hz, 1H), 6.20 (s, 1H), 4.21-4.09 (m, 2H), 3.33 (s, 2H), 3.23 (d, J = 15.3 Hz, 1H), 3.09 (d, J = 15.2 Hz, 1H), 2.20 (s, 3H), 1.41 (s, 3H), 1.22 (t, J = 7.1 Hz, 3H). ^{13}C NMR (100 MHz, Chloroform-*d*) δ 205.8, 173.0, 146.1, 144.1, 139.5, 131.6, 126.3, 125.0, 124.0, 119.5, 61.8, 60.4, 38.5, 32.0, 26.6, 19.8, 14.3. IR (thin film, cm^{-1}): 2983, 2936, 2360, 2341, 1712, 1459, 1185, 1097, 772, 558. HRMS (ESI) calcd. for $C_{17}H_{20}O_3H^+$: 273.1485, found: 273.1483.

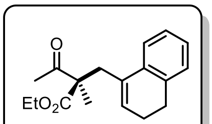

3as

3as: The compound was synthesized according to the general procedure, using corresponding *β*-ketoester (14.4 mg, 0.1 mmol) and alkene (72 mg, 0.5 mmol). The product was purified by column chromatography

(PE/EA=5:1), colorless oil, 10.9 mg, 38% yield, 82% *ee*, $[\alpha]_D^{20}$ = +28.8 (c = 0.90, $CHCl_3$). HPLC analysis: Daicel Chiralpak IC-H, hexane/iso-propanol = 98:2, flow rate = 1.0 mL/min, λ = 254 nm, retention time: 12.14 min (major) and 13.71 min (minor). ^1H NMR (400 MHz, Chloroform-*d*) δ 7.23 (d, *J* = 7.4 Hz, 1H), 7.17-7.10 (m, 3H), 5.86 (t, *J* = 4.6 Hz, 1H), 4.06-3.87 (m, 2H), 3.18 (d, *J* = 14.6 Hz, 1H), 2.94 (d, *J* = 14.7 Hz, 1H), 2.68 (t, *J* = 8.1 Hz, 2H), 2.22-2.16 (m, 2H), 2.14 (s, 3H), 1.26 (s, 3H), 1.18 (t, *J* = 7.2 Hz, 3H). ^{13}C NMR (100 MHz, Chloroform-*d*) δ 205.9, 172.9, 137.0, 135.4, 132.7, 129.9, 127.9, 127.1, 126.4, 123.2, 61.6, 60.4, 36.0, 28.9, 26.8, 23.6, 19.8, 14.2. IR (thin film, cm^{-1}): 2982, 2935, 2360, 2341, 1740, 1712, 1449, 1102, 762. HRMS (ESI) calcd. for $C_{18}H_{22}O_3Na^+$: 309.1461, found: 309.1456.

5. NMR spectra

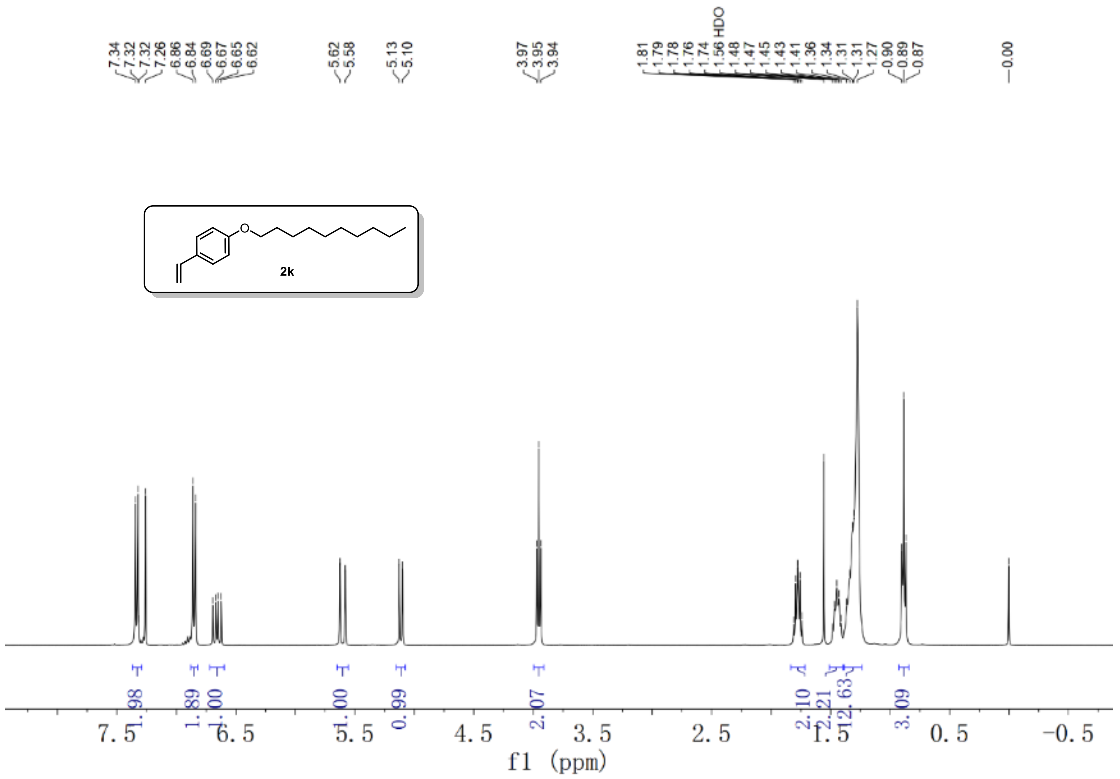

Supplementary Fig. 12. ^{1}H NMR spectra of compound **2k**. (400 MHz, 298K) in $CDCl_3$

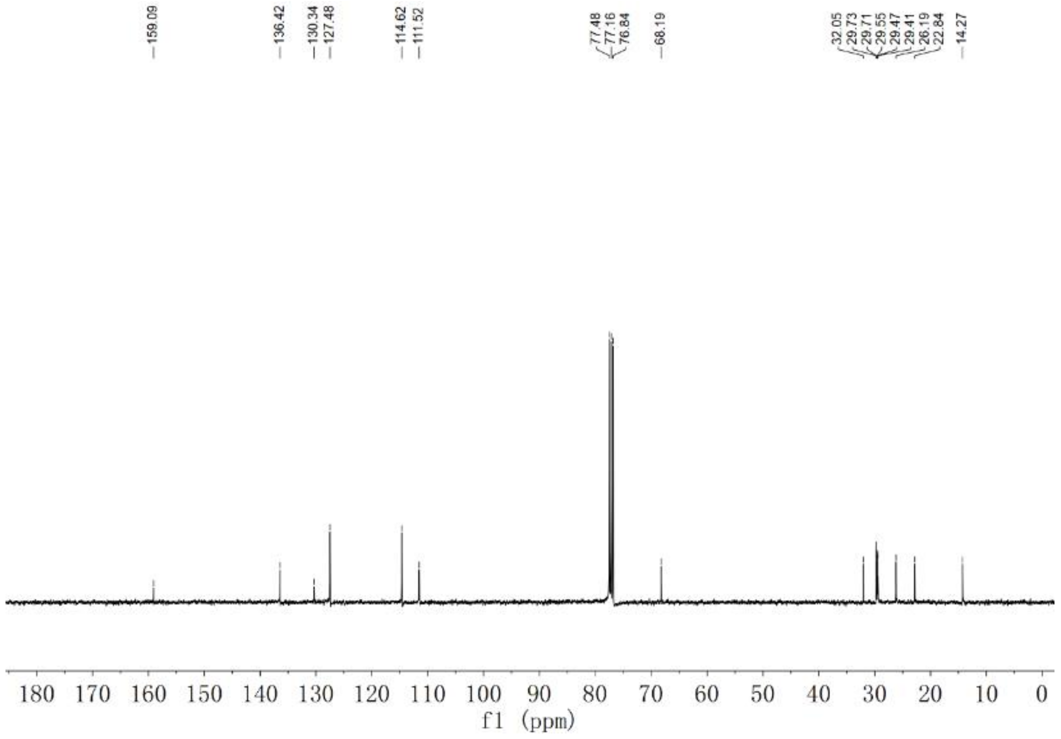

Supplementary Fig. 13. ^{13}C NMR spectra of compound **2k**. (100 MHz, 298K) in $CDCl_3$

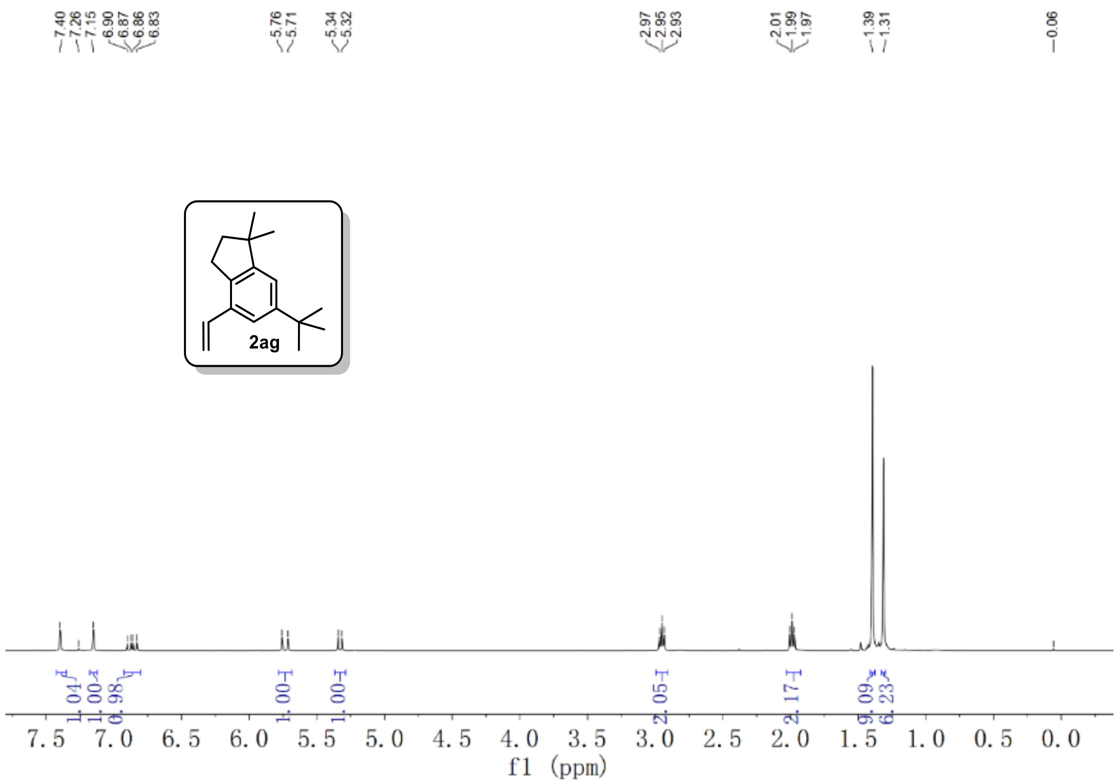

Supplementary Fig. 14. ^{1}H NMR spectra of compound **2ag**. (400 MHz, 298K) in $CDCl_3$

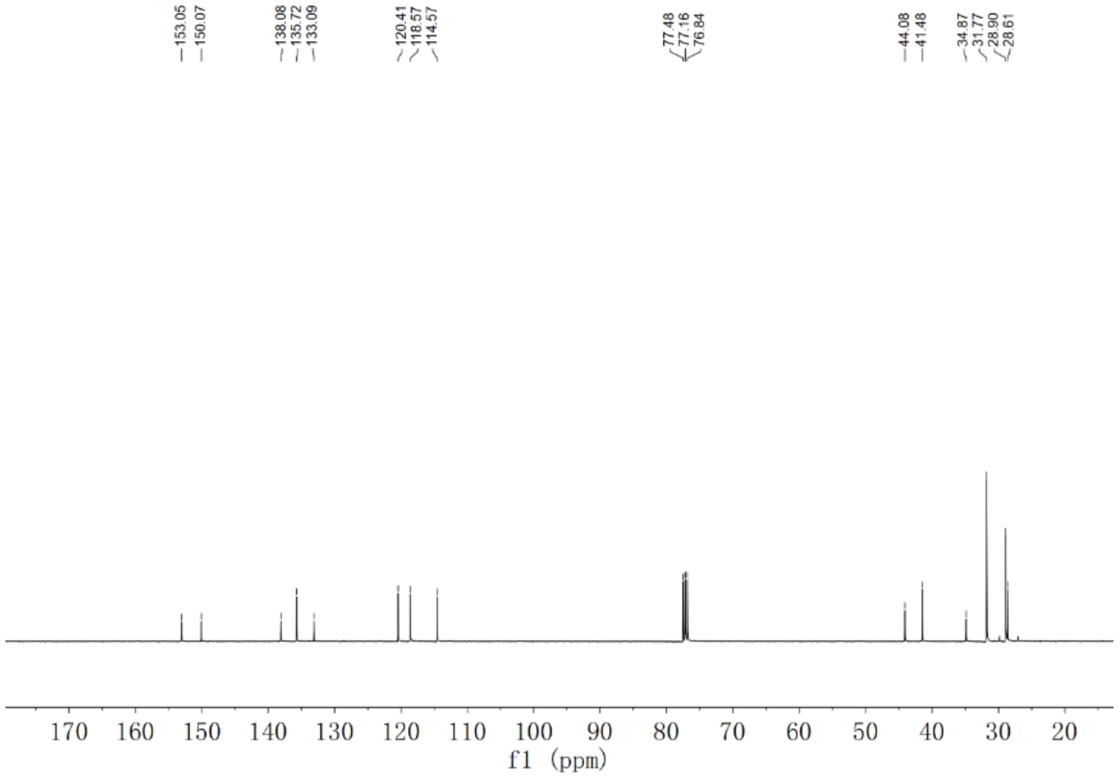

Supplementary Fig. 15. ^{13}C NMR spectra of compound **2ag**. (100 MHz, 298K) in $CDCl_3$

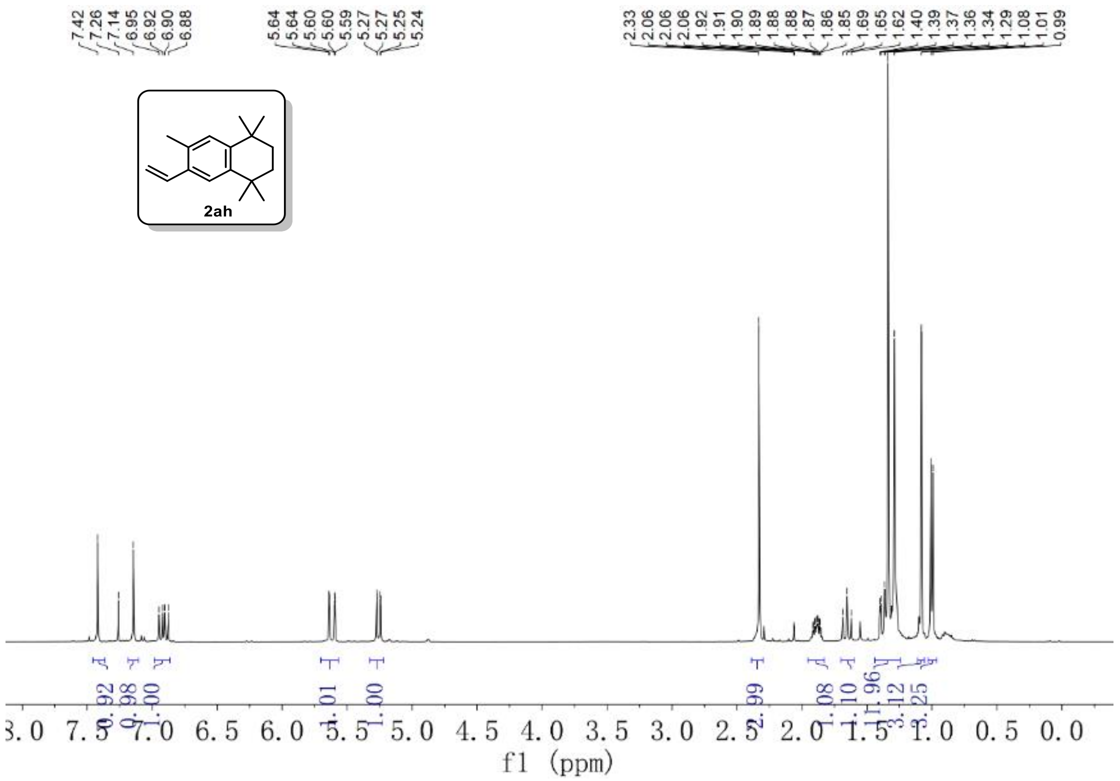

Supplementary Fig. 16. ^{1}H NMR spectra of compound **2ah**. (400 MHz, 298K) in $CDCl_3$

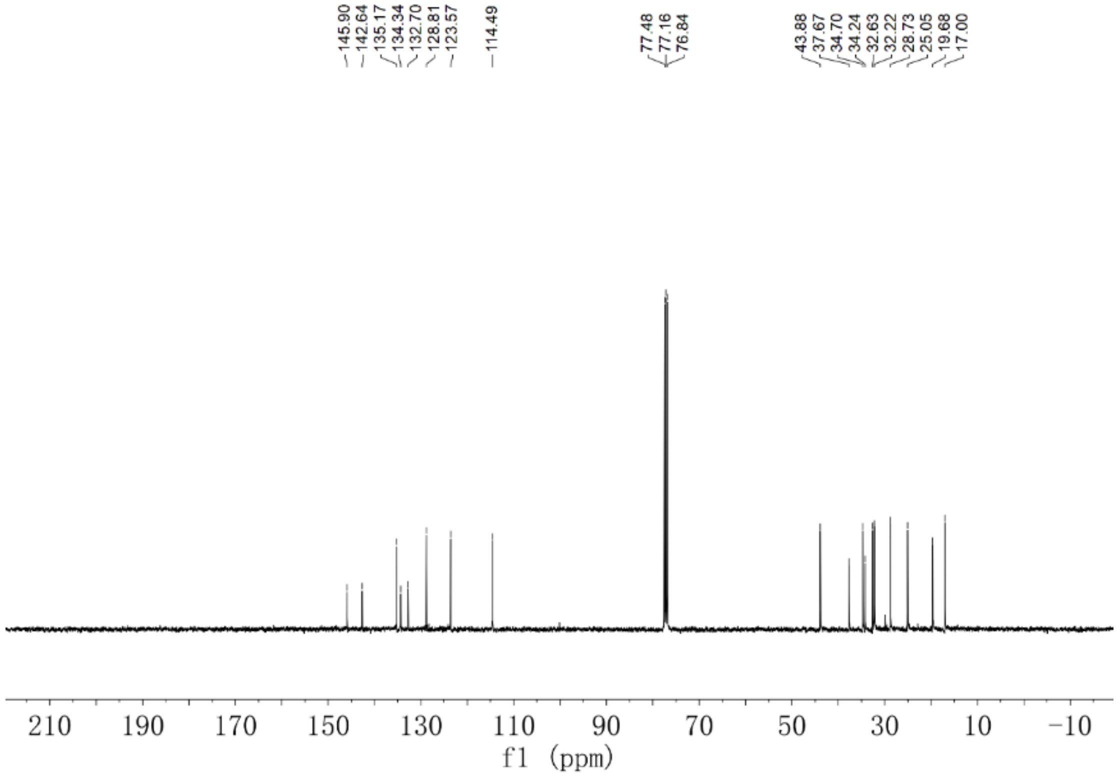

Supplementary Fig. 17. ^{13}C NMR spectra of compound **2ah**. (100 MHz, 298K) in $CDCl_3$

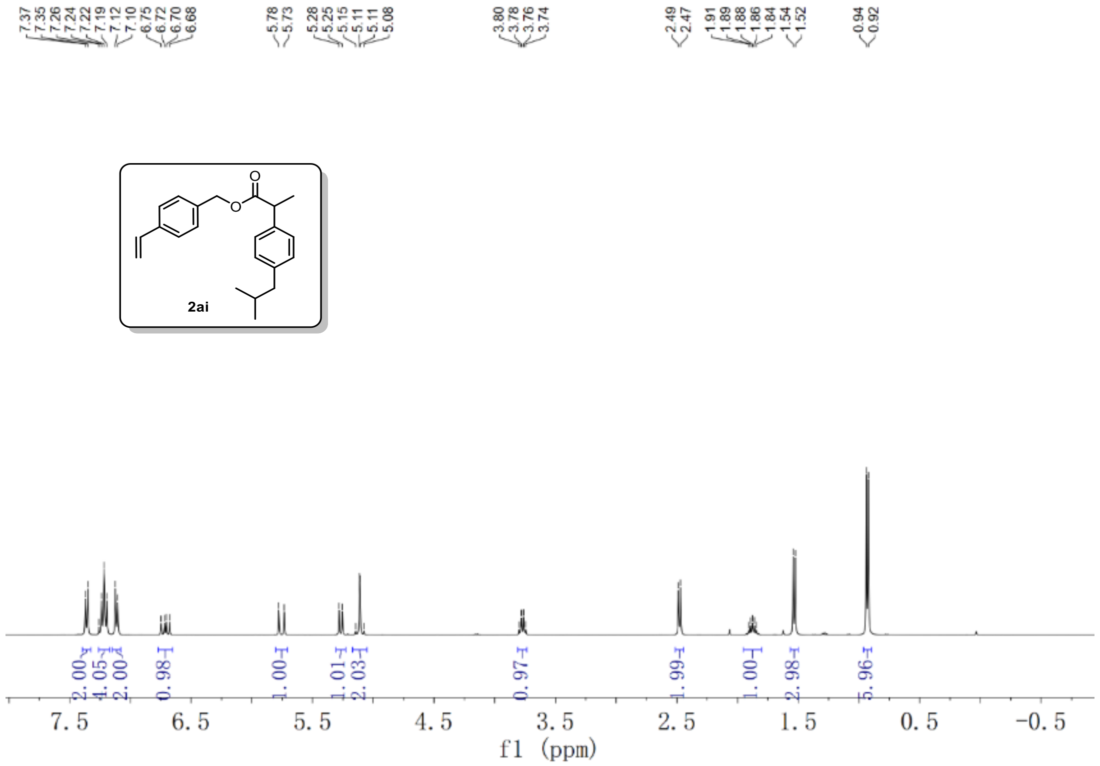

Supplementary Fig. 18. ^{1}H NMR spectra of compound **2ai**. (400 MHz, 298K) in $CDCl_3$

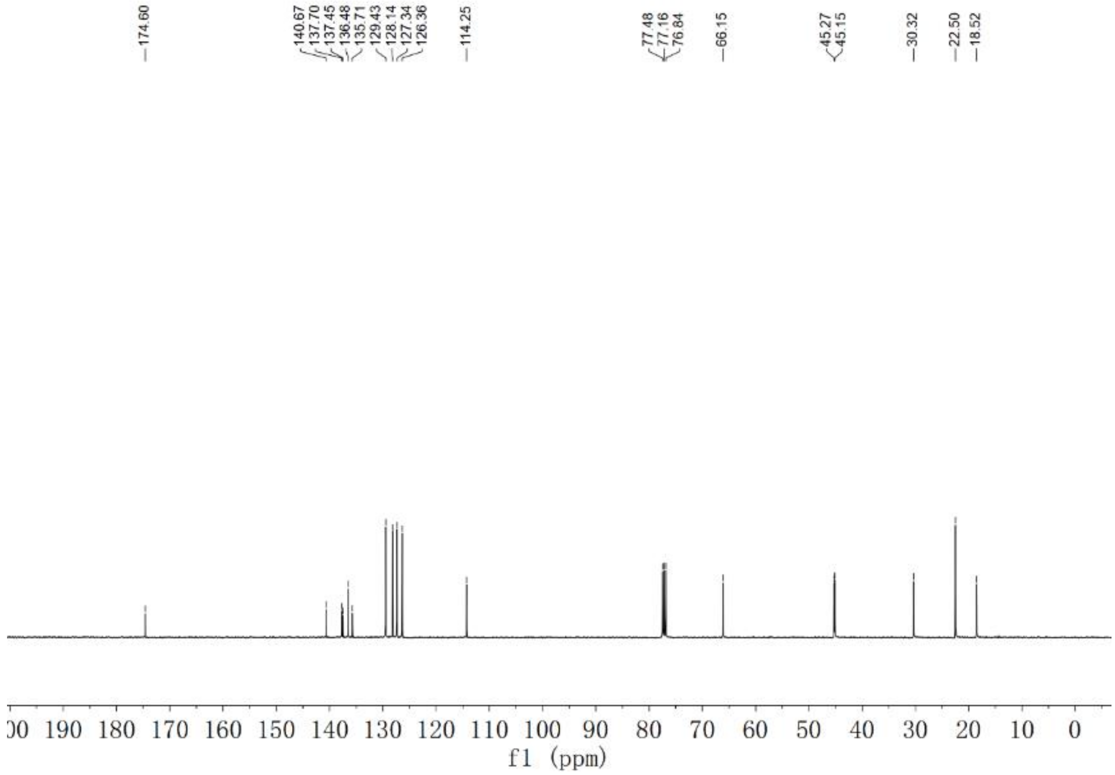

Supplementary Fig. 19. ^{13}C NMR spectra of compound **2ai**. (100 MHz, 298K) in $CDCl_3$

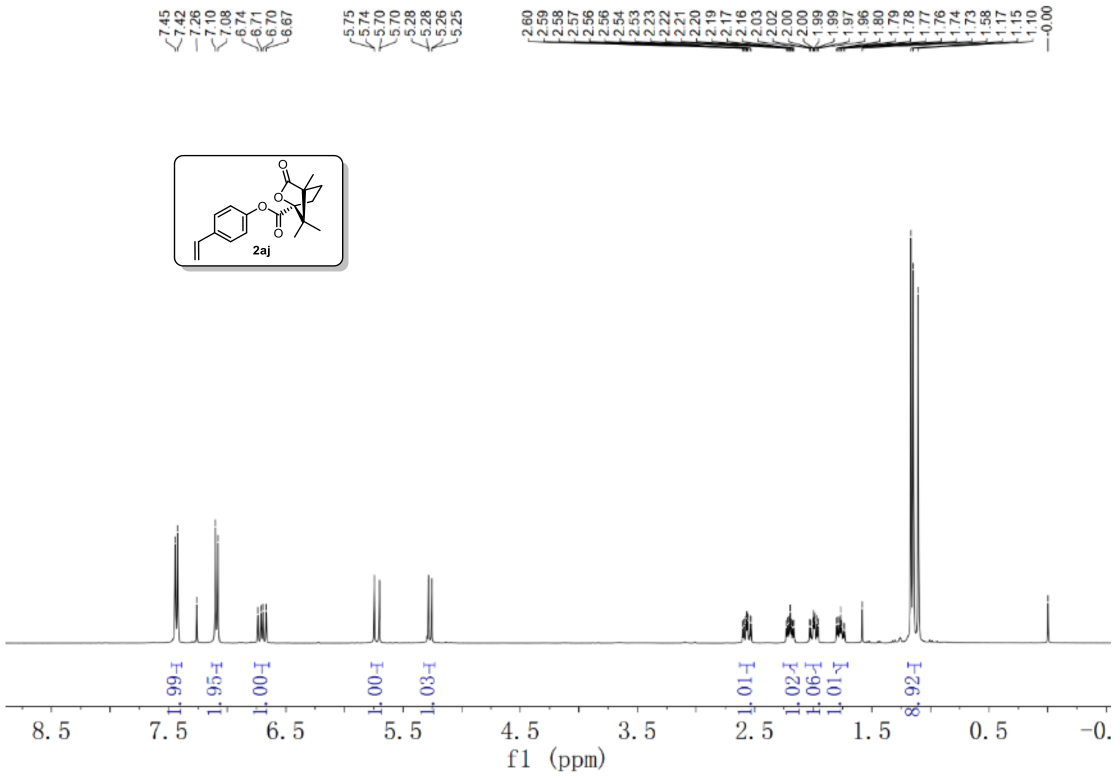

Supplementary Fig. 20. ^{1}H NMR spectra of compound **2aj**. (400 MHz, 298K) in $CDCl_3$

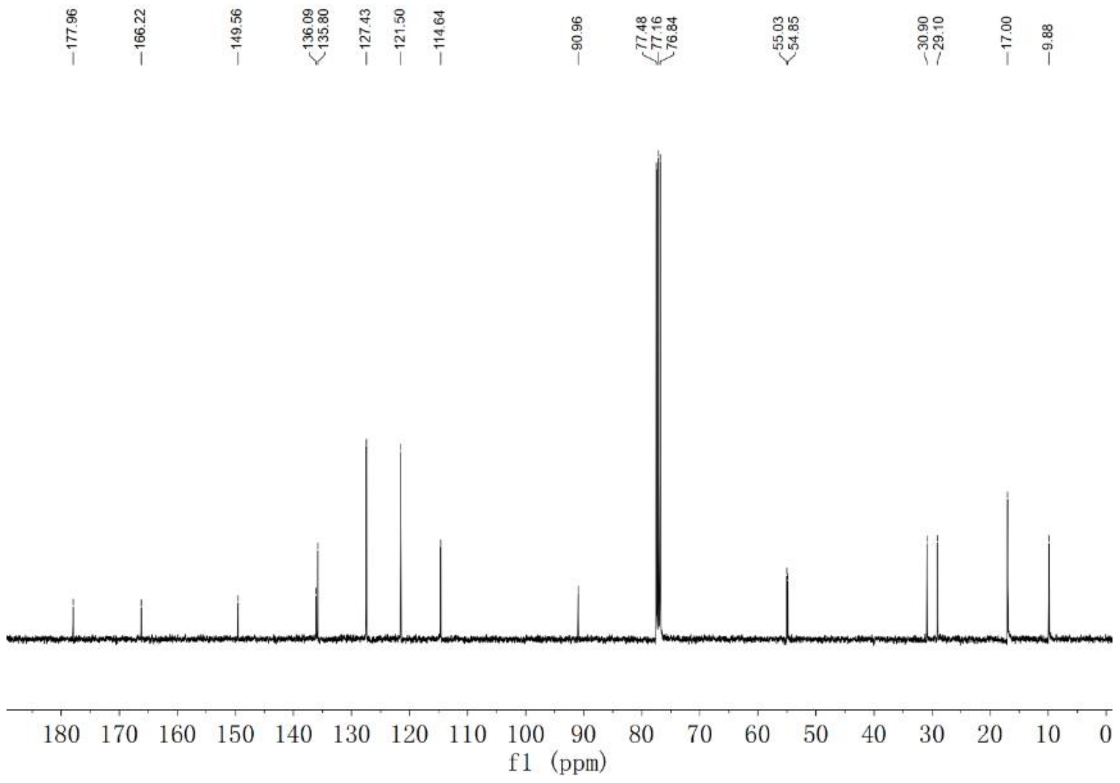

Supplementary Fig. 21. ^{13}C NMR spectra of compound **2aj**. (100 MHz, 298K) in $CDCl_3$

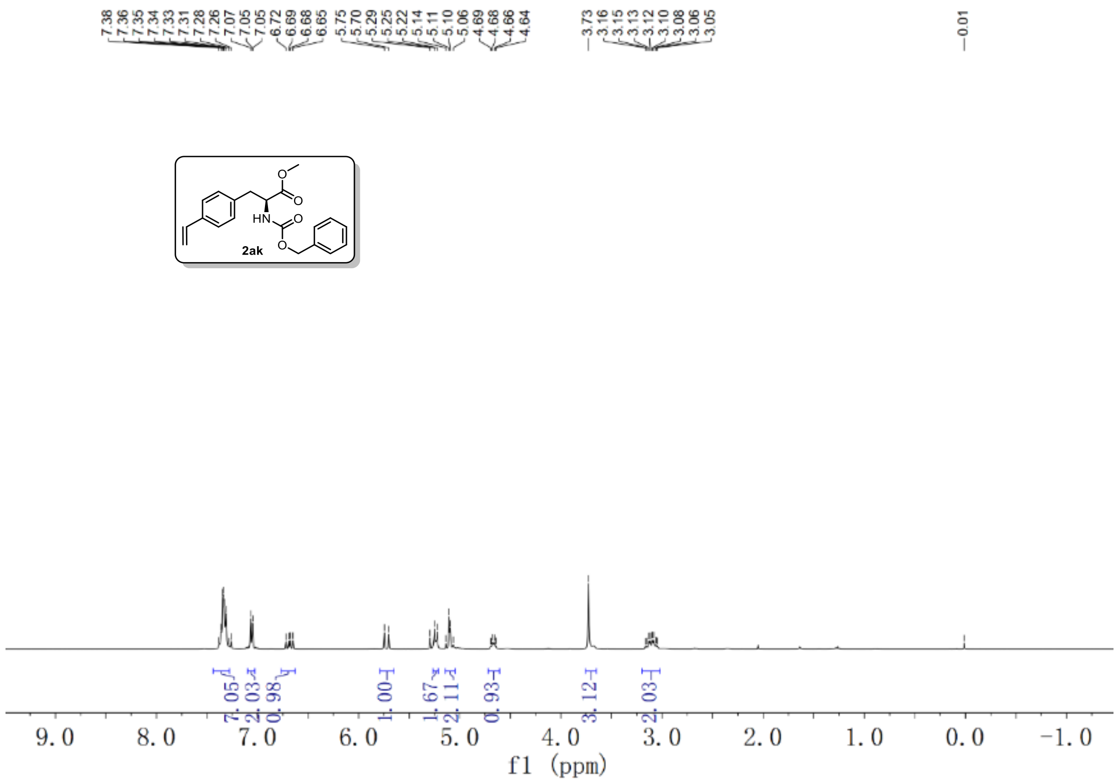

Supplementary Fig. 22. ^{1}H NMR spectra of compound **2ak**. (400 MHz, 298K) in $CDCl_3$

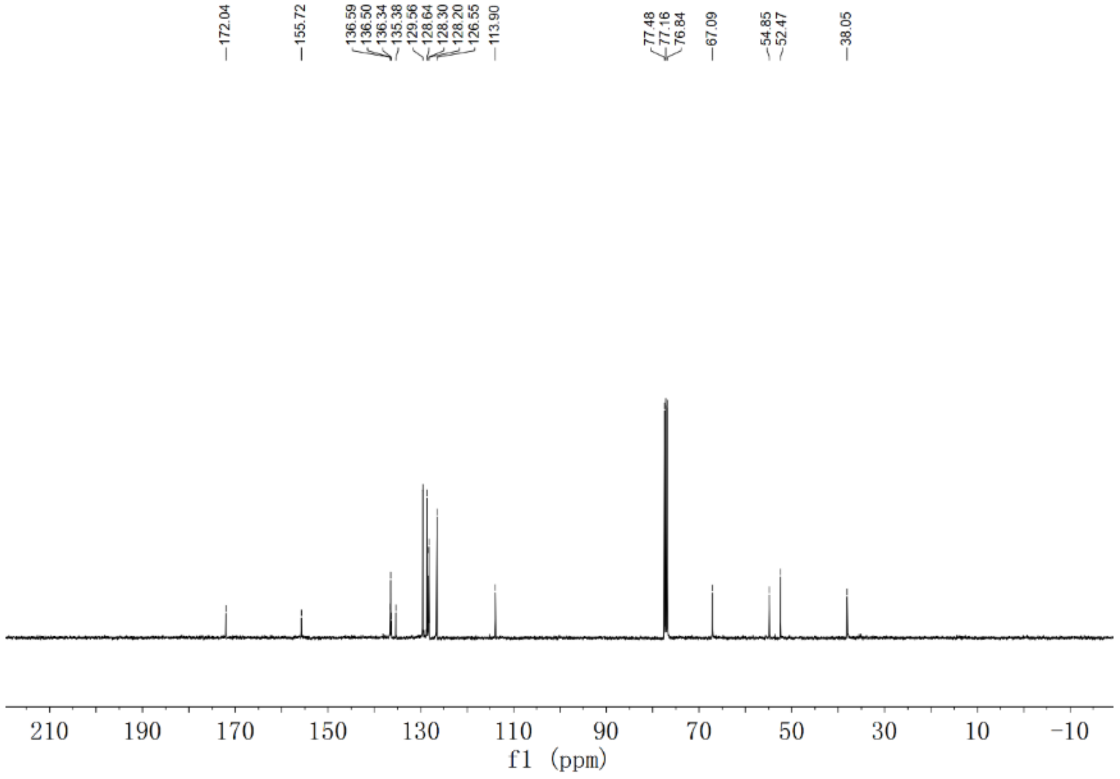

Supplementary Fig.23. ^{13}C NMR spectra of compound **2ak**. (100 MHz, 298K) in $CDCl_3$

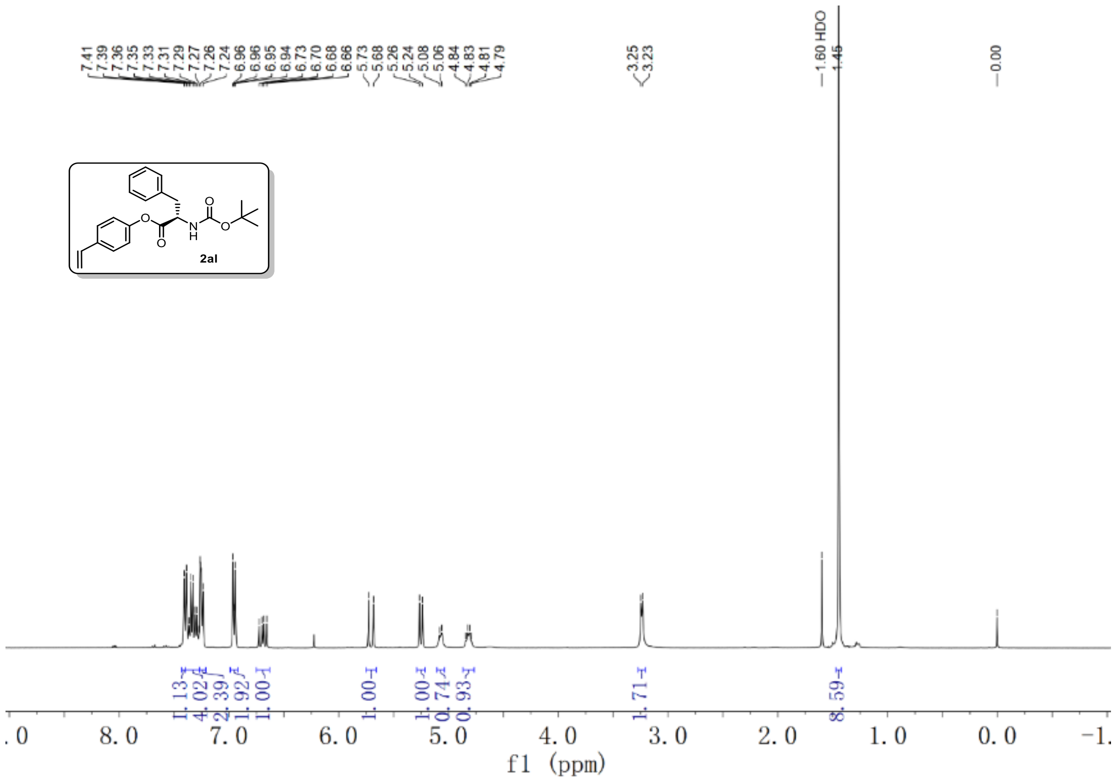

Supplementary Fig. 24. ^{1}H NMR spectra of compound **2al**. (400 MHz, 298K) in $CDCl_3$

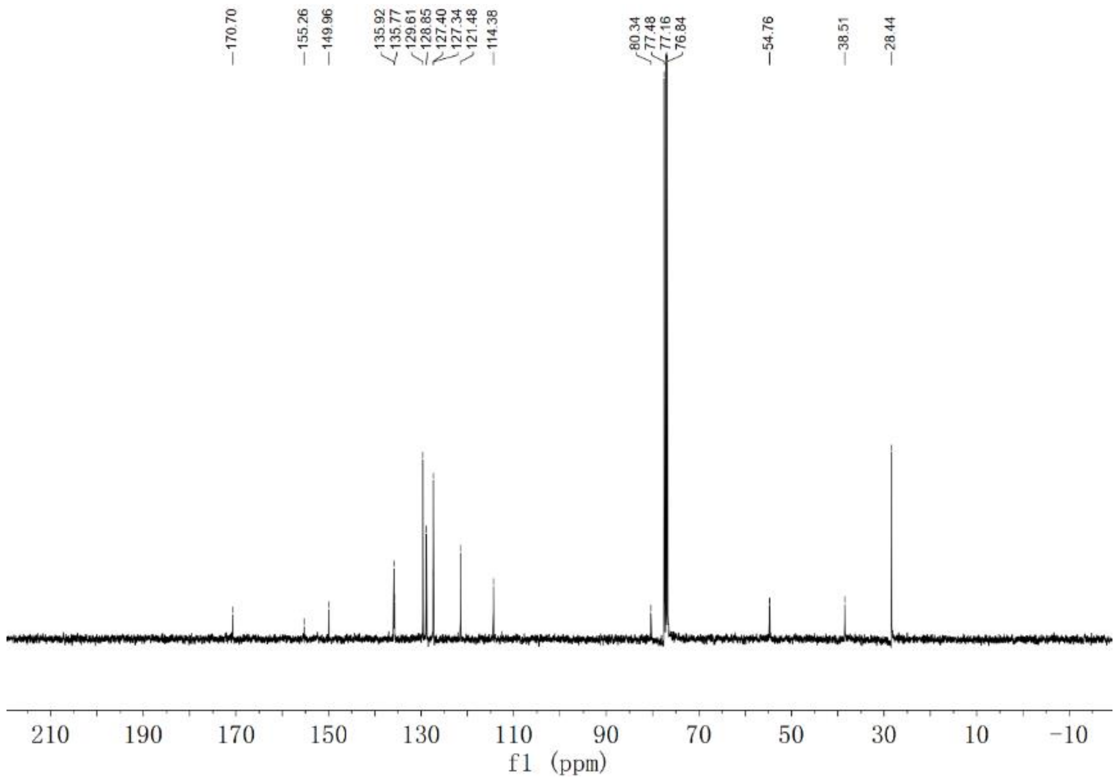

Supplementary Fig. 25. ^{13}C NMR spectra of compound **2al**. (100 MHz, 298K) in $CDCl_3$

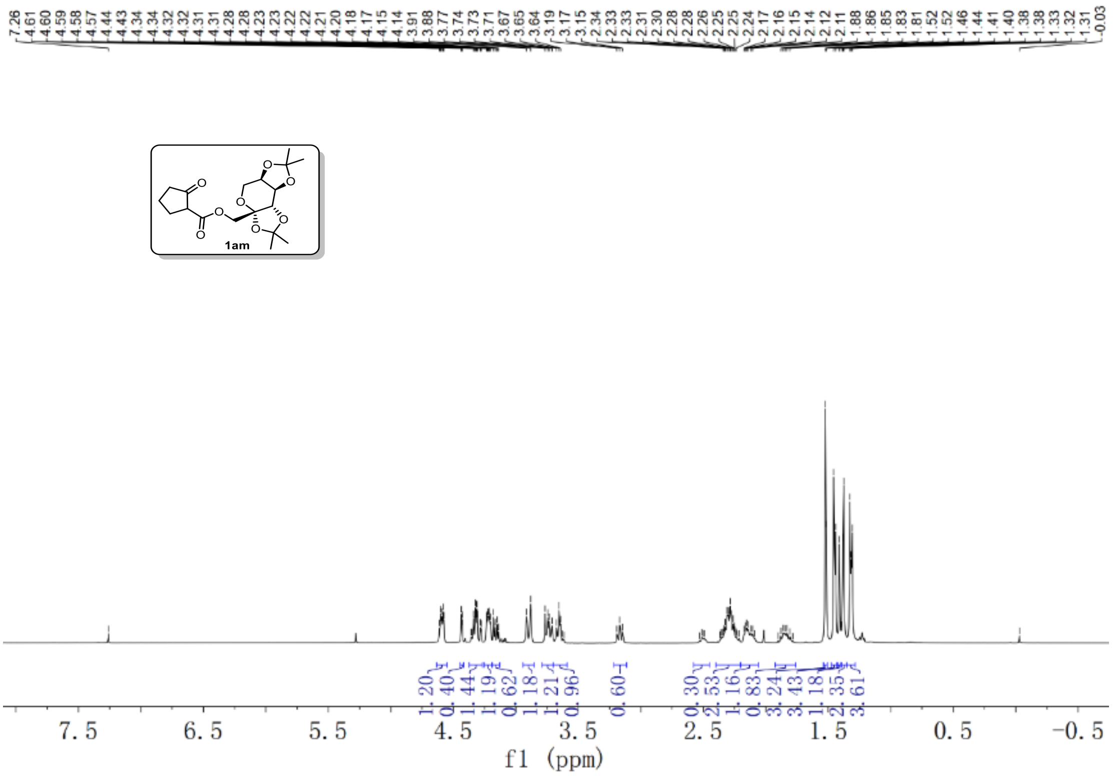

Supplementary Fig. 26. ^{1}H NMR spectra of compound **1am**. (400 MHz, 298K) in $CDCl_3$

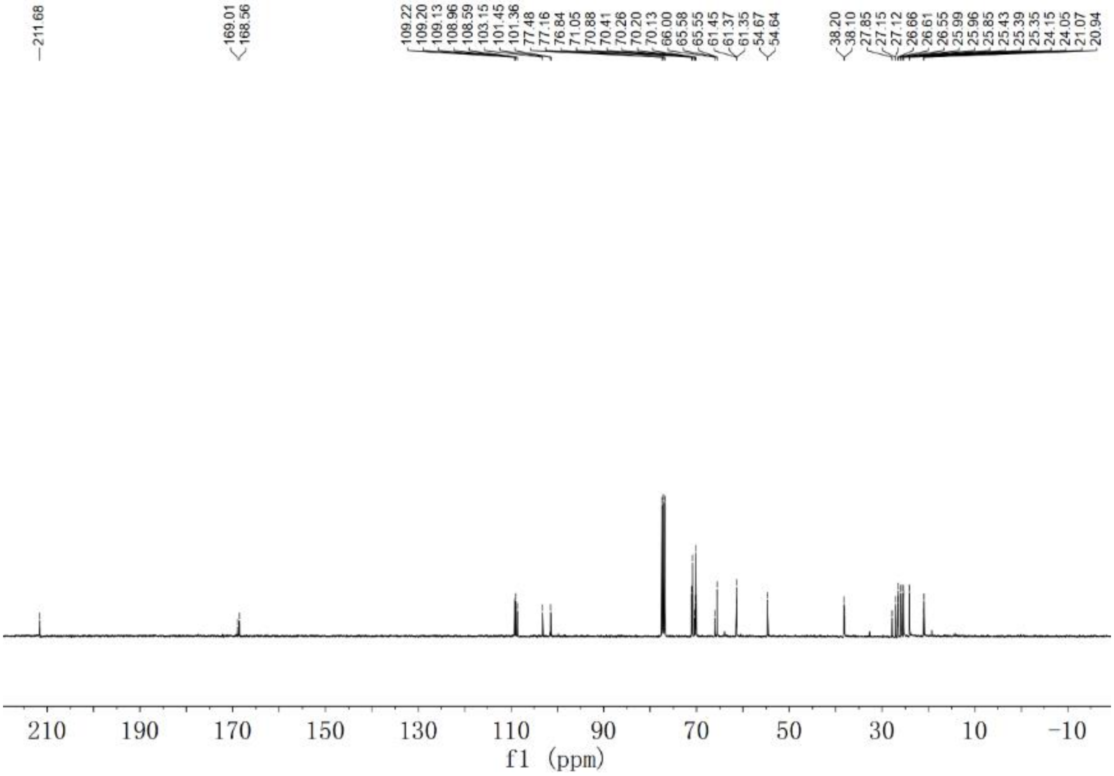

Supplementary Fig. 27. ^{13}C NMR spectra of compound **1am**. (100 MHz, 298K) in $CDCl_3$

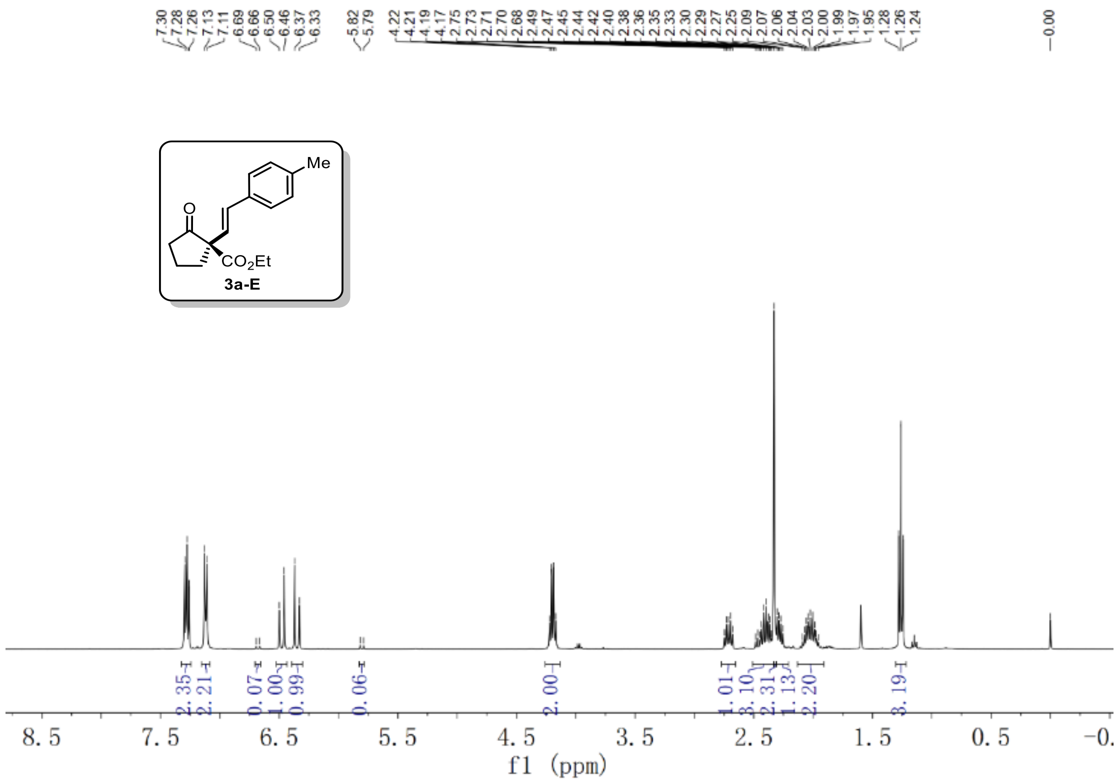

Supplementary Fig. 28. ^{1}H NMR spectra of compound **3a-*E***. (400 MHz, 298K) in $CDCl_3$

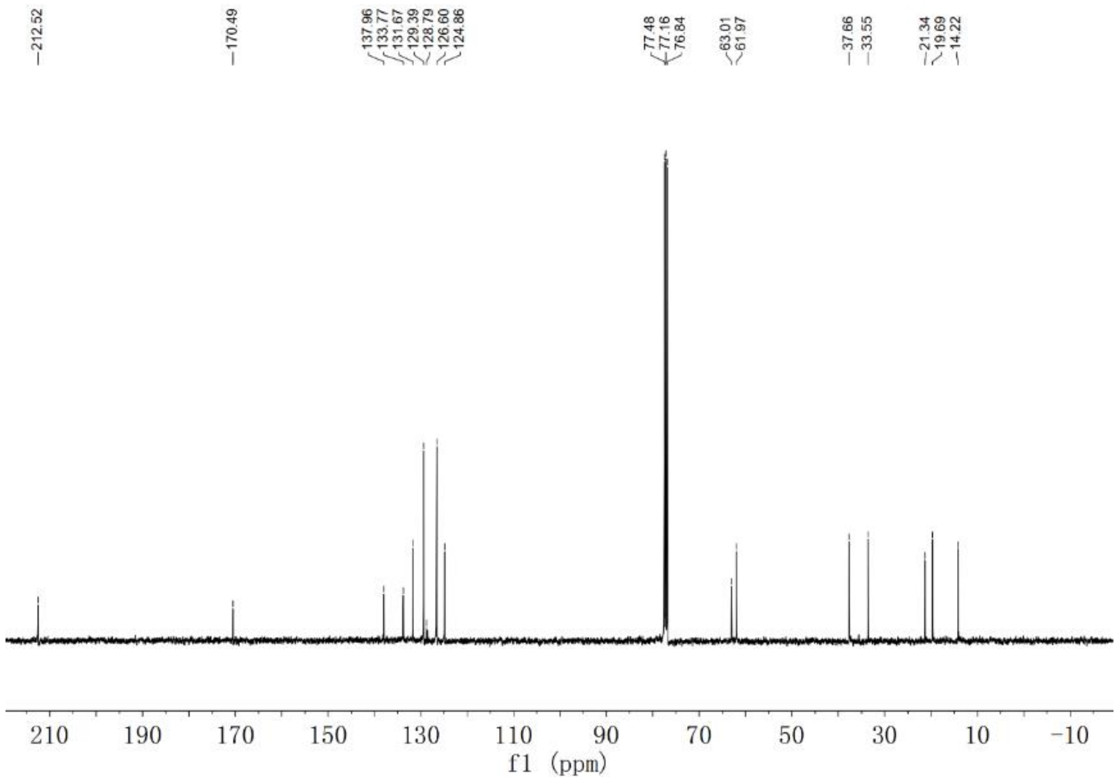

Supplementary Fig. 29. ^{13}C NMR spectra of compound **3a-*E***. (100 MHz, 298K) in $CDCl_3$

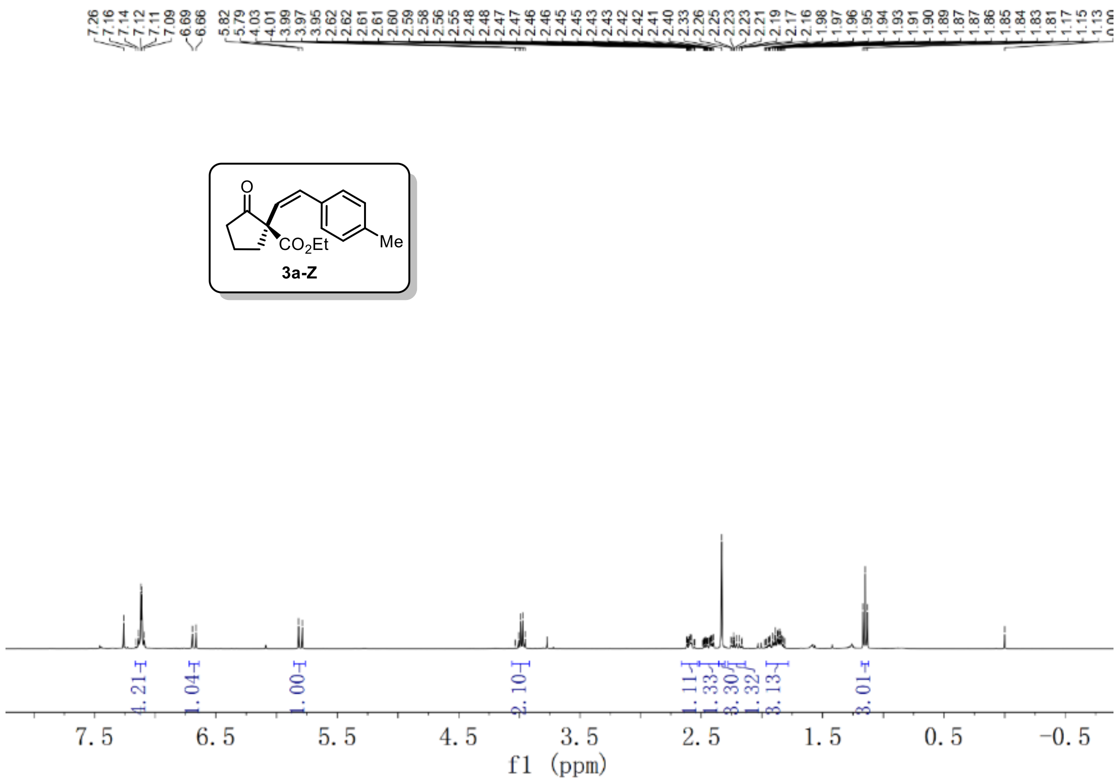

Supplementary Fig. 30. ^{1}H NMR spectra of compound **3a-*Z***. (400 MHz, 298K) in $CDCl_3$

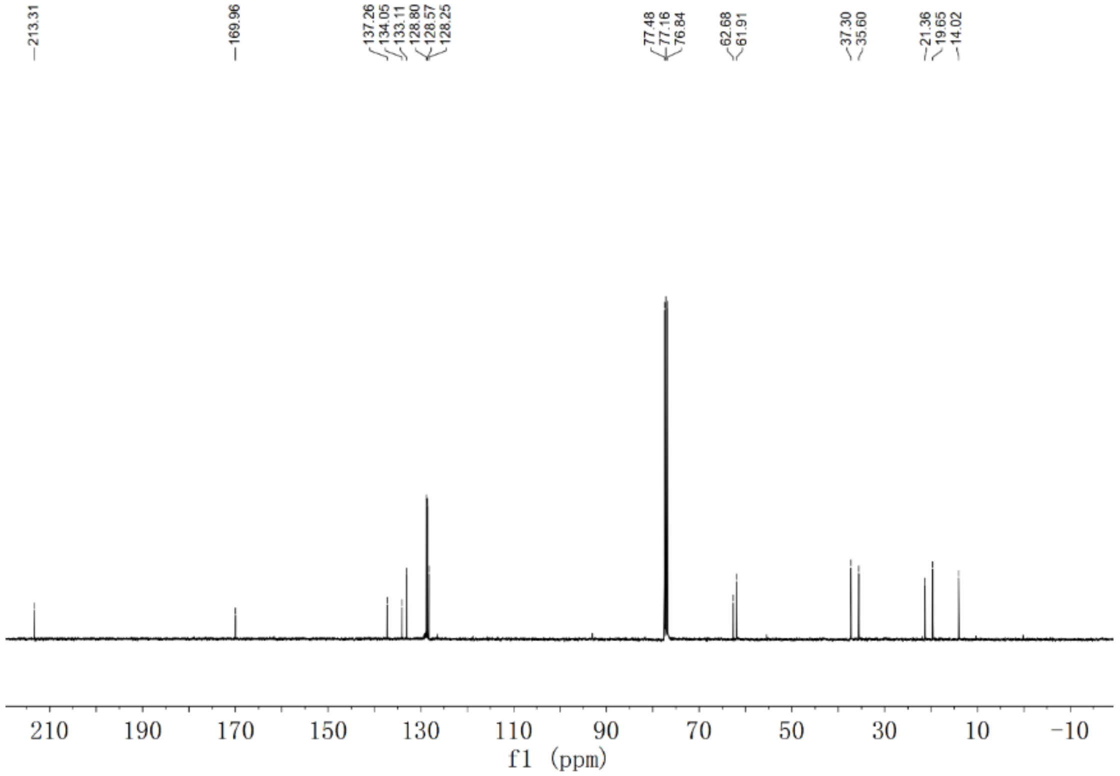

Supplementary Fig. 31. ^{13}C NMR spectra of compound **3a-*Z***. (100 MHz, 298K) in $CDCl_3$

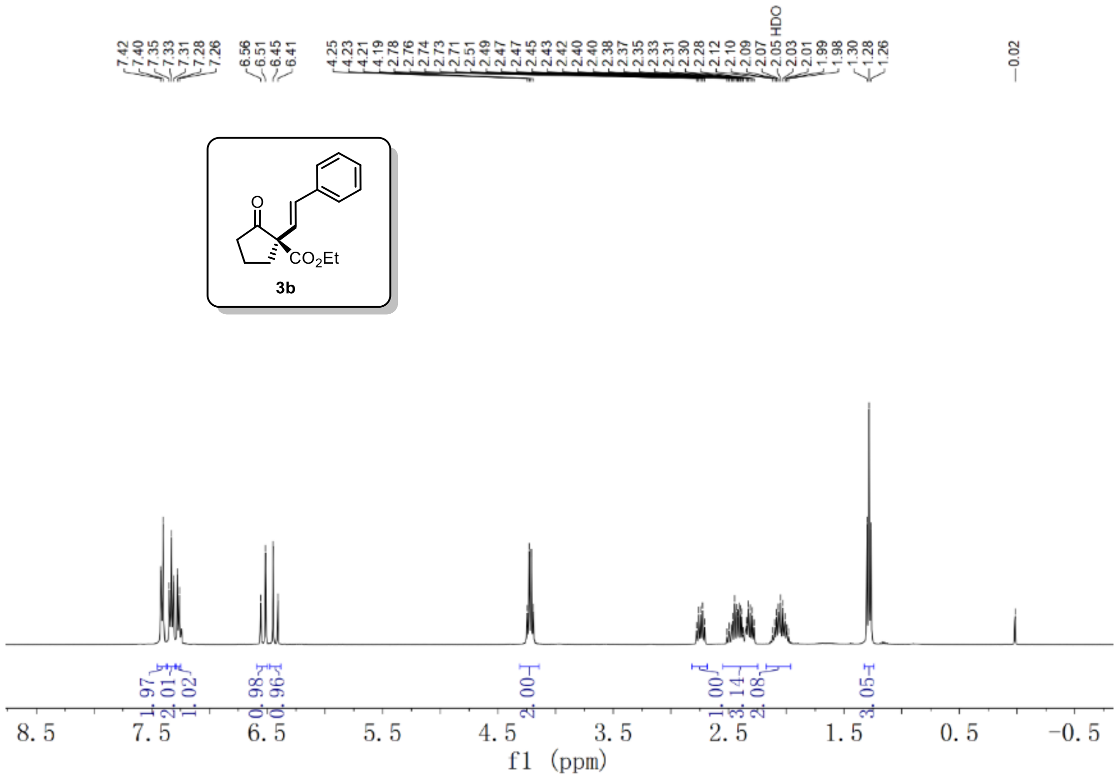

Supplementary Fig. 32. ^{1}H NMR spectra of compound **3b**. (400 MHz, 298K) in $CDCl_3$

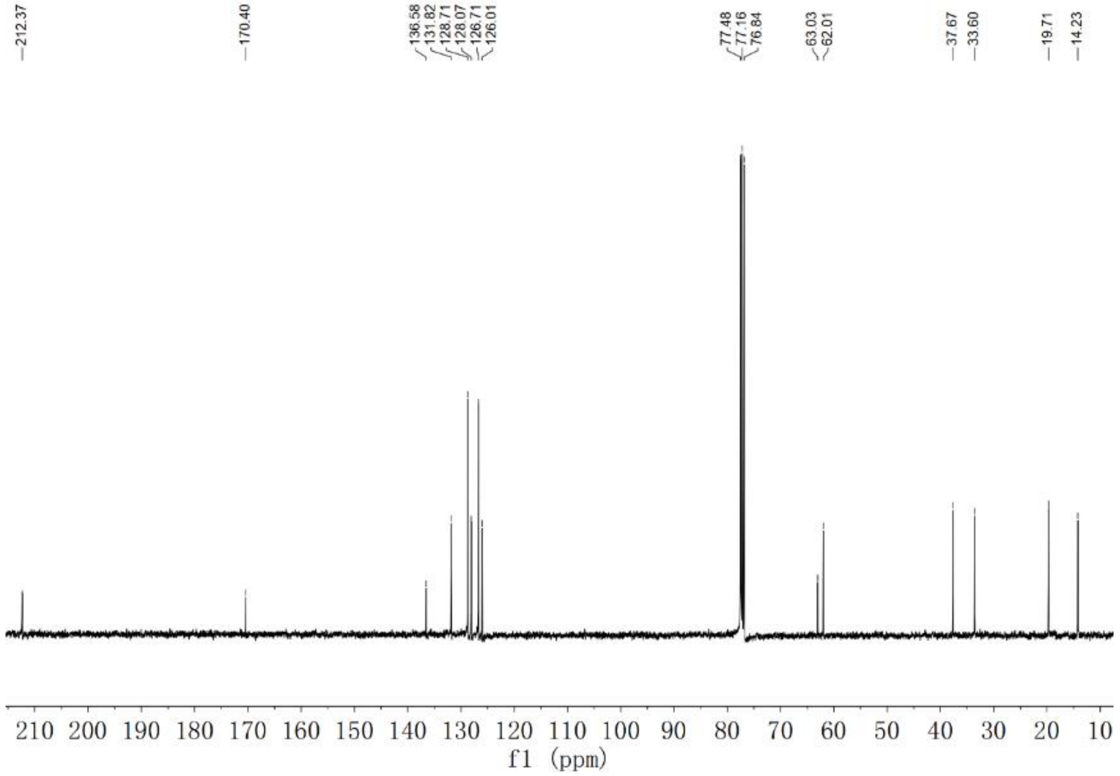

Supplementary Fig. 33. ^{13}C NMR spectra of compound **3b**. (100 MHz, 298K) in $CDCl_3$

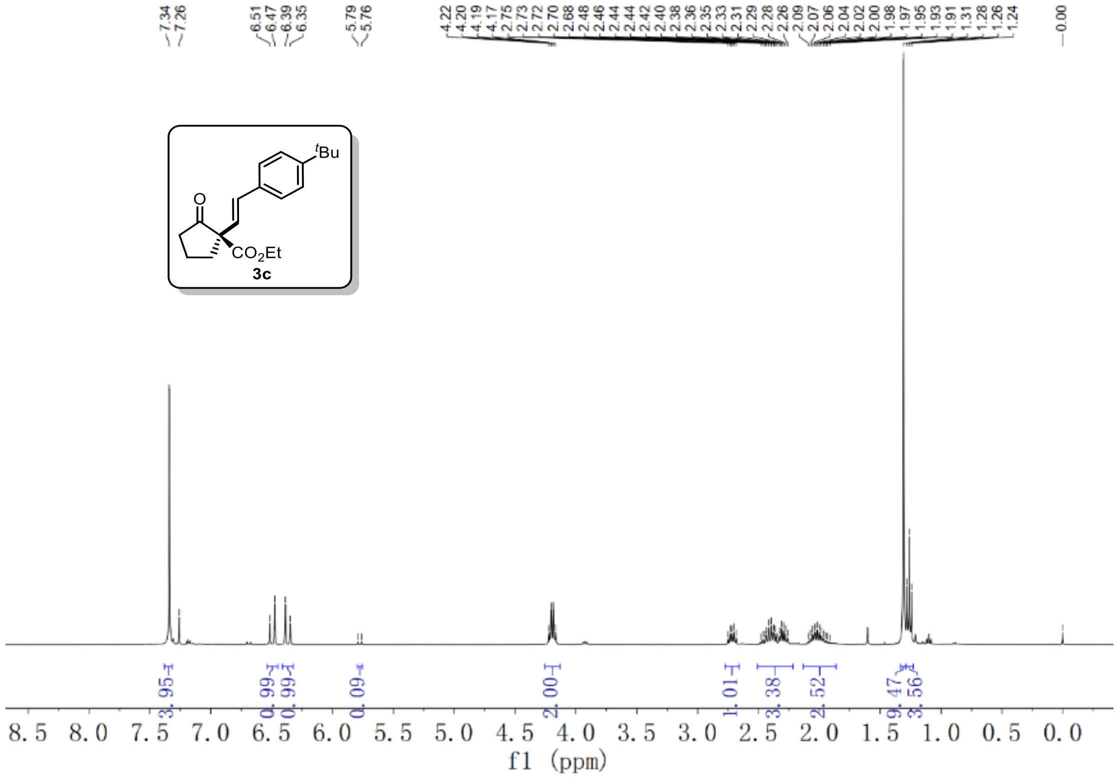

Supplementary Fig. 34. ^{1}H NMR spectra of compound **3c**. (400 MHz, 298K) in $CDCl_3$

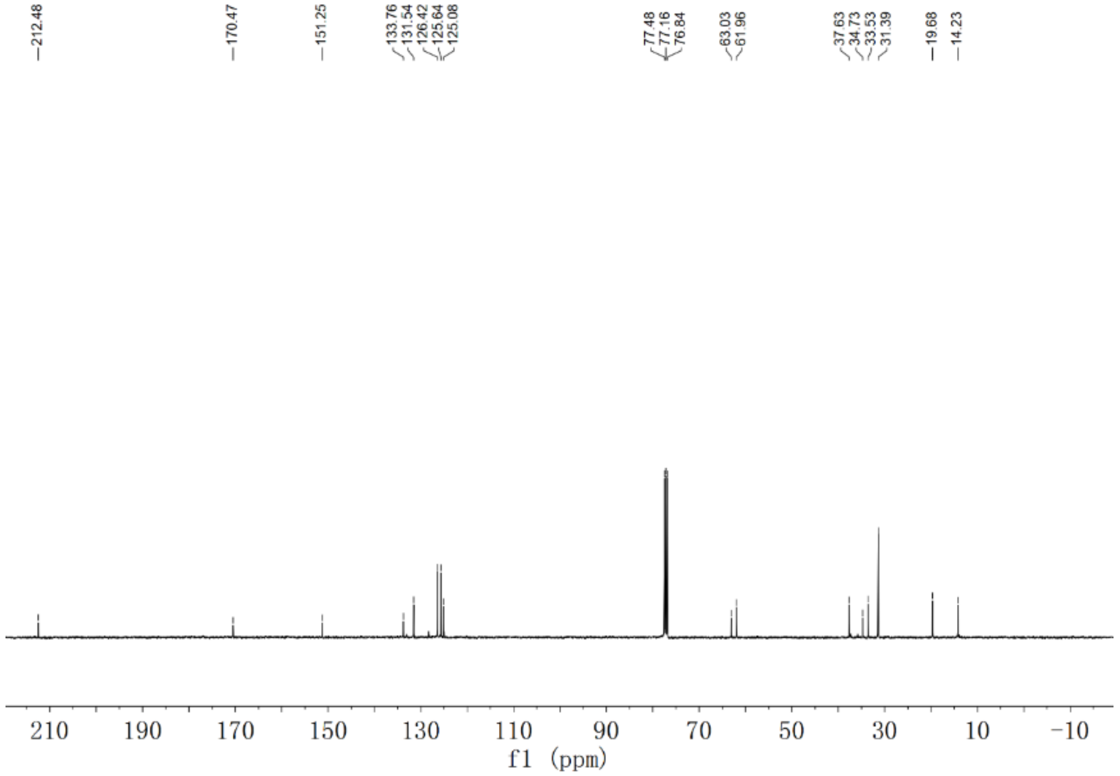

Supplementary Fig. 35. ^{13}C NMR spectra of compound **3c**. (100 MHz, 298K) in $CDCl_3$

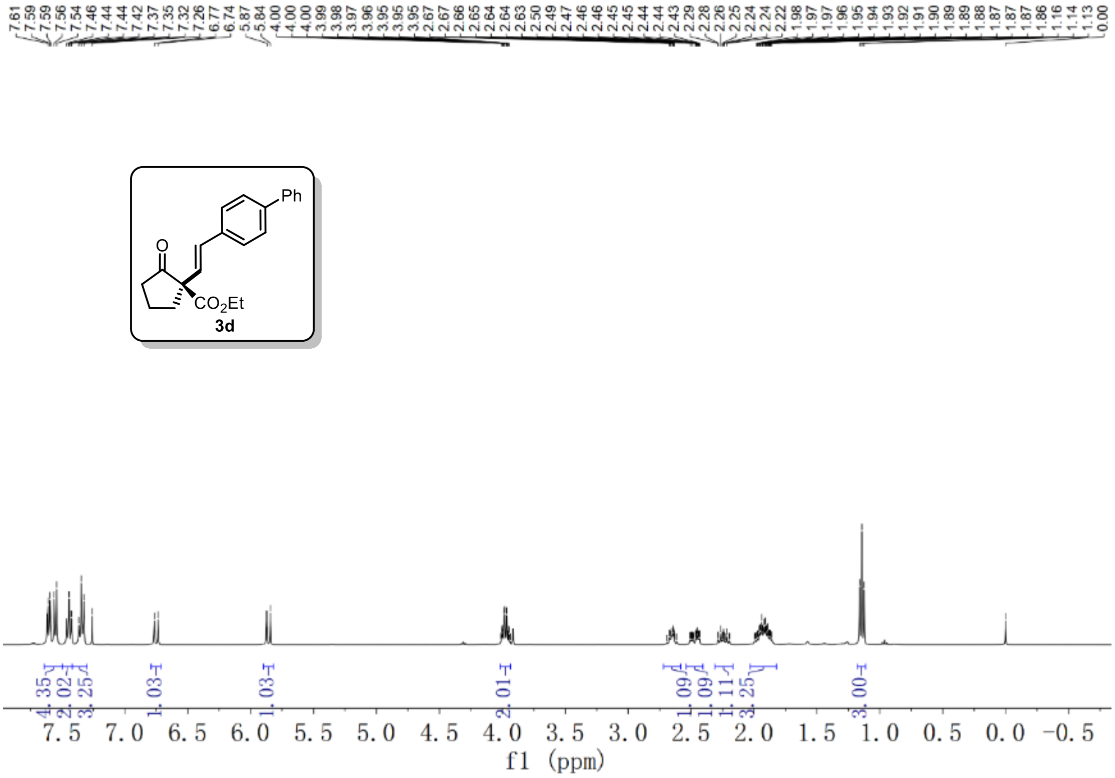

Supplementary Fig. 36. ^{1}H NMR spectra of compound **3d**. (400 MHz, 298K) in $CDCl_3$

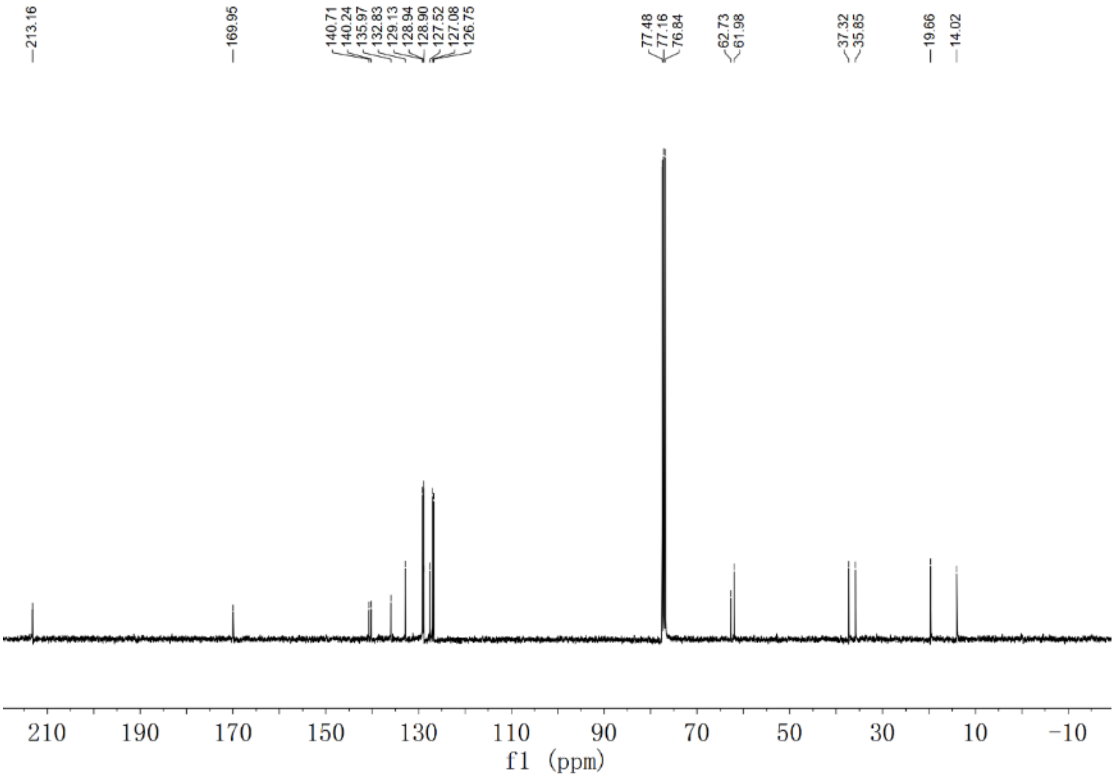

Supplementary Fig. 37. ^{13}C NMR spectra of compound **3d**. (100 MHz, 298K) in $CDCl_3$

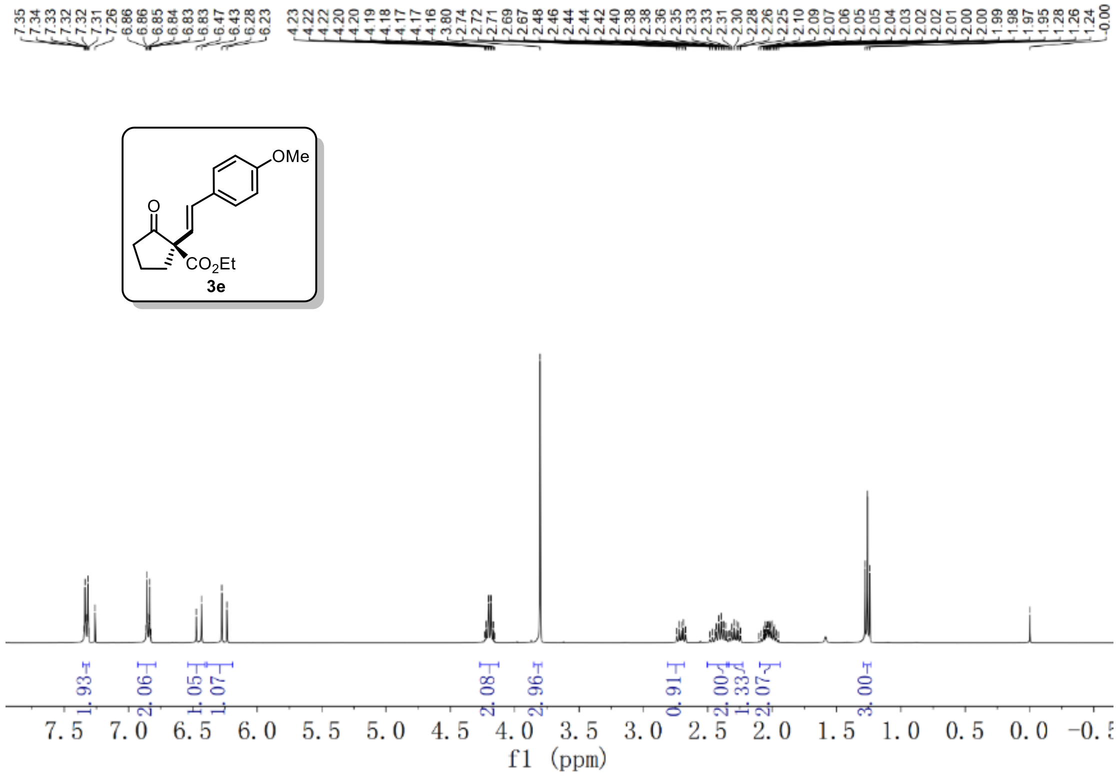

Supplementary Fig. 38. ^{1}H NMR spectra of compound **3e**. (400 MHz, 298K) in $CDCl_3$

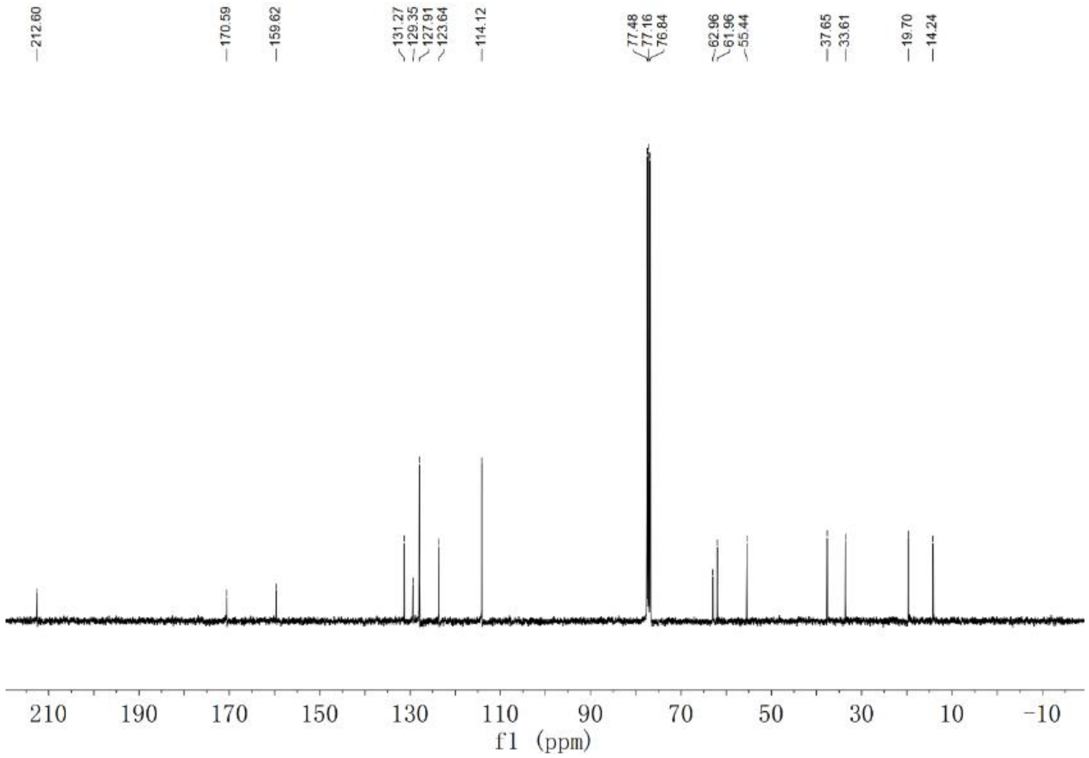

Supplementary Fig. 39. ^{13}C NMR spectra of compound **3e**. (100 MHz, 298K) in $CDCl_3$

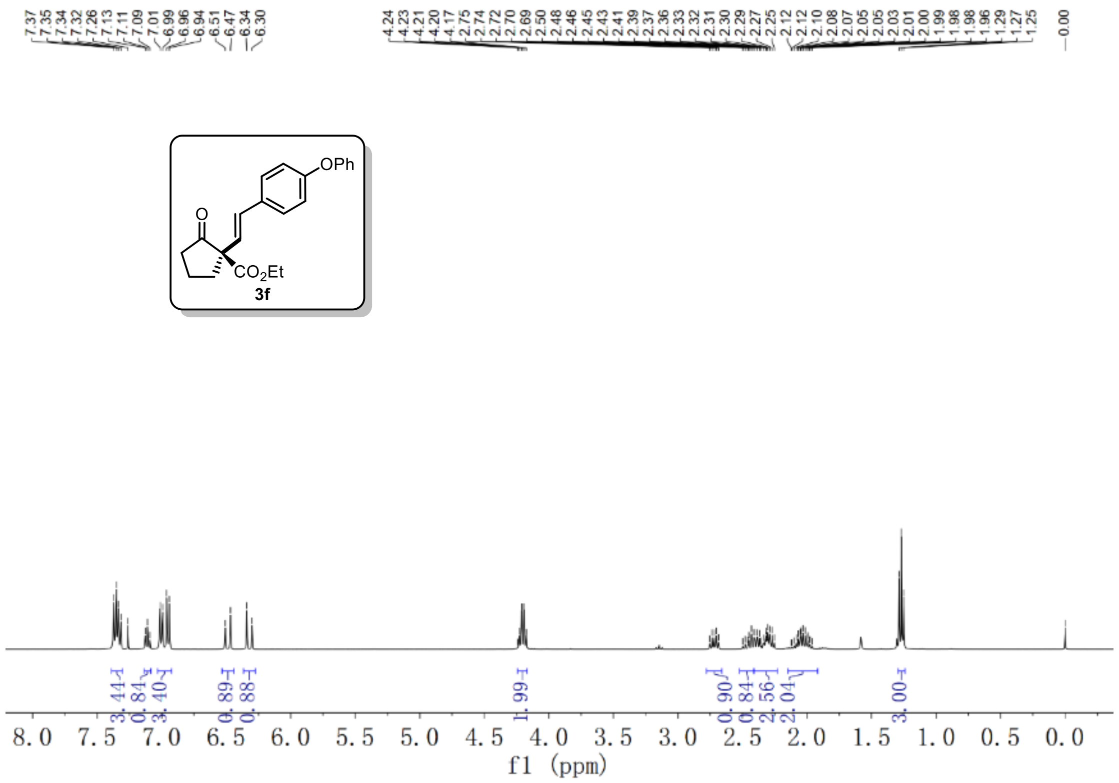

Supplementary Fig. 40. ^{1}H NMR spectra of compound **3f**. (400 MHz, 298K) in $CDCl_3$

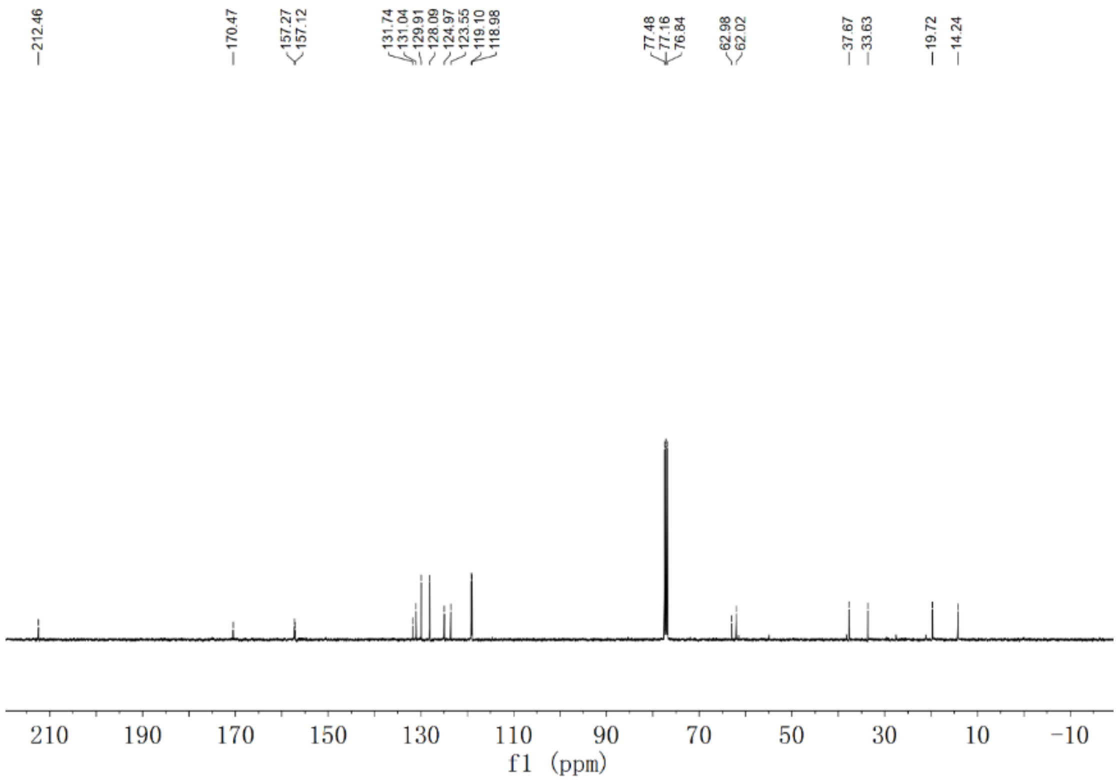

Supplementary Fig. 41. ^{13}C NMR spectra of compound **3f**. (100 MHz, 298K) in $CDCl_3$

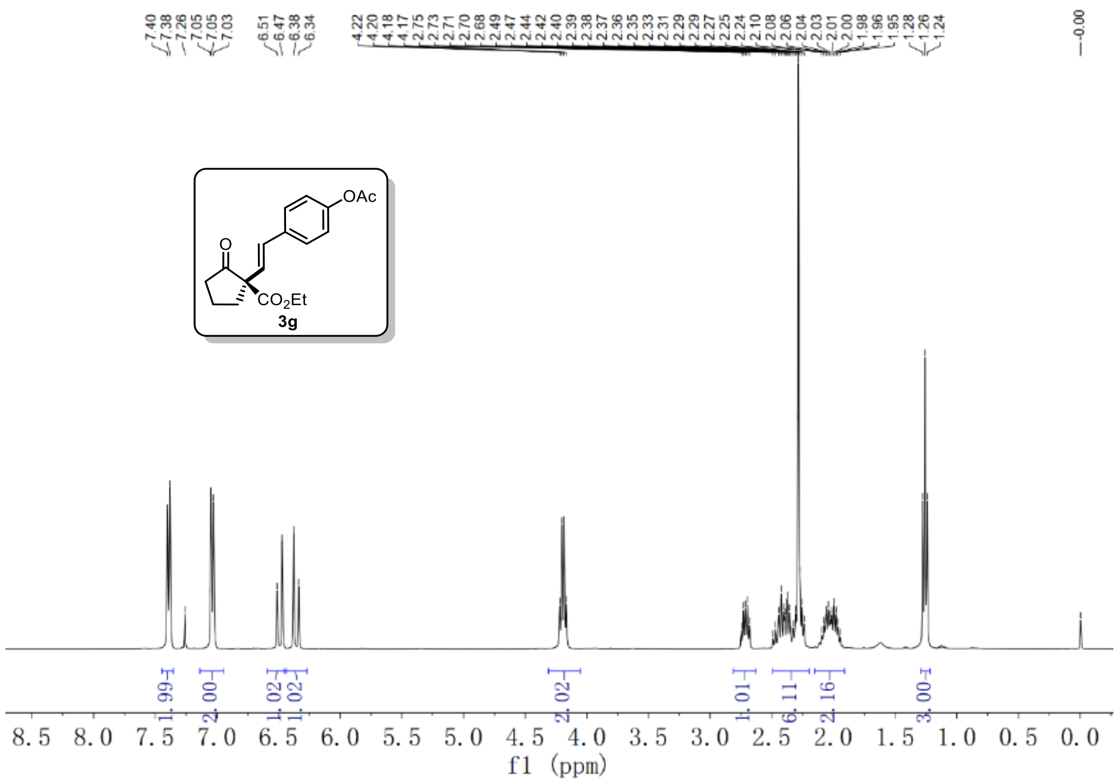

Supplementary Fig. 42. ^{1}H NMR spectra of compound **3g**. (400 MHz, 298K) in $CDCl_3$

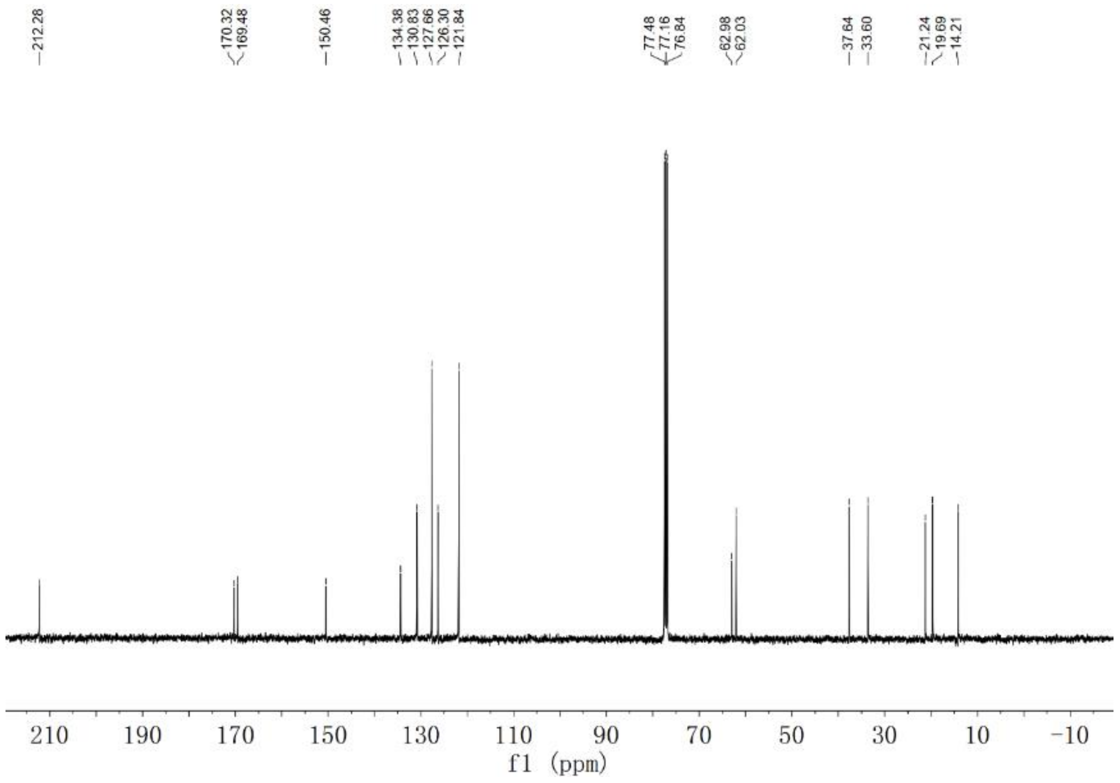

Supplementary Fig. 43. ^{13}C NMR spectra of compound **3g**. (100 MHz, 298K) in $CDCl_3$

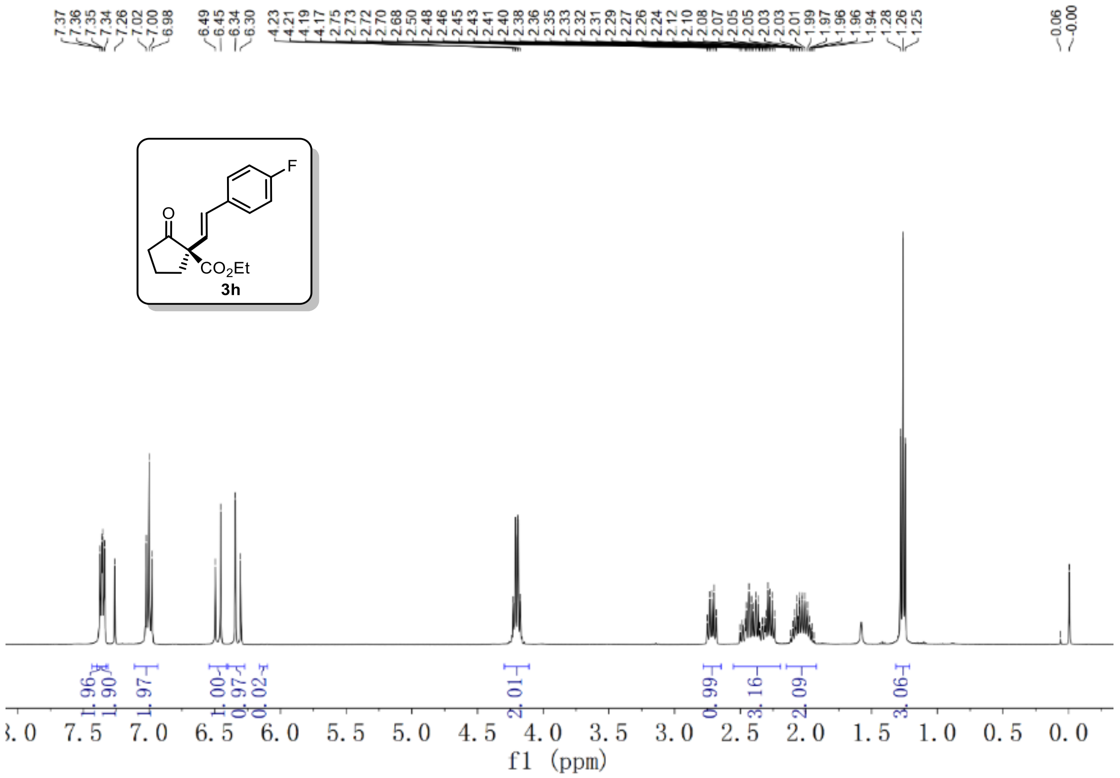

Supplementary Fig. 44. ^{1}H NMR spectra of compound **3h**. (400 MHz, 298K) in $CDCl_3$

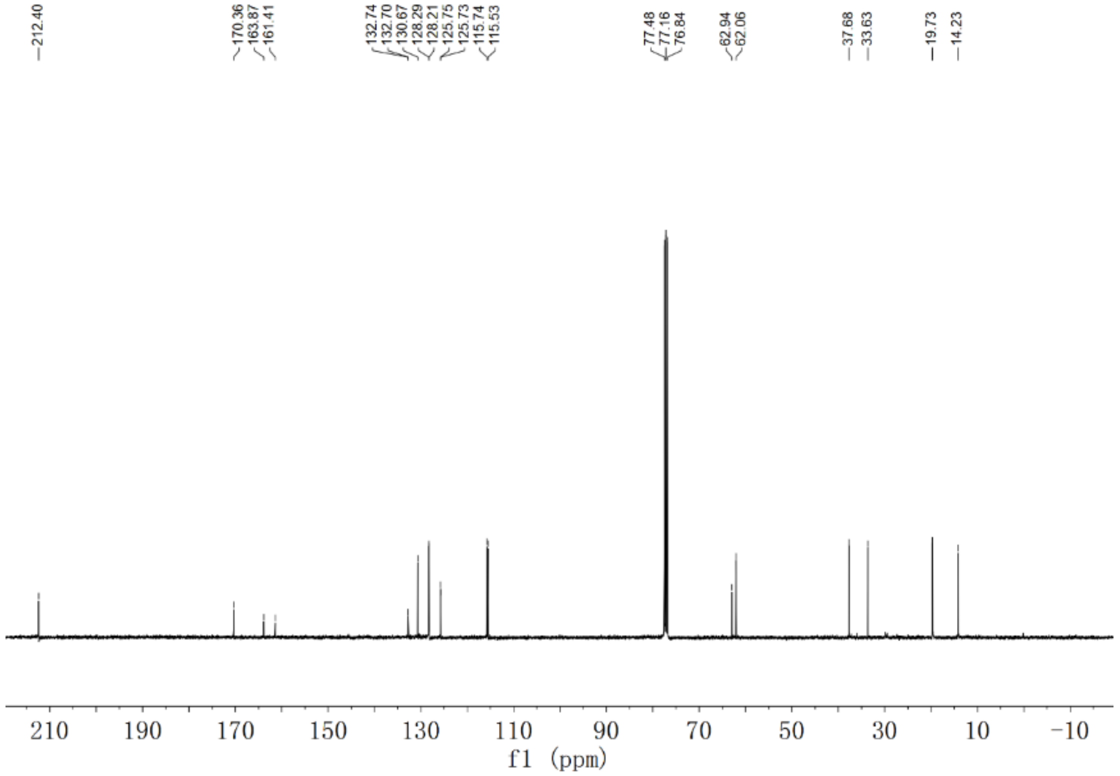

Supplementary Fig. 45. ^{13}C NMR spectra of compound **3h**. (100 MHz, 298K) in $CDCl_3$

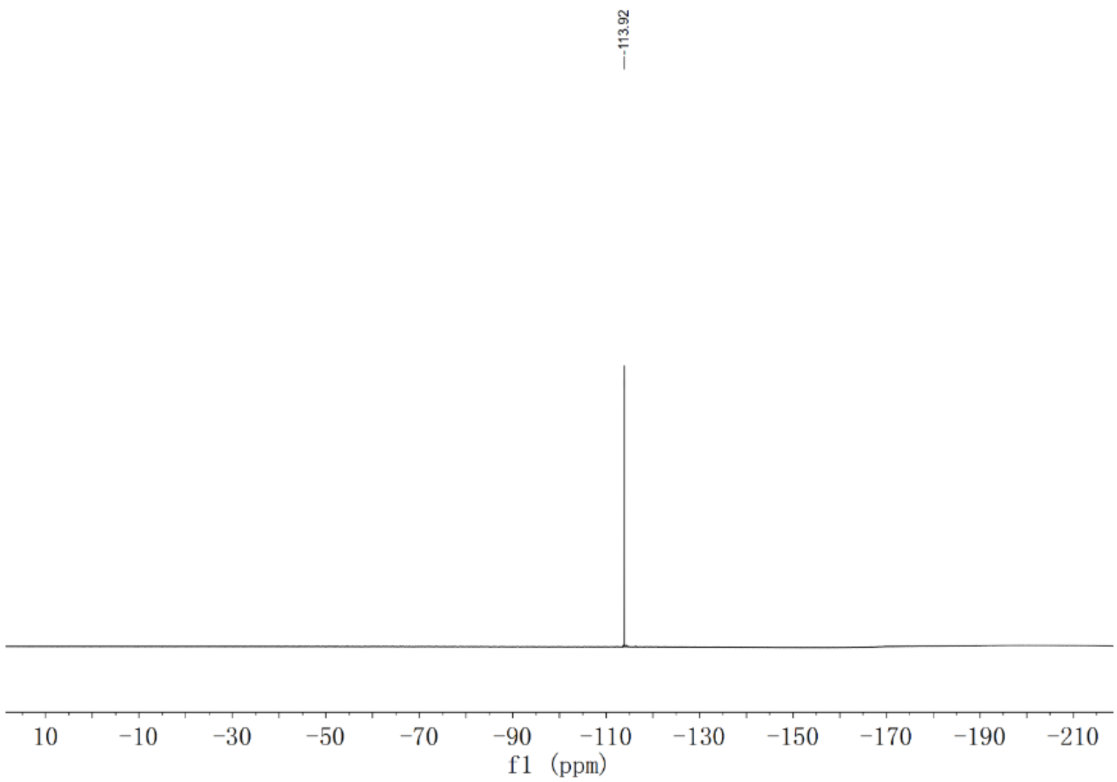

Supplementary Fig. 46. ^{19}F NMR spectra of compound **3h**. (376 MHz, 298K) in $CDCl_3$

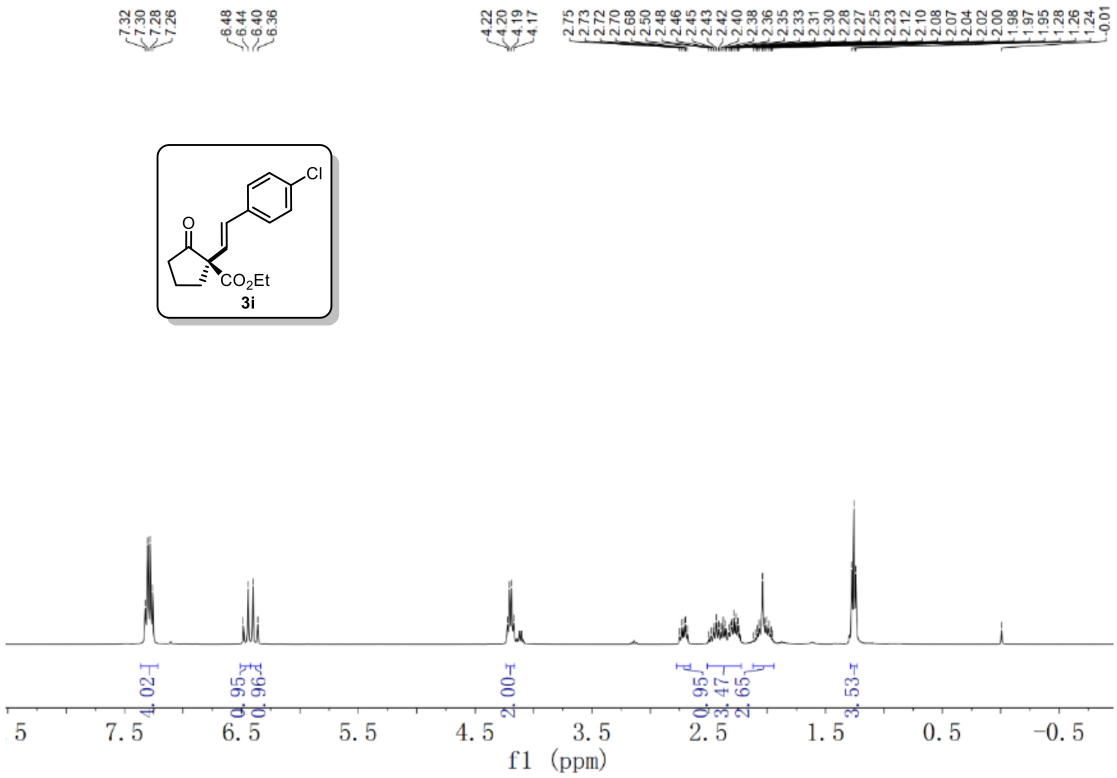

Supplementary Fig. 47. ^{1}H NMR spectra of compound **3i**. (400 MHz, 298K) in $CDCl_3$

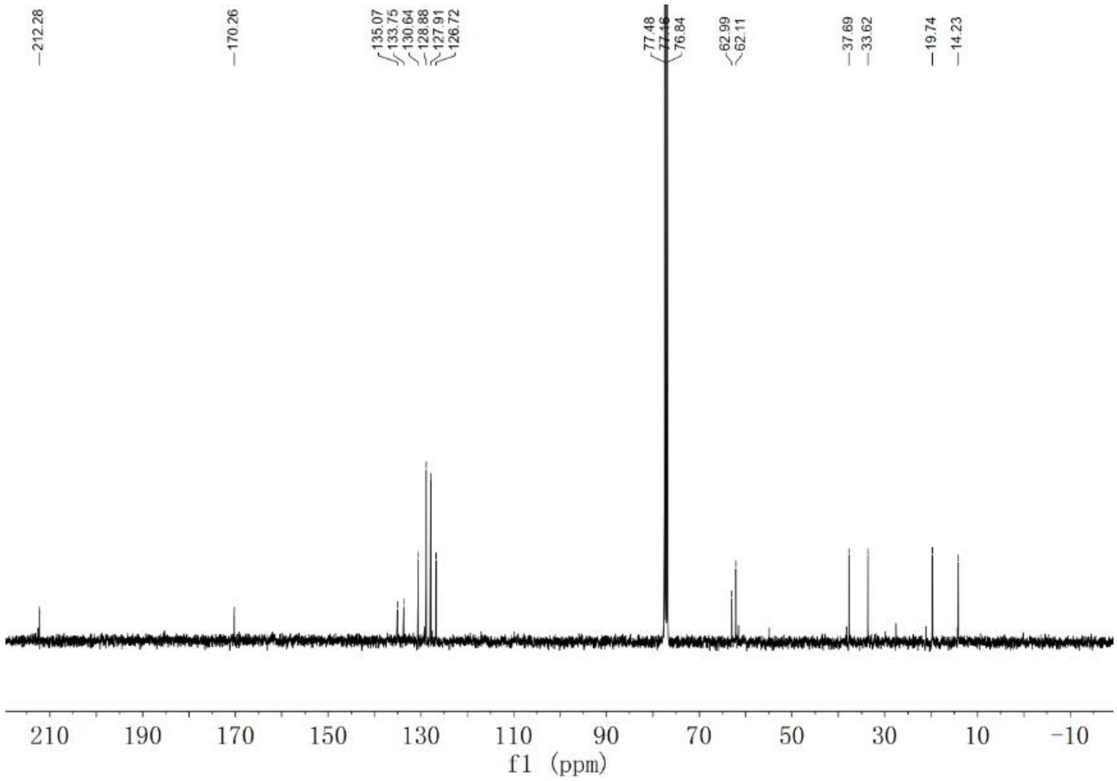

Supplementary Fig. 48. ^{13}C NMR spectra of compound **3i**. (100 MHz, 298K) in $CDCl_3$

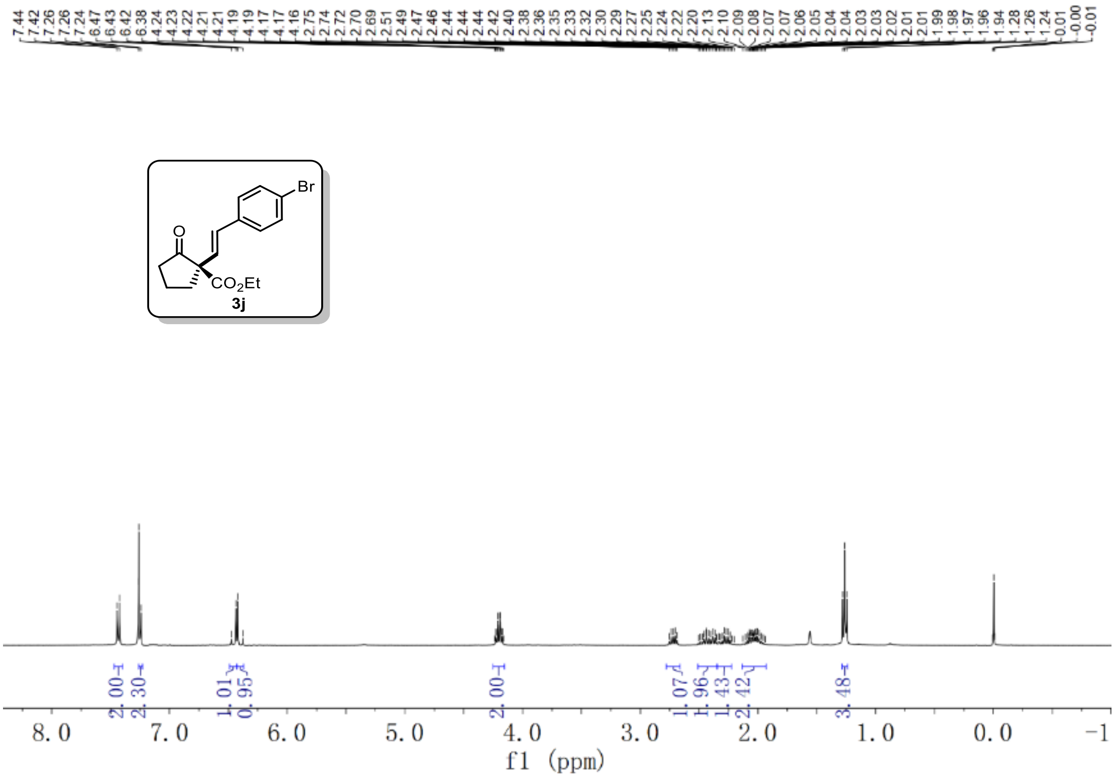

Supplementary Fig. 49. ^{1}H NMR spectra of compound **3j**. (400 MHz, 298K) in $CDCl_3$

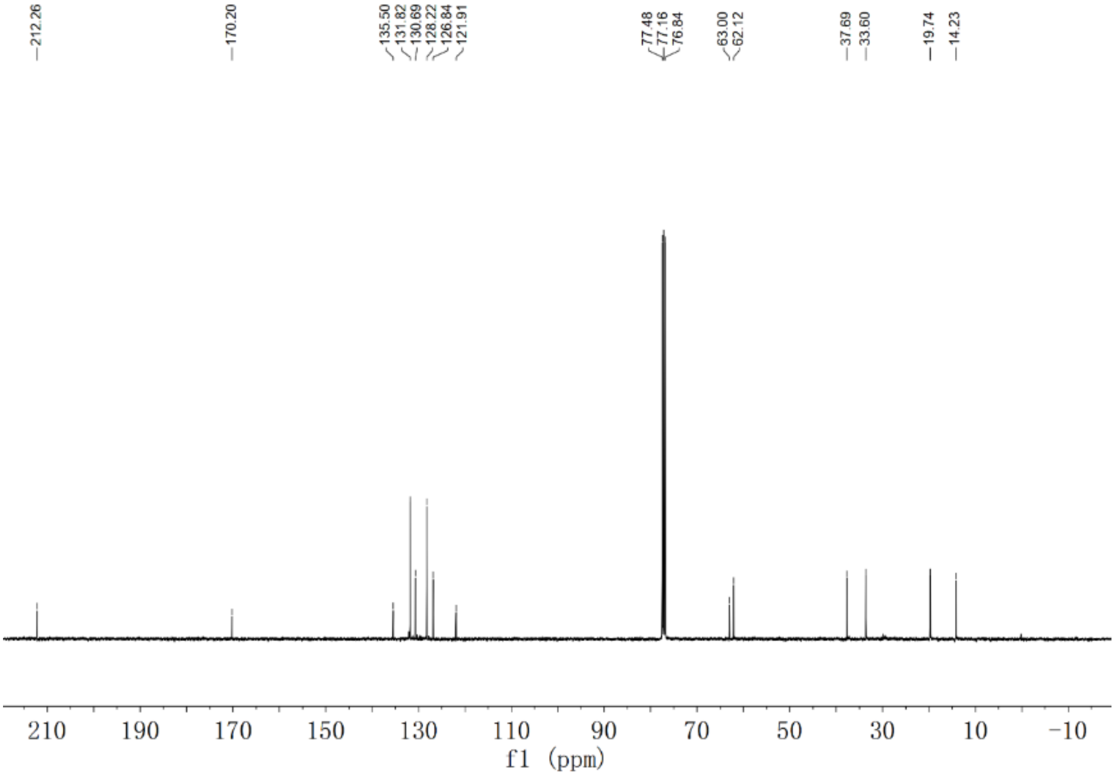

Supplementary Fig. 50. ^{13}C NMR spectra of compound **3j**. (100 MHz, 298K) in $CDCl_3$

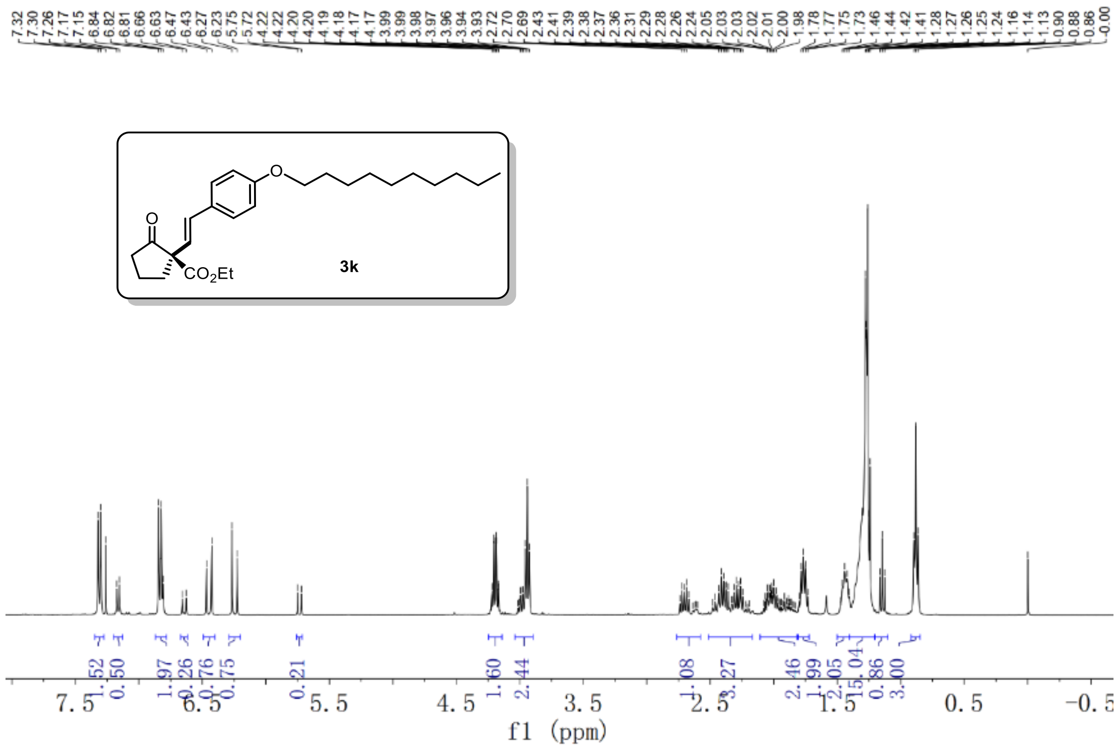

Supplementary Fig. 51. ^{1}H NMR spectra of compound **3k**. (400 MHz, 298K) in $CDCl_3$

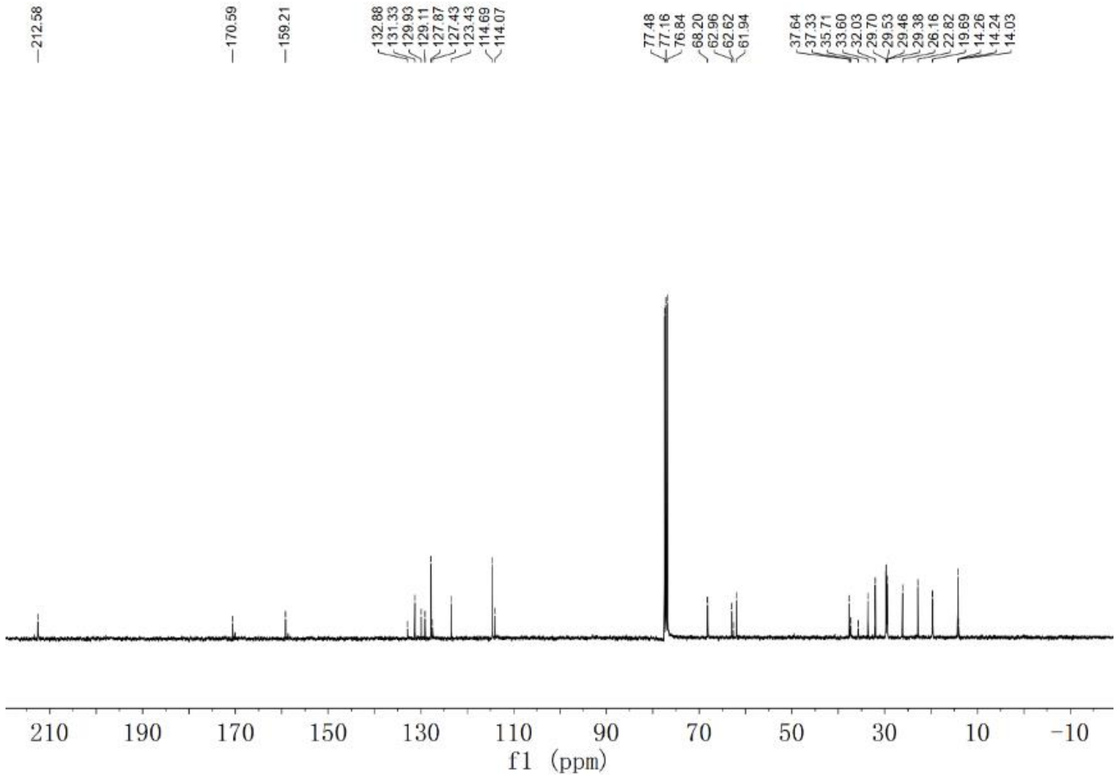

Supplementary Fig.52. ^{13}C NMR spectra of compound **3k**. (100 MHz, 298K) in $CDCl_3$

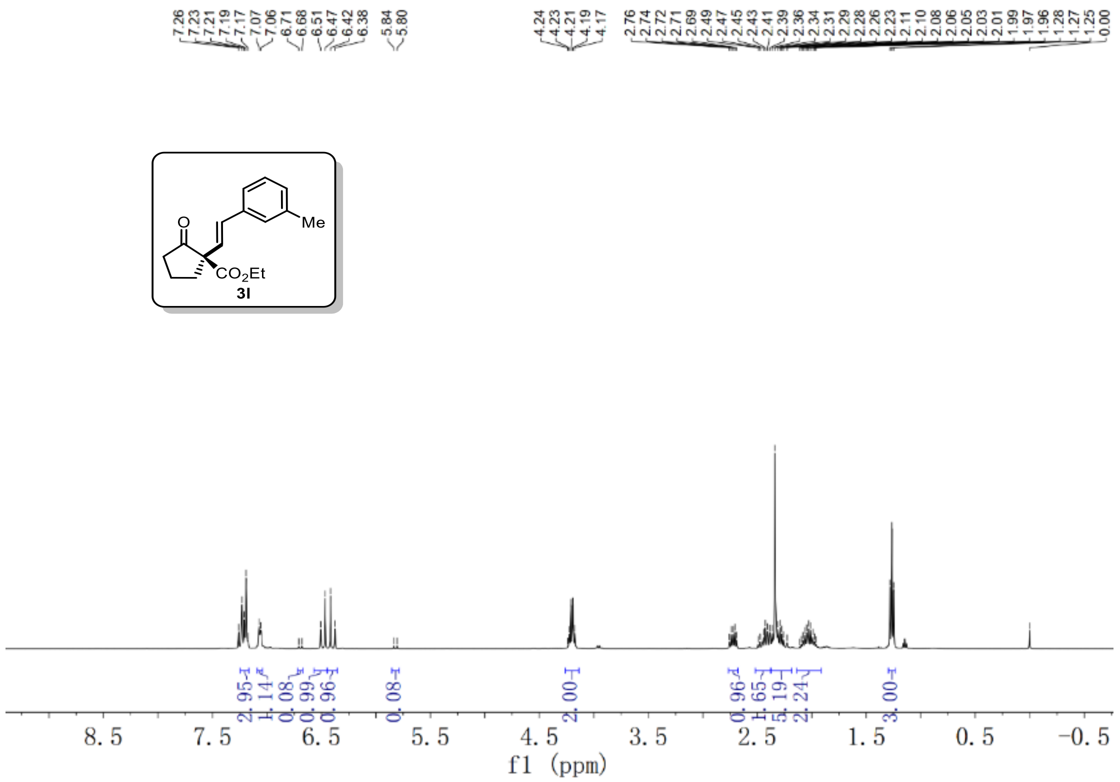

Supplementary Fig. 53. ^{1}H NMR spectra of compound **3l**. (400 MHz, 298K) in $CDCl_3$

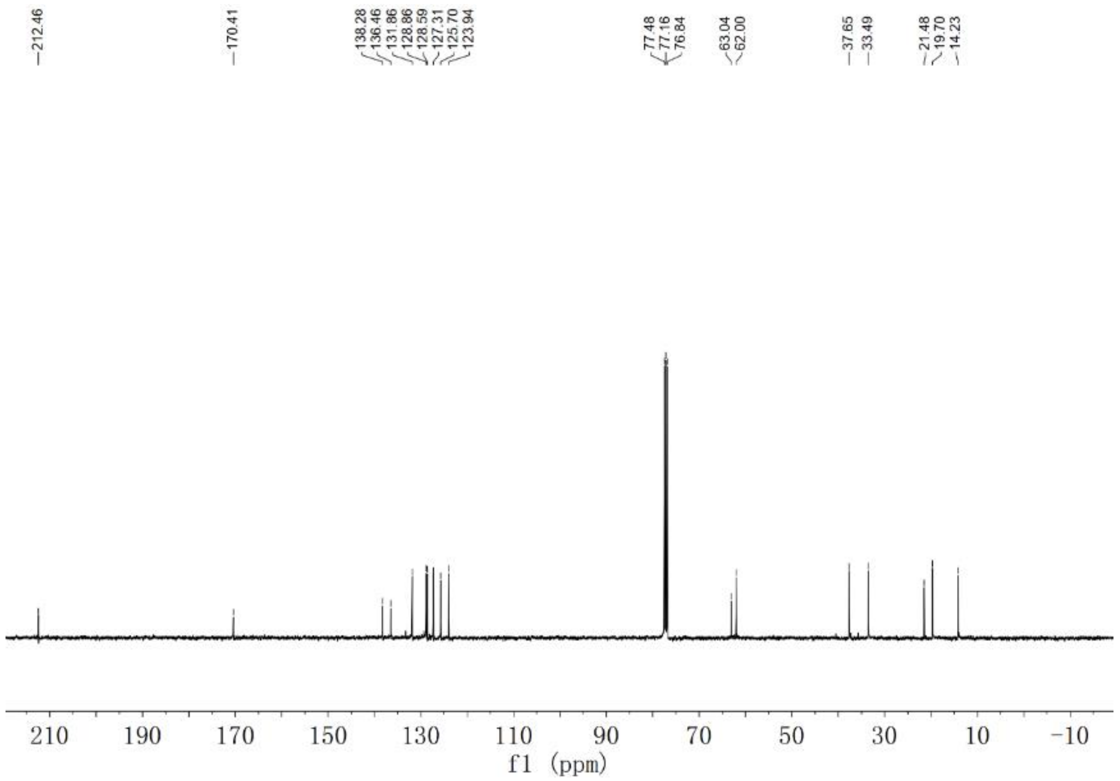

Supplementary Fig. 54. ^{13}C NMR spectra of compound **3l**. (100 MHz, 298K) in $CDCl_3$

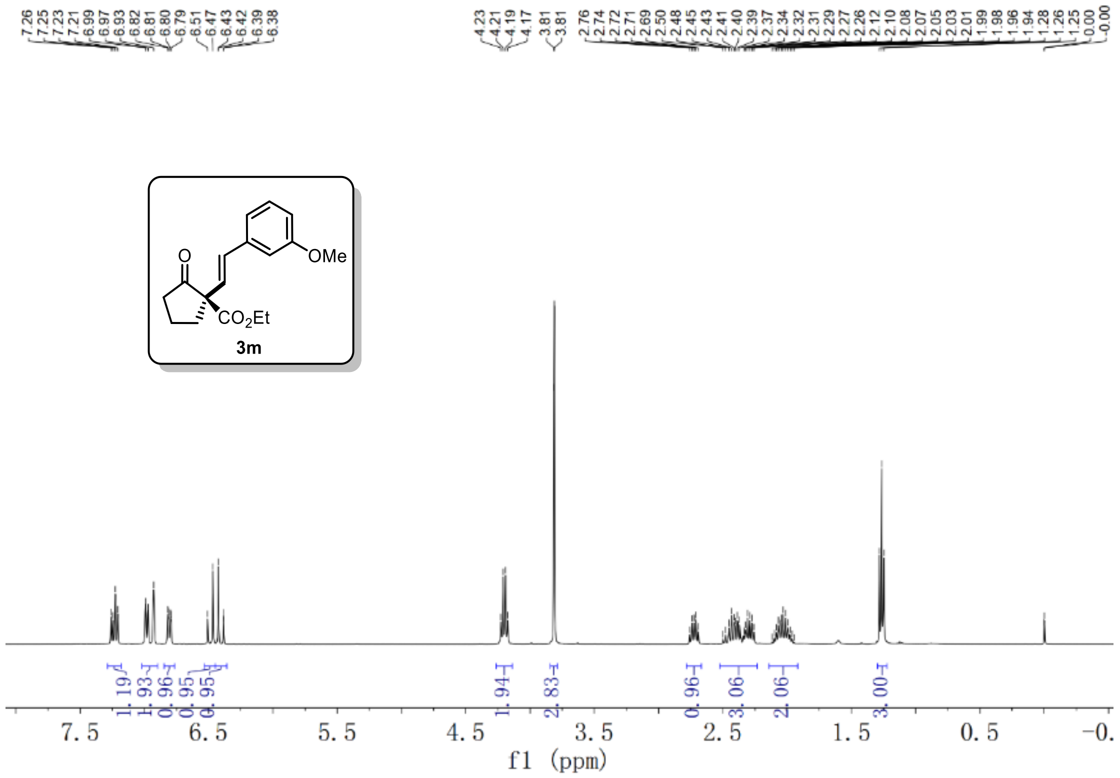

Supplementary Fig. 55. ^{1}H NMR spectra of compound **3m**. (400 MHz, 298K) in $CDCl_3$

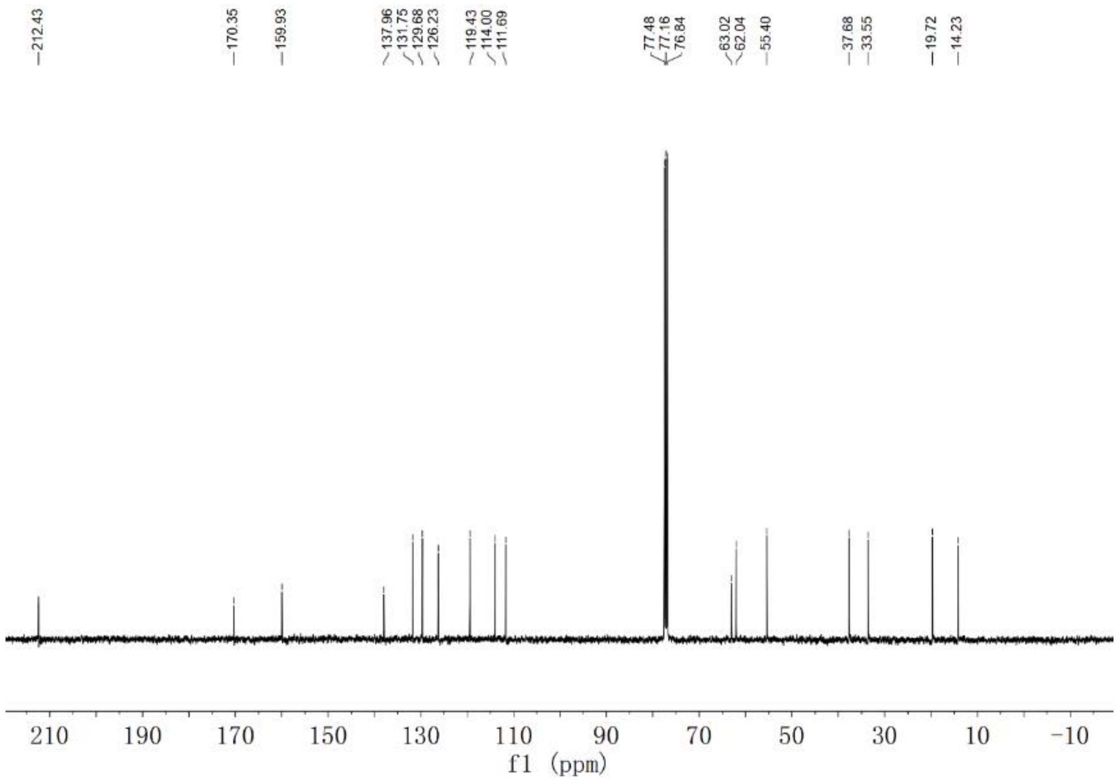

Supplementary Fig. 56. ^{13}C NMR spectra of compound **3m**. (100 MHz, 298K) in $CDCl_3$

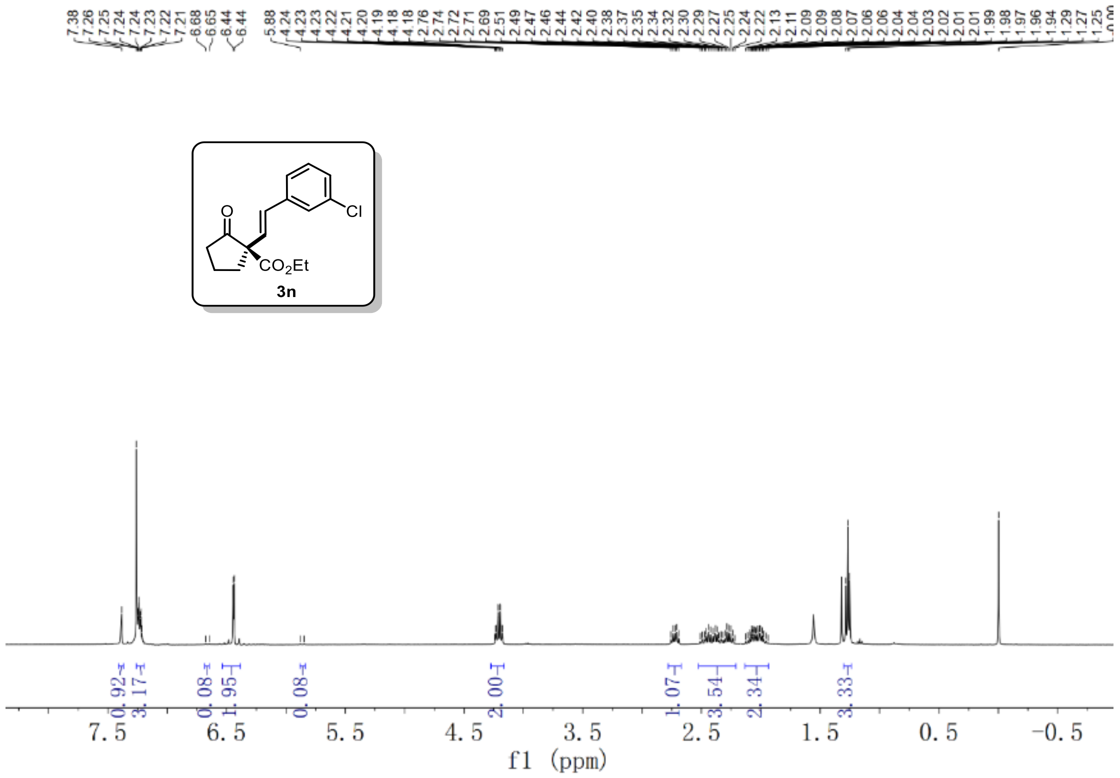

Supplementary Fig. 57. ^{1}H NMR spectra of compound **3n**. (400 MHz, 298K) in $CDCl_3$

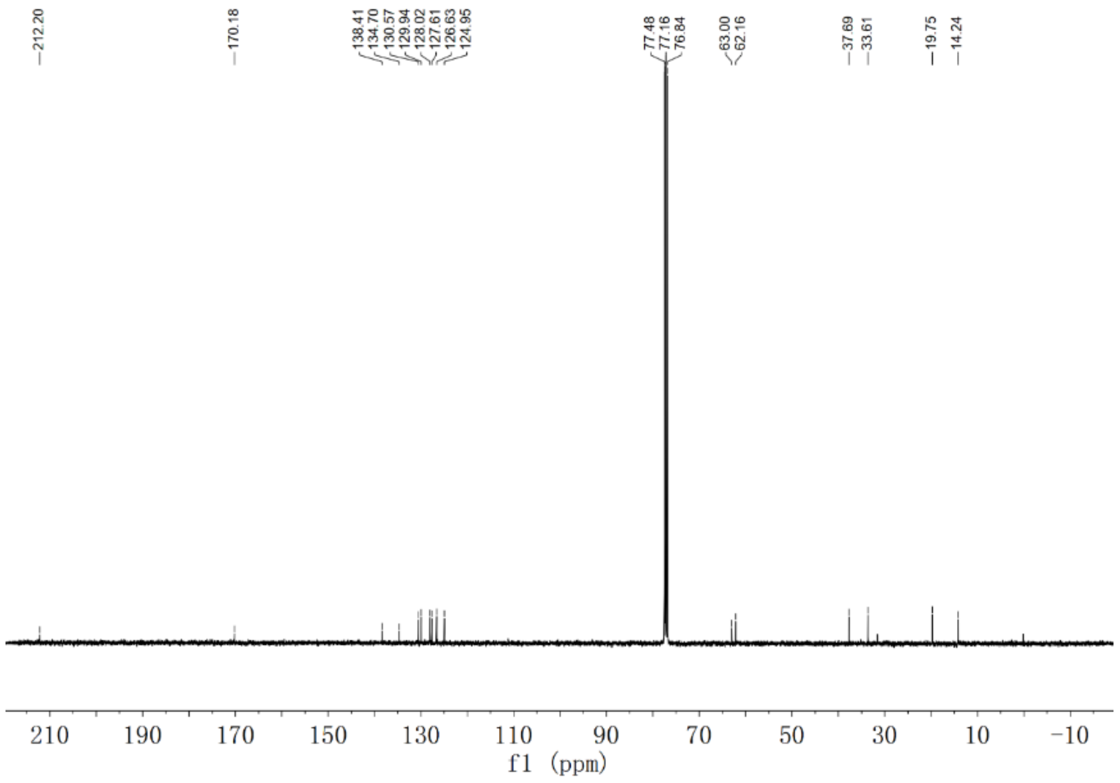

Supplementary Fig. 58. ^{13}C NMR spectra of compound **3n**. (100 MHz, 298K) in $CDCl_3$

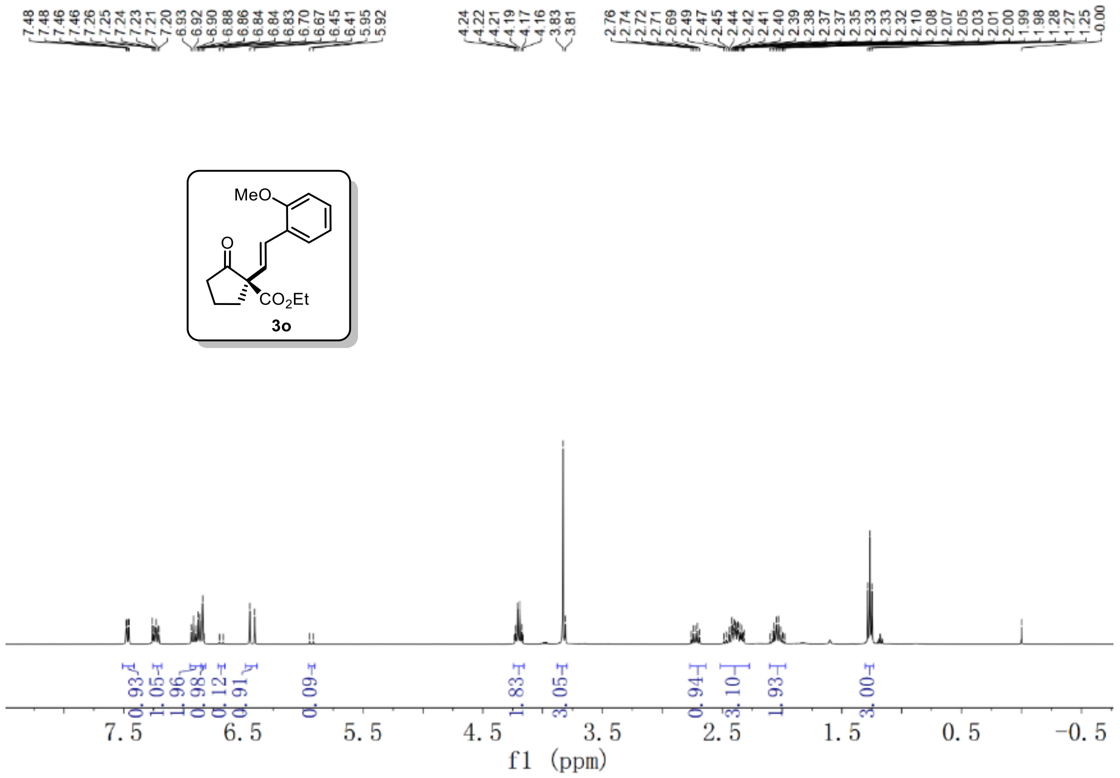

Supplementary Fig. 59. ^{1}H NMR spectra of compound **3o**. (400 MHz, 298K) in $CDCl_3$

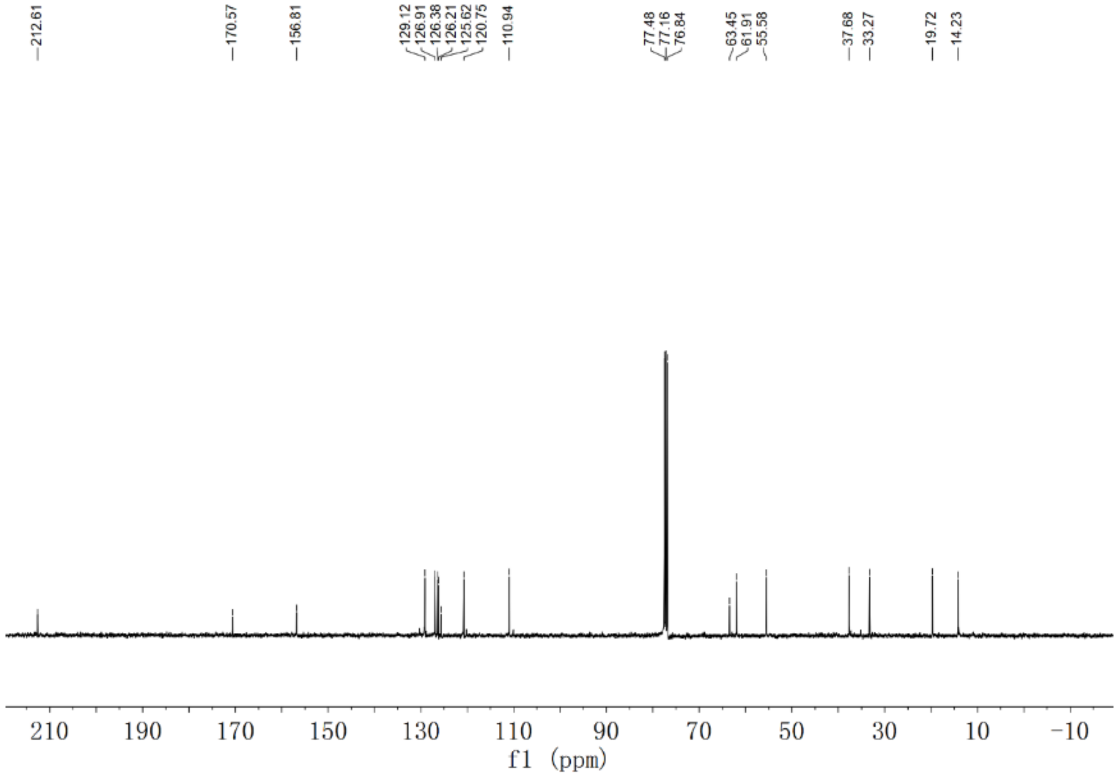

Supplementary Fig. 60. ^{13}C NMR spectra of compound **3o**. (100 MHz, 298K) in $CDCl_3$

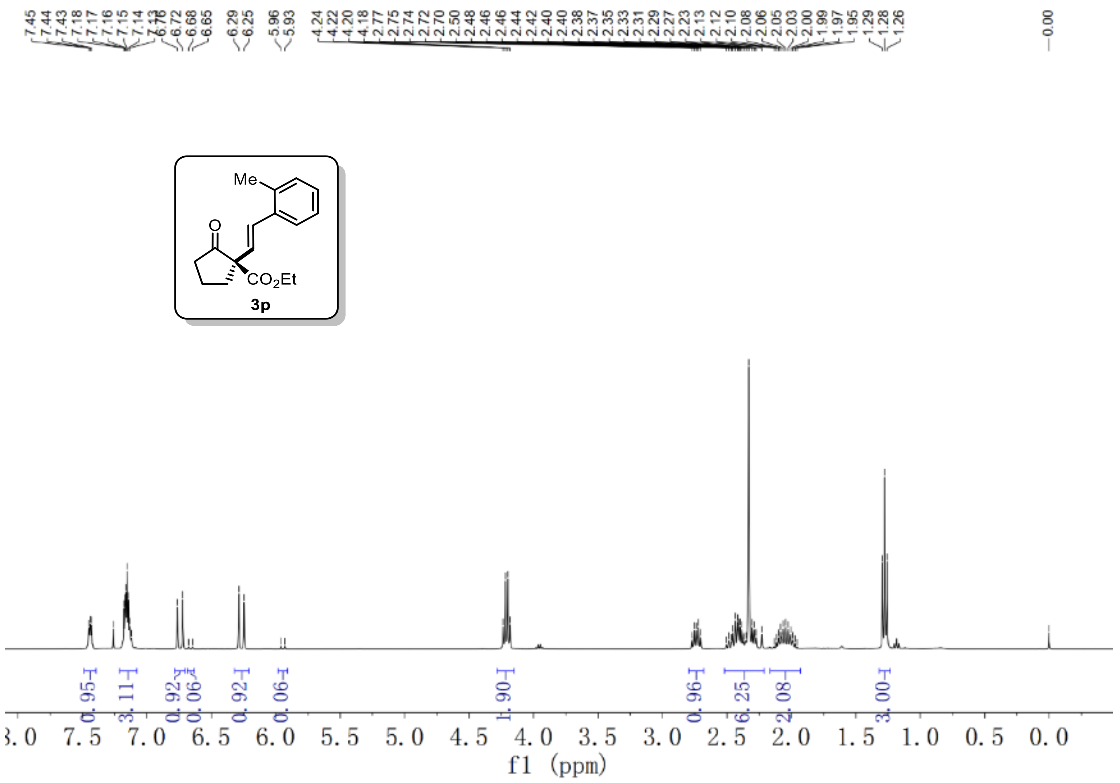

Supplementary Fig. 61. ^{1}H NMR spectra of compound **3p**. (400 MHz, 298K) in $CDCl_3$

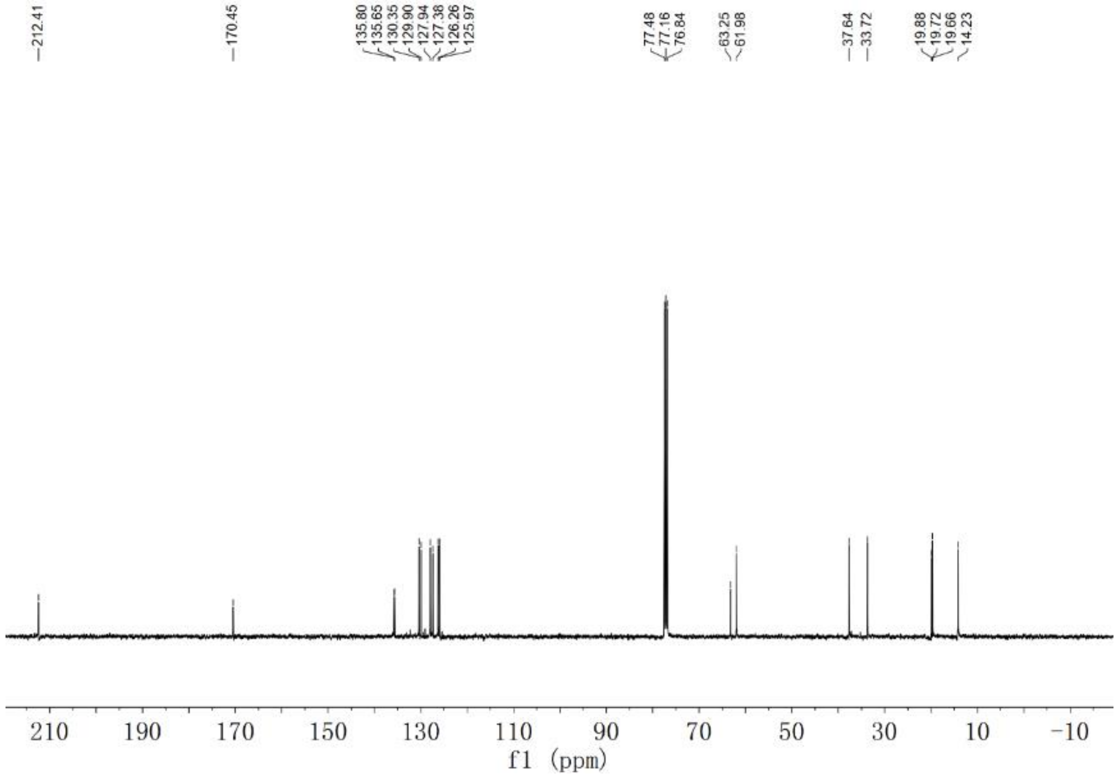

Supplementary Fig. 62. ^{13}C NMR spectra of compound **3p**. (100 MHz, 298K) in $CDCl_3$

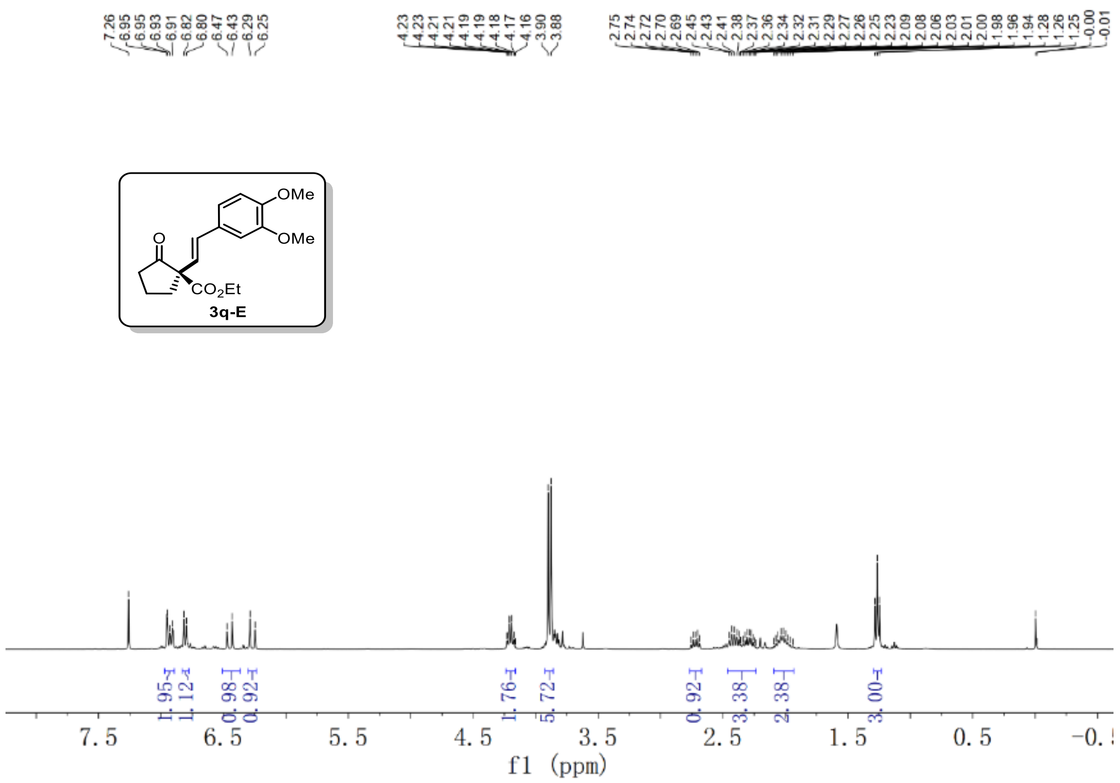

Supplementary Fig. 63. ^{1}H NMR spectra of compound **3q-*E***. (400 MHz, 298K) in $CDCl_3$

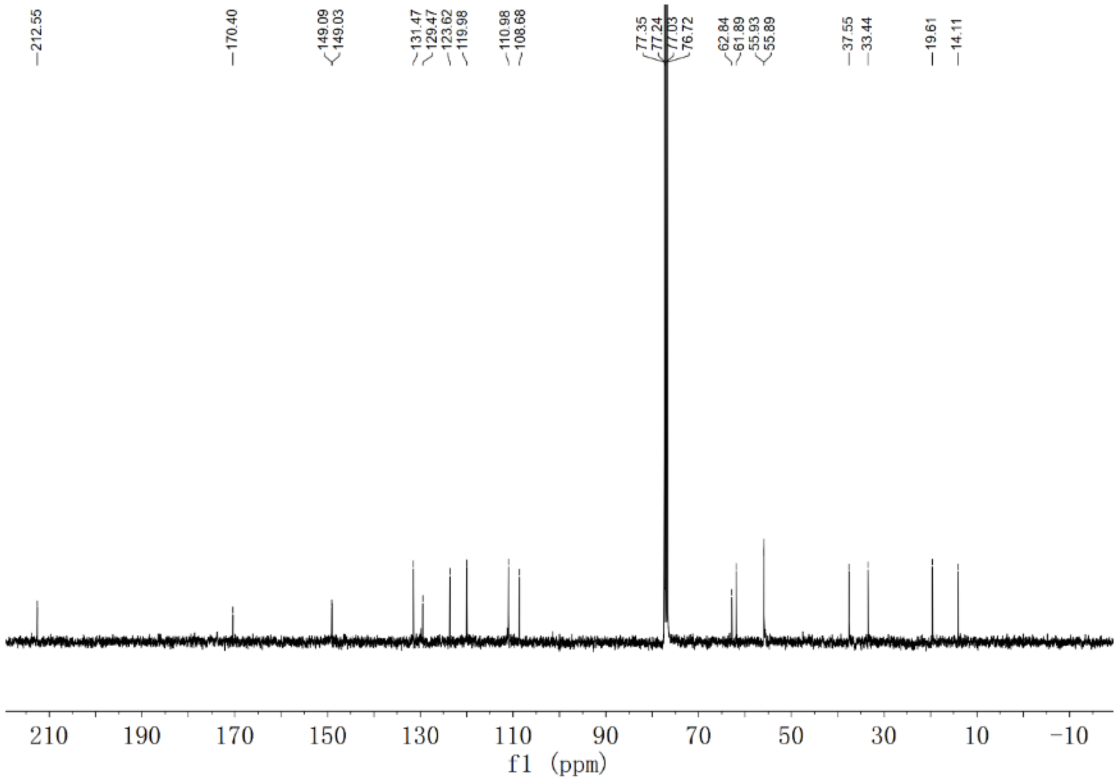

Supplementary Fig. 64. ^{13}C NMR spectra of compound **3q-*E***. (100 MHz, 298K) in $CDCl_3$

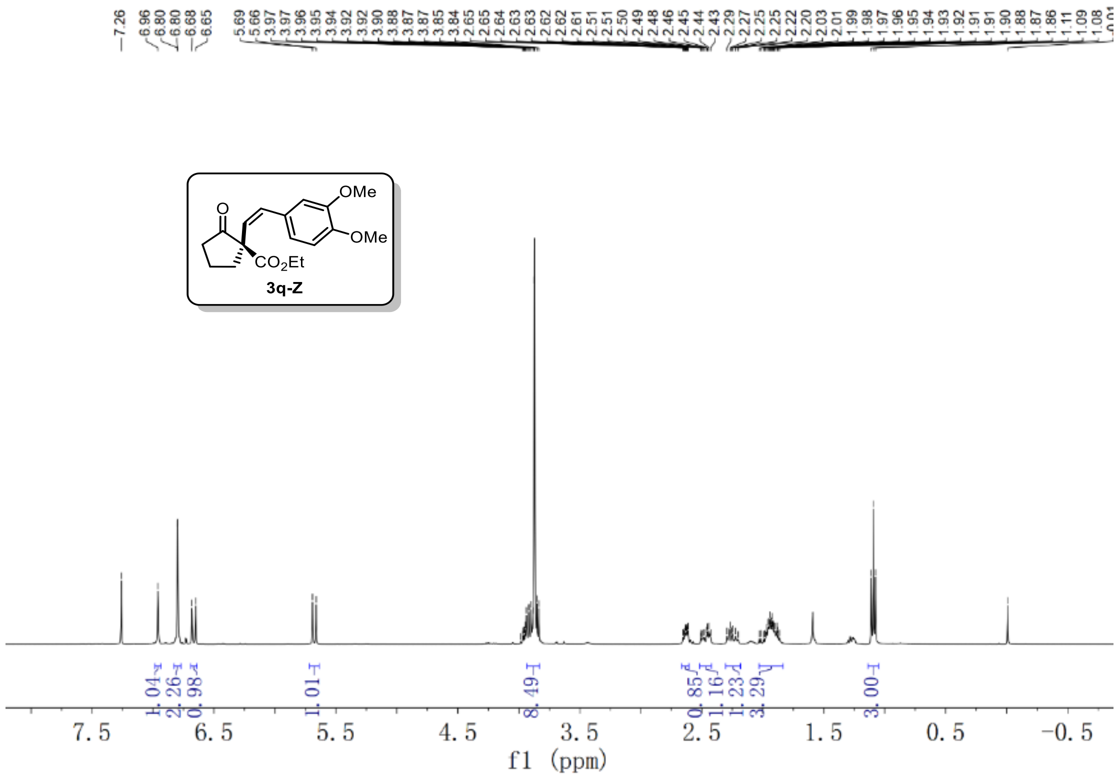

Supplementary Fig. 65. ^{1}H NMR spectra of compound **3q-*Z***. (400 MHz, 298K) in $CDCl_3$

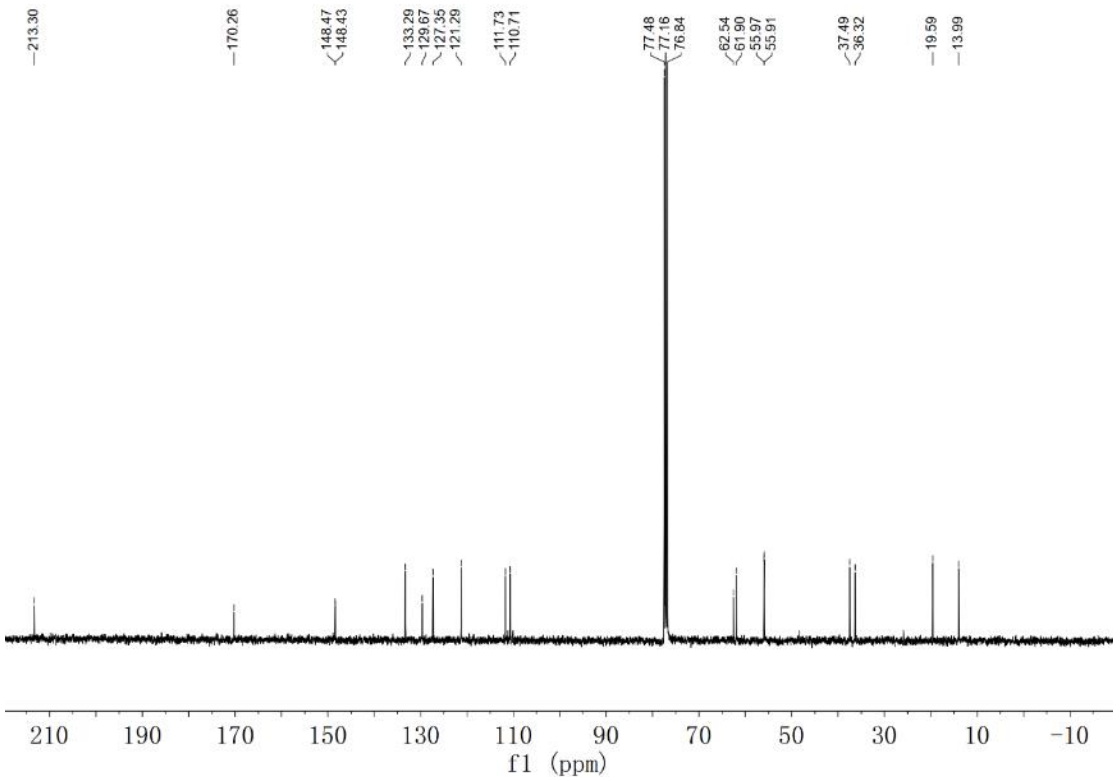

Supplementary Fig. 66. ^{13}C NMR spectra of compound **3q-*Z***. (100 MHz, 298K) in $CDCl_3$

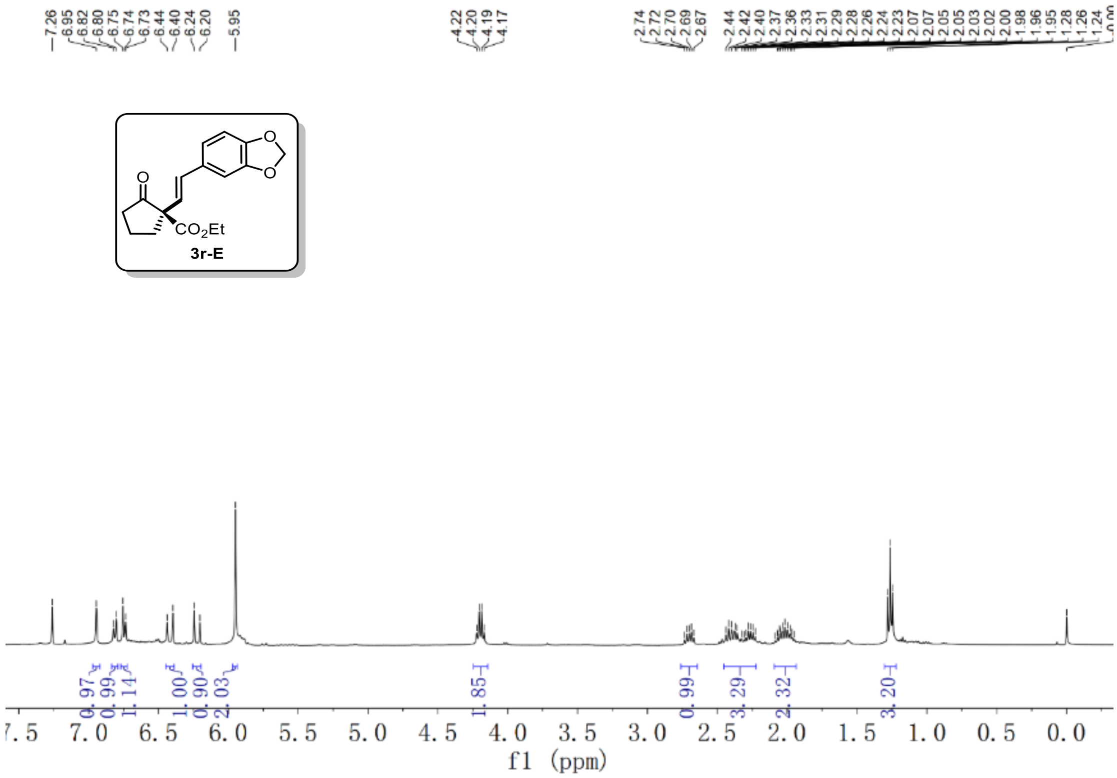

Supplementary Fig. 67. ^{1}H NMR spectra of compound **3r-*E***. (400 MHz, 298K) in $CDCl_3$

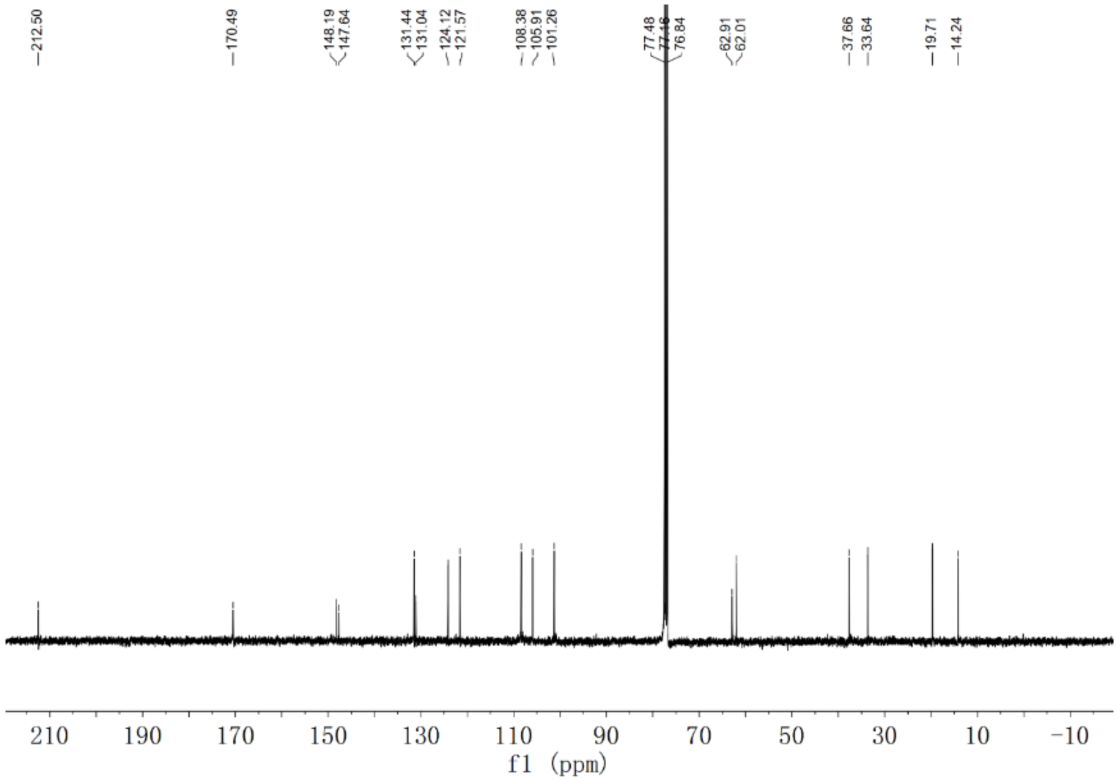

Supplementary Fig. 68. ^{13}C NMR spectra of compound **3r-*E***. (100 MHz, 298K) in $CDCl_3$

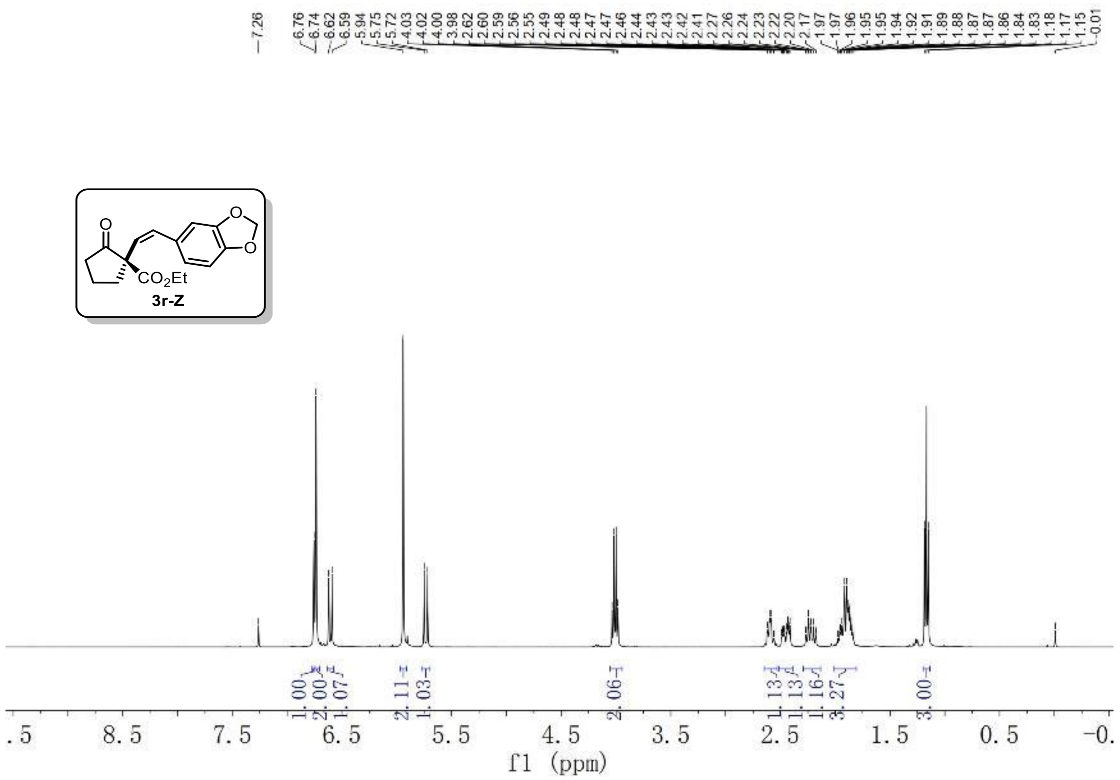

Supplementary Fig. 69. ^{1}H NMR spectra of compound **3r-*Z***. (400 MHz, 298K) in $CDCl_3$

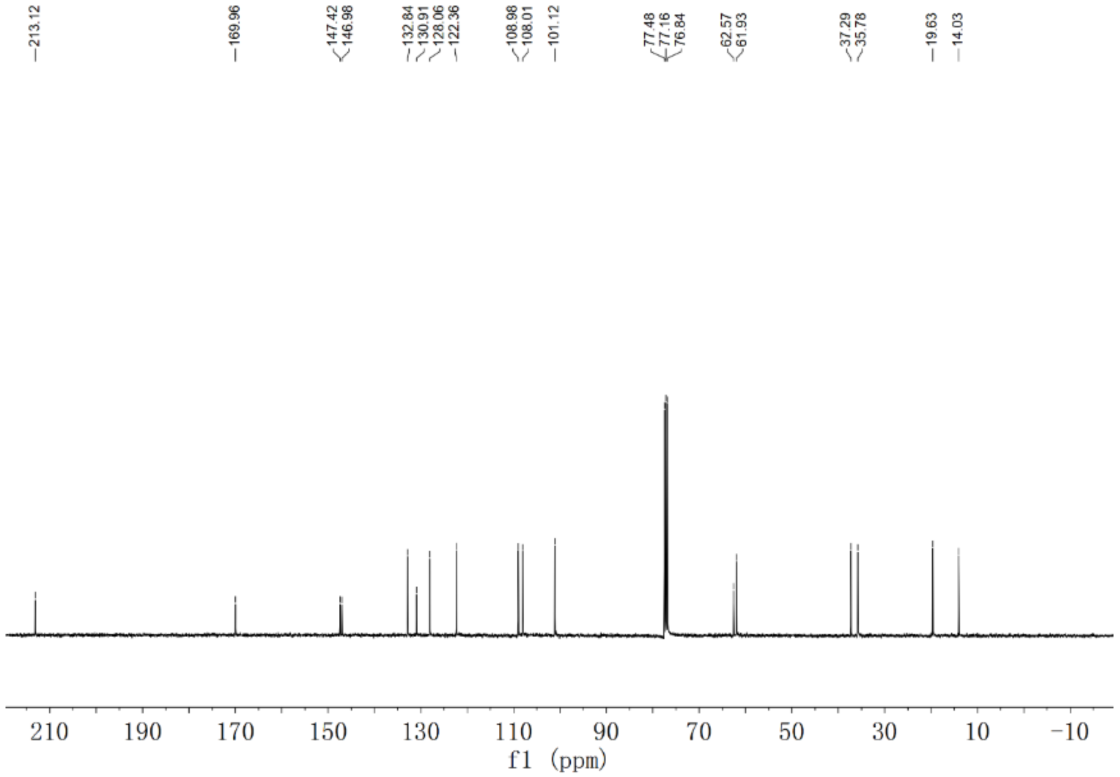

Supplementary Fig. 70. ^{13}C NMR spectra of compound **3r-*Z***. (100 MHz, 298K) in $CDCl_3$

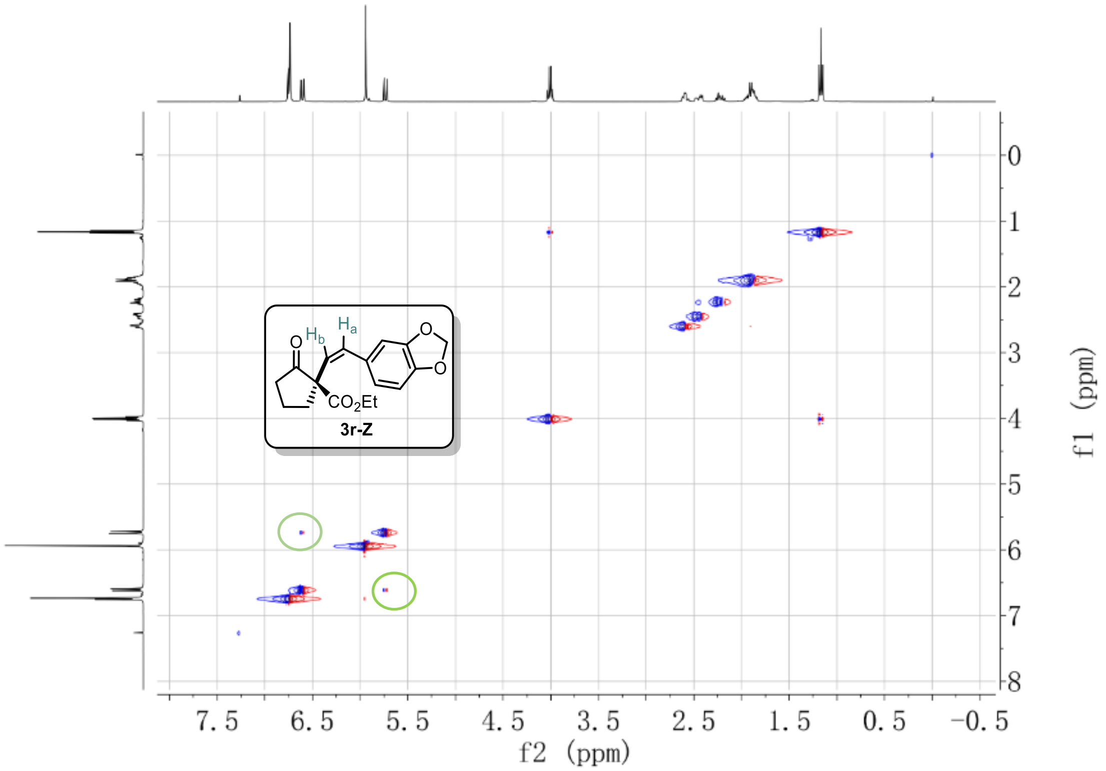

Supplementary Fig. 71. 2D NOE ^{1}H NMR spectra of compound **3r-*Z***. (400 MHz, 298K) in $CDCl_3$

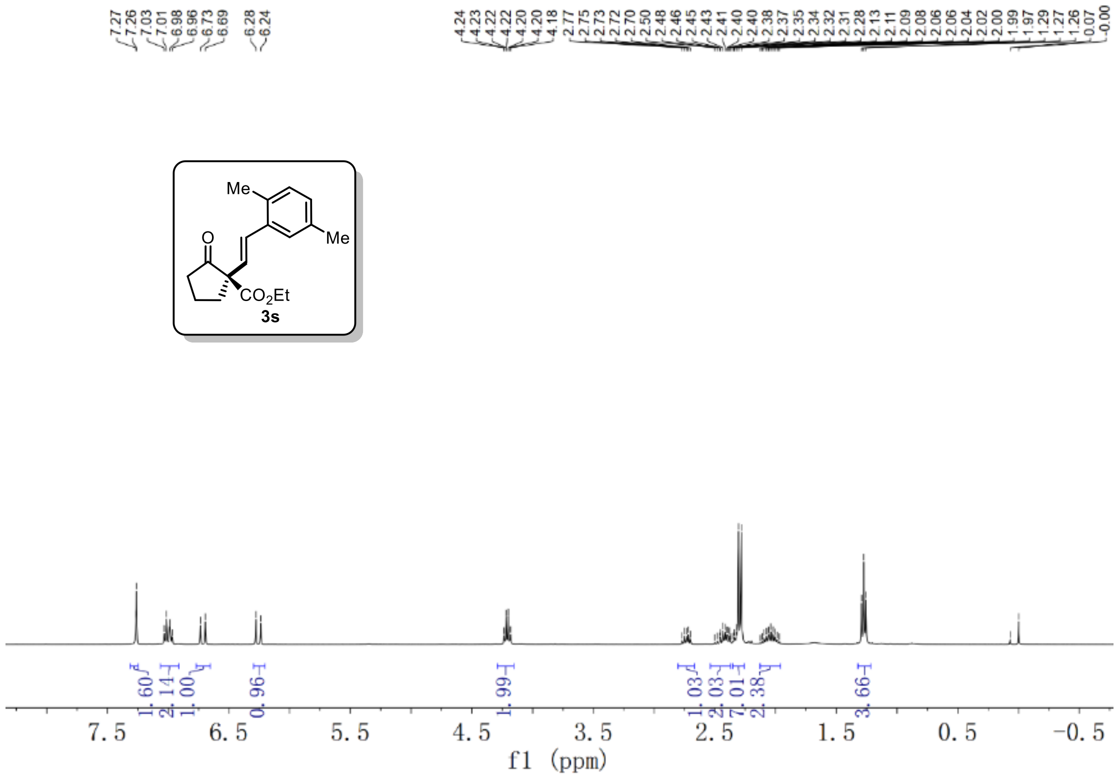

Supplementary Fig. 72. ^{1}H NMR spectra of compound **3s**. (400 MHz, 298K) in $CDCl_3$

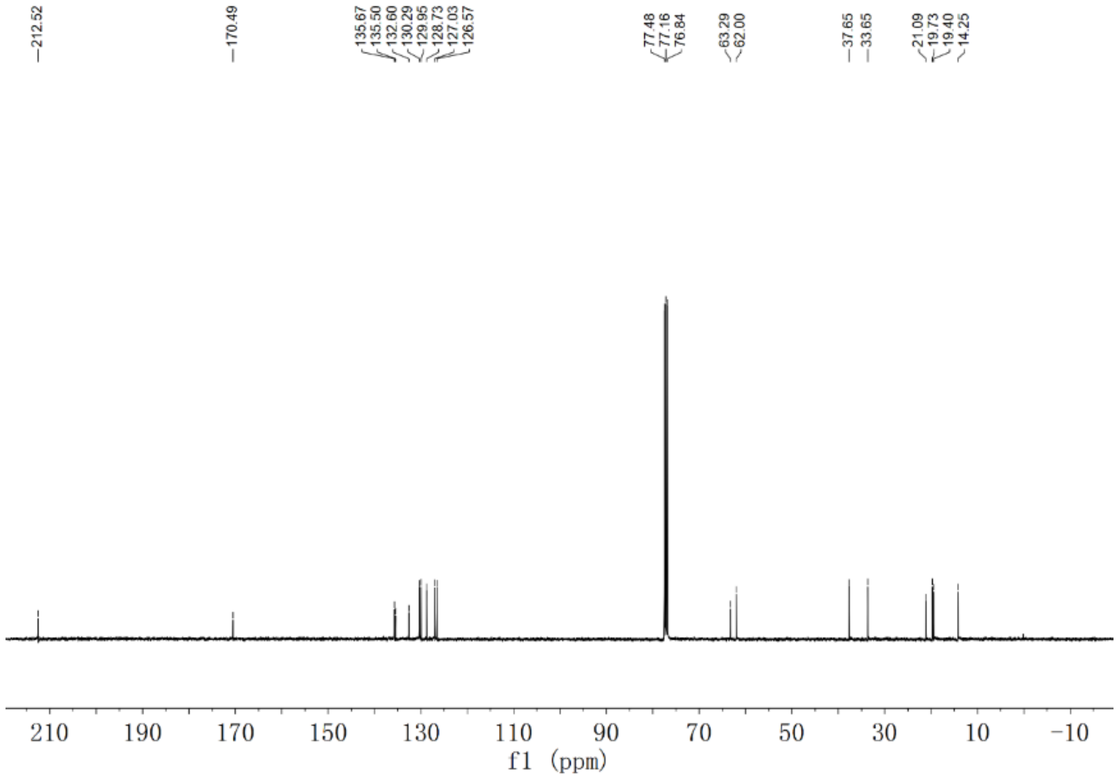

Supplementary Fig. 73. ^{13}C NMR spectra of compound **3s**. (100 MHz, 298K) in $CDCl_3$

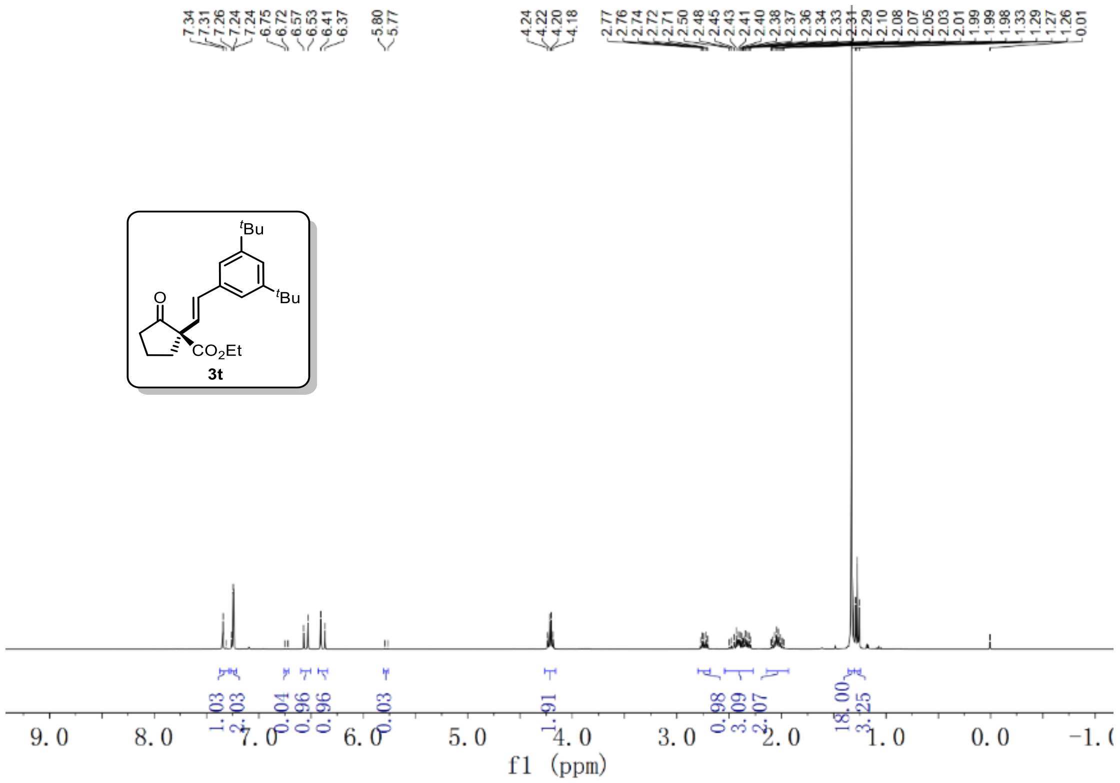

Supplementary Fig. 74. ^{1}H NMR spectra of compound **3t**. (400 MHz, 298K) in $CDCl_3$

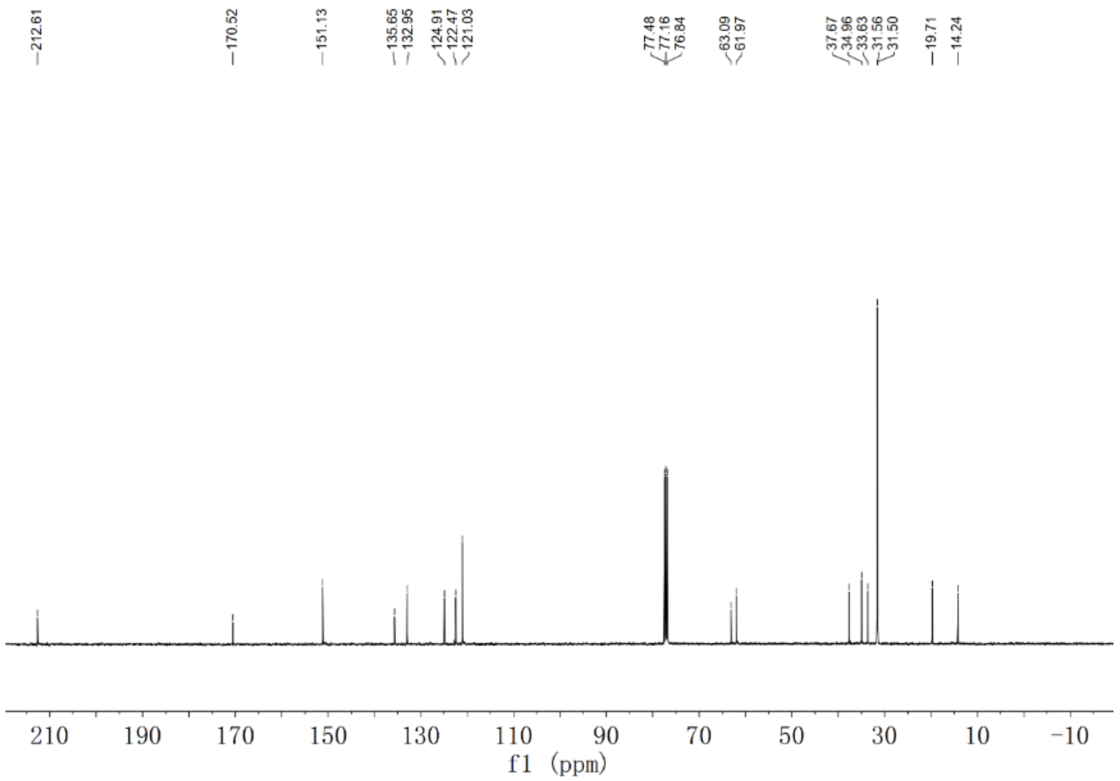

Supplementary Fig. 75. ^{13}C NMR spectra of compound **3t**. (100 MHz, 298K) in $CDCl_3$

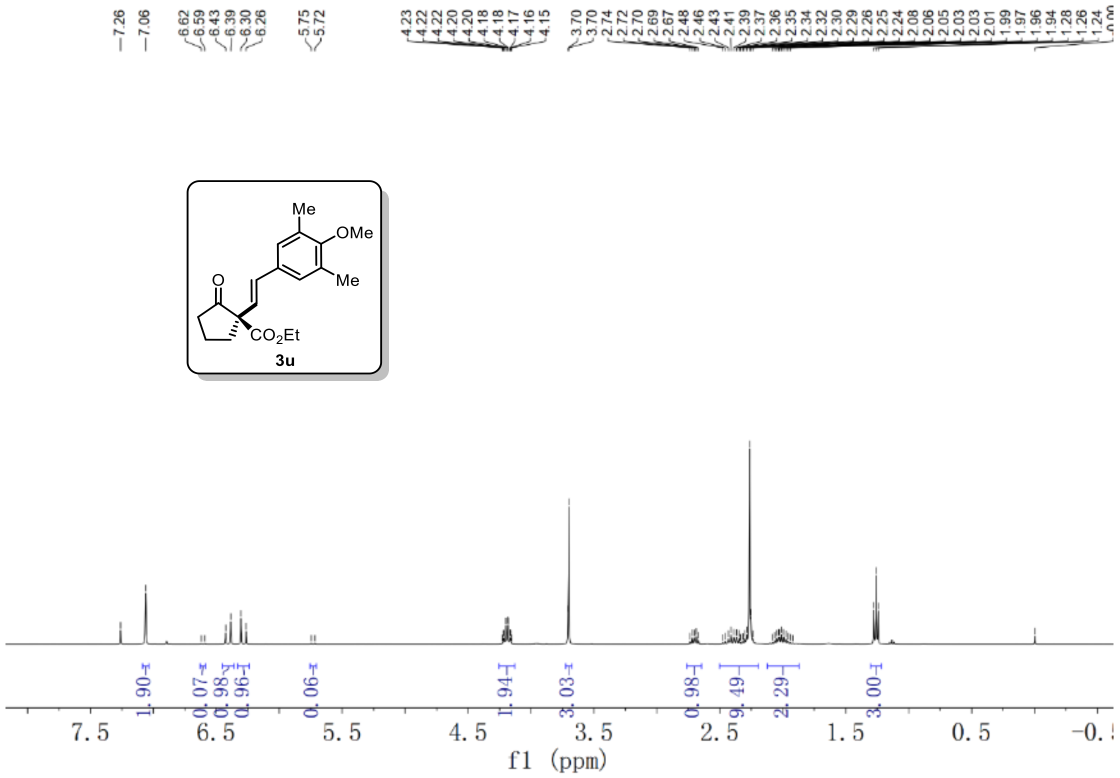

Supplementary Fig. 76. ^{1}H NMR spectra of compound **3u**. (400 MHz, 298K) in $CDCl_3$

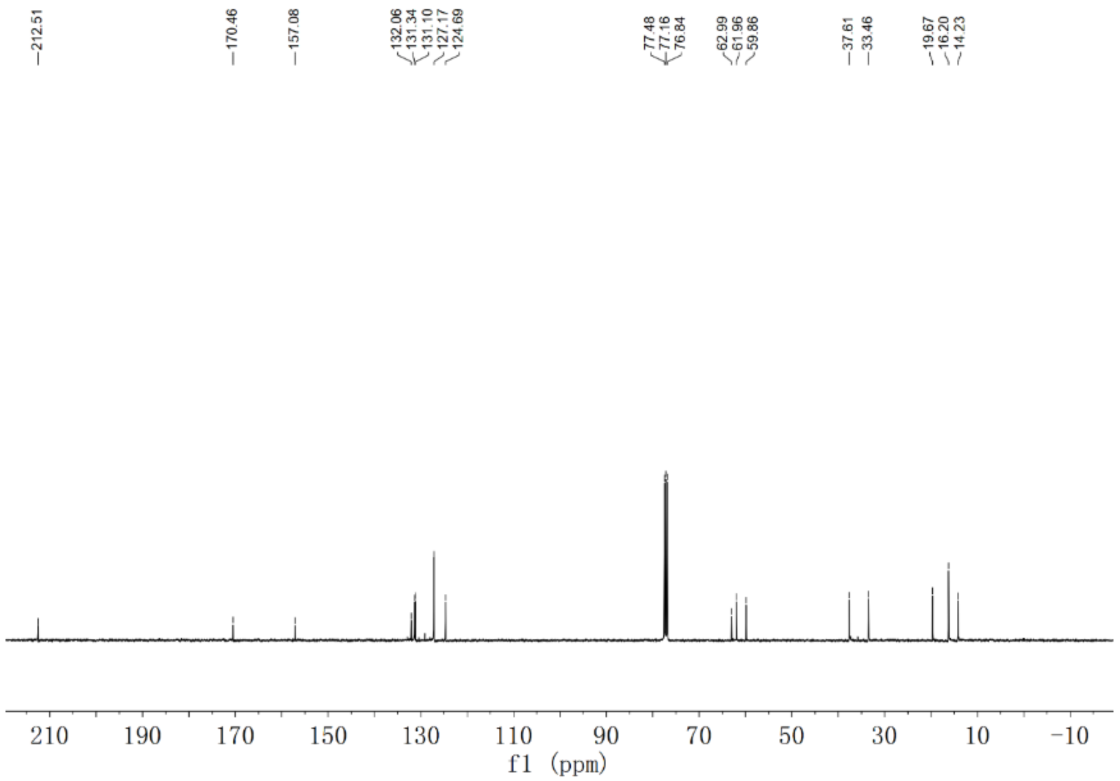

Supplementary Fig. 77. ^{13}C NMR spectra of compound **3u**. (100 MHz, 298K) in $CDCl_3$

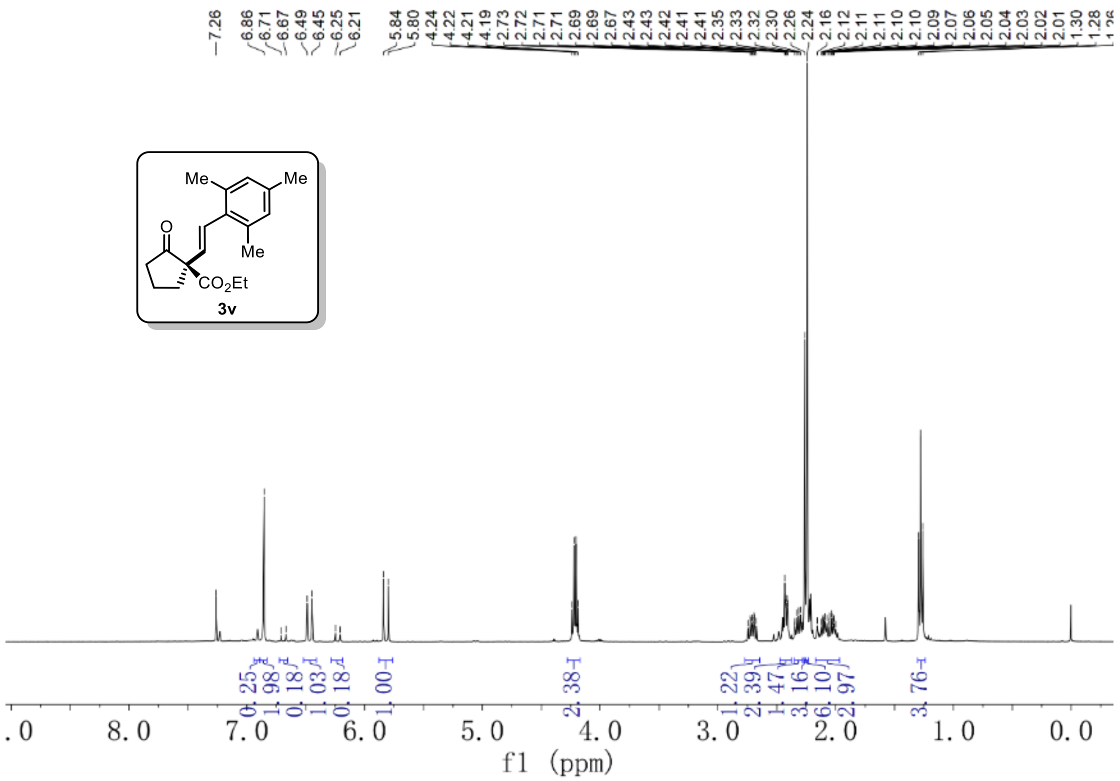

Supplementary Fig. 78. ^{1}H NMR spectra of compound **3v**. (400 MHz, 298K) in $CDCl_3$

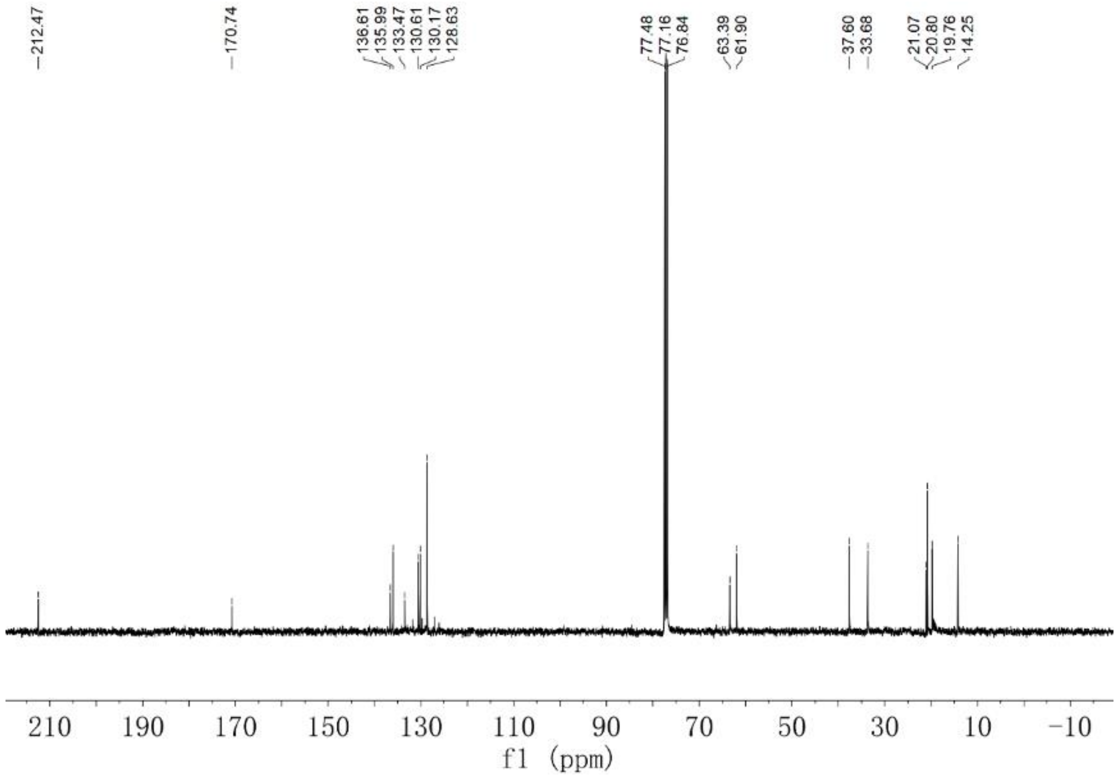

Supplementary Fig. 79. ^{13}C NMR spectra of compound **3v**. (100 MHz, 298K) in $CDCl_3$

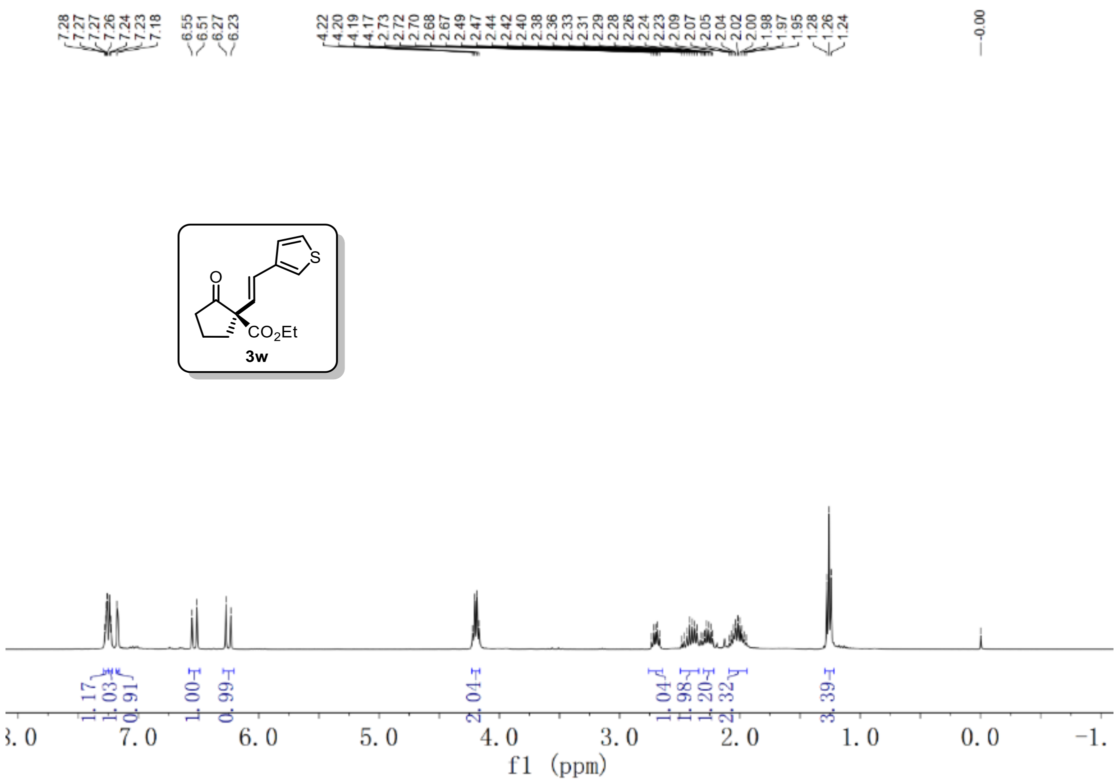

Supplementary Fig. 80. ^{1}H NMR spectra of compound **3w**. (400 MHz, 298K) in $CDCl_3$

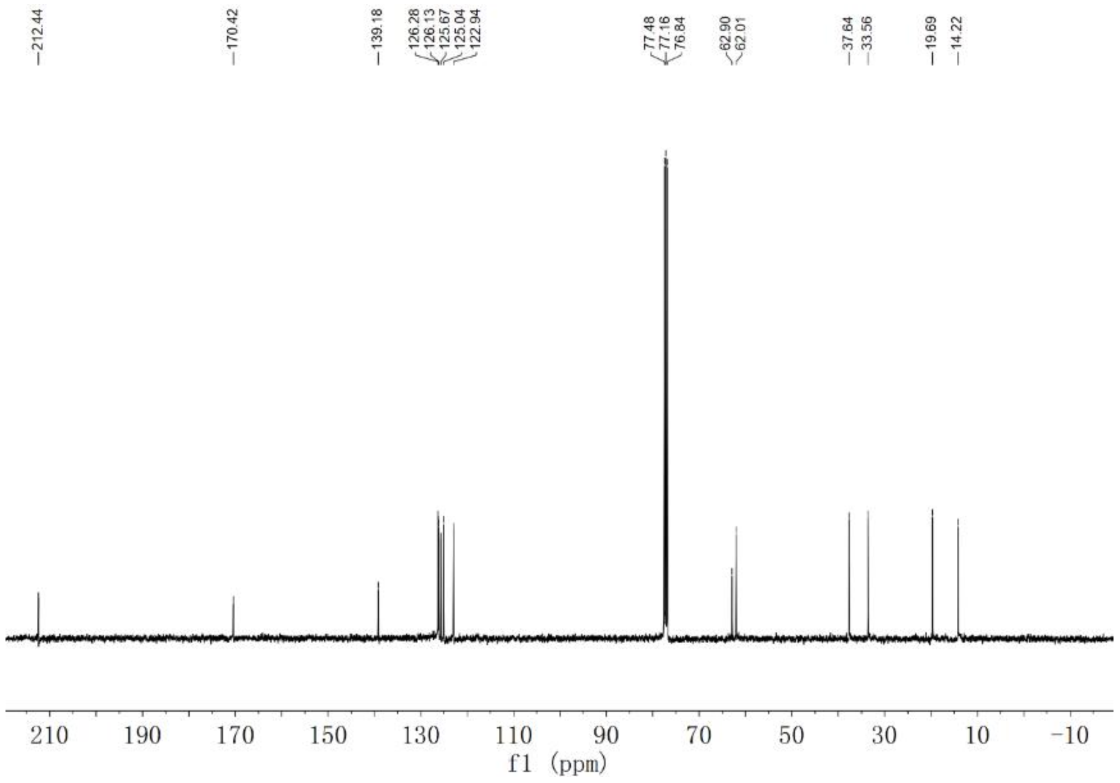

Supplementary Fig. 81. ^{13}C NMR spectra of compound **3w**. (100 MHz, 298K) in $CDCl_3$

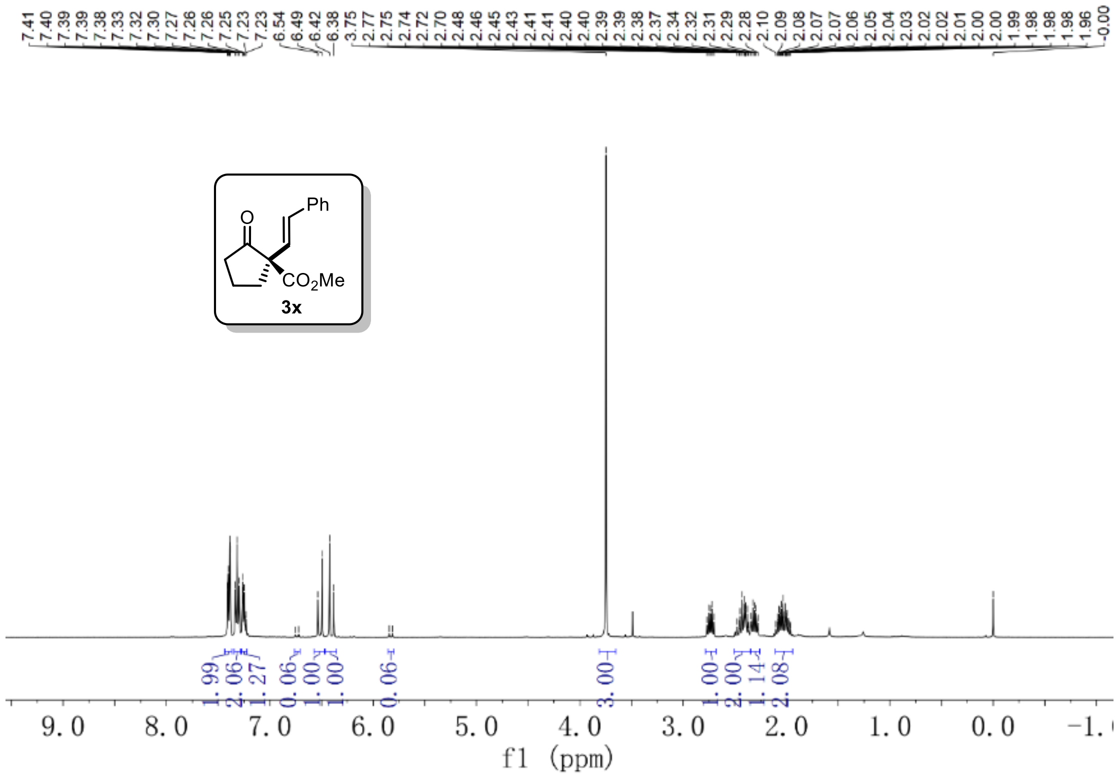

Supplementary Fig. 82. ^{1}H NMR spectra of compound **3x**. (400 MHz, 298K) in $CDCl_3$

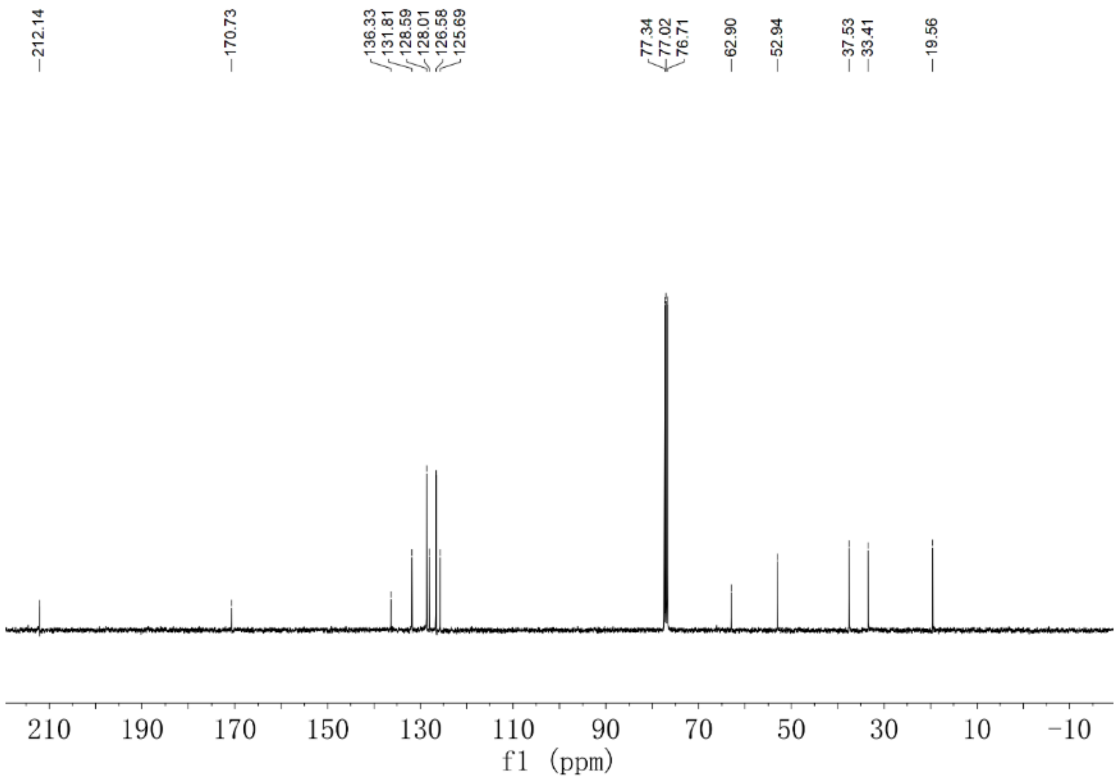

Supplementary Fig. 83. ^{13}C NMR spectra of compound **3x**. (100 MHz, 298K) in $CDCl_3$

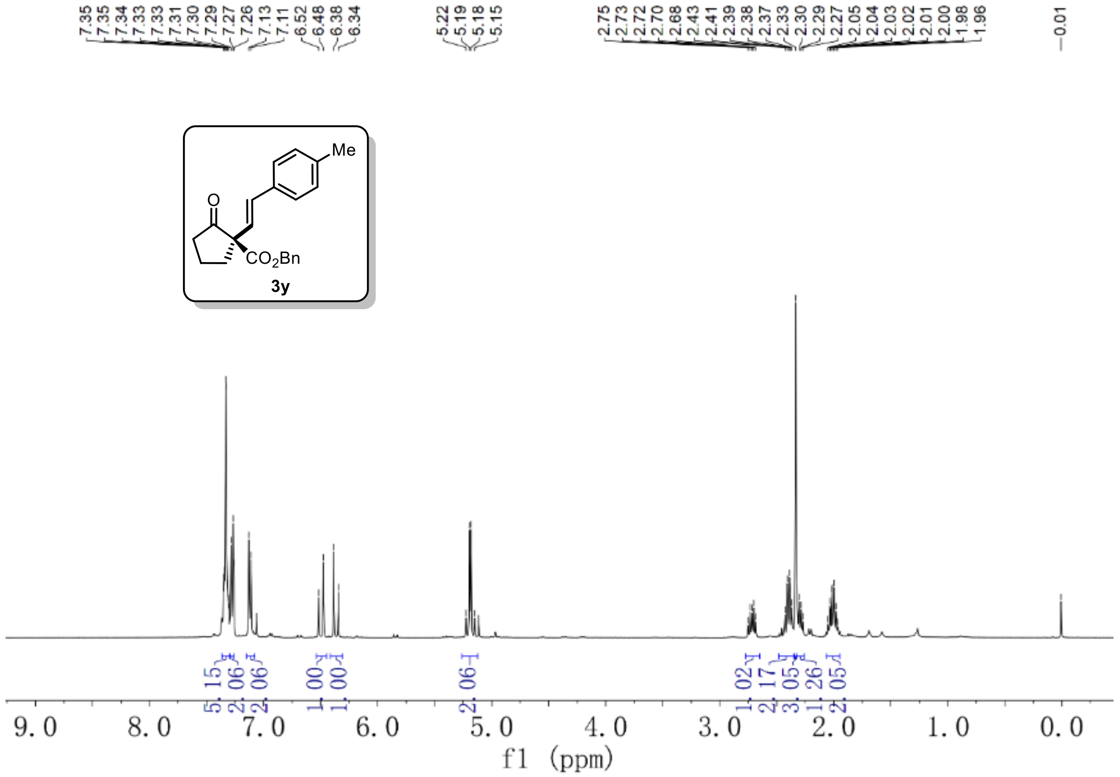

Supplementary Fig. 84. ^{1}H NMR spectra of compound **3y**. (400 MHz, 298K) in $CDCl_3$

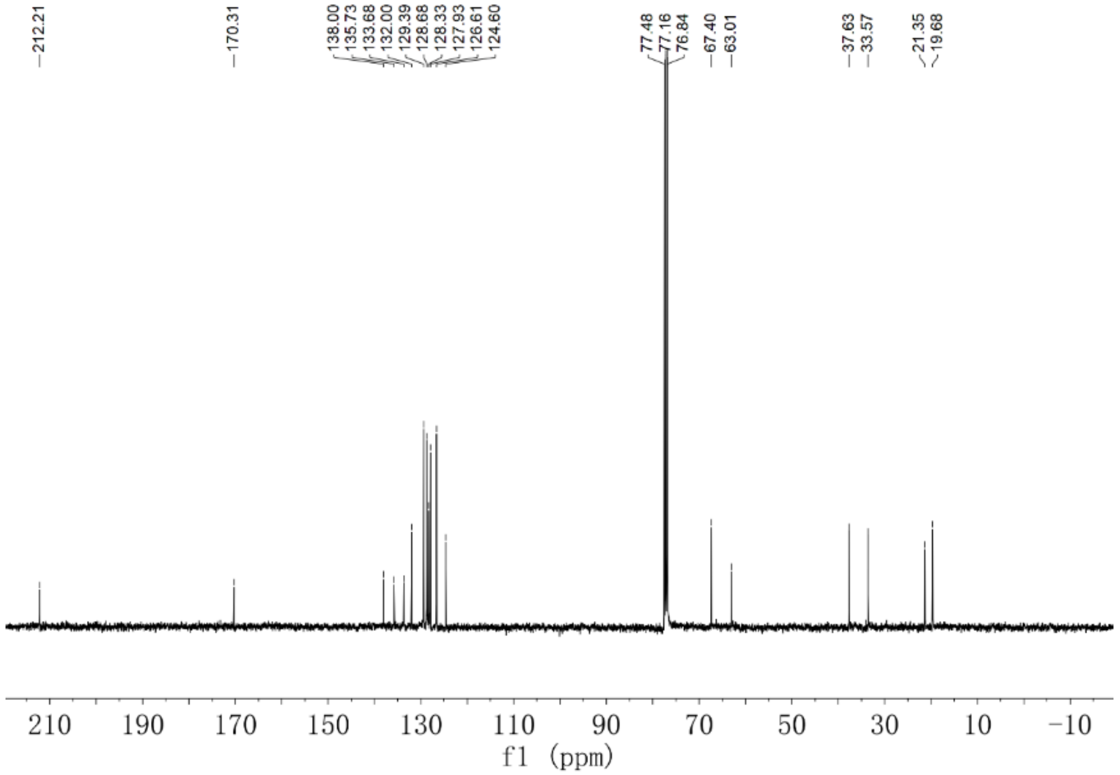

Supplementary Fig. 85. ^{13}C NMR spectra of compound **3y**. (100 MHz, 298K) in $CDCl_3$

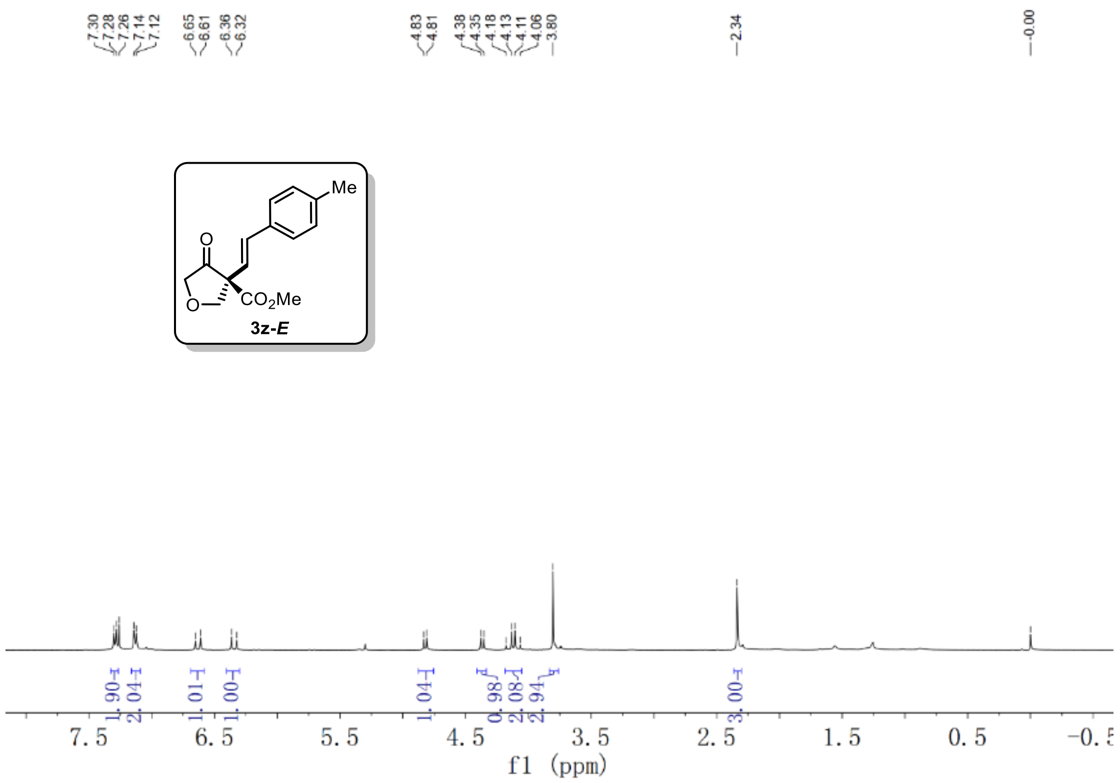

Supplementary Fig. 86. ^{1}H NMR spectra of compound **3z-*E***. (400 MHz, 298K) in $CDCl_3$

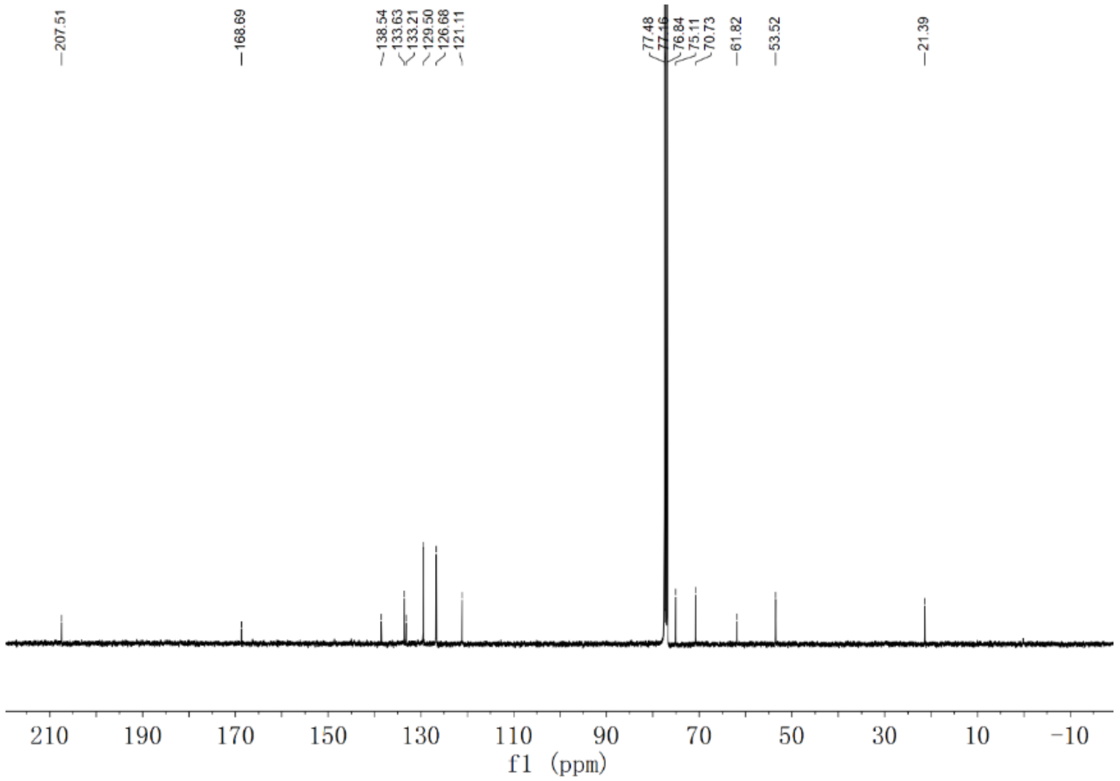

Supplementary Fig. 87. ^{13}C NMR spectra of compound **3z-*E***. (100 MHz, 298K) in $CDCl_3$

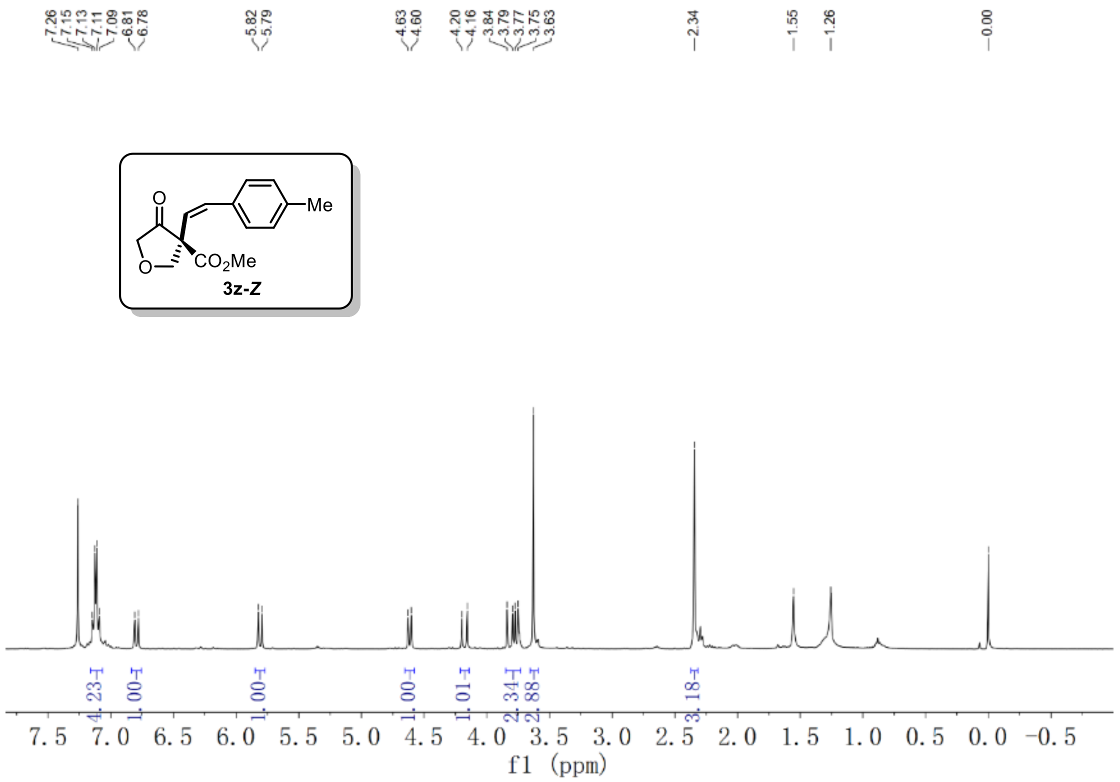

Supplementary Fig. 88. ^{1}H NMR spectra of compound **3z-*Z***. (400 MHz, 298K) in $CDCl_3$

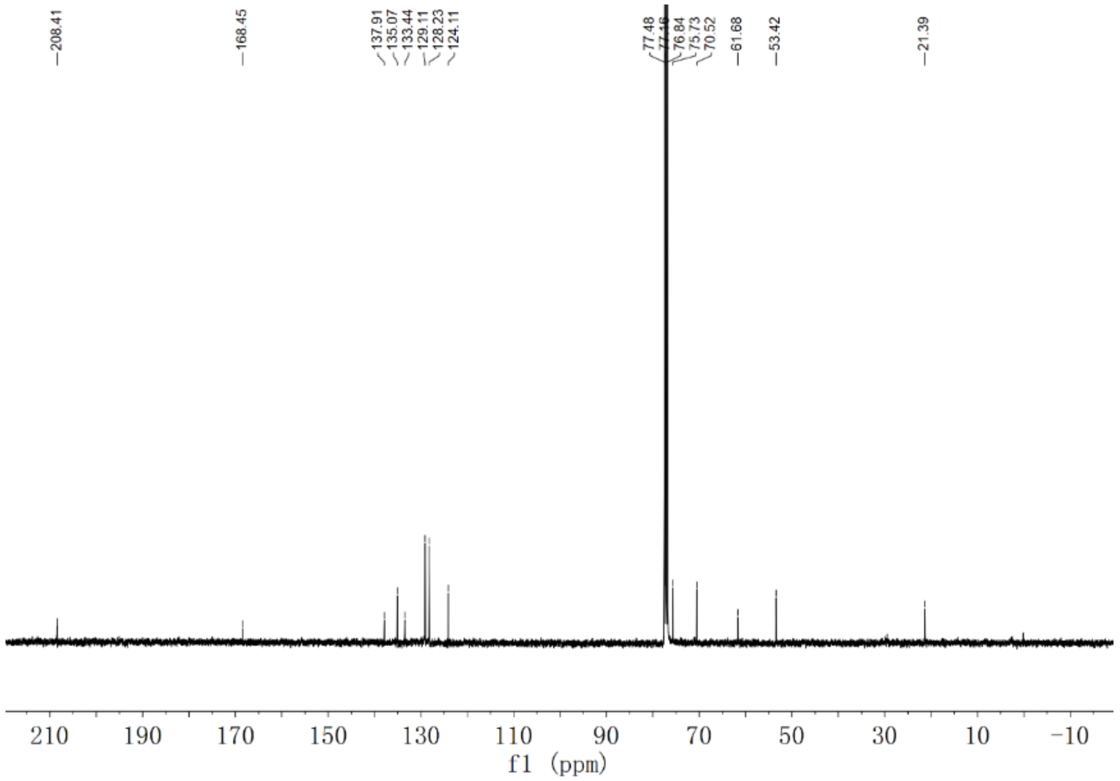

Supplementary Fig. 89. ^{13}C NMR spectra of compound **3z-*Z***. (100 MHz, 298K) in $CDCl_3$

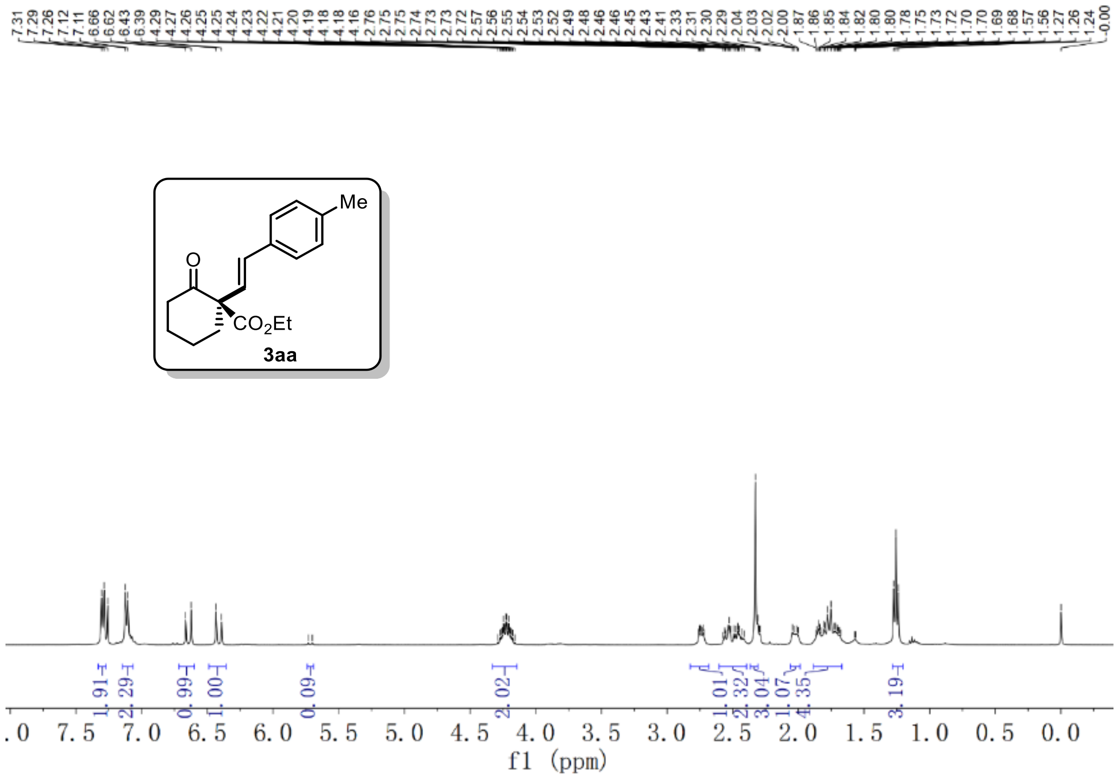

Supplementary Fig. 90. ^{1}H NMR spectra of compound **3aa**. (400 MHz, 298K) in $CDCl_3$

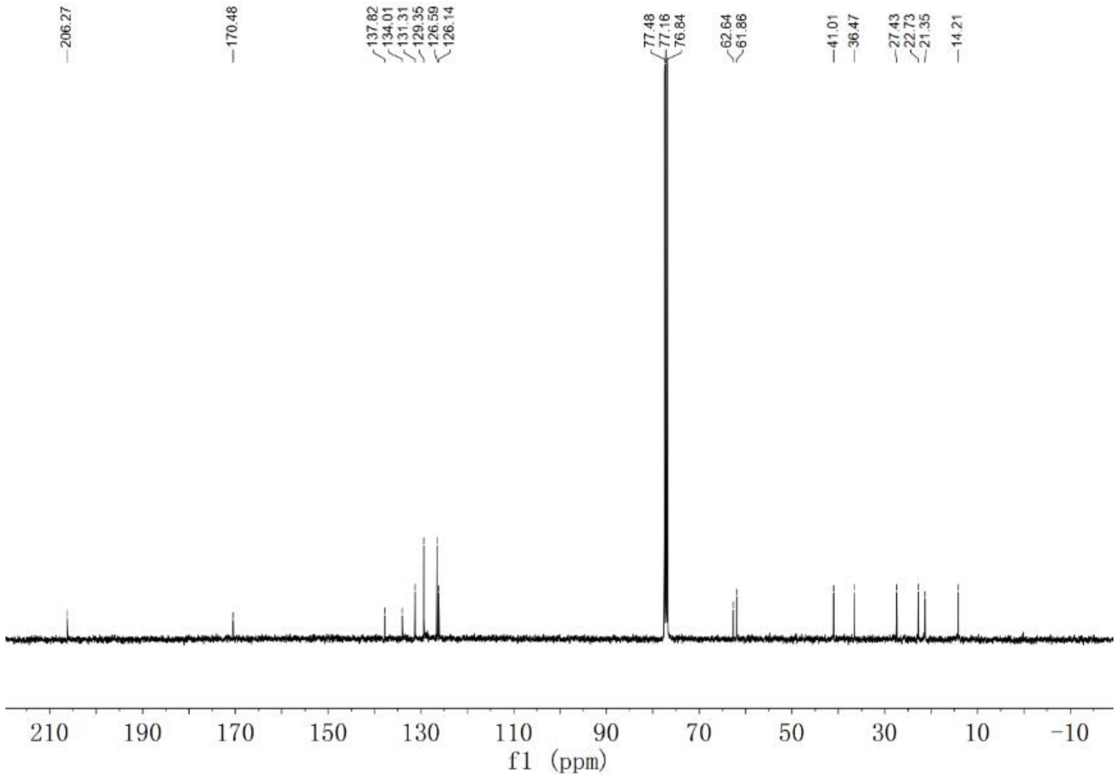

Supplementary Fig. 91. ^{13}C NMR spectra of compound **3aa**. (100 MHz, 298K) in $CDCl_3$

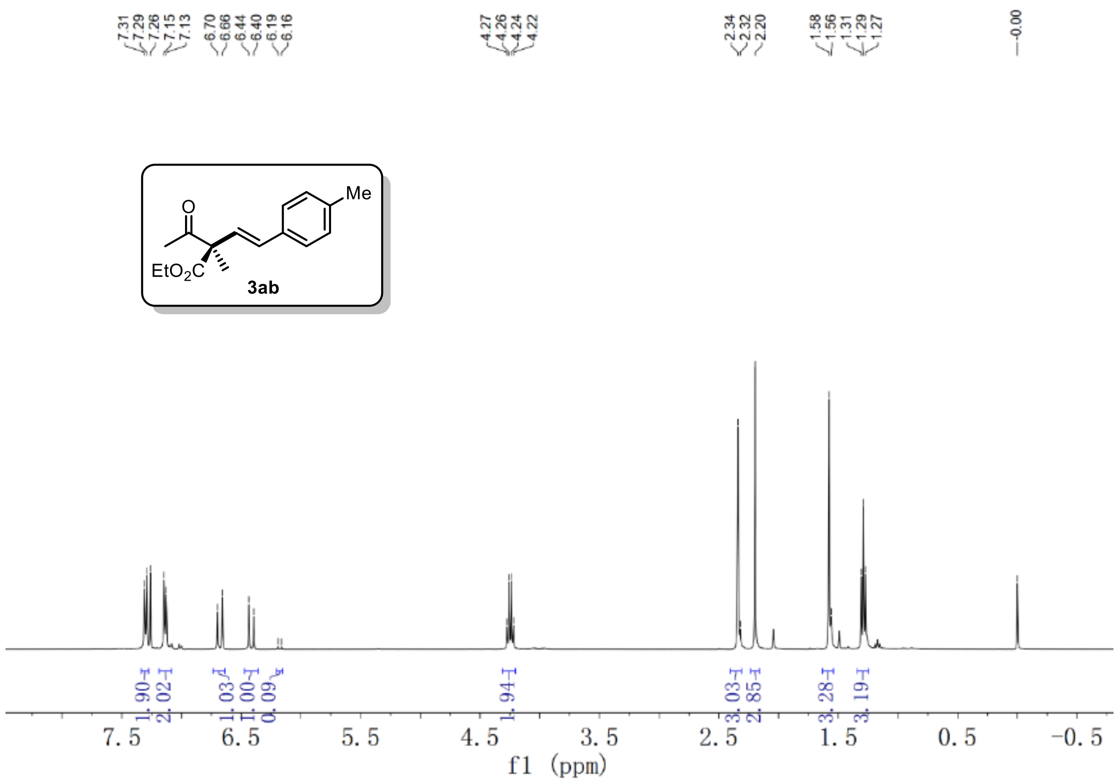

Supplementary Fig. 92. ^{1}H NMR spectra of compound **3ab**. (400 MHz, 298K) in $CDCl_3$

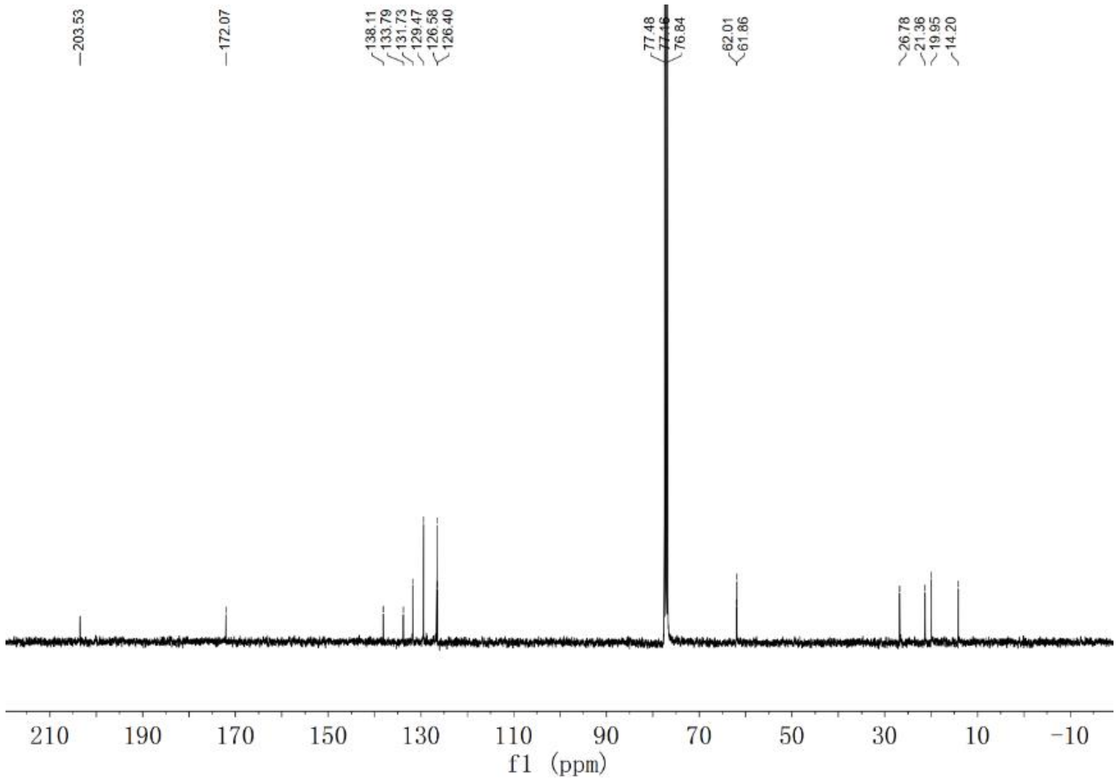

Supplementary Fig. 93. ^{13}C NMR spectra of compound **3ab**. (100 MHz, 298K) in $CDCl_3$

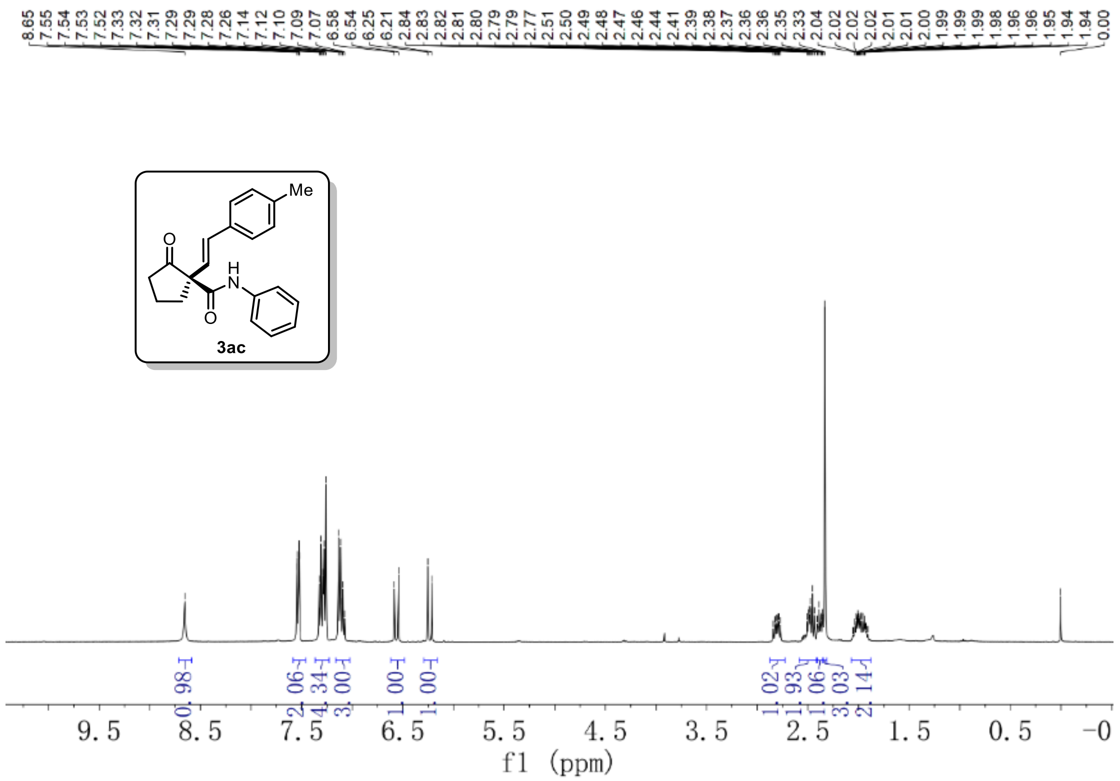

Supplementary Fig. 94. ^{1}H NMR spectra of compound **3ac**. (400 MHz, 298K) in $CDCl_3$

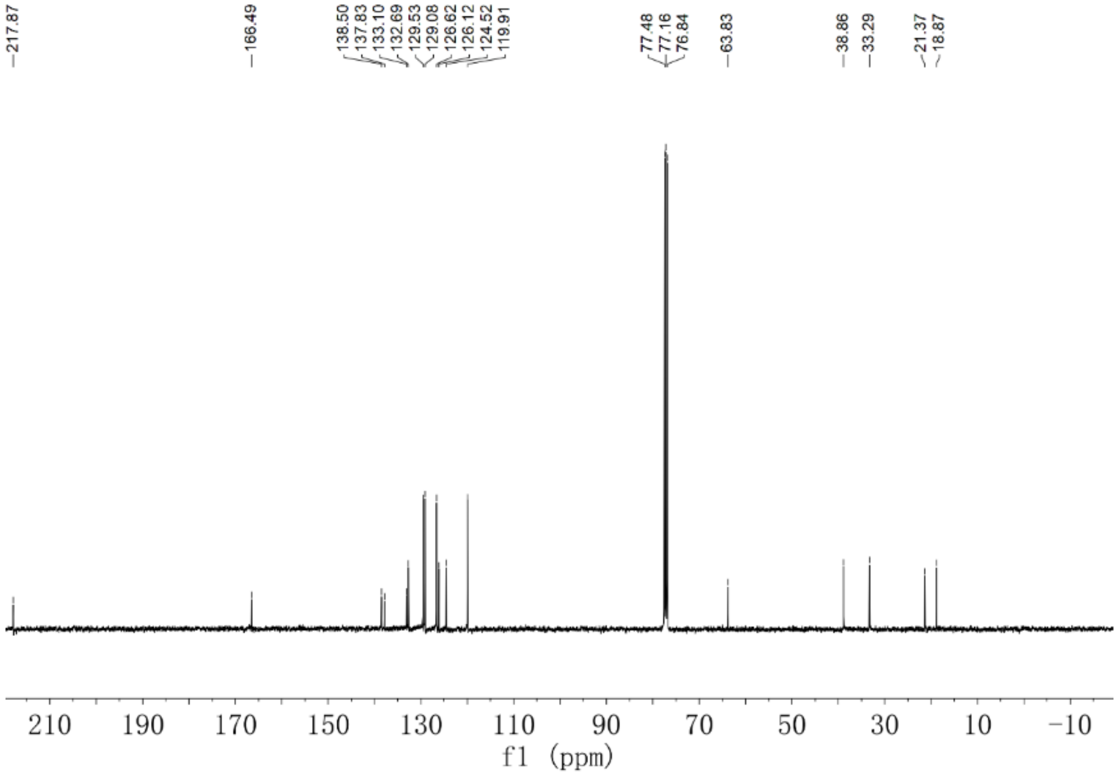

Supplementary Fig. 95. ^{13}C NMR spectra of compound **3ac**. (100 MHz, 298K) in $CDCl_3$

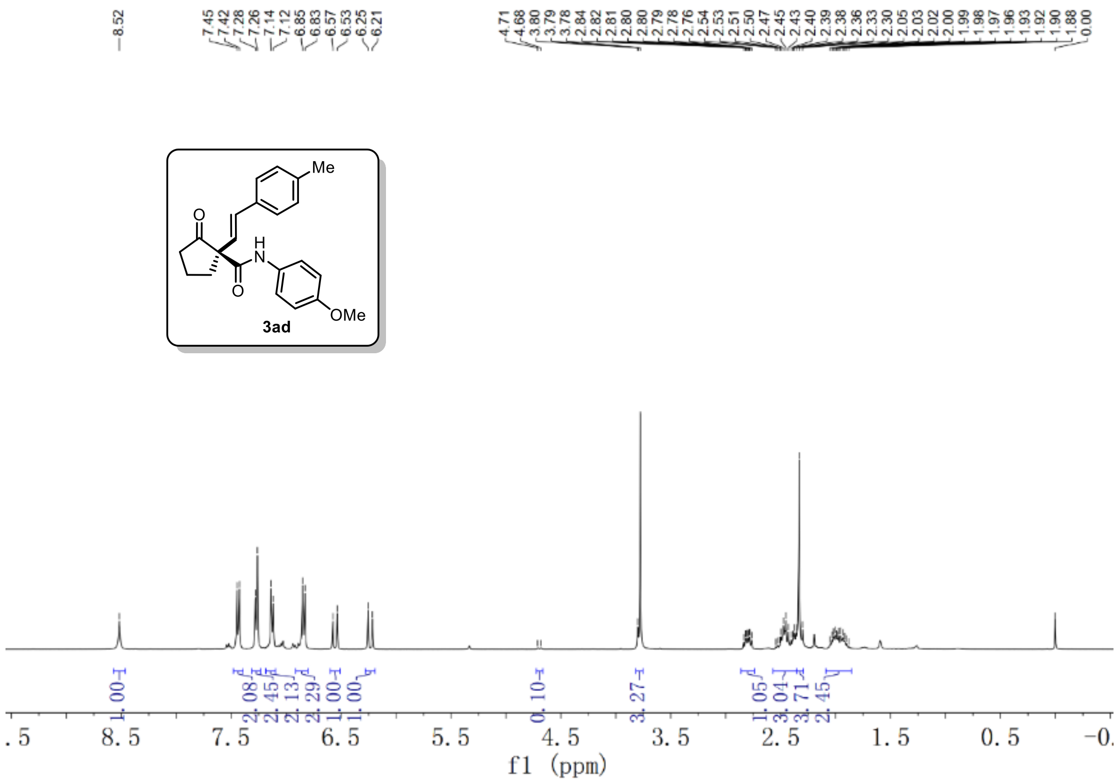

Supplementary Fig. 96. ^{1}H NMR spectra of compound **3ad**. (400 MHz, 298K) in $CDCl_3$

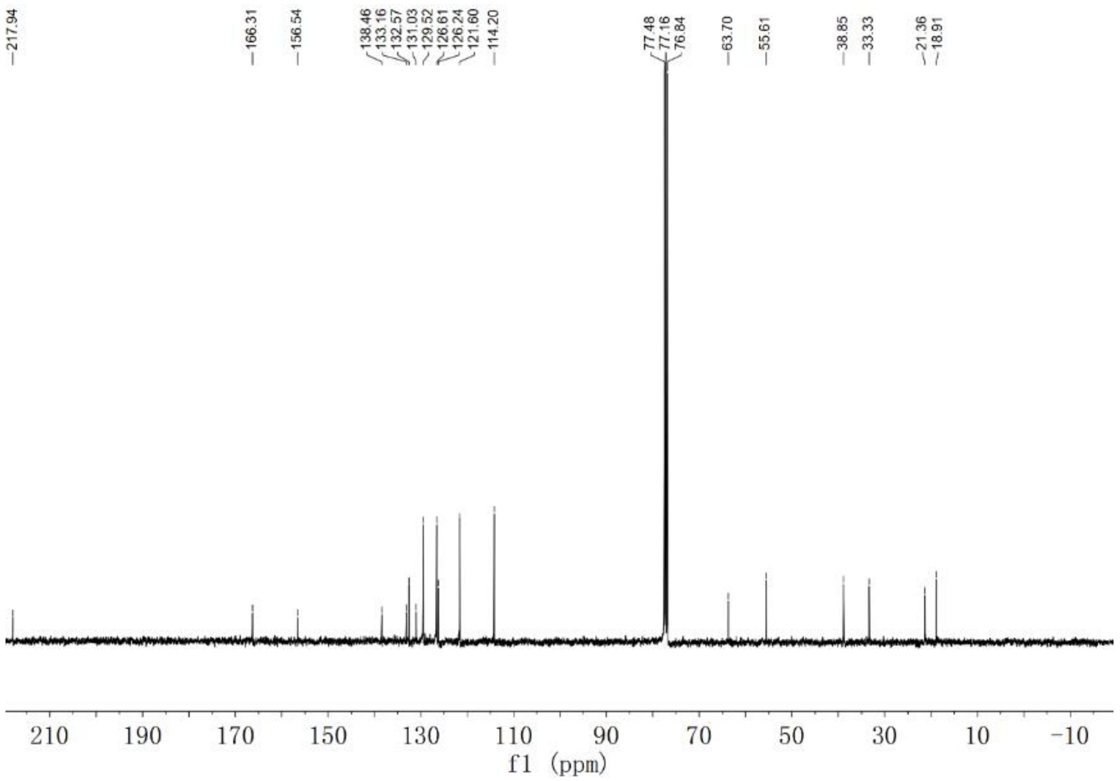

Supplementary Fig. 97. ^{13}C NMR spectra of compound **3ad**. (100 MHz, 298K) in $CDCl_3$

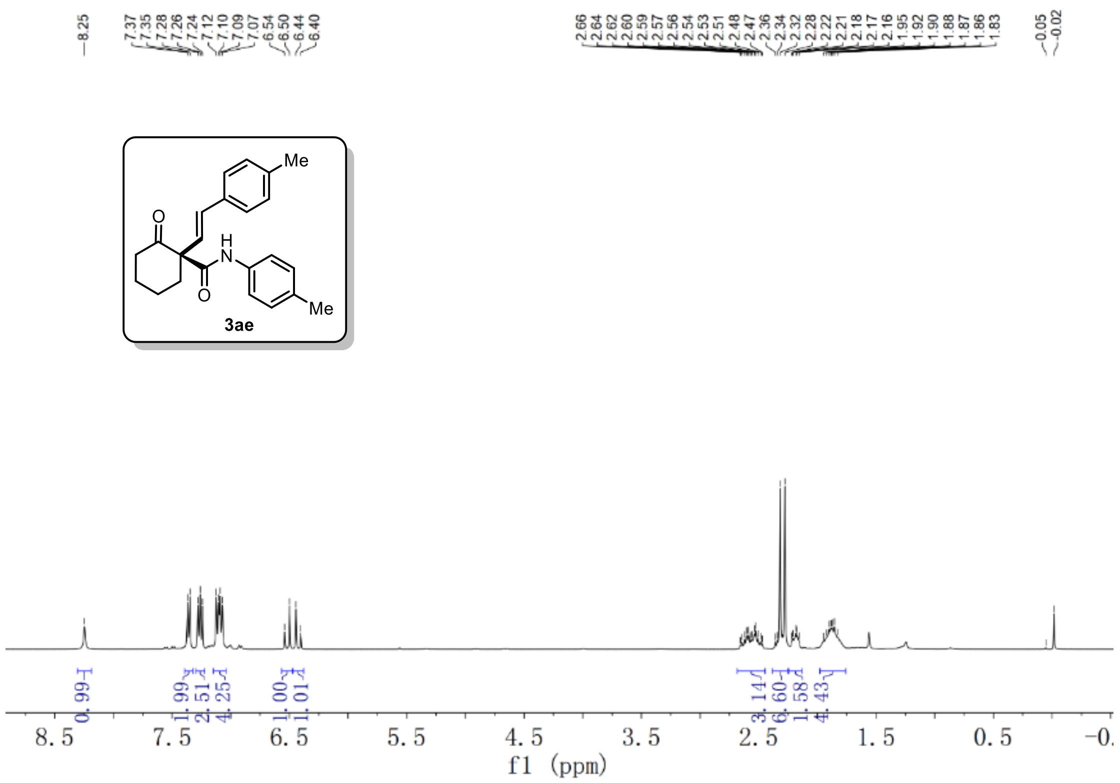

Supplementary Fig. 98. ^{1}H NMR spectra of compound **3ae**. (400 MHz, 298K) in $CDCl_3$

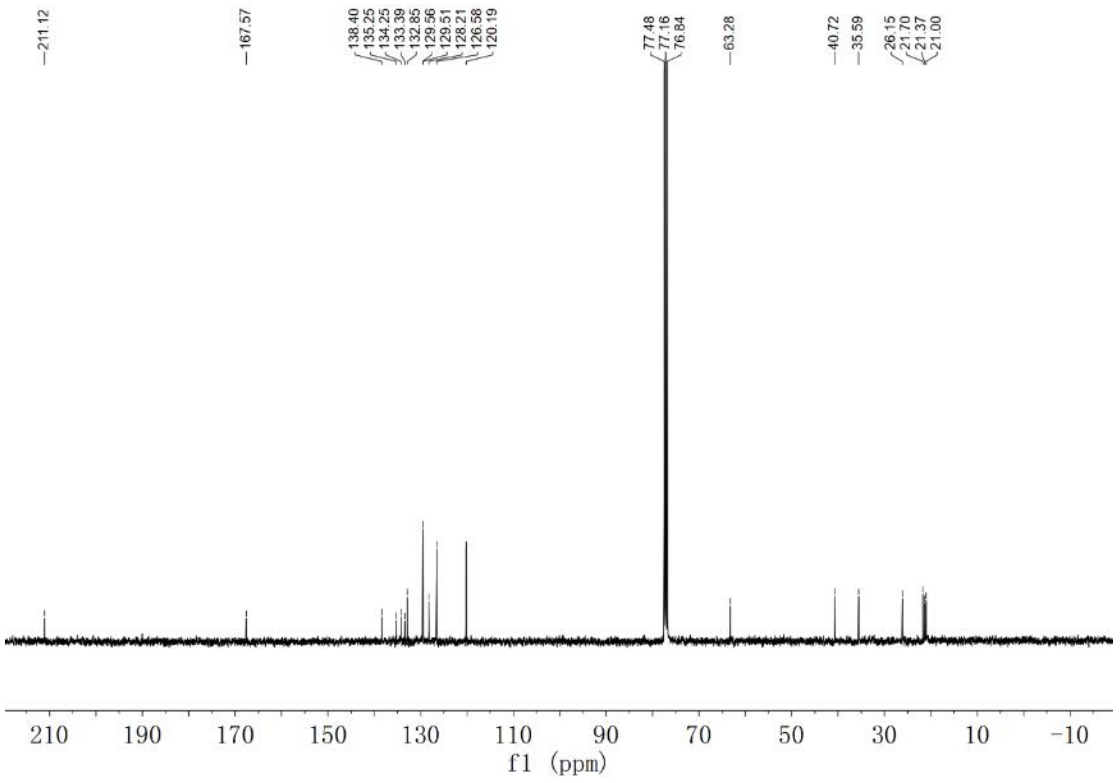

Supplementary Fig. 99. ^{13}C NMR spectra of compound **3ae**. (100 MHz, 298K) in $CDCl_3$

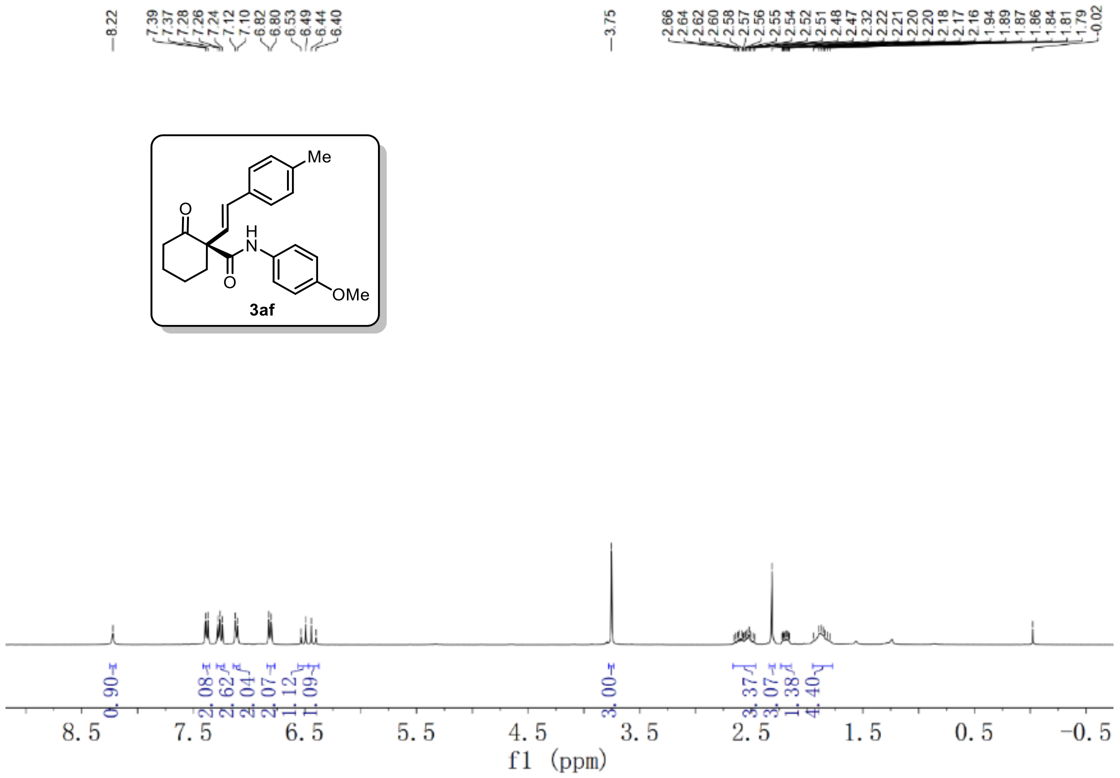

Supplementary Fig. 100. ^{1}H NMR spectra of compound **3af**. (400 MHz, 298K) in $CDCl_3$

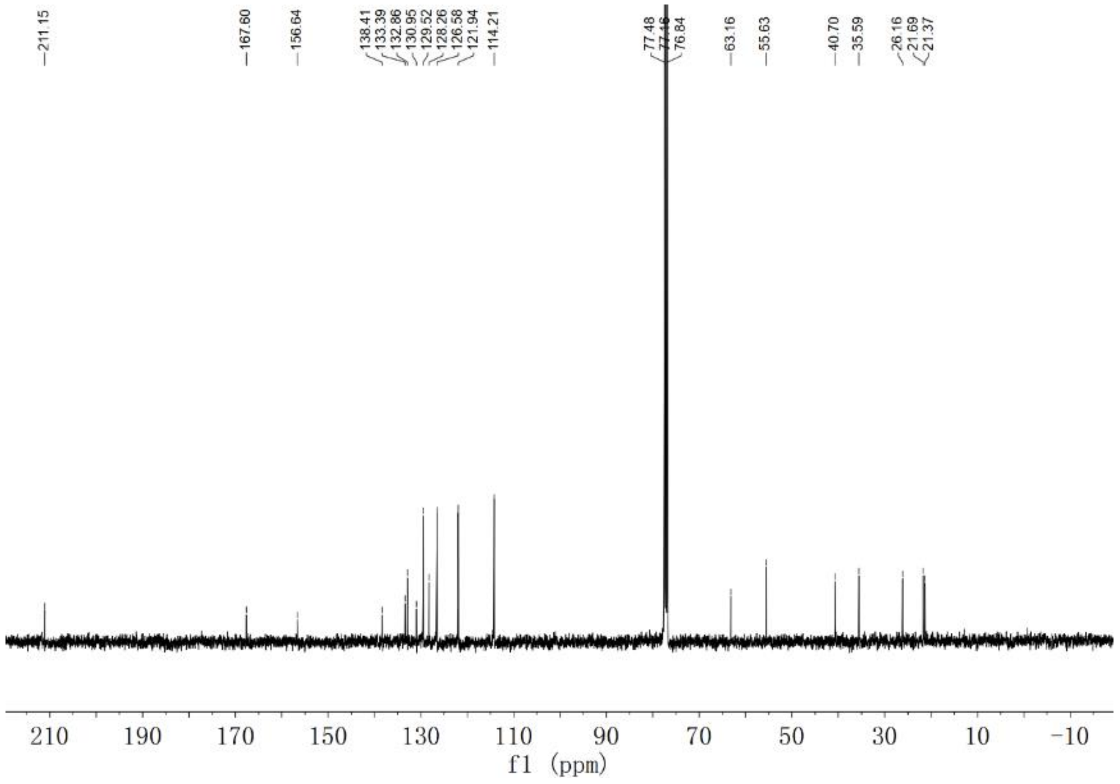

Supplementary Fig. 101. ^{13}C NMR spectra of compound **3af**. (100 MHz, 298K) in $CDCl_3$

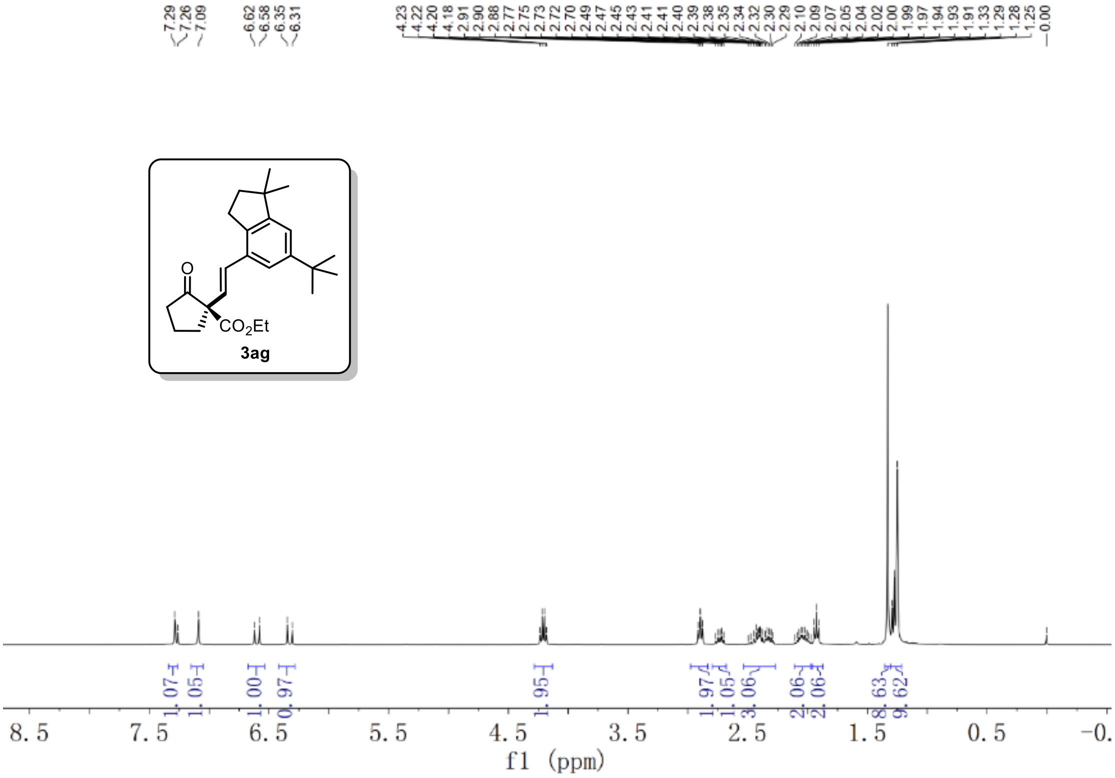

Supplementary Fig. 102. ^{1}H NMR spectra of compound **3ag**. (400 MHz, 298K) in $CDCl_3$

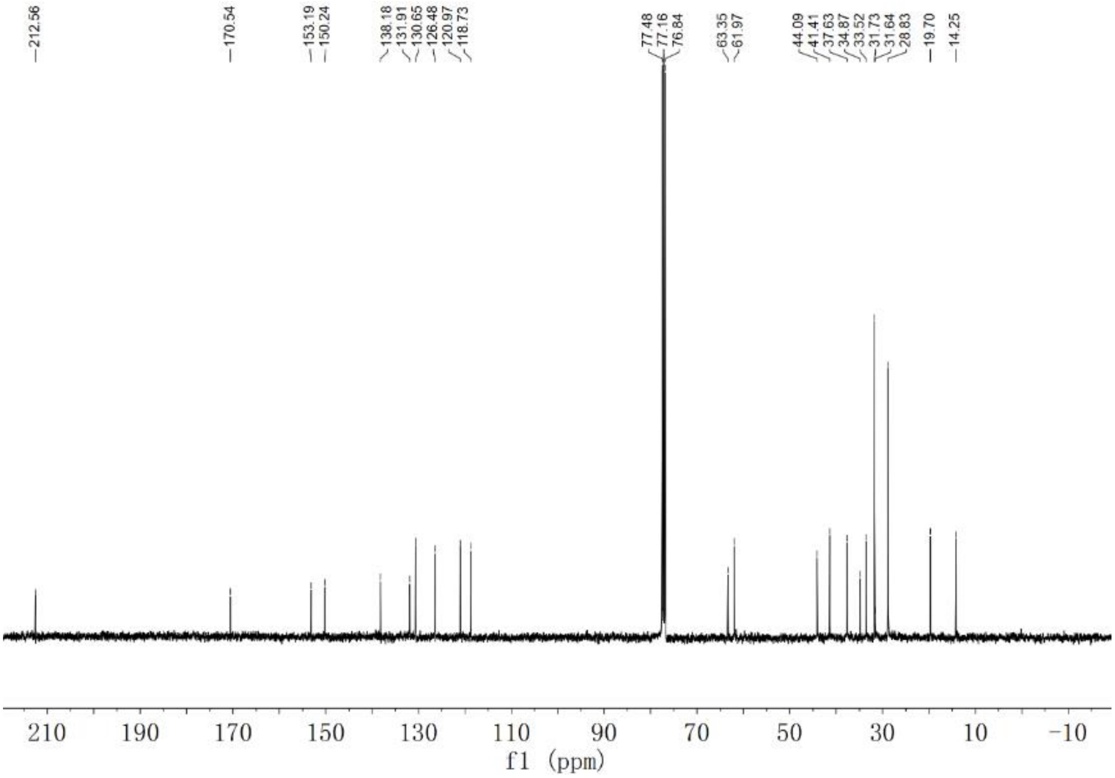

Supplementary Fig. 103. ^{13}C NMR spectra of compound **3ag**. (100 MHz, 298K) in $CDCl_3$

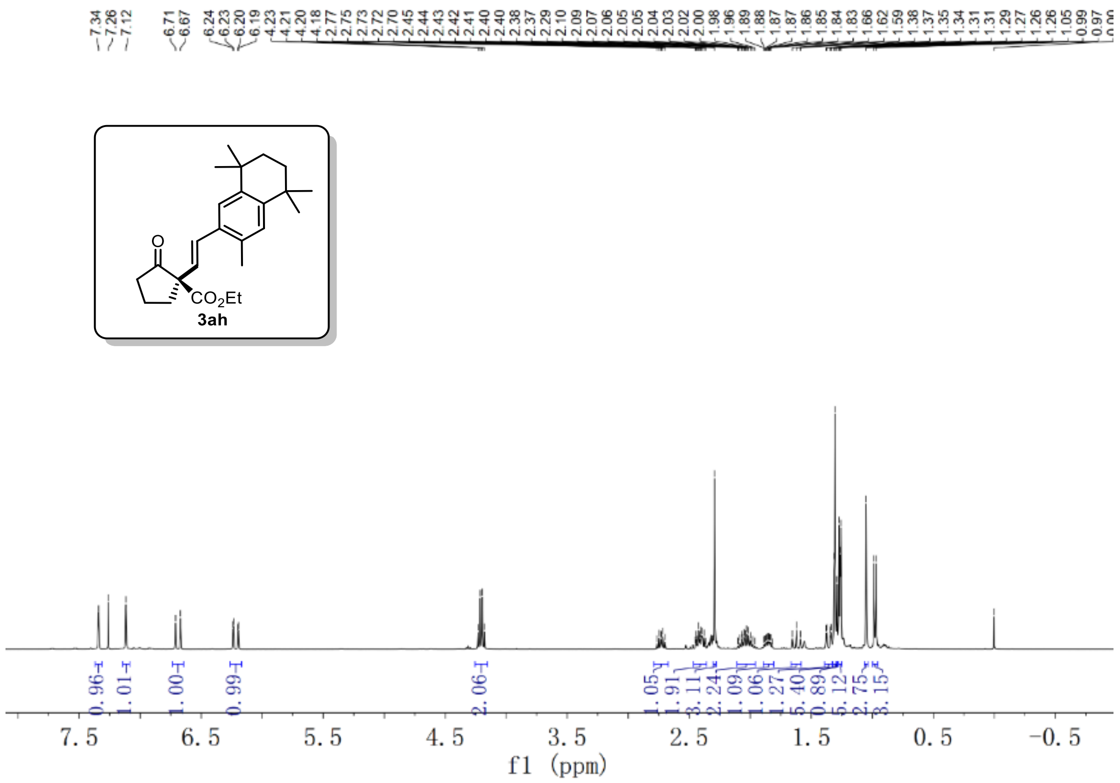

Supplementary Fig. 104. ^{1}H NMR spectra of compound **3ah**. (400 MHz, 298K) in $CDCl_3$

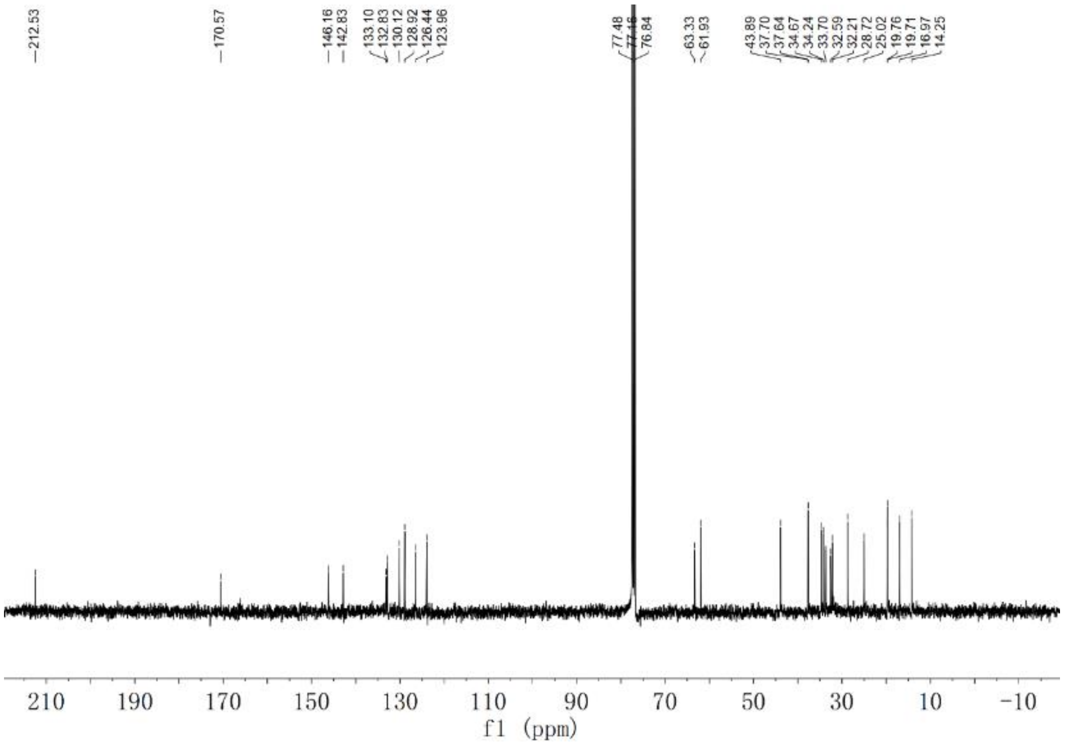

Supplementary Fig. 105. ^{13}C NMR spectra of compound **3ah**. (100 MHz, 298K) in $CDCl_3$

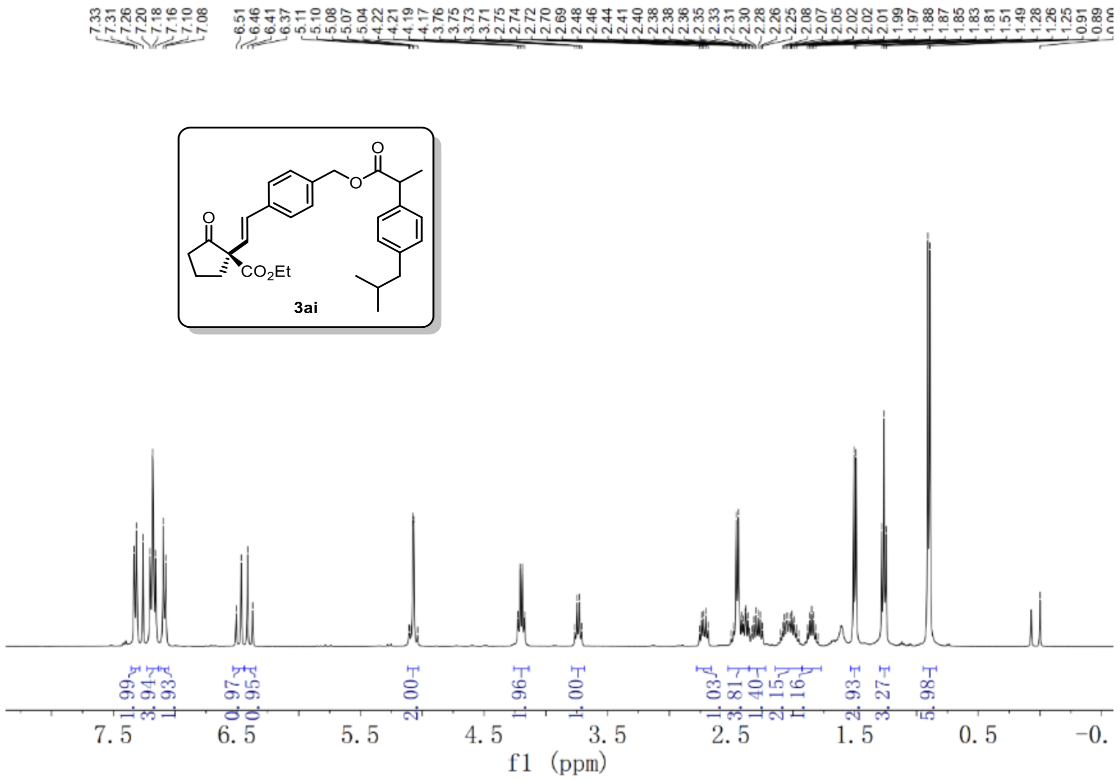

Supplementary Fig. 106. ^{1}H NMR spectra of compound **3ai**. (400 MHz, 298K) in $CDCl_3$

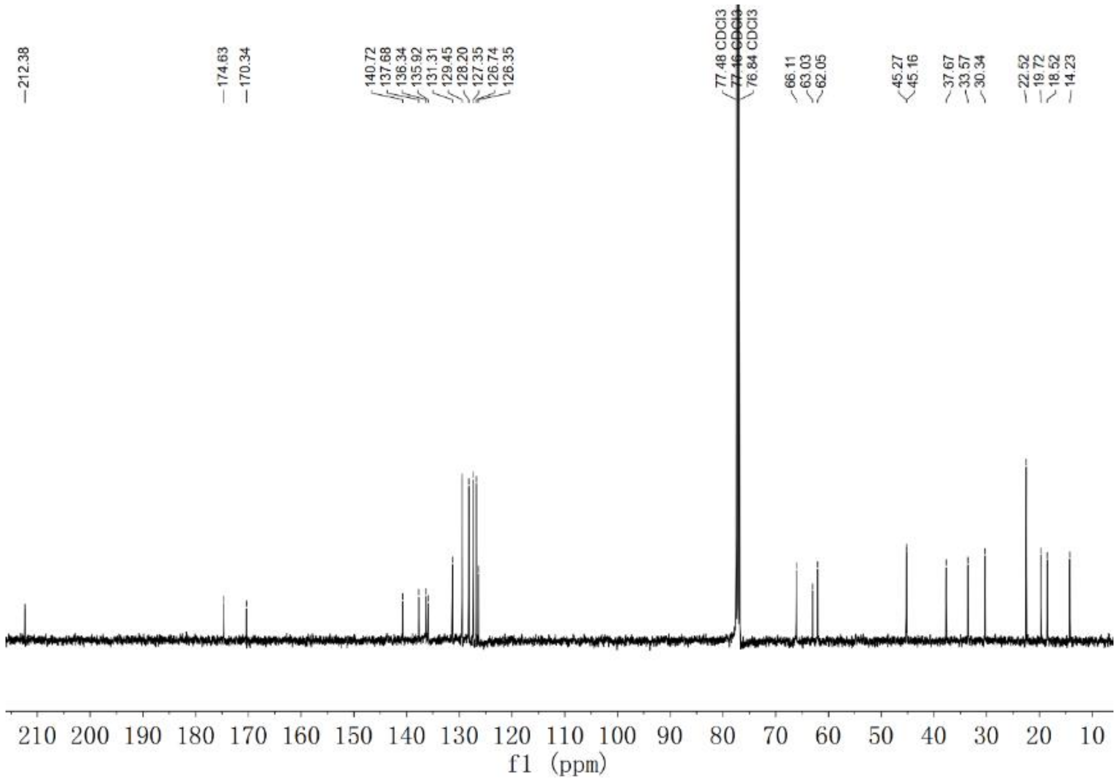

Supplementary Fig. 107. ^{13}C NMR spectra of compound **3ai**. (100 MHz, 298K) in $CDCl_3$

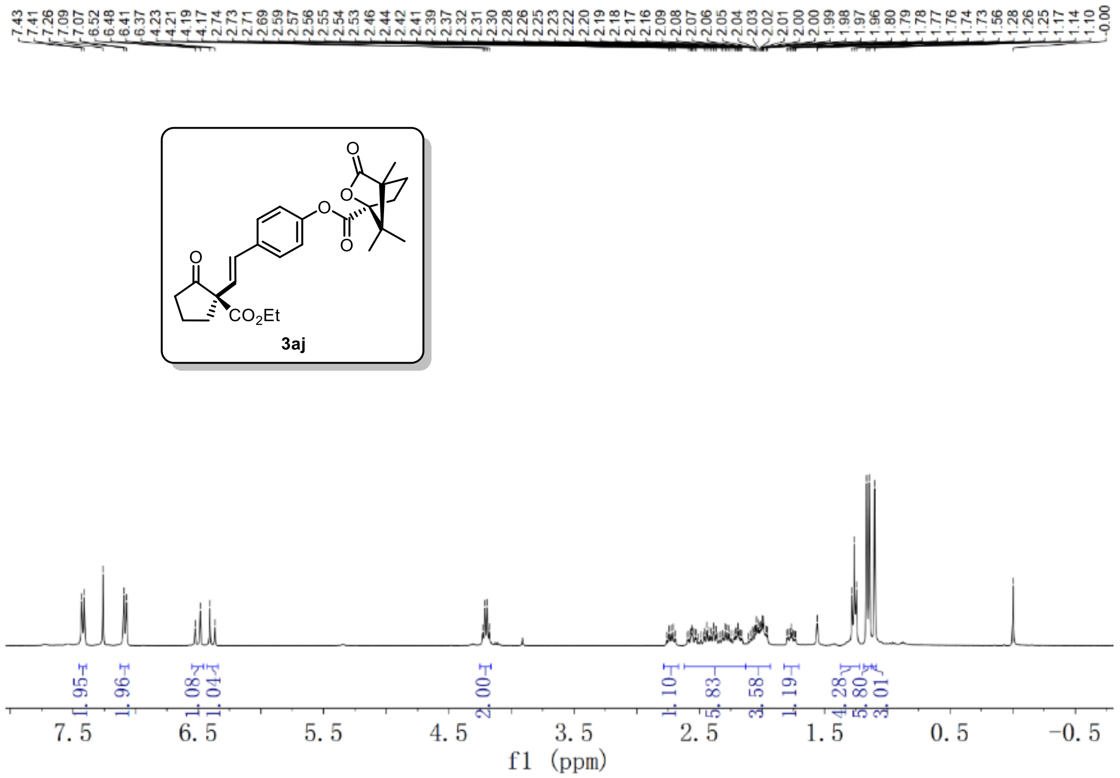

Supplementary Fig. 108. ^{1}H NMR spectra of compound **3aj**. (400 MHz, 298K) in $CDCl_3$

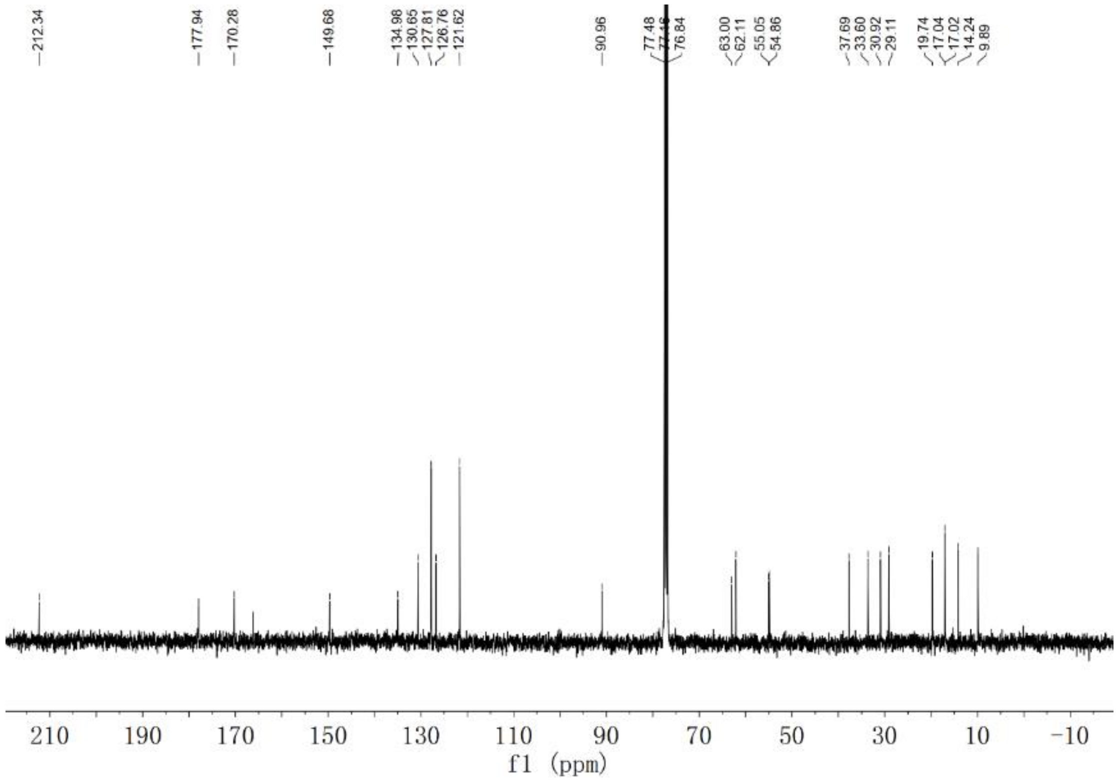

Supplementary Fig. 109. ^{13}C NMR spectra of compound **3aj**. (100 MHz, 298K) in $CDCl_3$

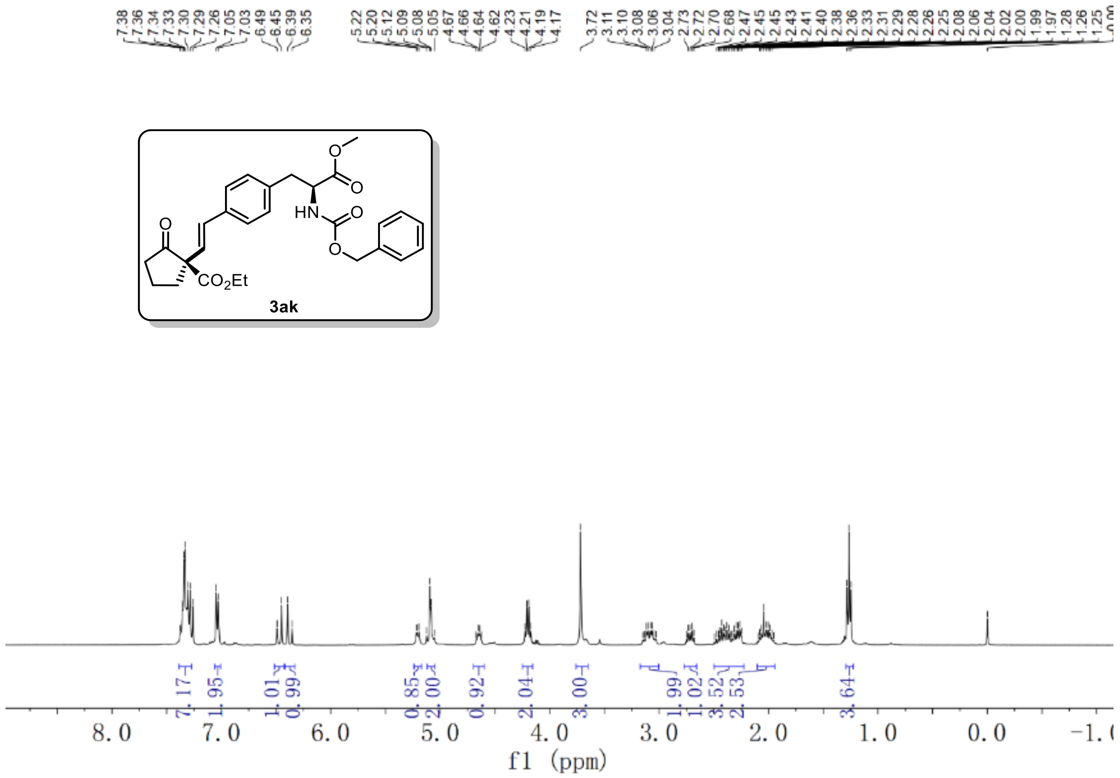

Supplementary Fig. 110. ^{1}H NMR spectra of compound **3ak**. (400 MHz, 298K) in $CDCl_3$

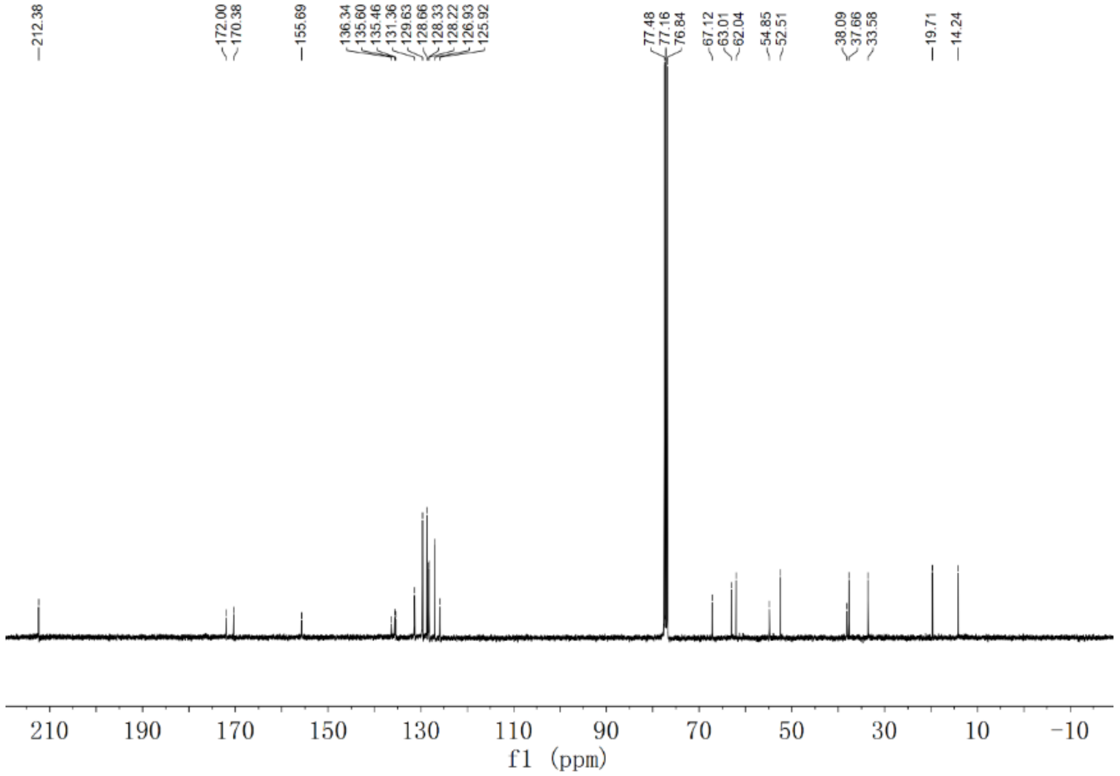

Supplementary Fig. 111. ^{13}C NMR spectra of compound **3ak**. (100 MHz, 298K) in $CDCl_3$

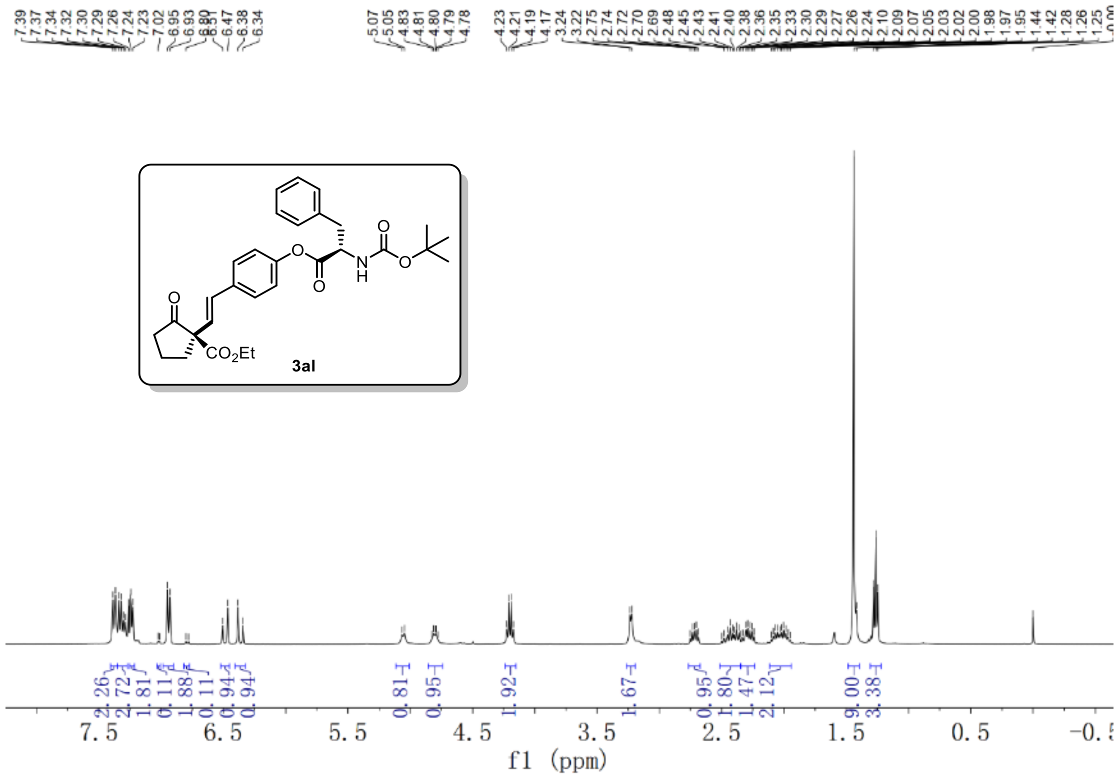

Supplementary Fig. 112. ^{1}H NMR spectra of compound **3al**. (400 MHz, 298K) in $CDCl_3$

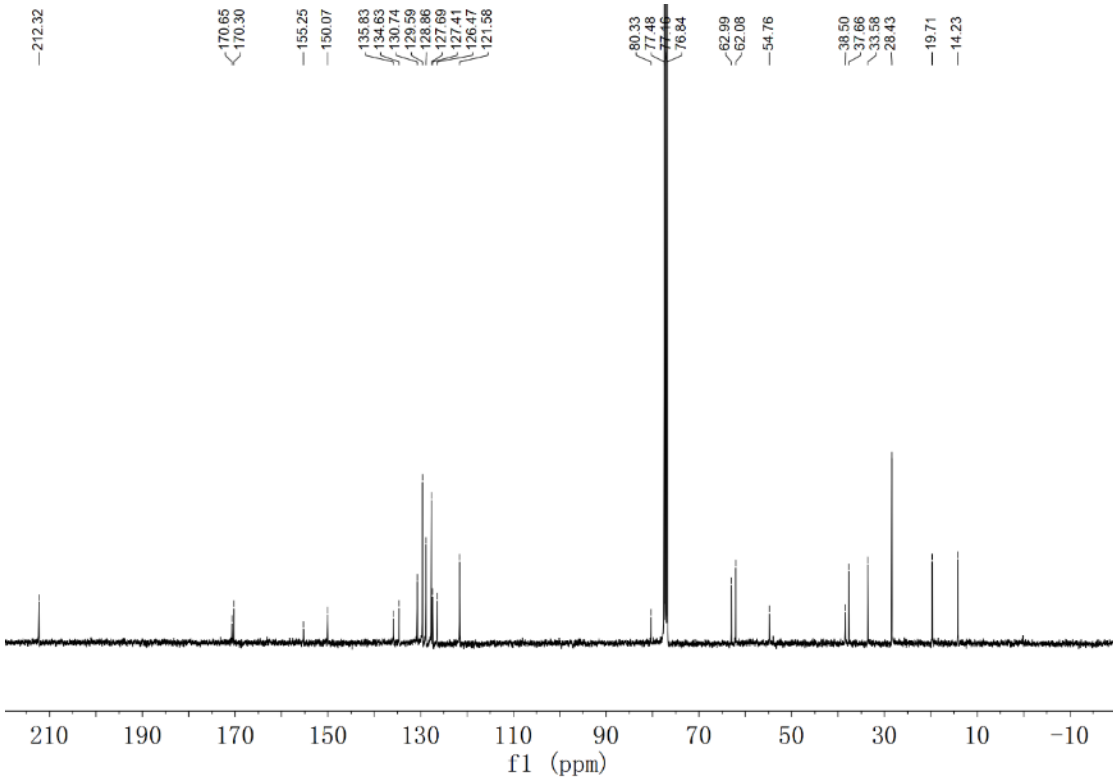

Supplementary Fig. 113. ^{13}C NMR spectra of compound **3al**. (100 MHz, 298K) in $CDCl_3$

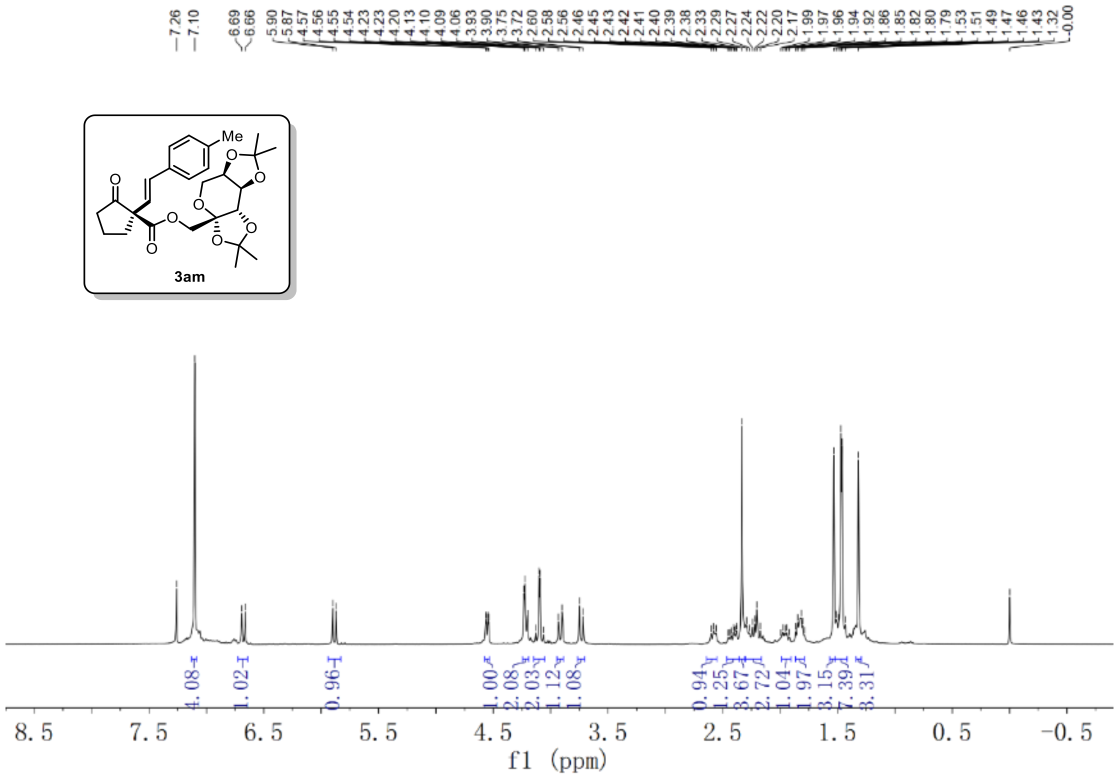

Supplementary Fig. 114. ^{1}H NMR spectra of compound **3am**. (400 MHz, 298K) in $CDCl_3$

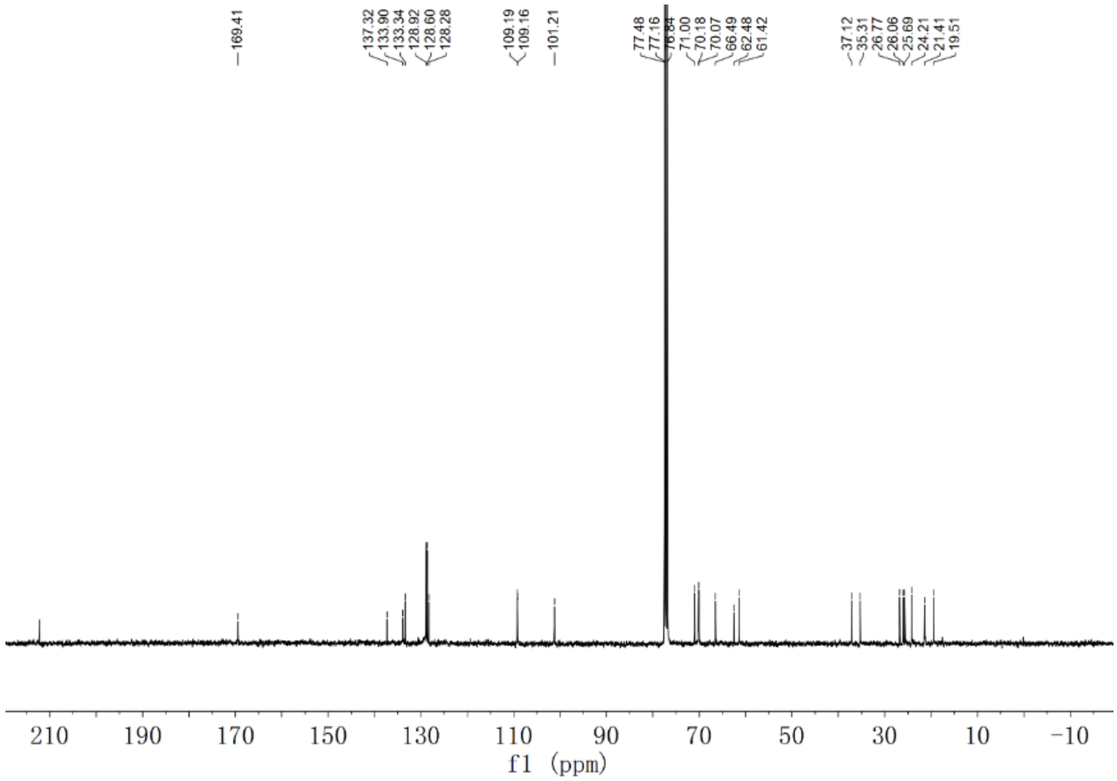

Supplementary Fig. 115. ^{13}C NMR spectra of compound **3am**. (100 MHz, 298K) in $CDCl_3$

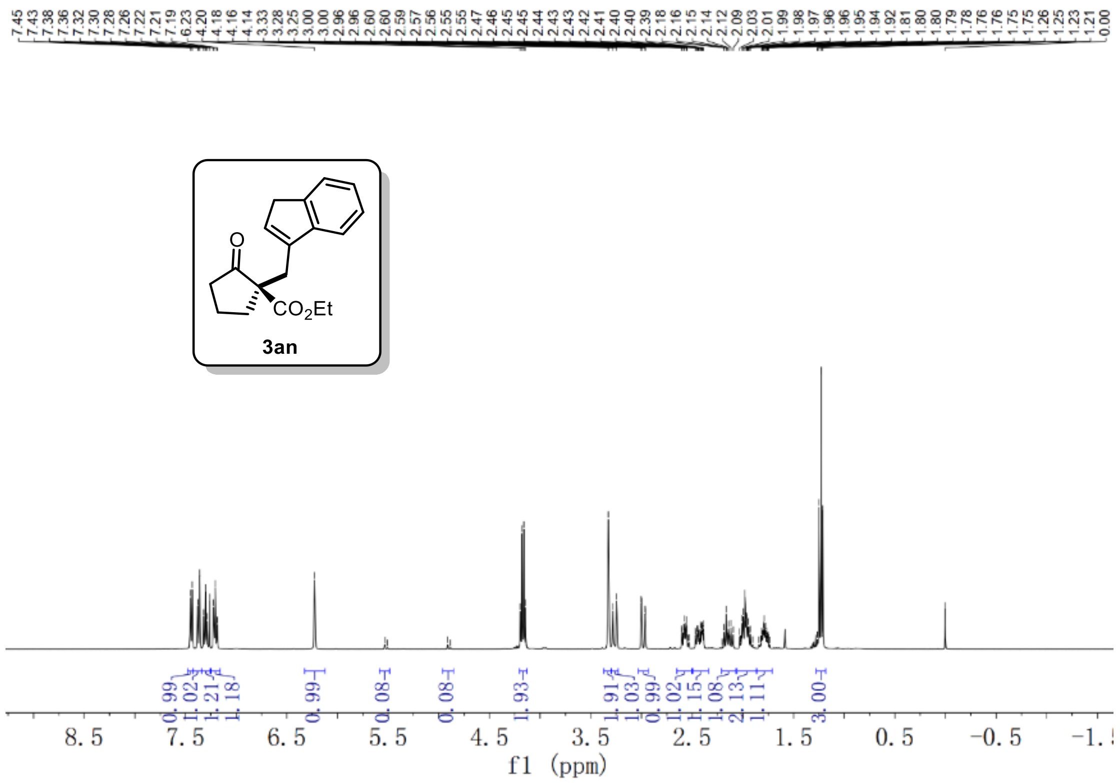

Supplementary Fig. 116. ^{1}H NMR spectra of compound **3an**. (400 MHz, 298K) in $CDCl_3$

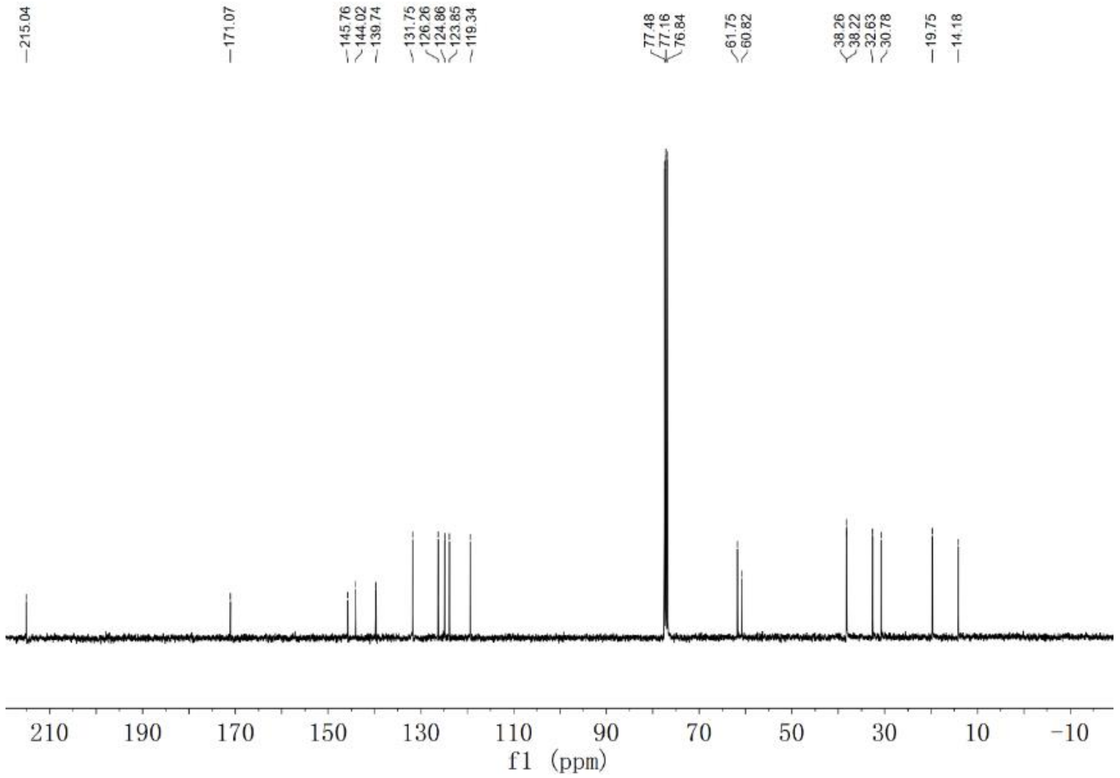

Supplementary Fig. 117. ^{13}C NMR spectra of compound **3an**. (100 MHz, 298K) in $CDCl_3$

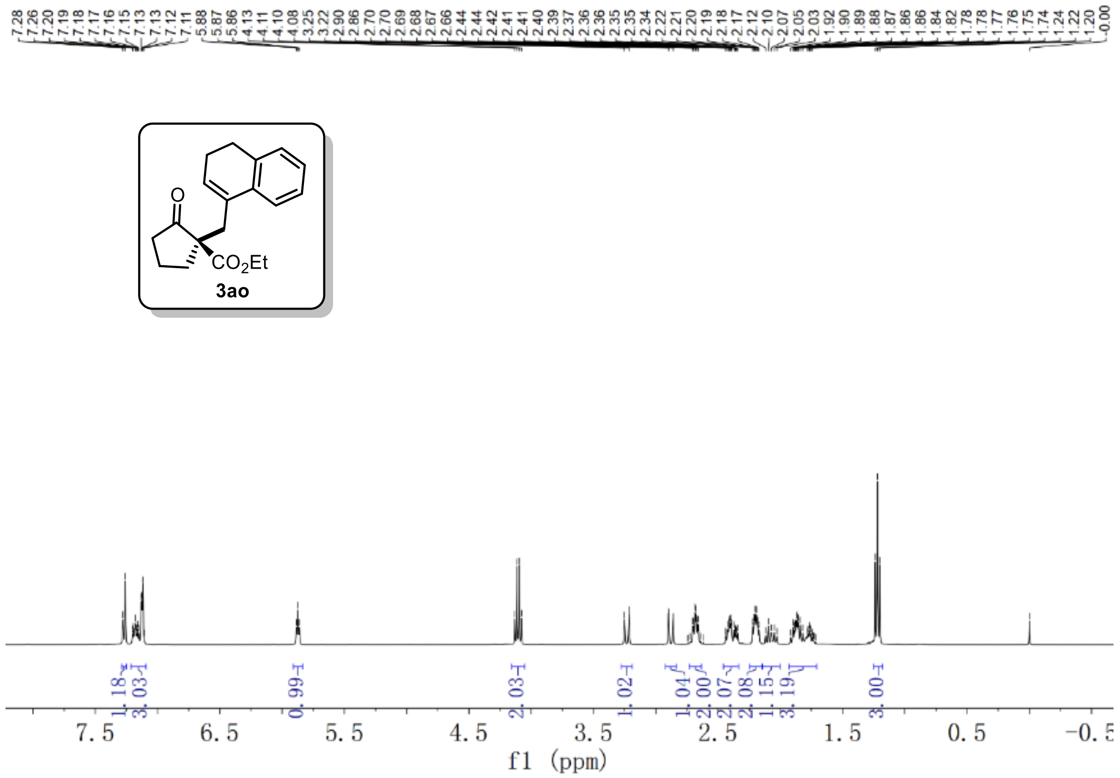

Supplementary Fig. 118. ^{1}H NMR spectra of compound **3ao**. (400 MHz, 298K) in $CDCl_3$

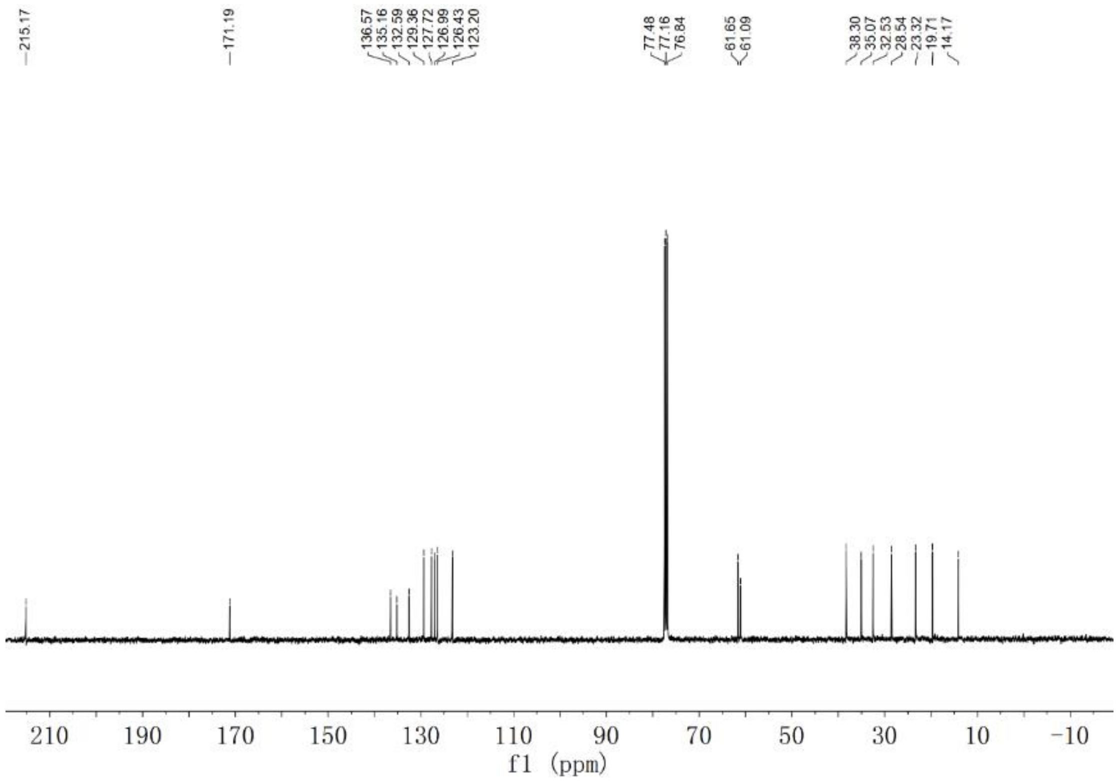

Supplementary Fig. 119. ^{13}C NMR spectra of compound **3ao**. (100 MHz, 298K) in $CDCl_3$

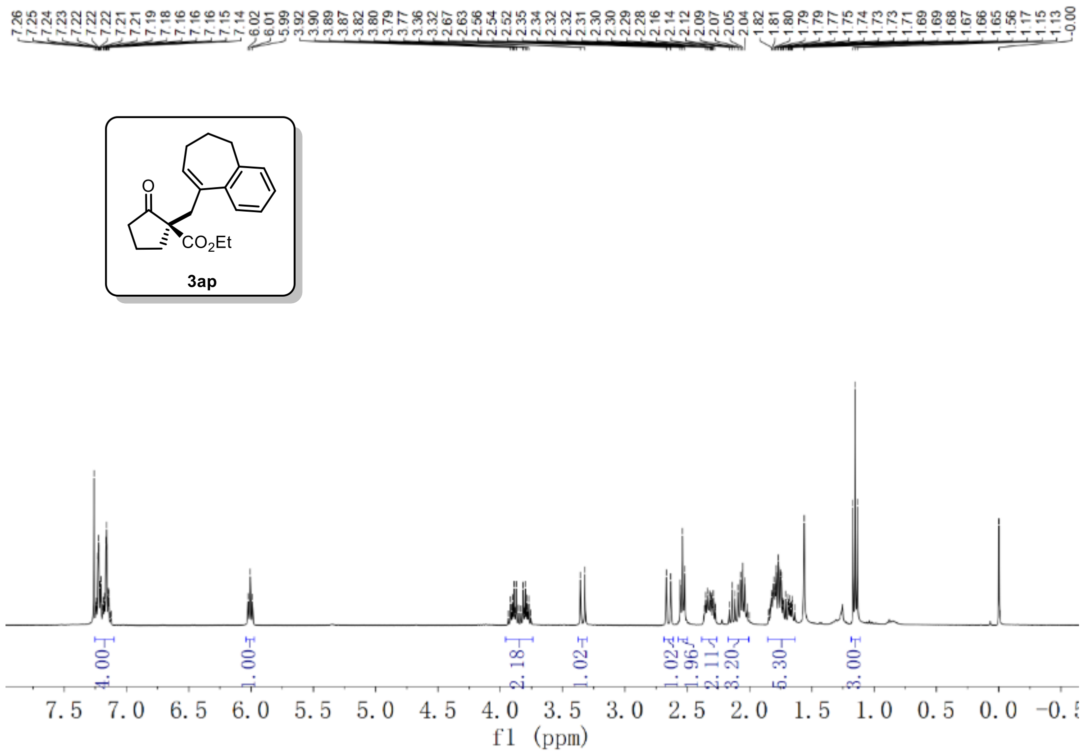

Supplementary Fig. 120. ^{1}H NMR spectra of compound **3ap**. (400 MHz, 298K) in $CDCl_3$

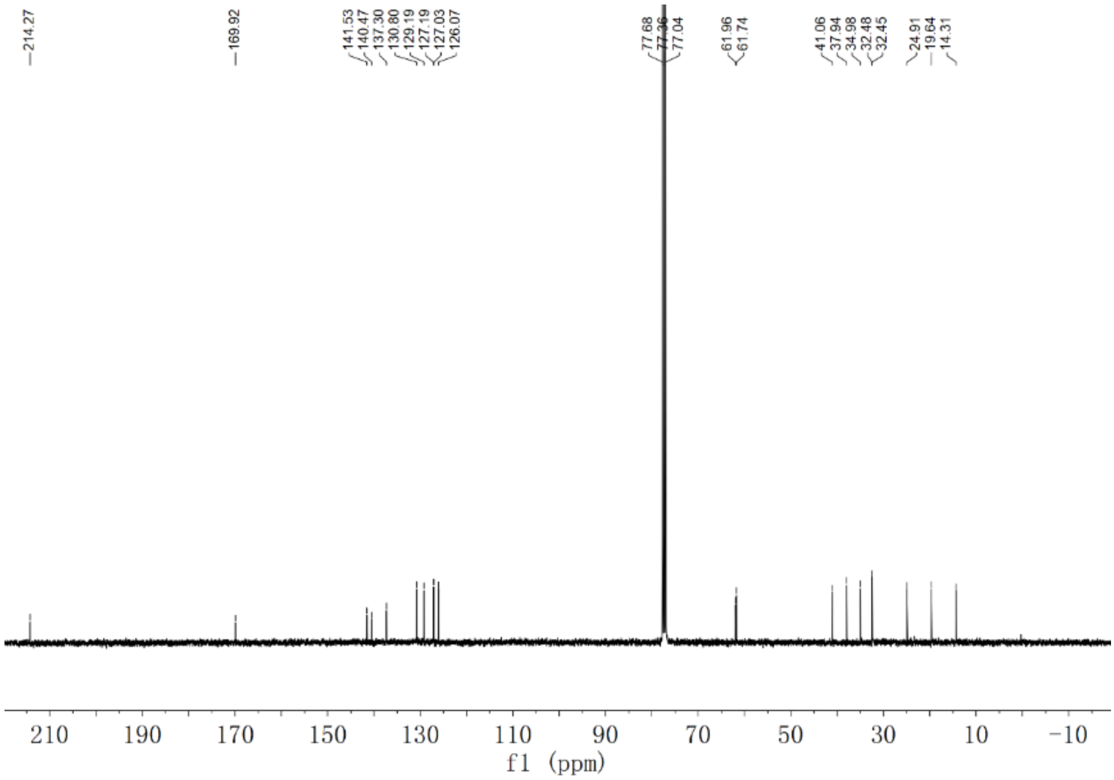

Supplementary Fig. 121. ^{13}C NMR spectra of compound **3ap**. (100 MHz, 298K) in $CDCl_3$

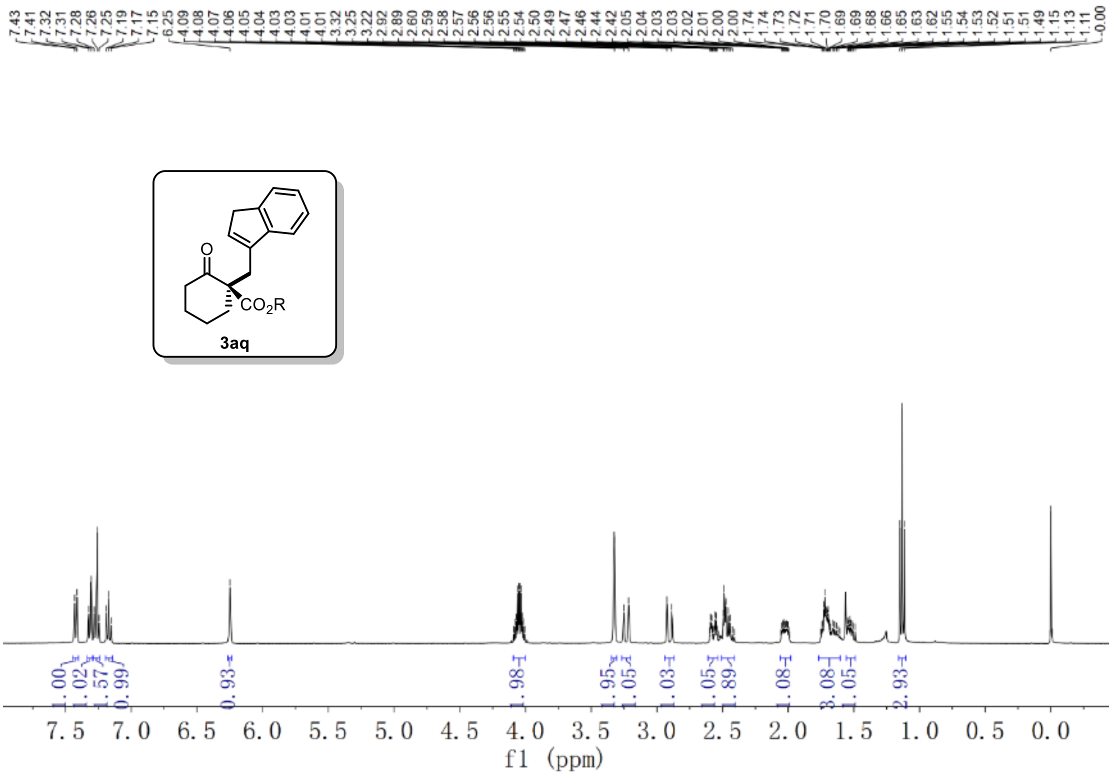

Supplementary Fig. 122. ^{1}H NMR spectra of compound **3aq**. (400 MHz, 298K) in $CDCl_3$

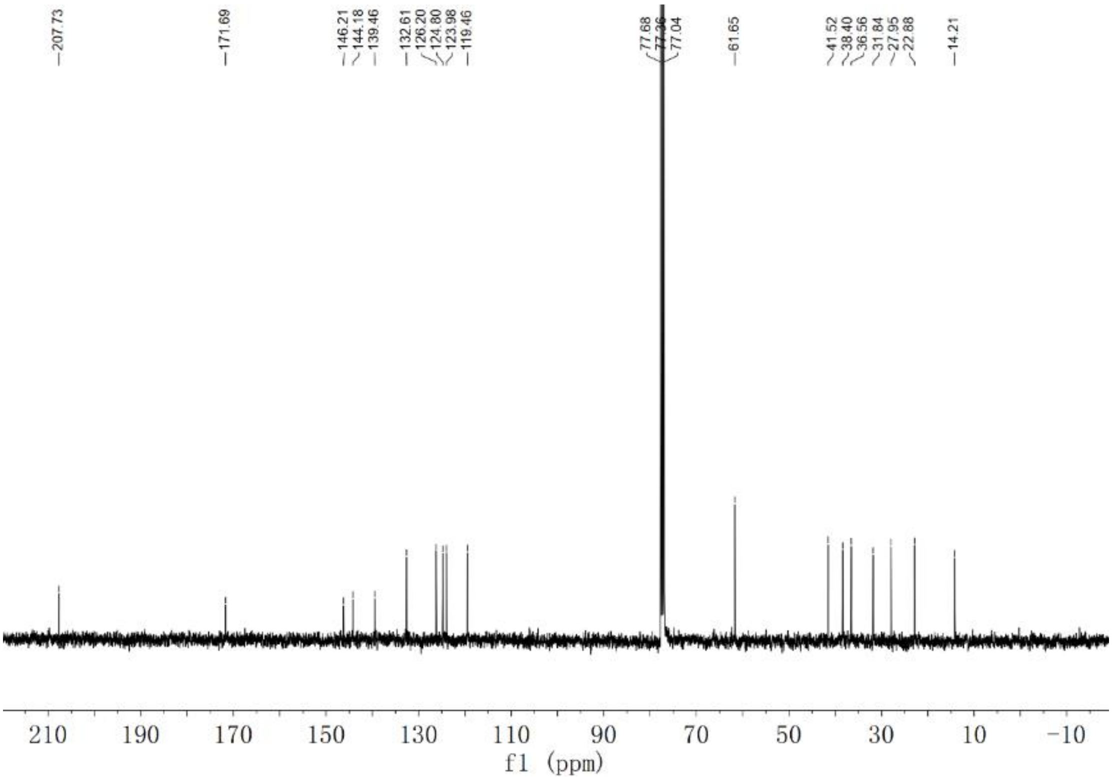

Supplementary Fig. 123. ^{13}C NMR spectra of compound **3aq**. (100 MHz, 298K) in $CDCl_3$

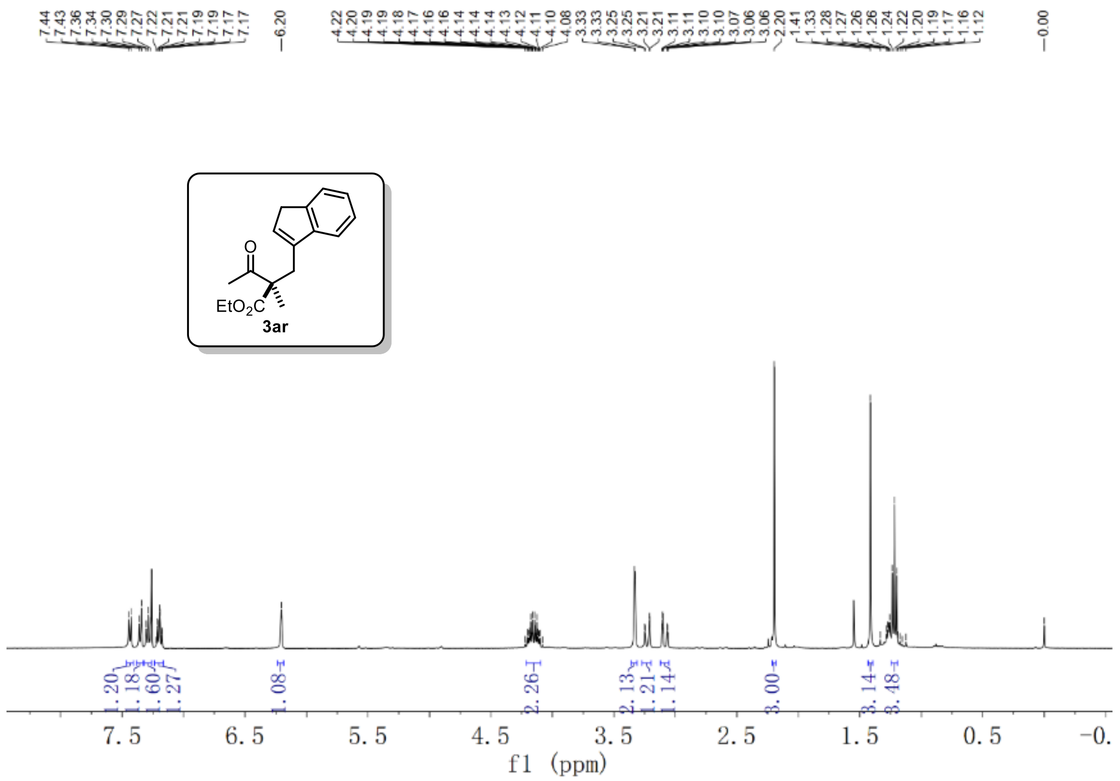

Supplementary Fig. 124. ^{1}H NMR spectra of compound **3ar**. (400 MHz, 298K) in $CDCl_3$

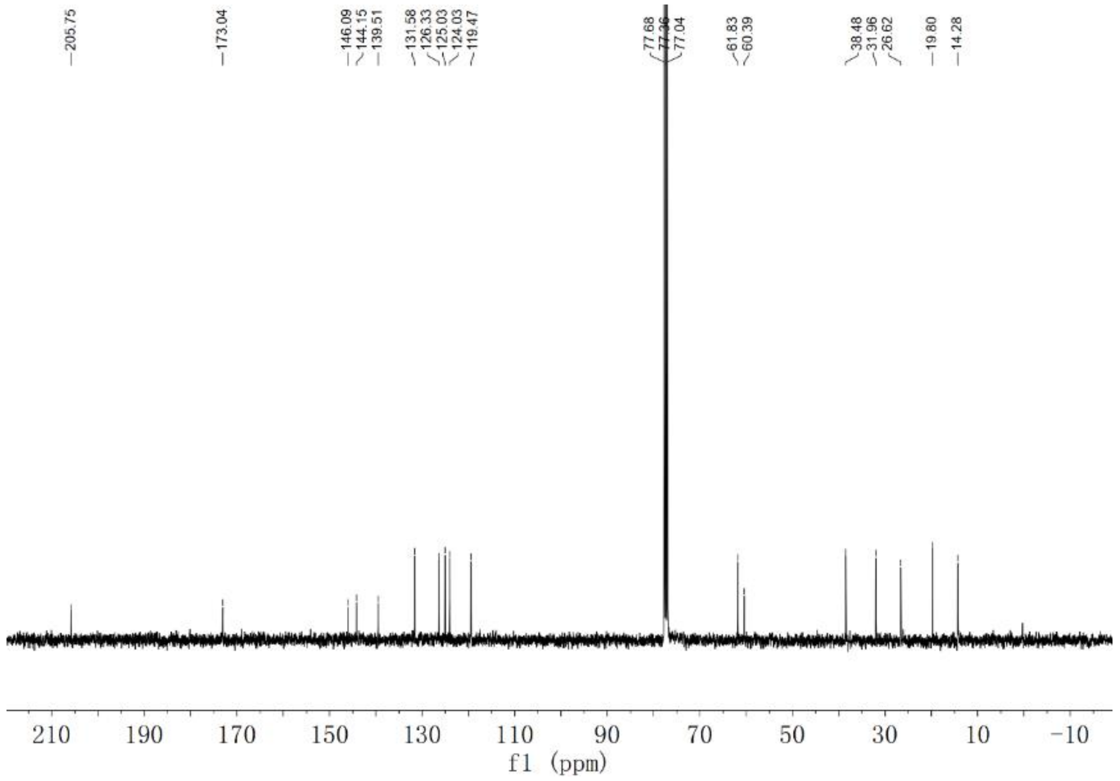

Supplementary Fig. 125. ^{13}C NMR spectra of compound **3ar**. (100 MHz, 298K) in $CDCl_3$

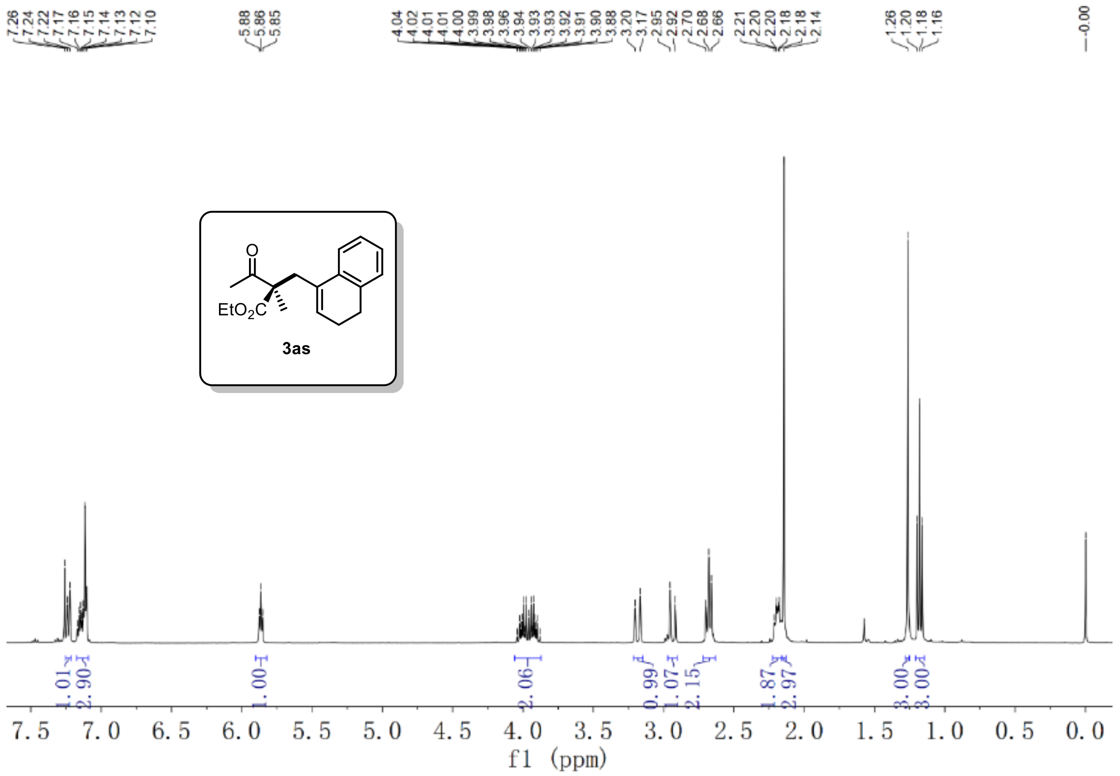

Supplementary Fig. 126. ^{1}H NMR spectra of compound **3as**. (400 MHz, 298K) in $CDCl_3$

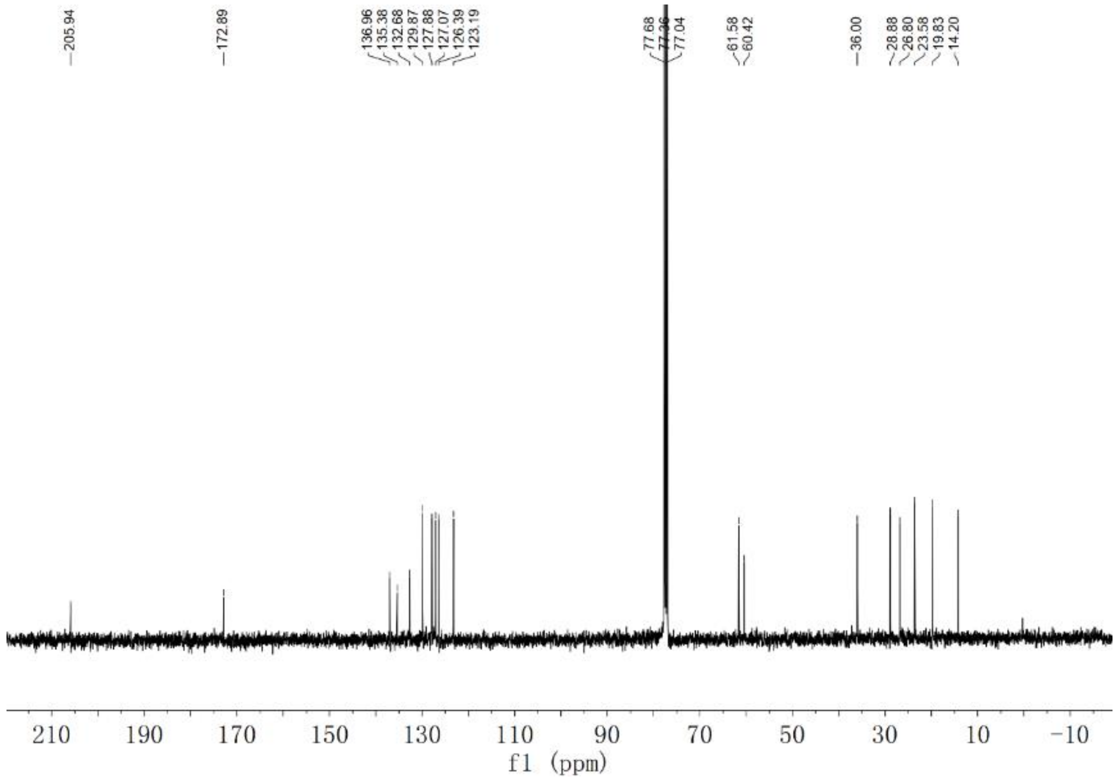

Supplementary Fig. 127. ^{13}C NMR spectra of compound **3as**. (100 MHz, 298K) in $CDCl_3$

6. HPLC spectra

HPLC (ChiralPak IC, 2% *i*-PrOH in hexanes, 1.0 mL/min, 254 nm)

<Chromatogram>

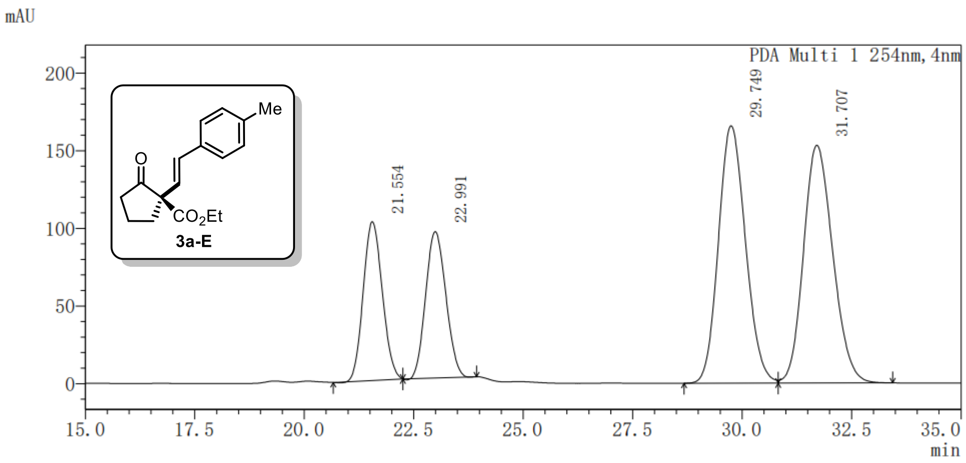

<Peak Results>

PDA Ch1 254nm

Index	Time/min	Height/mAU	Quantity/Area	Area %/%
1	21.554	102225	3100553	15.198
2	22.991	94229	3027319	14.839
3	29.749	165755	7134452	34.971
4	31.707	153073	7139018	34.993

<Chromatogram>

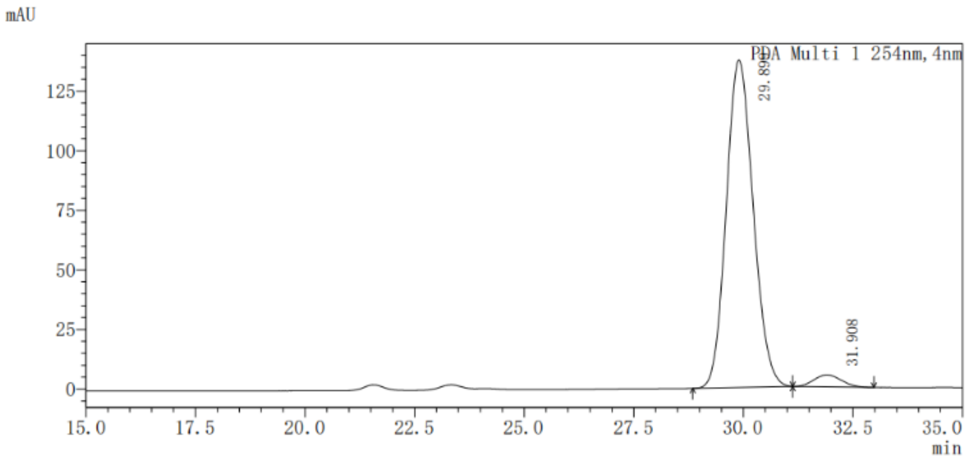

<Peak Results>

PDA Ch1 254nm

Index	Time/min	Height/mAU	Quantity/Area	Area %/%
1	29.899	137349	5875526	96.426
2	31.908	5021	217804	3.574

Supplementary Fig. 128. HPLC chromatograms of compound **3a-*E*.**

HPLC (ChiralPak IC, 2% *i*-PrOH in hexanes, 1.0 mL/min, 248 nm)

<Chromatogram>

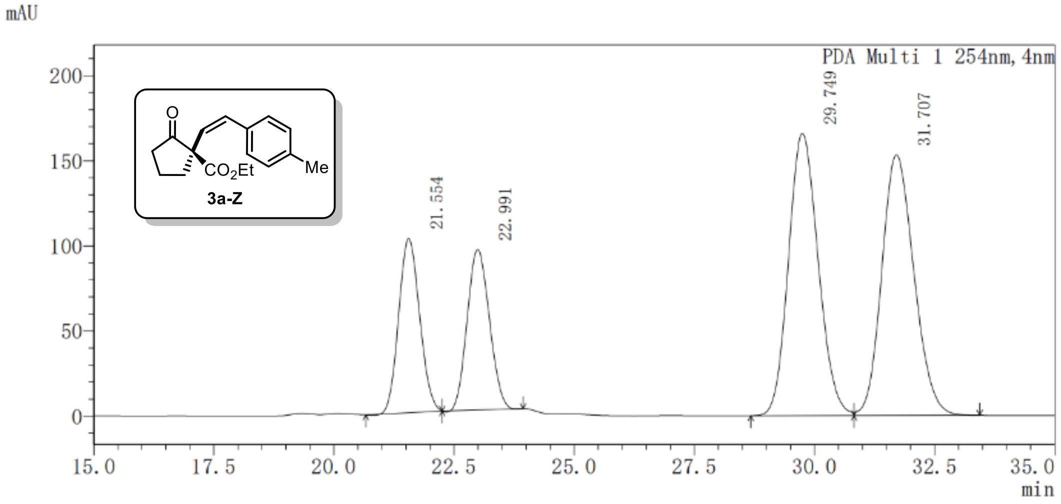

<Peak Results>

PDA Ch1 254nm

Index	Time/min	Height/mAU	Quantity/Area	Area %/%
1	21.554	102225	3100553	15.198
2	22.991	94229	3027319	14.839
3	29.749	165755	7134452	34.971
4	31.707	153073	7139018	34.993

<Chromatogram>

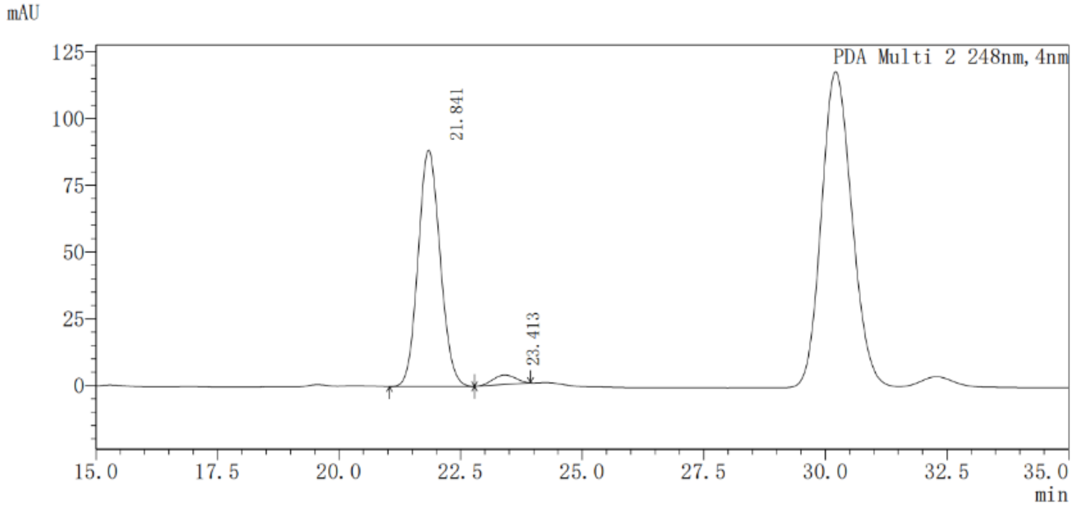

<Peak Results>

PDA Ch2 248nm

Index	Time/min	Height/mAU	Quantity/Area	Area %/%
1	21.841	88554	2798721	96.024
2	23.413	3535	115891	3.976

Supplementary Fig. 129. HPLC chromatograms of compound **3a-*Z*.**

HPLC (ChiralPak OJ-H, 5% *i*-PrOH in hexanes, 1.0 mL/min, 254 nm)

<Chromatogram>

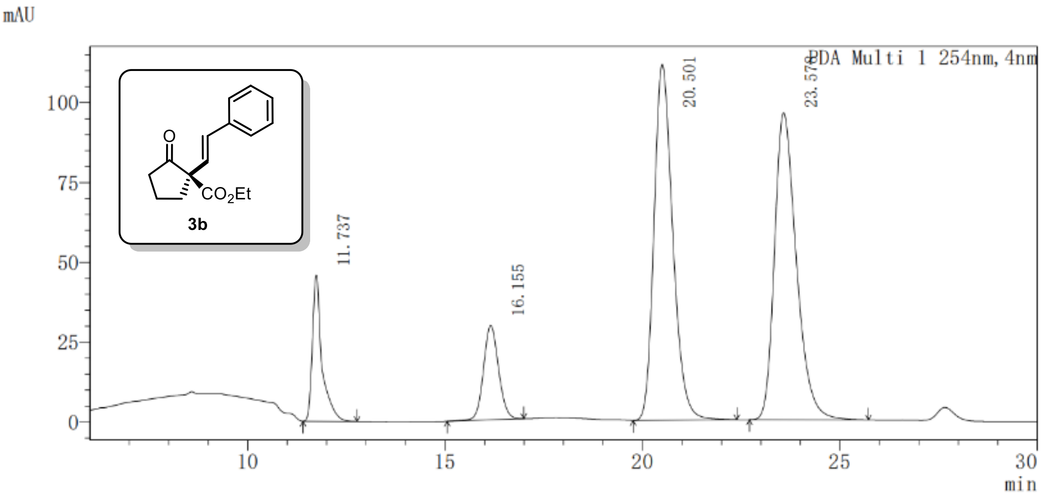

<Peak Results>

PDA Ch1 254nm

Index	Time/min	Height/mAU	Quantity/Area	Area %/%
1	11.737	45657	772362	8.630
2	16.155	29517	786466	8.788
3	20.501	111444	3679363	41.112
4	23.578	96172	3711509	41.471

<Chromatogram>

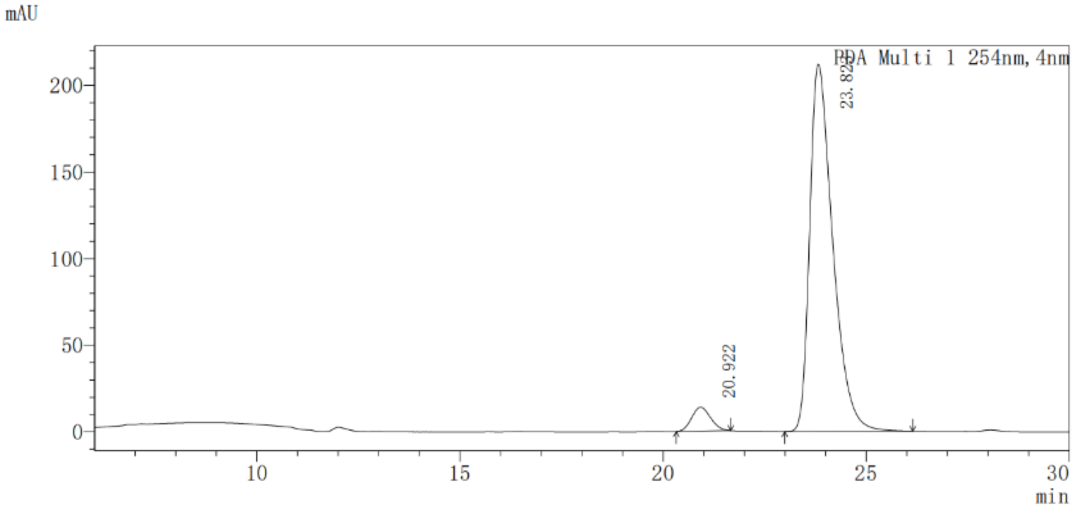

<Peak Results>

PDA Ch1 254nm

Index	Time/min	Height/mAU	Quantity/Area	Area %/%
1	20.922	13850	450369	5.107
2	23.823	211901	8369145	94.893

Supplementary Fig. 130. HPLC chromatograms of compound **3b.**

HPLC (ChiralPak AD-H, 2% *i*-PrOH in hexanes, 1.0 mL/min, 254 nm)

<Chromatogram>

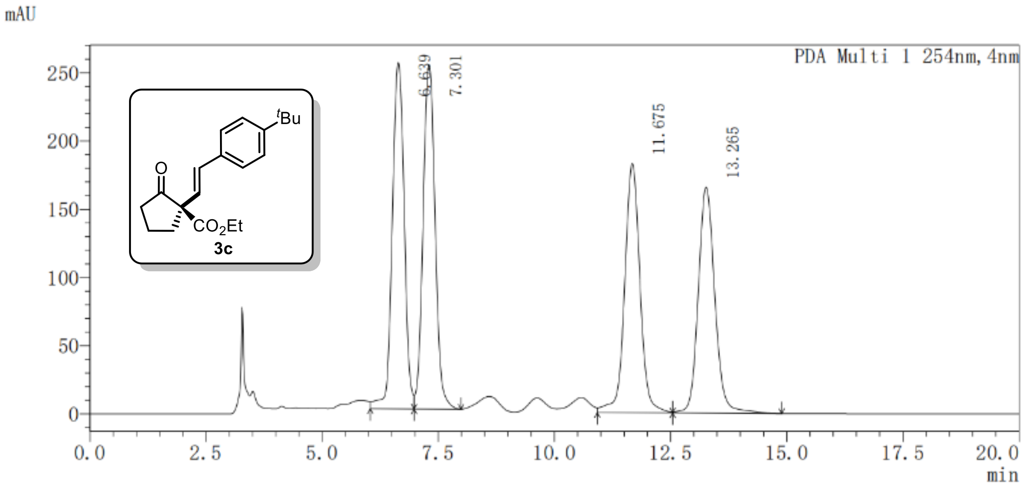

<Peak Results>

PDA Ch1 254nm

Index	Time/min	Height/mAU	Quantity/Area	Area %/%
1	6. 639	253816	4538275	26. 081
2	7. 301	252306	4549090	26. 143
3	11. 675	182691	4203237	24. 156
4	13. 265	165500	4109888	23. 619

<Chromatogram>

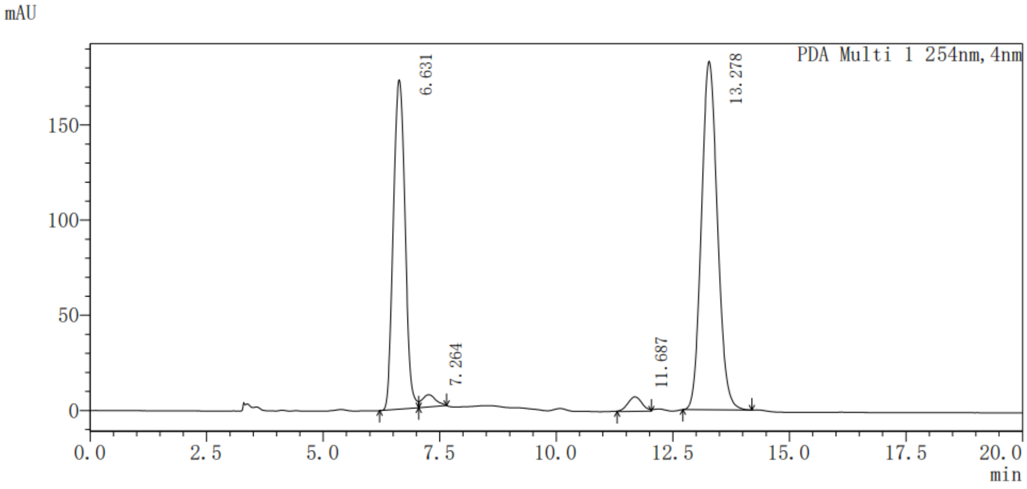

<Peak Results>

PDA Ch1 254nm

Index	Time/min	Height/mAU	Quantity/Area	Area %/%
1	6. 631	173056	3029422	39. 360
2	7. 264	6387	130567	1. 696
3	11. 687	7630	162333	2. 109
4	13. 278	183065	4374292	56. 834

Supplementary Fig. 131. HPLC chromatograms of compound **3c.**

HPLC (ChiralPak AD-H, 2% *i*-PrOH in hexanes, 1.0 mL/min, 254 nm)

<Chromatogram>

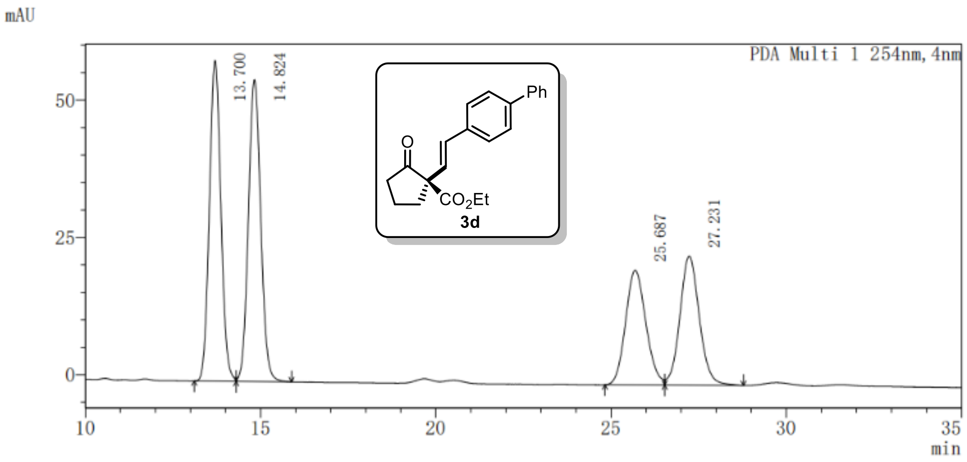

<Peak Results>

PDA Ch1 254nm

Index	Time/min	Height/mAU	Quantity/Area	Area %/%
1	13.700	58344	1334503	30.133
2	14.824	54941	1345462	30.380
3	25.687	20880	841141	18.993
4	27.231	23499	907623	20.494

<Chromatogram>

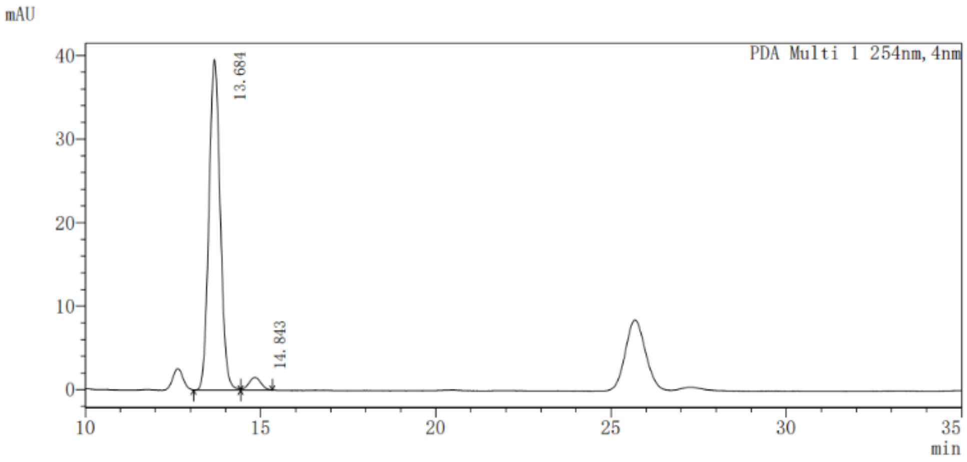

<Peak Results>

PDA Ch1 254nm

Index	Time/min	Height/mAU	Quantity/Area	Area %/%
1	13.684	39545	886696	96.095
2	14.843	1514	36031	3.905

Supplementary Fig. 132. HPLC chromatograms of compound **3d.**

HPLC (ChiralPak OD-H, 5% *i*-PrOH in hexanes, 1.0 mL/min, 287 nm)

<Chromatogram>

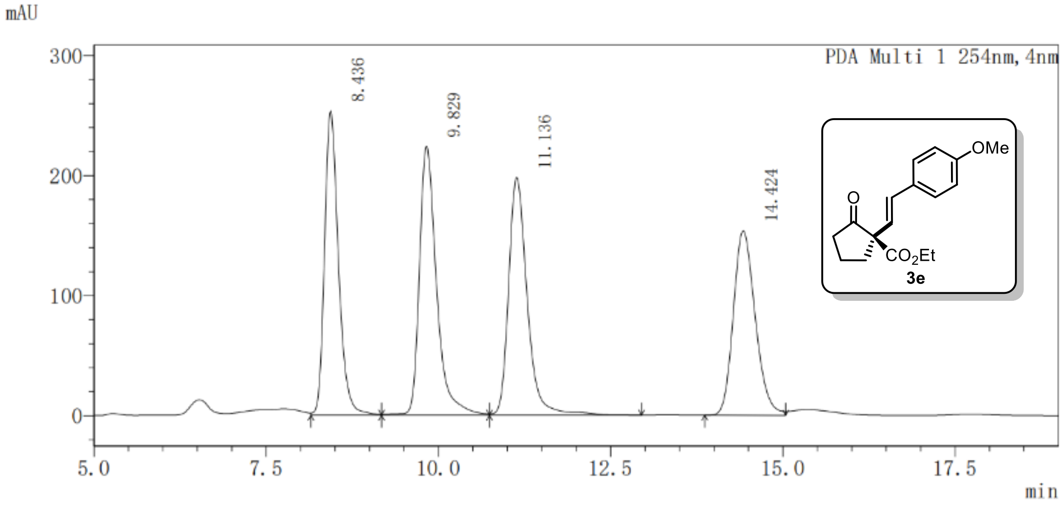

<Peak Results>

PDA Ch1 254nm

Index	Time/min	Height/mAU	Quantity/Area	Area %/%
1	8.436	253281	3554785	24.427
2	9.829	223947	3881219	26.670
3	11.136	198049	3716095	25.536
4	14.424	153615	3400452	23.367

<Chromatogram>

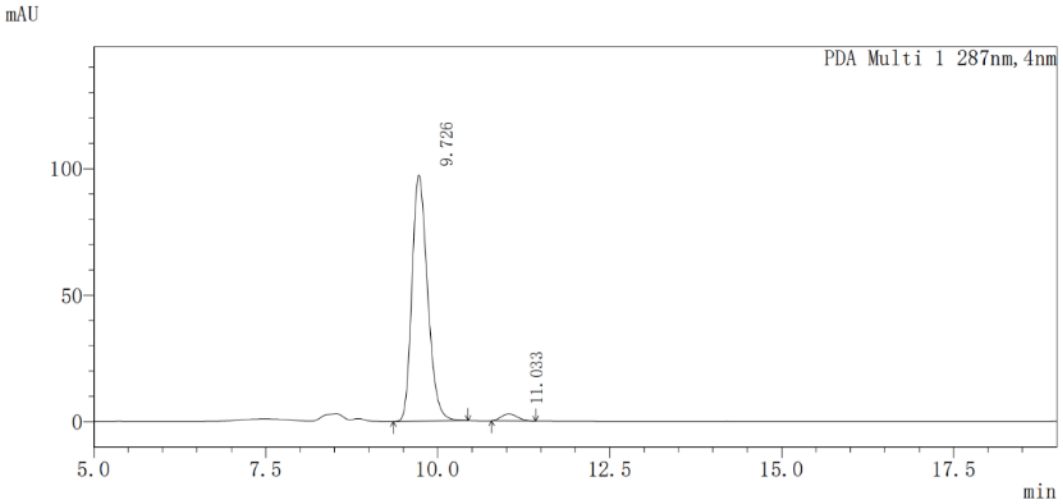

<Peak Results>

PDA Ch1 287nm

Index	Time/min	Height/mAU	Quantity/Area	Area %/%
1	9.726	97174	1536037	97.141
2	11.033	2764	45212	2.859

Supplementary Fig. 133. HPLC chromatograms of compound **3e.**

HPLC (ChiralPak AS-H, 5% *i*-PrOH in hexanes, 1.0 mL/min, 267 nm)

<Chromatogram>

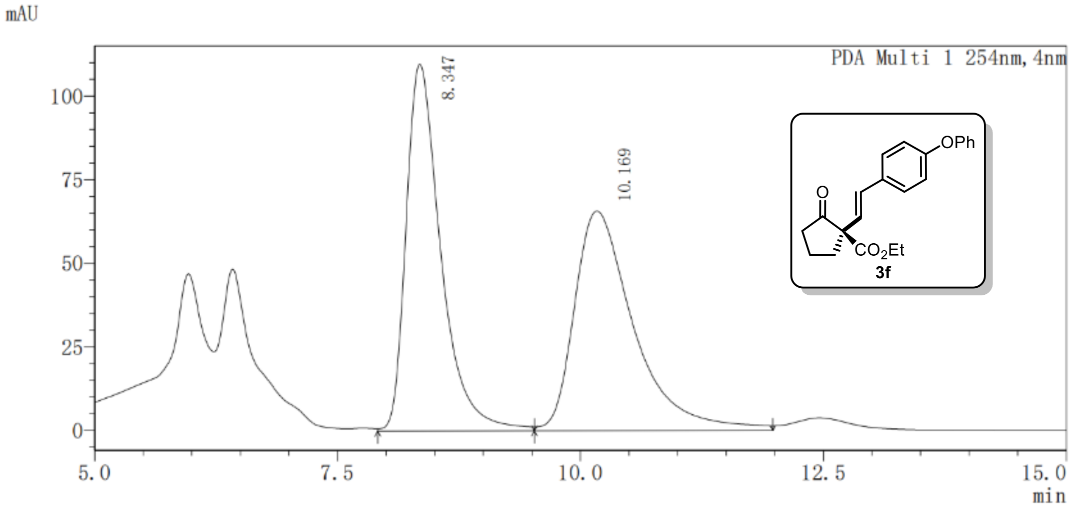

<Peak Results>

PDA Ch1 254nm

Index	Time/min	Height/mAU	Quantity/Area	Area %/%
1	8.347	109647	2739022	49.399
2	10.169	65742	2805648	50.601

<Chromatogram>

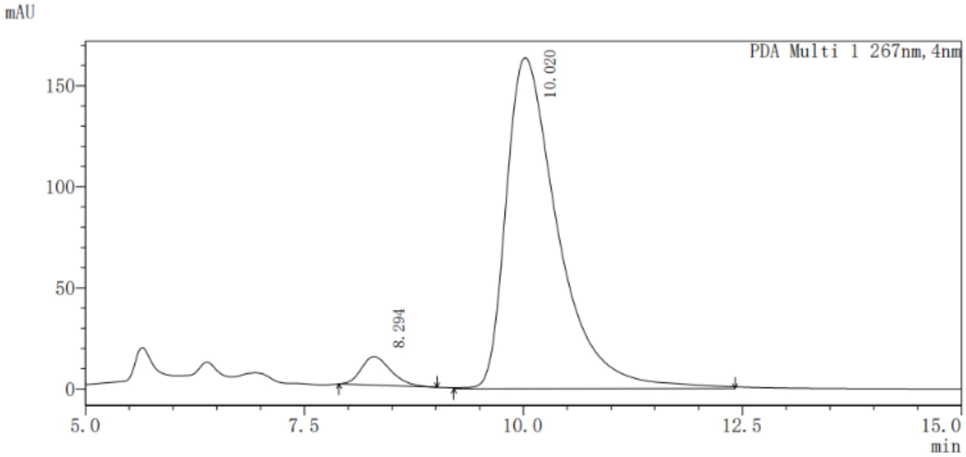

<Peak Results>

PDA Ch1 267nm

Index	Time/min	Height/mAU	Quantity/Area	Area %/%
1	8.294	14104	331473	4.681
2	10.020	163628	6749067	95.319

Supplementary Fig. 134. HPLC chromatograms of compound **3f.**

HPLC (ChiralPak OD-H, 5% *i*-PrOH in hexanes, 1.0 mL/min, 254 nm)

<Chromatogram>

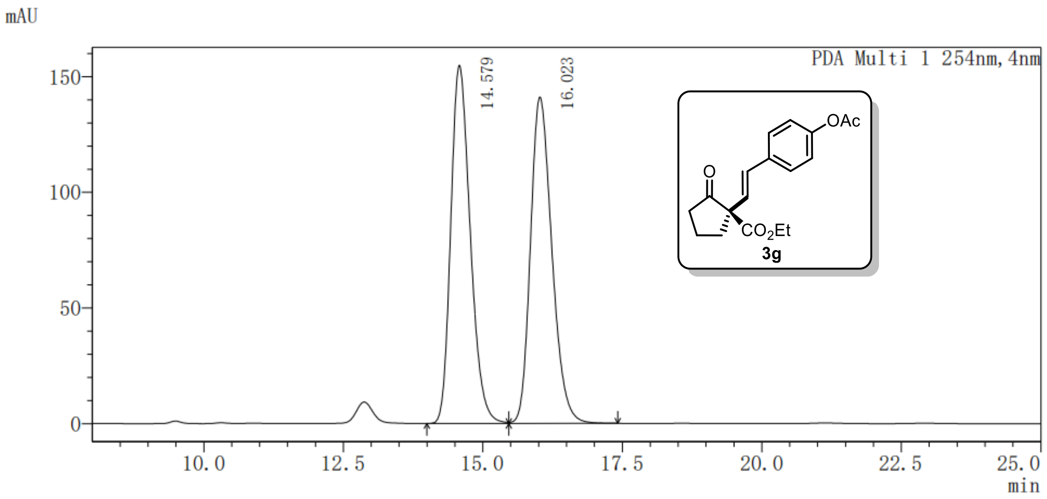

<Peak Results>

PDA Ch1 254nm

Index	Time/min	Height/mAU	Quantity/Area	Area %/%
1	14.579	154767	3760103	49.978
2	16.023	141072	3763365	50.022

<Chromatogram>

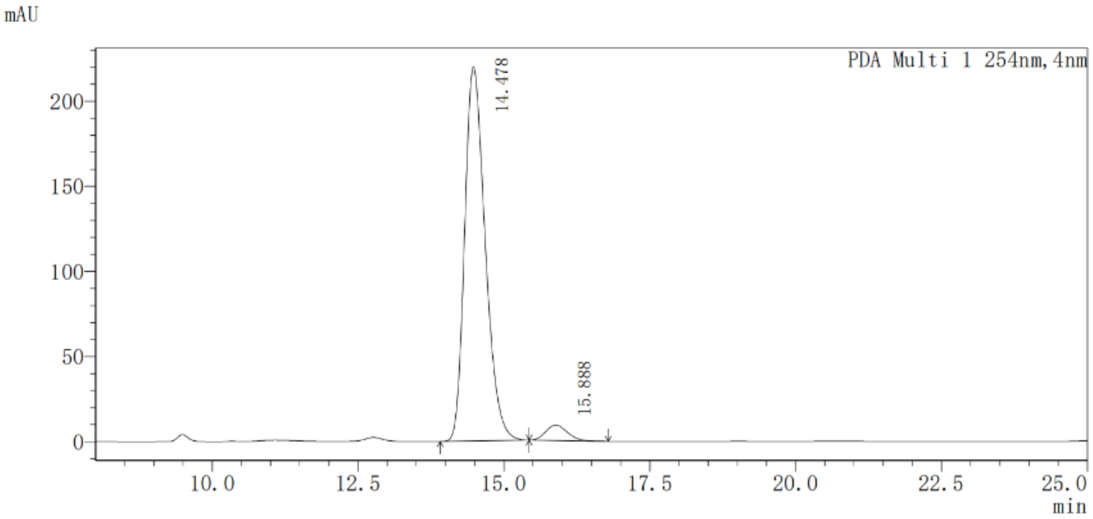

<Peak Results>

PDA Ch1 254nm

Index	Time/min	Height/mAU	Quantity/Area	Area %/%
1	14.478	219778	5382374	95.821
2	15.888	9118	234730	4.179

Supplementary Fig. 135. HPLC chromatograms of compound **3g.**

HPLC (ChiralPak OJ-H, 5% *i*-PrOH in hexanes, 1.0 mL/min, 254 nm)

<Chromatogram>

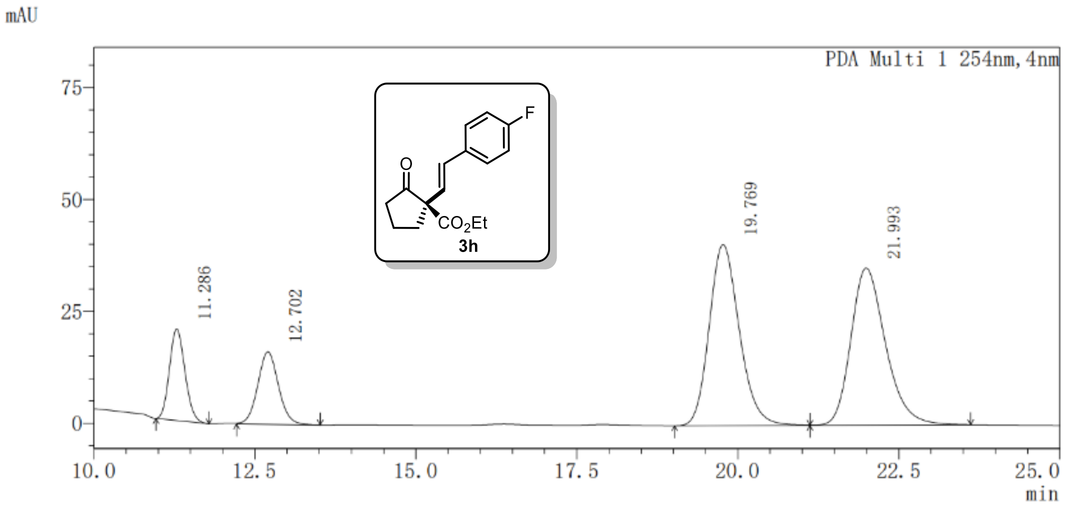

<Peak Results>

PDA Ch1 254nm

Index	Time/min	Height/mAU	Quantity/Area	Area %/%
1	11.286	20461	342375	10.267
2	12.702	16228	363896	10.912
3	19.769	40431	1315852	39.458
4	21.993	35068	1312671	39.363

<Chromatogram>

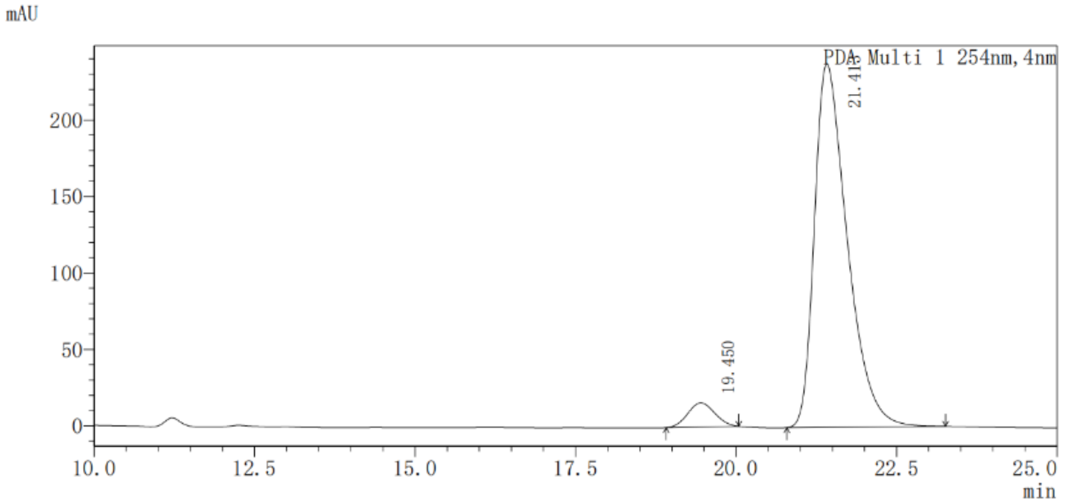

<Peak Results>

PDA Ch1 254nm

Index	Time/min	Height/mAU	Quantity/Area	Area %/%
1	19.450	15788	463134	5.197
2	21.413	237535	8448349	94.803

Supplementary Fig. 136. HPLC chromatograms of compound **3h.**

HPLC (ChiralPak OJ-H, 5% *i*-PrOH in hexanes, 1.0 mL/min, 260 nm)

<Chromatogram>

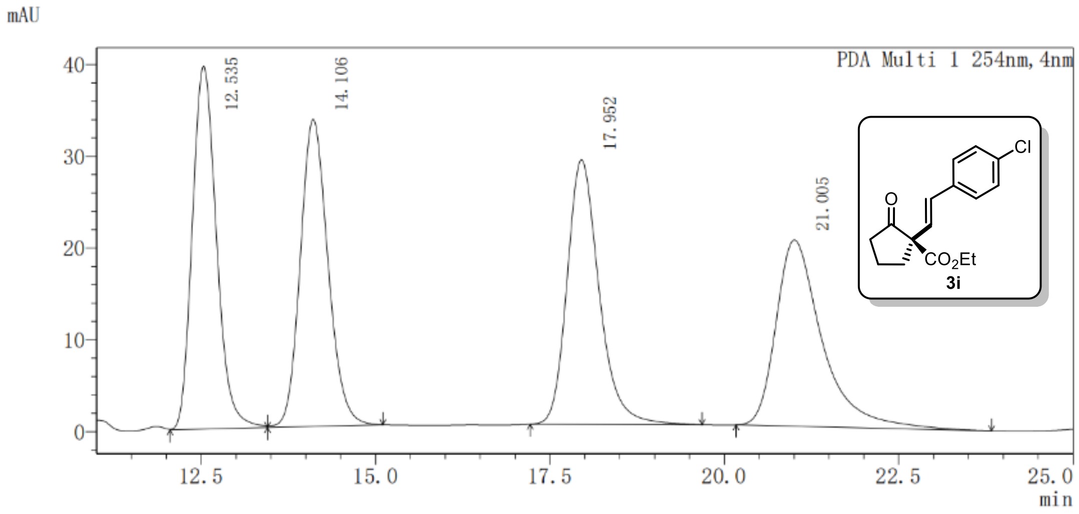

<Peak Results>

PDA Ch1 254nm

Index	Time/min	Height/mAU	Quantity/Area	Area %/%
1	12.535	39544	951686	25.489
2	14.106	33454	924609	24.763
3	17.952	28834	922434	24.705
4	21.005	20303	935031	25.043

<Chromatogram>

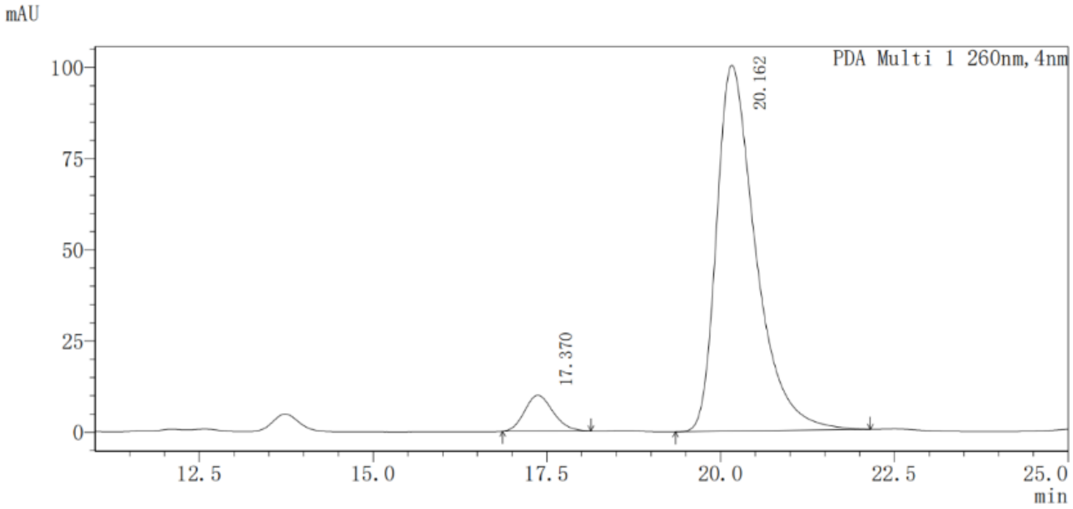

<Peak Results>

PDA Ch1 260nm

Index	Time/min	Height/mAU	Quantity/Area	Area %/%
1	17.370	9812	280088	6.649
2	20.162	100329	3932137	93.351

Supplementary Fig. 137. HPLC chromatograms of compound **3i.**

HPLC (ChiralPak IC, 2% *i*-PrOH in hexanes, 1.0 mL/min, 254 nm)

〈Chromatogram〉

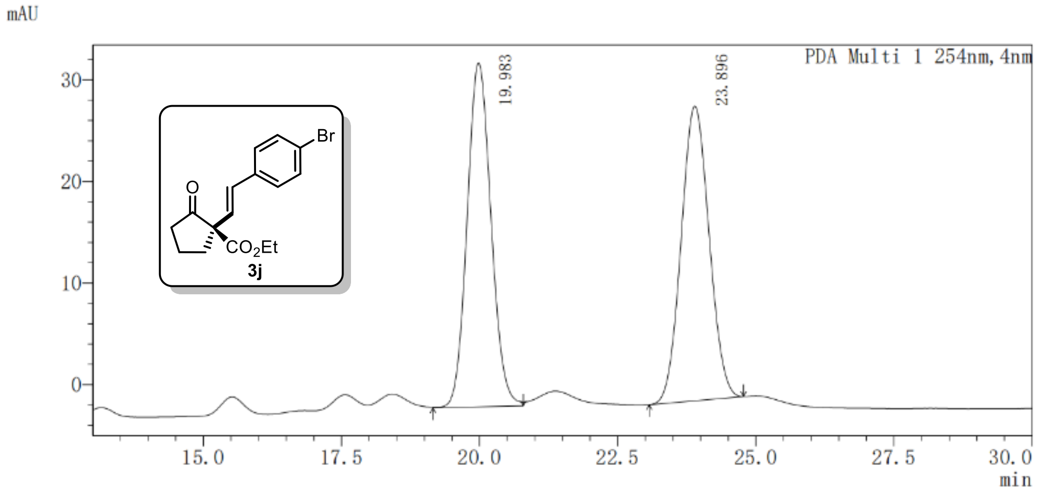

〈Peak Results〉

PDA Ch1 254nm

Index	Time/min	Height/mAU	Quantity/Area	Area %/%
1	19.983	33850	1017906	49.919
2	23.896	29005	1021192	50.081

〈Chromatogram〉

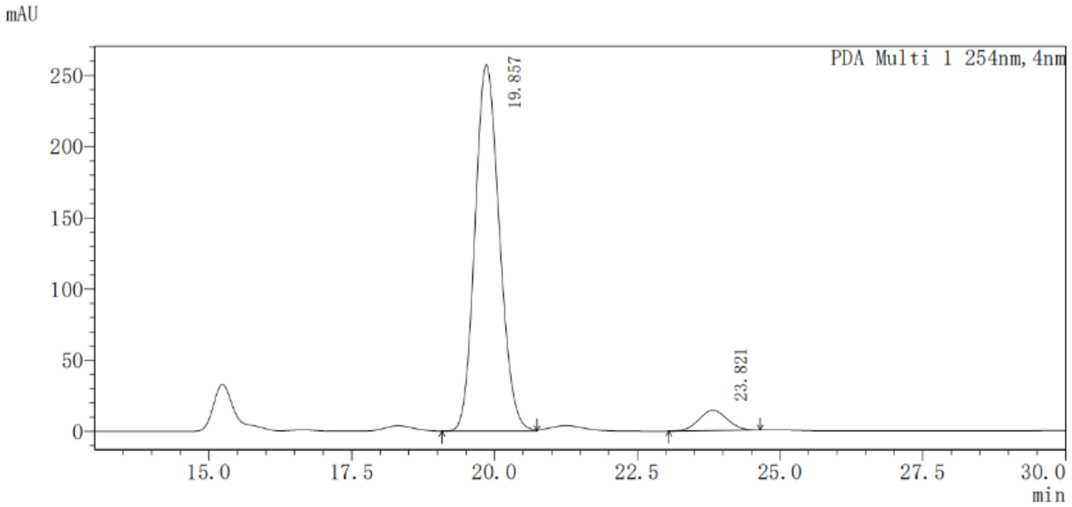

〈Peak Results〉

PDA Ch1 254nm

Index	Time/min	Height/mAU	Quantity/Area	Area %/%
1	19.857	257410	7666421	93.946
2	23.821	14313	493999	6.054

Supplementary Fig. 138. HPLC chromatograms of compound **3j.**

HPLC (ChiralPak IC, 2% *i*-PrOH in hexanes, 1.0 mL/min, 254 nm)

<Chromatogram>

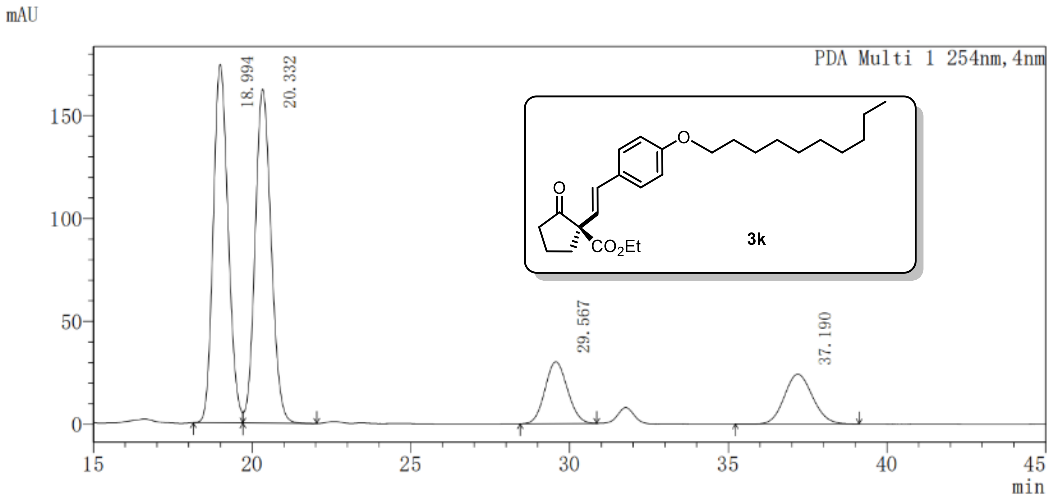

<Peak Results>

PDA Ch1 254nm

Index	Time/min	Height/mAU	Quantity/Area	Area %/%
1	18. 994	174205	5680356	39. 579
2	20. 332	162342	5700002	39. 716
3	29. 567	30033	1478470	10. 302
4	37. 190	24229	1493082	10. 403

<Chromatogram>

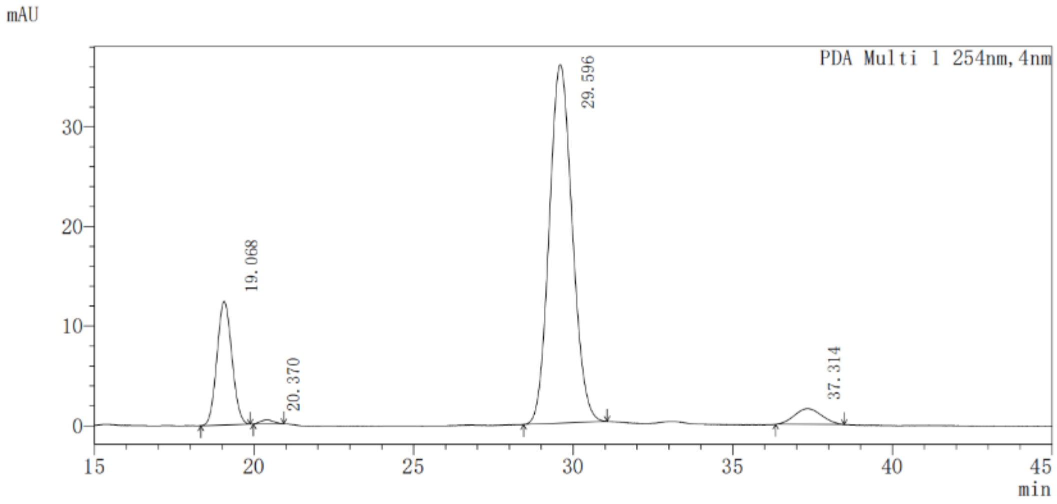

<Peak Results>

PDA Ch1 254nm

Index	Time/min	Height/mAU	Quantity/Area	Area %/%
1	19. 068	12410	404294	17. 456
2	20. 370	393	11063	0. 478
3	29. 596	35993	1806550	78. 002
4	37. 314	1586	94125	4. 064

Supplementary Fig. 139. HPLC chromatograms of compound **3k.**

HPLC (ChiralPak OJ-H, 5% *i*-PrOH in hexanes, 1.0 mL/min, 254 nm)

<Chromatogram>

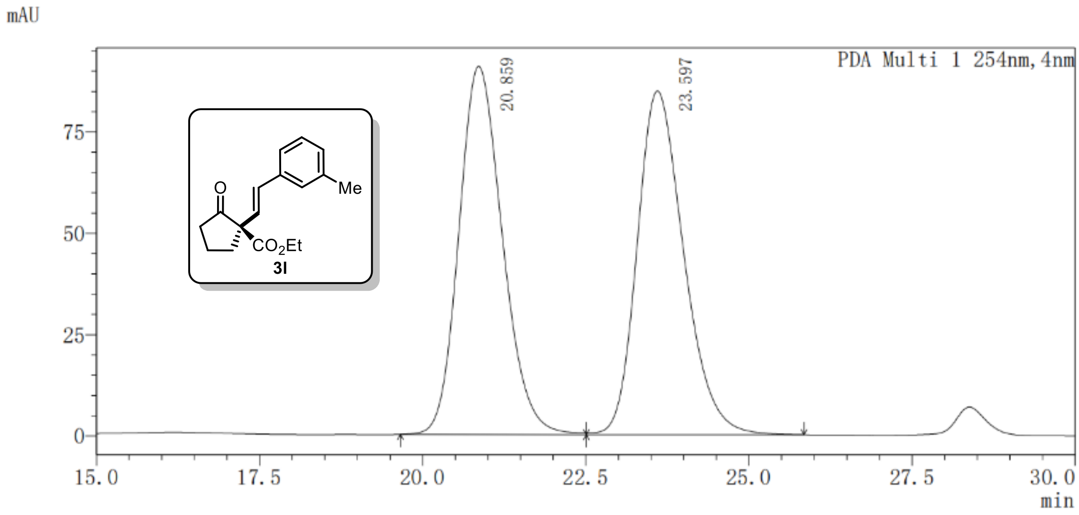

<Peak Results>

PDA Ch1 254nm

Index	Time/min	Height/mAU	Quantity/Area	Area %/%
1	20.859	90789	4214014	49.993
2	23.597	84771	4215165	50.007

<Chromatogram>

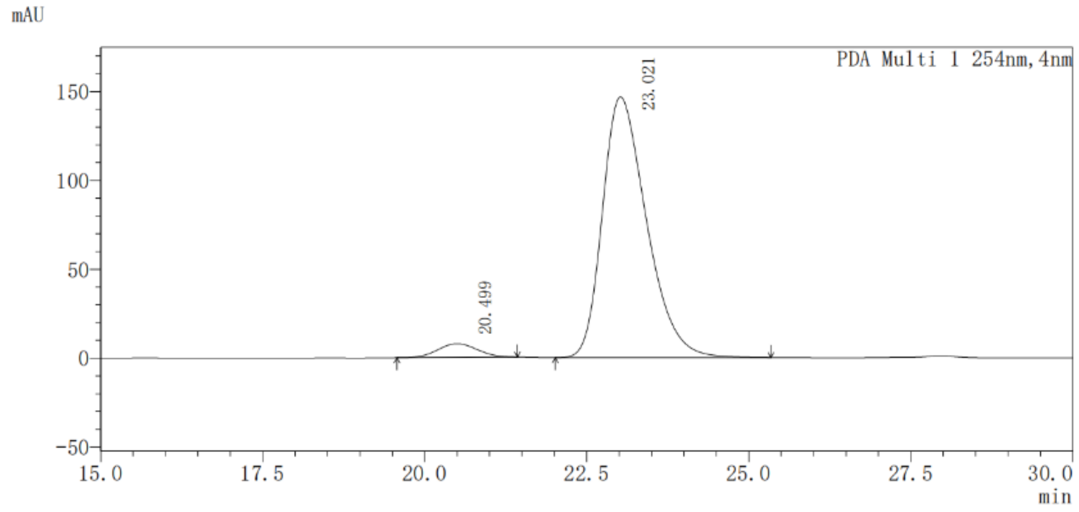

<Peak Results>

PDA Ch1 254nm

Index	Time/min	Height/mAU	Quantity/Area	Area %/%
1	20.499	7736	325369	4.478
2	23.021	146696	6941253	95.522

Supplementary Fig. 140. HPLC chromatograms of compound **3l.**

HPLC (ChiralPak OD-H, 2% *i*-PrOH in hexanes, 1.0 mL/min, 250 nm)

<Chromatogram>

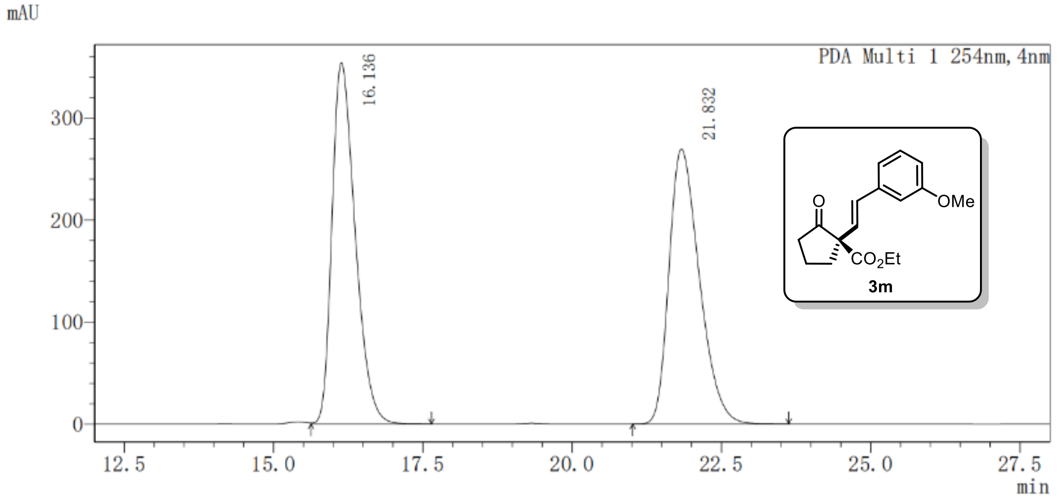

<Peak Results>

PDA Ch1 254nm

Index	Time/min	Height/mAU	Quantity/Area	Area %/%
1	16.136	353887	9561252	50.038
2	21.832	269350	9546686	49.962

<Chromatogram>

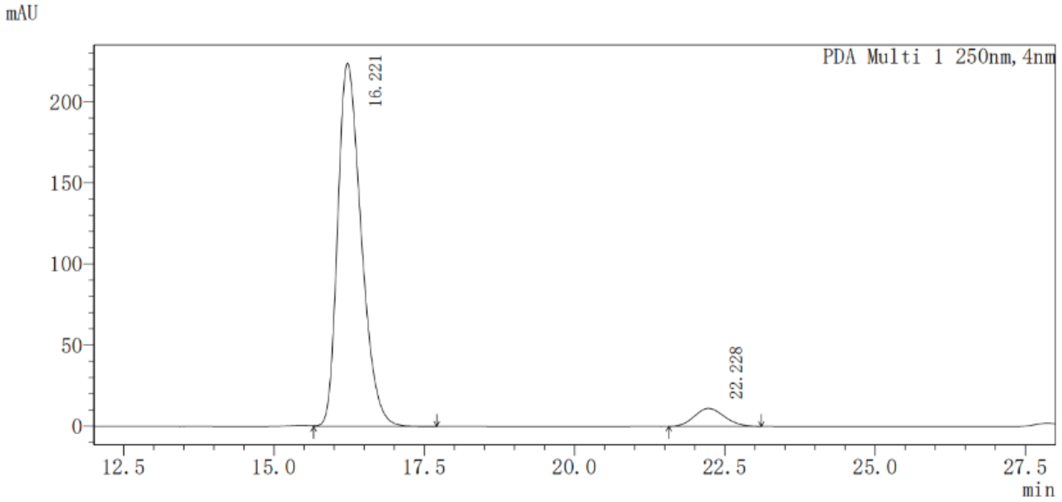

<Peak Results>

PDA Ch1 250nm

Index	Time/min	Height/mAU	Quantity/Area	Area %/%
1	16.221	223836	6084319	94.033
2	22.228	11090	386111	5.967

Supplementary Fig. 141. HPLC chromatograms of compound **3m.**

HPLC (ChiralPak OD-H, 2% *i*-PrOH in hexanes, 1.0 mL/min, 254 nm)

<Chromatogram>

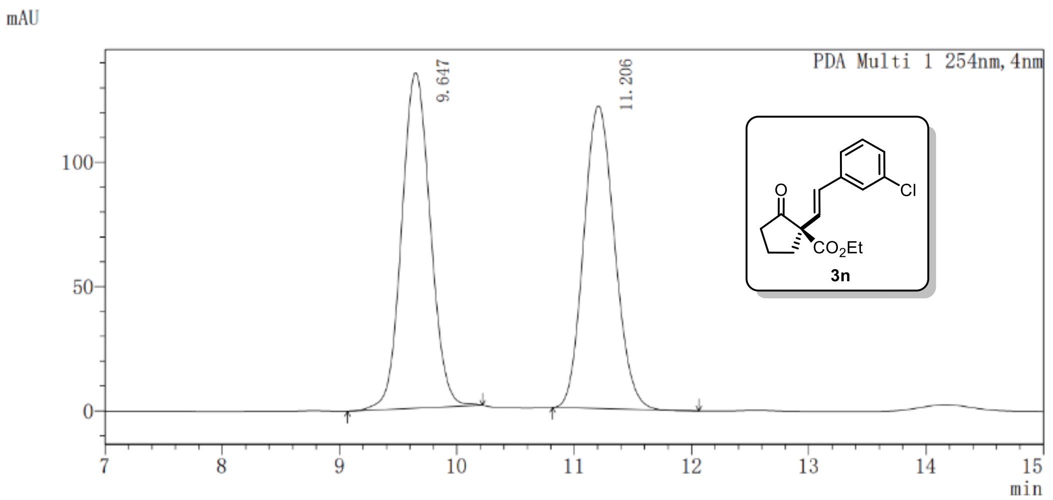

<Peak Results>

PDA Ch1 254nm

Index	Time/min	Height/mAU	Quantity/Area	Area %/%
1	9.647	134993	2292779	50.679
2	11.206	121739	2231308	49.321

<Chromatogram>

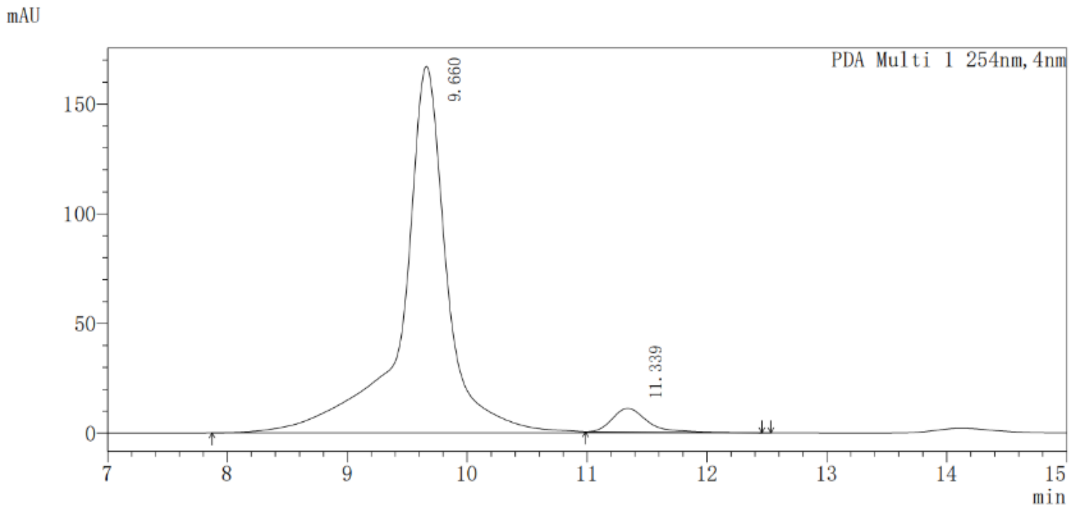

<Peak Results>

PDA Ch1 254nm

Index	Time/min	Height/mAU	Quantity/Area	Area %/%
1	9.660	167009	4348058	95.277
2	11.339	10774	215523	4.723

Supplementary Fig. 142. HPLC chromatograms of compound **3n.**

HPLC (ChiralPak OD-H, 5% *i*-PrOH in hexanes, 1.0 mL/min, 254 nm)

<Chromatogram>

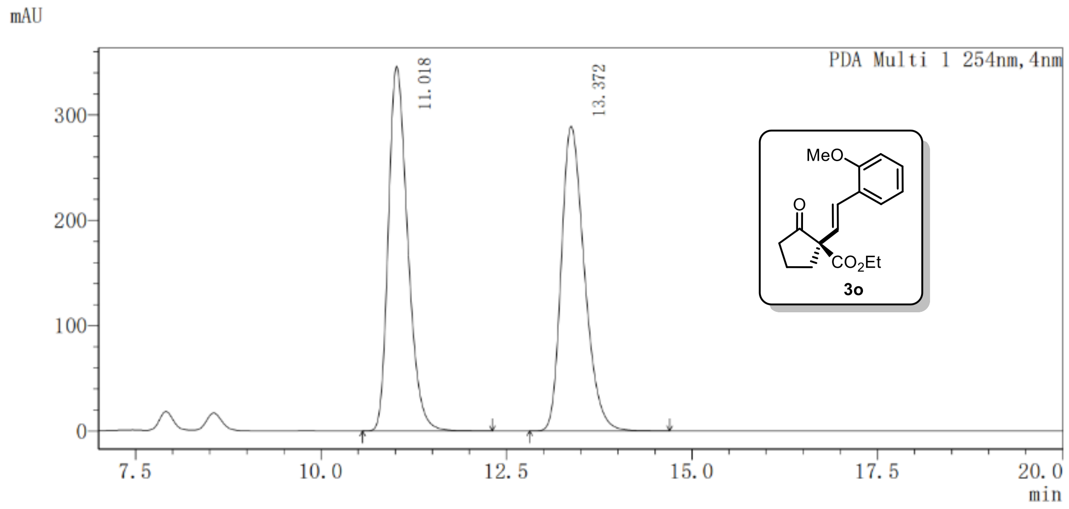

<Peak Results>

PDA Ch1 254nm

Index	Time/min	Height/mAU	Quantity/Area	Area %/%
1	11.018	346059	6254003	50.045
2	13.372	289100	6242879	49.955

<Chromatogram>

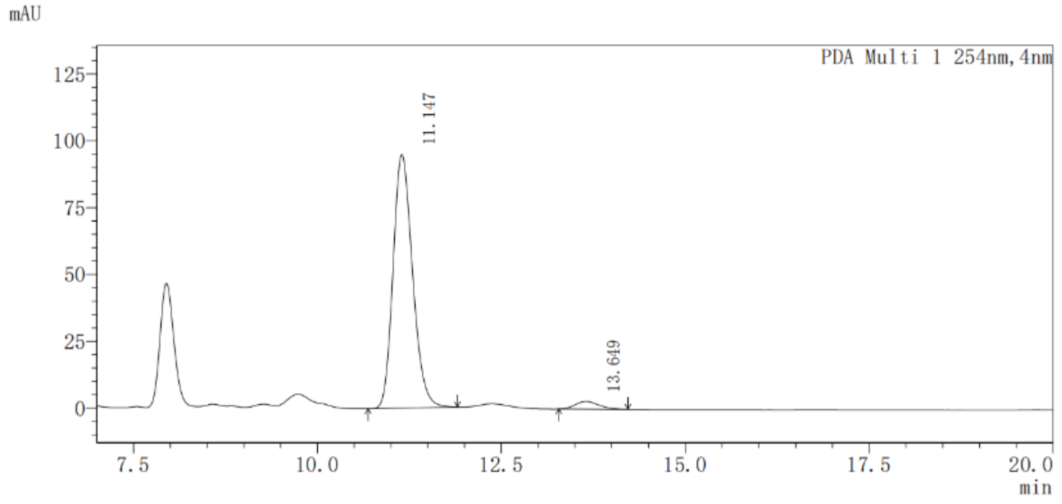

<Peak Results>

PDA Ch1 254nm

Index	Time/min	Height/mAU	Quantity/Area	Area %/%
1	11.147	94898	1746099	96.497
2	13.649	2833	63394	3.503

Supplementary Fig. 143. HPLC chromatograms of compound **3o.**

HPLC (ChiralPak AD-H, 2% *i*-PrOH in hexanes, 1.0 mL/min, 254 nm)

<Chromatogram>

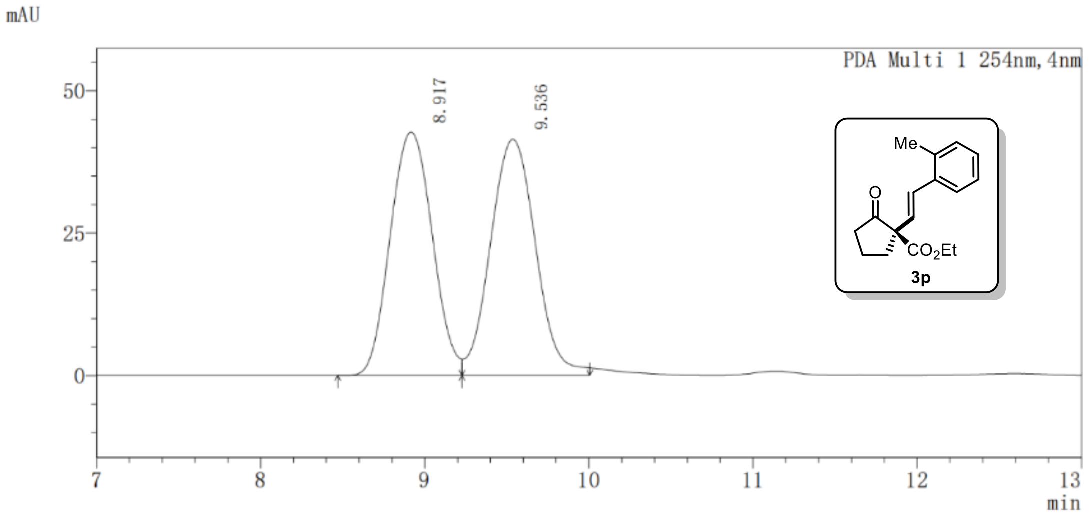

<Peak Results>

PDA Ch1 254nm

Index	Time/min	Height/mAU	Quantity/Area	Area %/%
1	8.917	42750	763937	49.158
2	9.536	41489	790122	50.842

<Chromatogram>

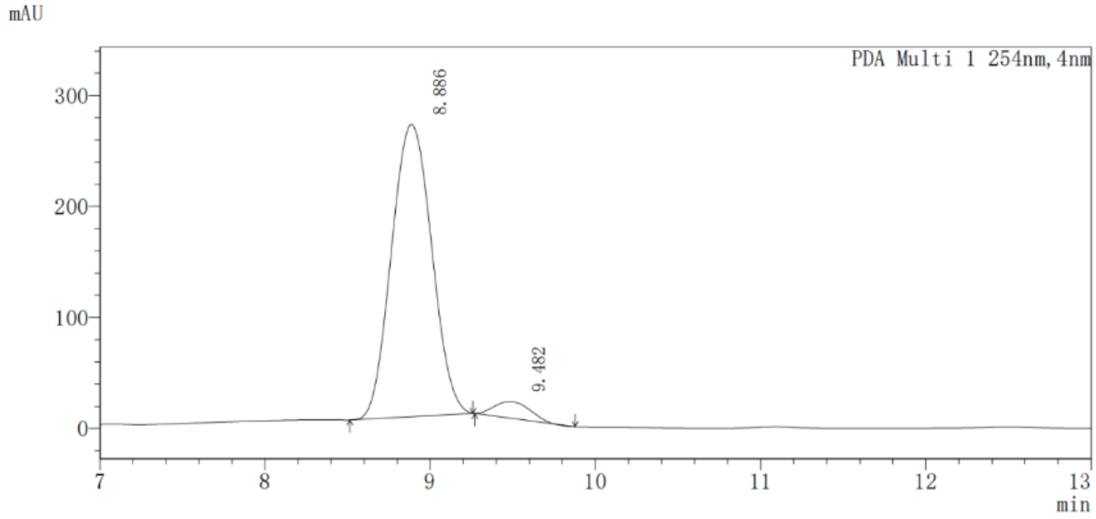

<Peak Results>

PDA Ch1 254nm

Index	Time/min	Height/mAU	Quantity/Area	Area %/%
1	8.886	263537	4463185	95.273
2	9.482	15005	221451	4.727

Supplementary Fig. 144. HPLC chromatograms of compound **3p.**

HPLC (ChiralPak OJ-H, 20% *i*-PrOH in hexanes, 1.0 mL/min, 270 nm)

<Chromatogram>

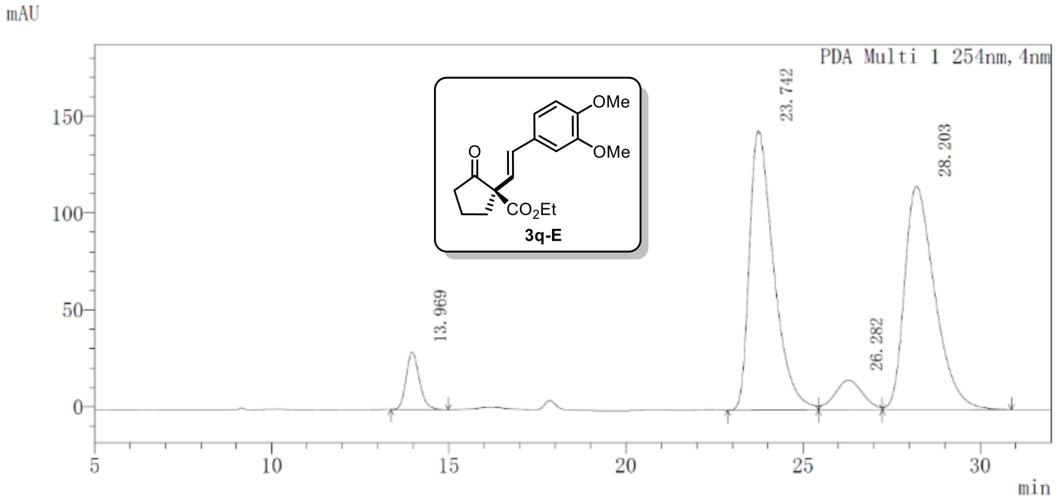

<Peak Results>

PDA Ch1 254nm

Index	Time/min	Height/mAU	Quantity/Area	Area %/%
1	13.969	29755	775694	5.036
2	23.742	144308	6888674	44.721
3	26.282	15435	849334	5.514
4	28.203	115503	6889837	44.729

<Chromatogram>

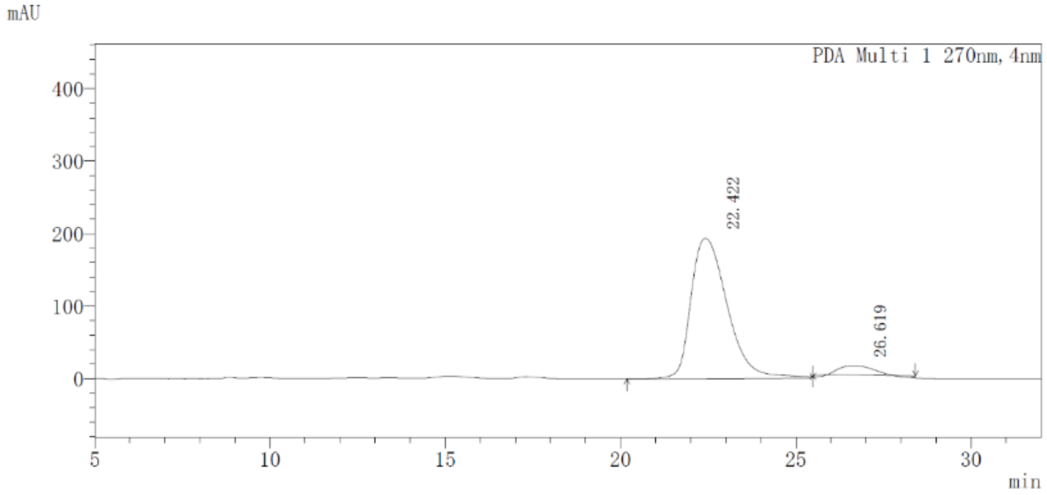

<Peak Results>

PDA Ch1 270nm

Index	Time/min	Height/mAU	Quantity/Area	Area %/%
1	22.422	193642	13978916	94.787
2	26.619	12743	768749	5.213

Supplementary Fig. 145. HPLC chromatograms of compound **3q-*E*.**

HPLC (ChiralPak OJ-H, 20% *i*-PrOH in hexanes, 1.0 mL/min, 254 nm)

<Chromatogram>

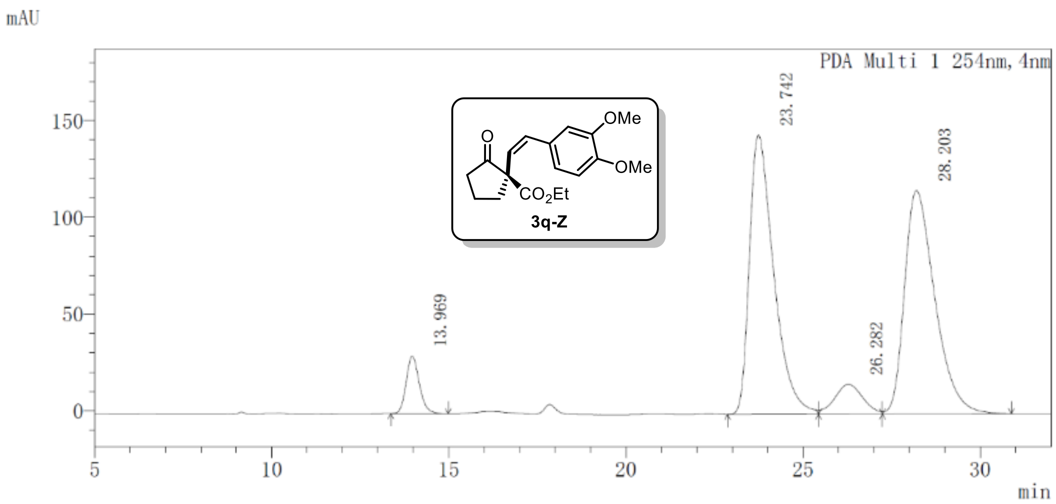

<Peak Results>

PDA Ch1 254nm

Index	Time/min	Height/mAU	Quantity/Area	Area %/%
1	13.969	29755	775694	5.036
2	23.742	144308	6888674	44.721
3	26.282	15435	849334	5.514
4	28.203	115503	6889837	44.729

<Chromatogram>

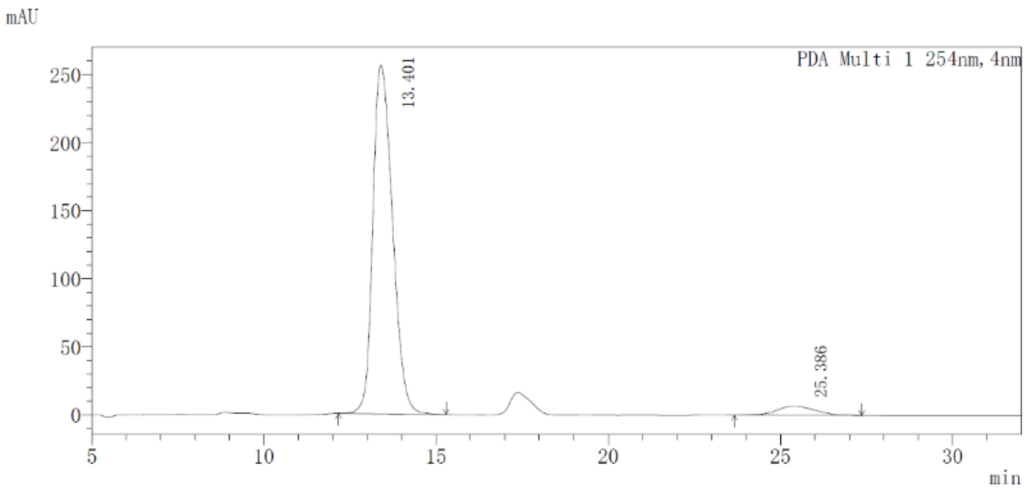

<Peak Results>

PDA Ch1 254nm

Index	Time/min	Height/mAU	Quantity/Area	Area %/%
1	13.401	256106	10166895	95.173
2	25.386	6687	515609	4.827

Supplementary Fig. 146. HPLC chromatograms of compound **3q-*Z*.**

HPLC (ChiralPak AD-H, 2% *i*-PrOH in hexanes, 1.0 mL/min, 254 nm)

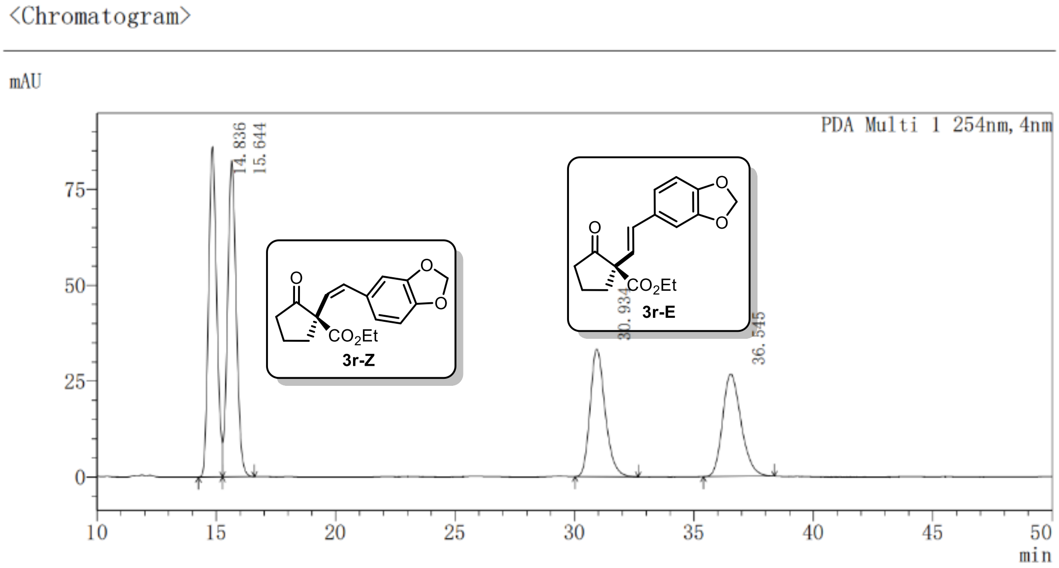

<Peak Results>

PDA Ch1 254nm

Index	Time/min	Height/mAU	Quantity/Area	Area %/%
1	14.836	85985	2046449	29.097
2	15.644	82395	2085827	29.657
3	30.934	33213	1456363	20.707
4	36.545	26724	1444529	20.539

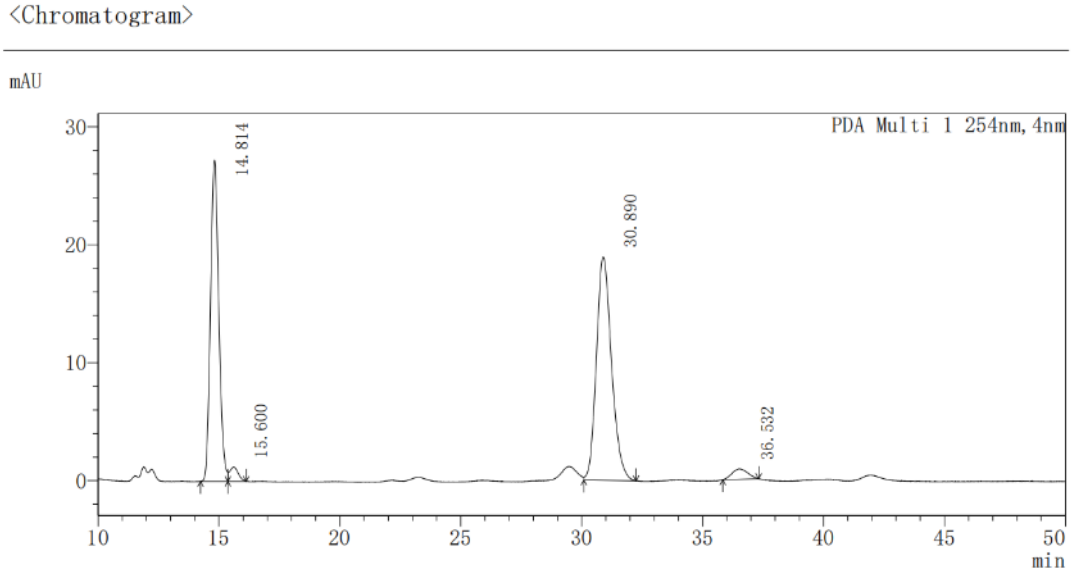

<Peak Results>

PDA Ch1 254nm

Index	Time/min	Height/mAU	Quantity/Area	Area %/%
1	14.814	27198	651180	42.201
2	15.600	1218	29913	1.939
3	30.890	18923	822407	53.297
4	36.532	883	39554	2.563

Supplementary Fig. 147. HPLC chromatograms of compound **3r.**

HPLC (ChiralPak AD-H, 2% *i*-PrOH in hexanes, 1.0 mL/min, 254 nm)

<Chromatogram>

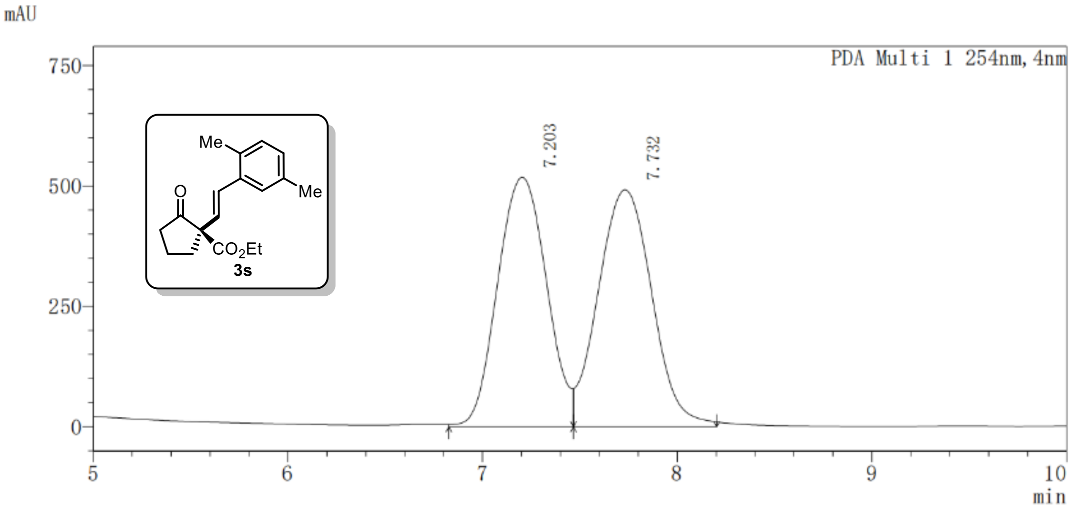

<Peak Results>

PDA Ch1 254nm

Index	Time/min	Height/mAU	Quantity/Area	Area %/%
1	7.203	517568	9082775	49.081
2	7.732	491903	9422774	50.919

<Chromatogram>

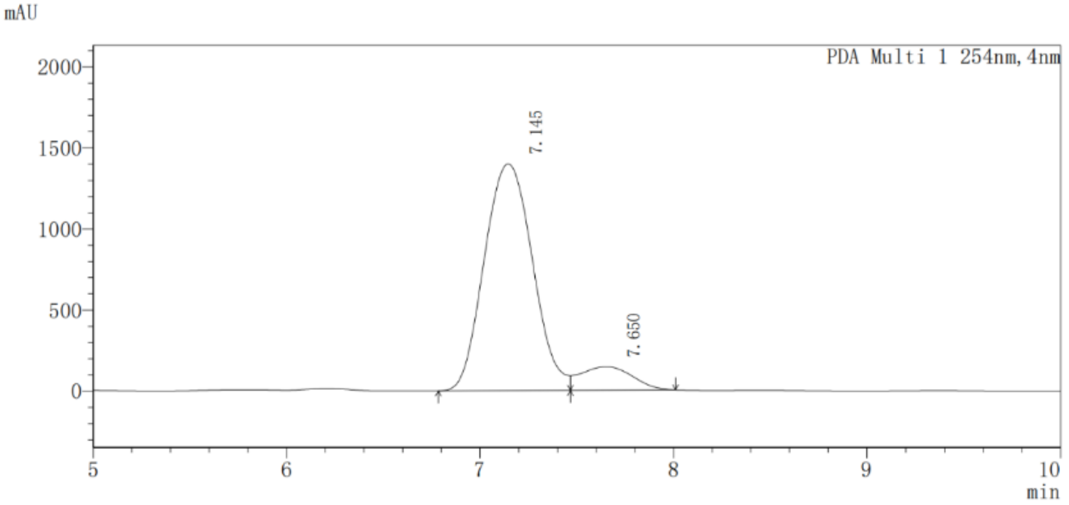

<Peak Results>

PDA Ch1 254nm

Index	Time/min	Height/mAU	Quantity/Area	Area %/%
1	7.145	1399408	24326589	90.078
2	7.650	145246	2679429	9.922

Supplementary Fig. 148. HPLC chromatograms of compound **3s.**

HPLC (ChiralPak AD-H, 2% *i*-PrOH in hexanes, 1.0 mL/min, 254 nm)

<Chromatogram>

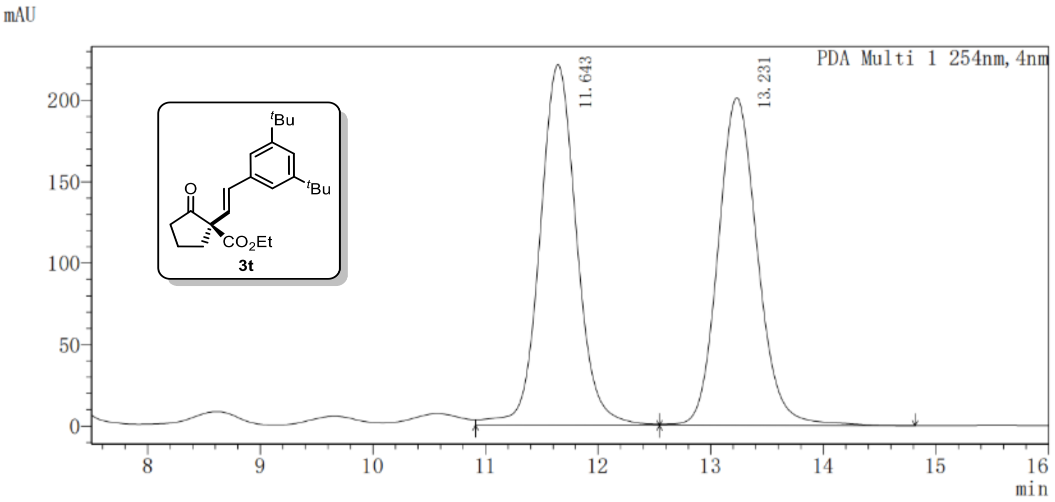

<Peak Results>

PDA Ch1 254nm

Index	Time/min	Height/mAU	Quantity/Area	Area %/%
1	11.643	221358	5020945	50.585
2	13.231	201115	4904782	49.415

<Chromatogram>

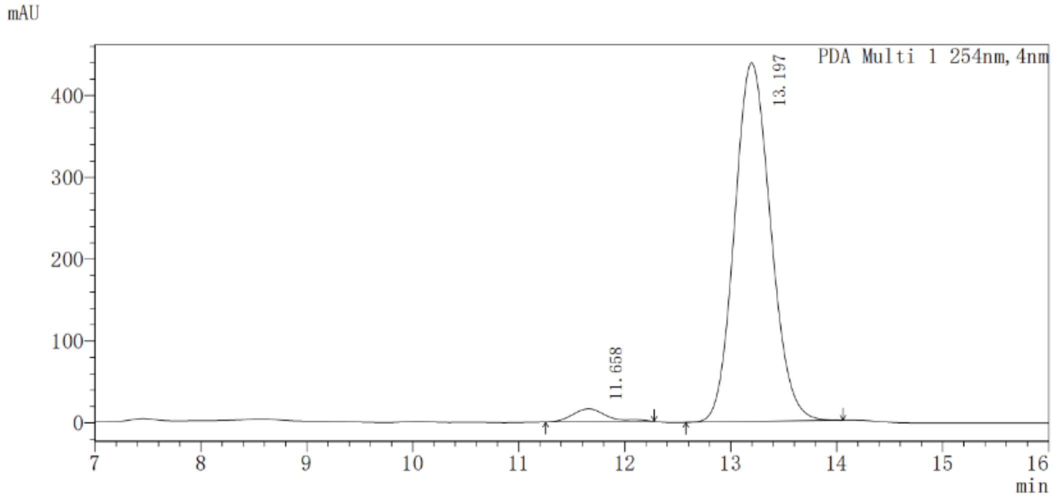

<Peak Results>

PDA Ch1 254nm

Index	Time/min	Height/mAU	Quantity/Area	Area %/%
1	11.658	15693	354934	3.302
2	13.197	438216	10395170	96.698

Supplementary Fig. 149. HPLC chromatograms of compound **3t.**

HPLC (ChiralPak IC, 2% *i*-PrOH in hexanes, 1.0 mL/min, 265 nm)

<Chromatogram>

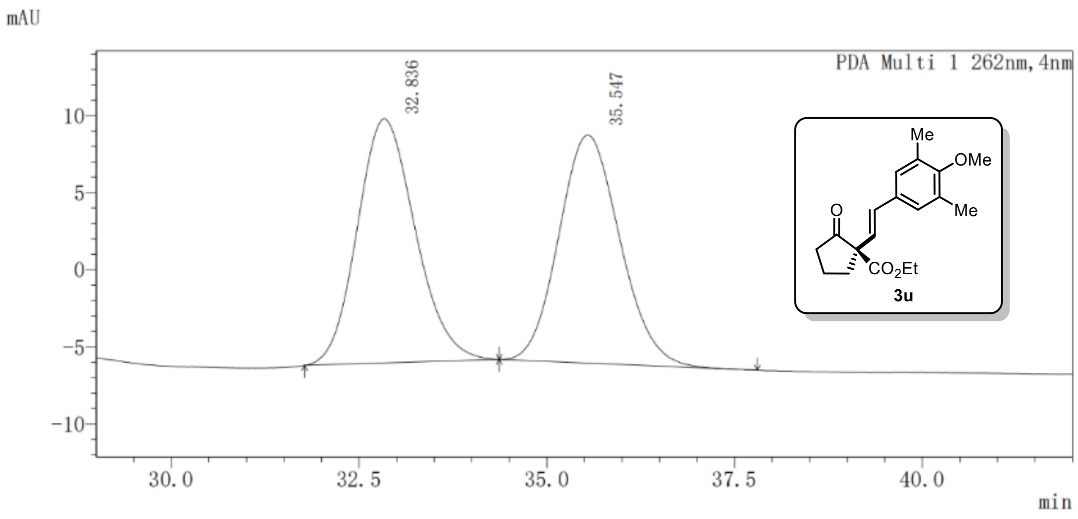

<Peak Results>

PDA Ch1 262nm

Index	Time/min	Height/mAU	Quantity/Area	Area %/%
1	32.836	15837	852175	50.397
2	35.547	14795	838734	49.603

<Chromatogram>

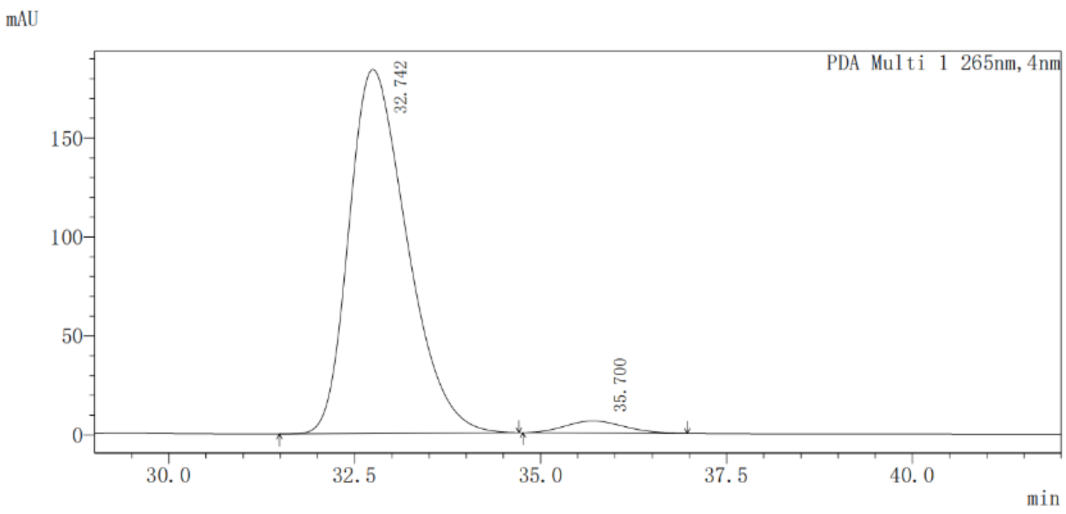

<Peak Results>

PDA Ch1 265nm

Index	Time/min	Height/mAU	Quantity/Area	Area %/%
1	32.742	183877	9997358	96.795
2	35.700	6081	331030	3.205

Supplementary Fig. 150. HPLC chromatograms of compound **3u.**

HPLC (ChiralPak IC, 2% *i*-PrOH in hexanes, 1.0 mL/min, 254 nm)

<Chromatogram>

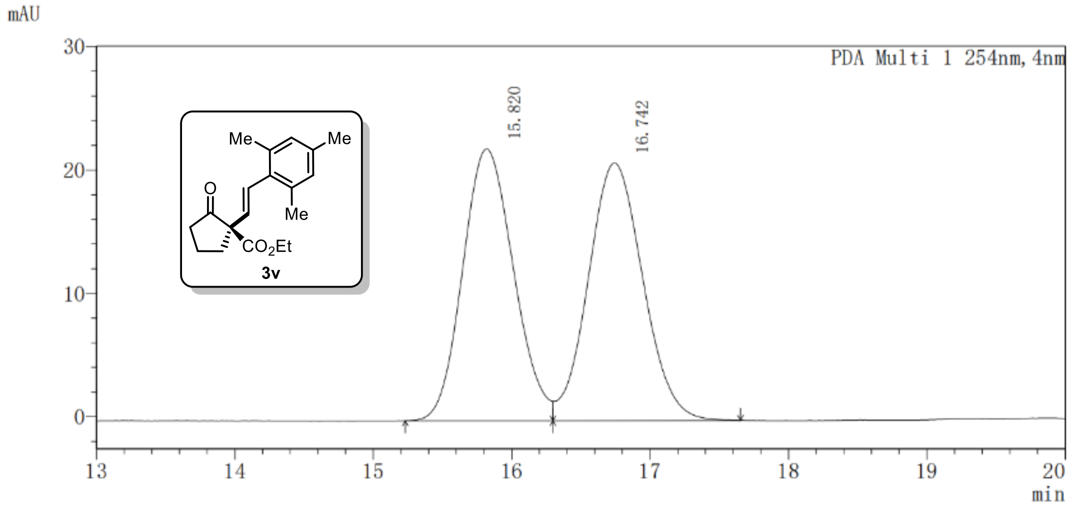

<Peak Results>

PDA Ch1 254nm

Index	Time/min	Height/mAU	Quantity/Area	Area %/%
1	15.820	22007	563735	49.643
2	16.742	20856	571852	50.357

<Chromatogram>

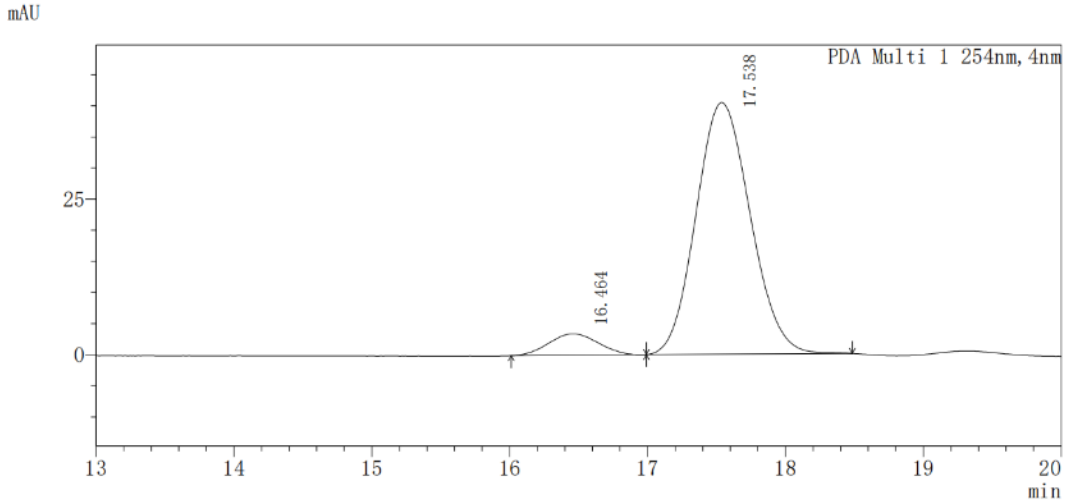

<Peak Results>

PDA Ch1 254nm

Index	Time/min	Height/mAU	Quantity/Area	Area %/%
1	16.464	3427	84728	7.108
2	17.538	40477	1107354	92.892

Supplementary Fig. 151. HPLC chromatograms of compound **3v.**

HPLC (ChiralPak IC, 2% *i*-PrOH in hexanes, 1.0 mL/min, 254 nm)

<Chromatogram>

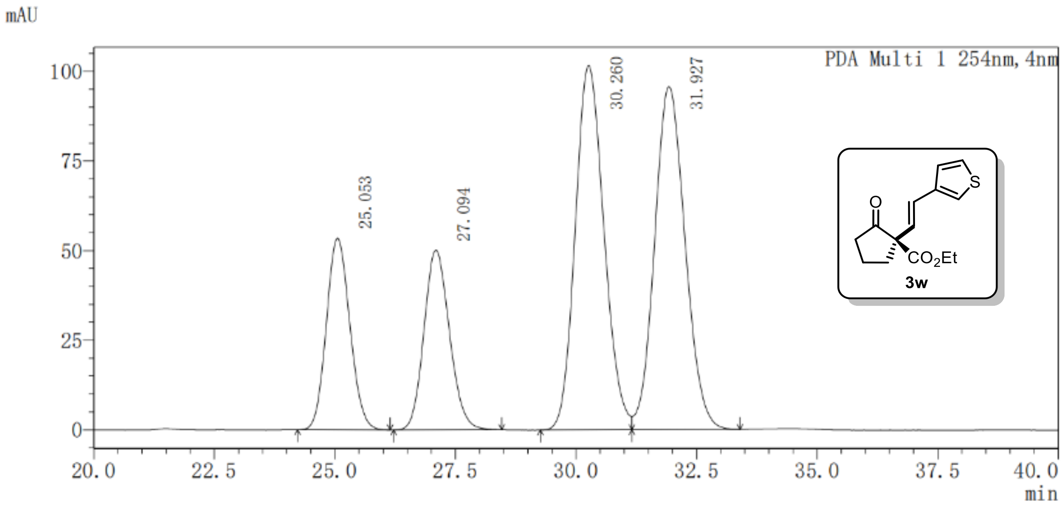

<Peak Results>

PDA Ch1 254nm

Index	Time/min	Height/mAU	Quantity/Area	Area %/%
1	25.053	53496	1814691	15.003
2	27.094	50153	1847609	15.275
3	30.260	101667	4217093	34.865
4	31.927	95698	4216077	34.857

<Chromatogram>

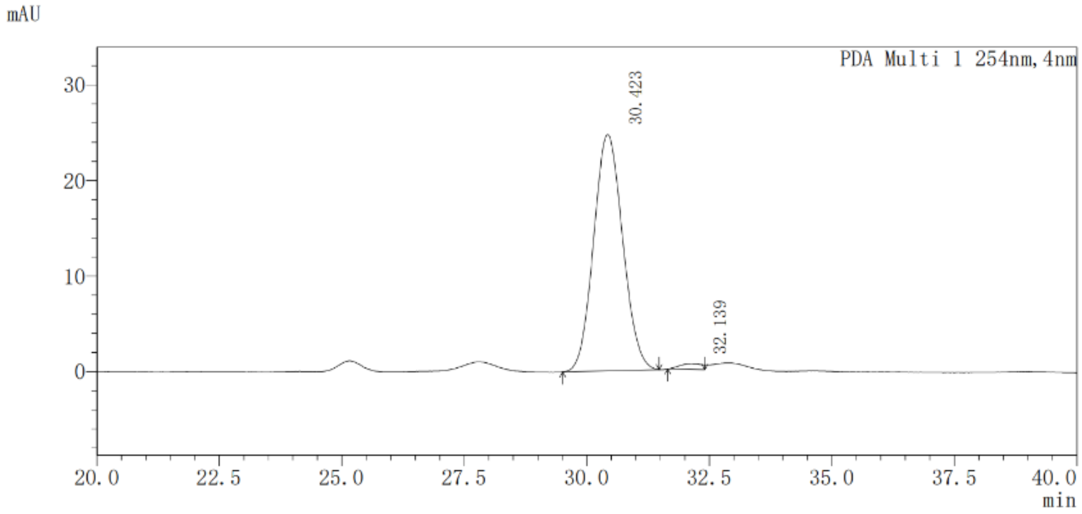

<Peak Results>

PDA Ch1 254nm

Index	Time/min	Height/mAU	Quantity/Area	Area %/%
1	30.423	24737	1023131	98.188
2	32.139	600	18881	1.812

Supplementary Fig. 152. HPLC chromatograms of compound **3w.**

HPLC (ChiralPak OJ-H, 5% *i*-PrOH in hexanes, 1.0 mL/min, 254 nm)

<Chromatogram>

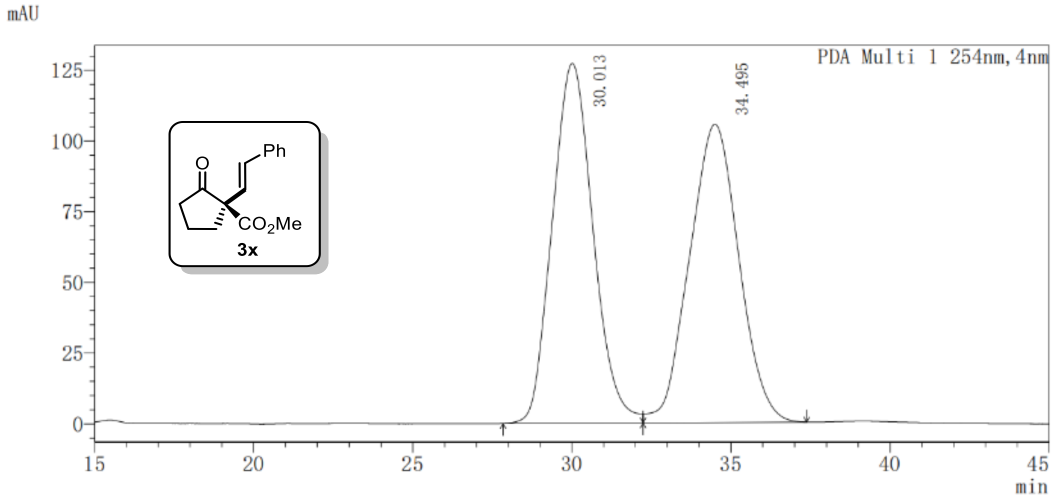

<Peak Results>

PDA Ch1 254nm

Index	Time/min	Height/mAU	Quantity/Area	Area %/%
1	30.013	127185	11325328	49.821
2	34.495	105464	11406493	50.179

<Chromatogram>

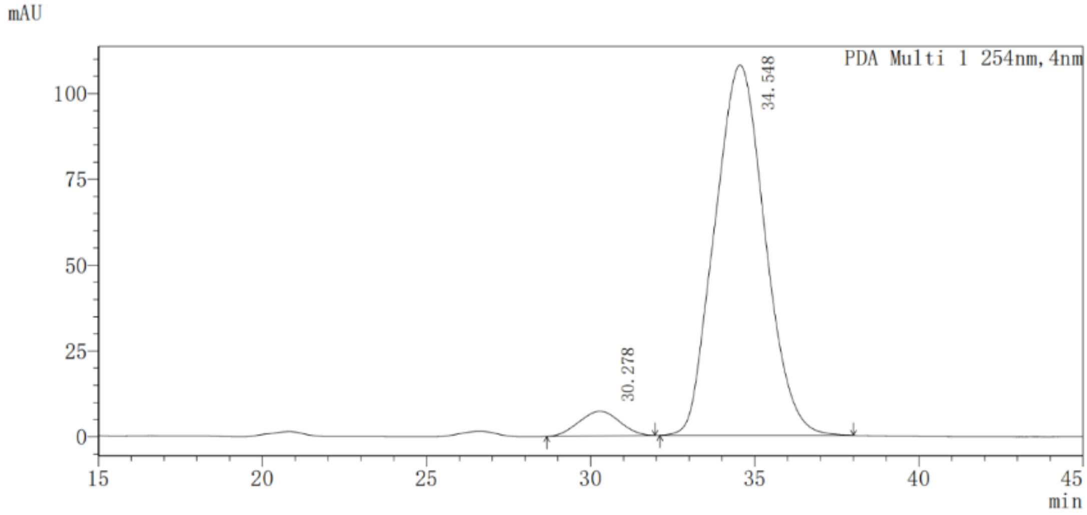

<Peak Results>

PDA Ch1 254nm

Index	Time/min	Height/mAU	Quantity/Area	Area %/%
1	30.278	7195	615628	5.090
2	34.548	107888	11479602	94.910

Supplementary Fig. 153. HPLC chromatograms of compound **3x.**

HPLC (ChiralPak IC, 2% *i*-PrOH in hexanes, 1.0 mL/min, 254 nm)

<Chromatogram>

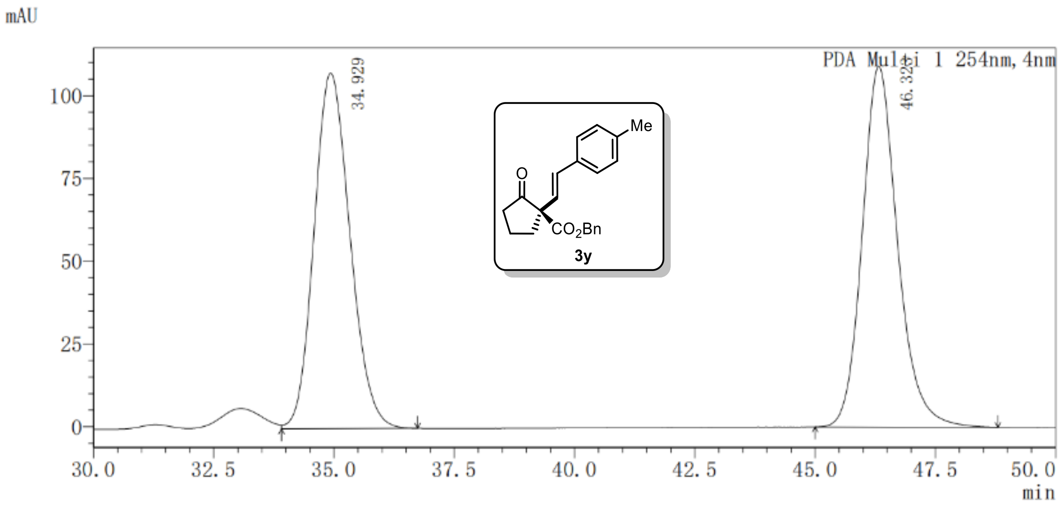

<Peak Results>

PDA Ch1 254nm

Index	Time/min	Height/mAU	Quantity/Area	Area %/%
1	34.929	107365	5588107	50.029
2	46.323	109070	5581700	49.971

<Chromatogram>

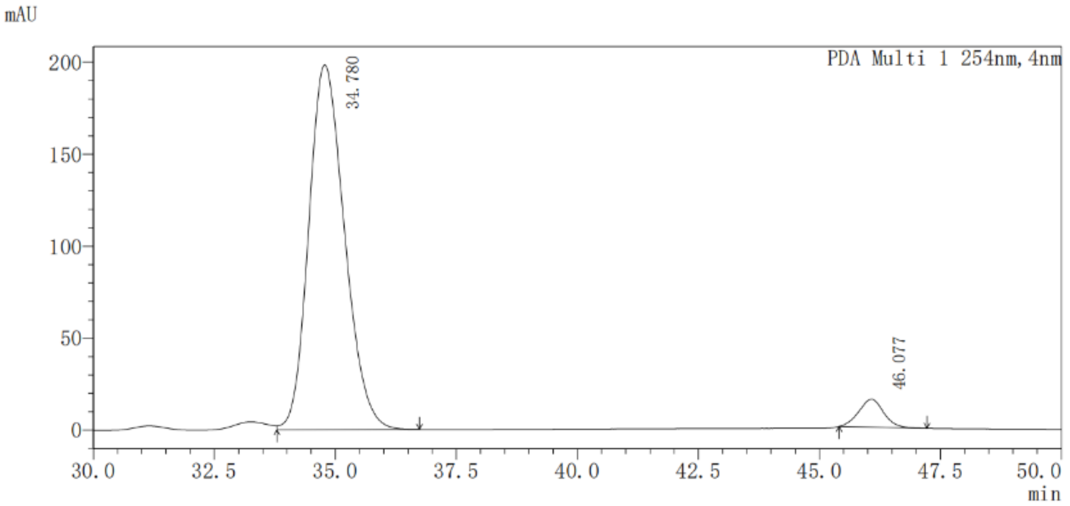

<Peak Results>

PDA Ch1 254nm

Index	Time/min	Height/mAU	Quantity/Area	Area %/%
1	34.780	198312	10232912	94.956
2	46.077	15216	543589	5.044

Supplementary Fig. 154. HPLC chromatograms of compound **3y.**

HPLC (ChiralPak AD-H, 2% *i*-PrOH in hexanes, 1.0 mL/min, 254 nm)

<Chromatogram>

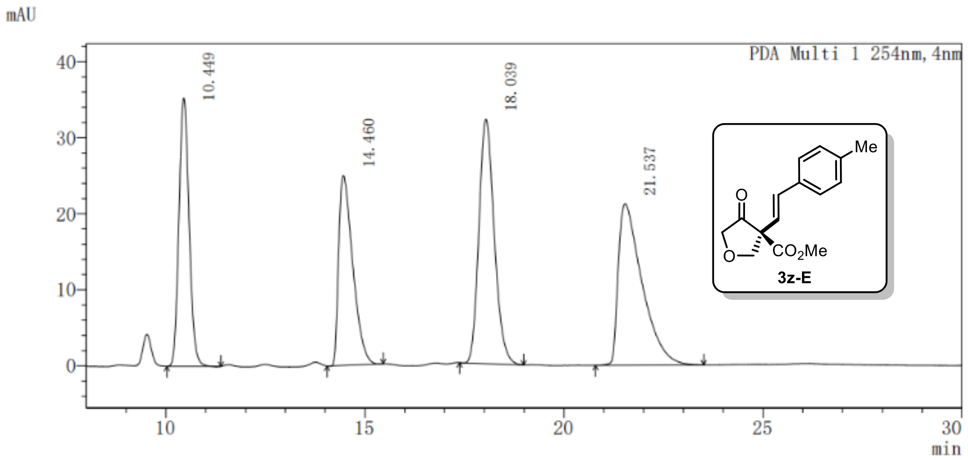

<Peak Results>

PDA Ch1 254nm

Index	Time/min	Height/mAU	Quantity/Area	Area %/%
1	10.449	35269	627824	21.006
2	14.460	24985	615390	20.589
3	18.039	32140	860452	28.789
4	21.537	21256	885188	29.616

<Chromatogram>

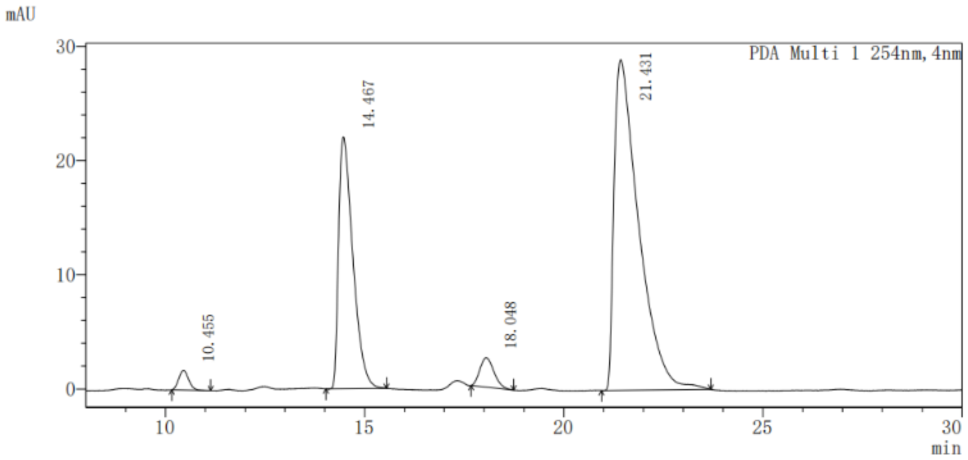

<Peak Results>

PDA Ch1 254nm

Index	Time/min	Height/mAU	Quantity/Area	Area %/%
1	10.455	1712	29665	1.575
2	14.467	22051	549762	29.184
3	18.048	2574	63272	3.359
4	21.431	28955	1241051	65.882

Supplementary Fig. 155. HPLC chromatograms of compound **3z.**

HPLC (ChiralPak IC, 2% *i*-PrOH in hexanes, 1.0 mL/min, 254 nm)

<Chromatogram>

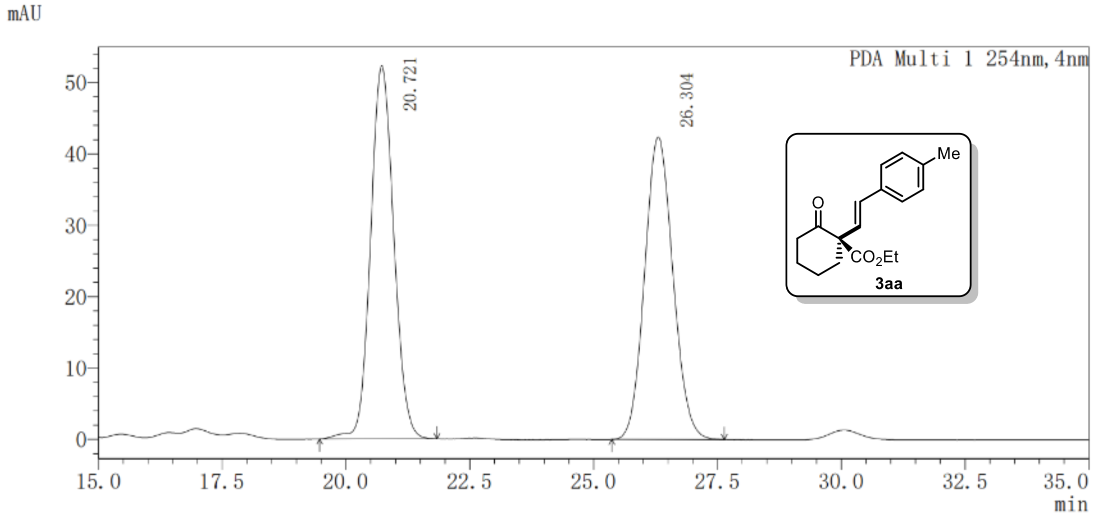

<Peak Results>

PDA Ch1 254nm

Index	Time/min	Height/mAU	Quantity/Area	Area %/%
1	20.721	52304	1708267	50.290
2	26.304	42397	1688591	49.710

<Chromatogram>

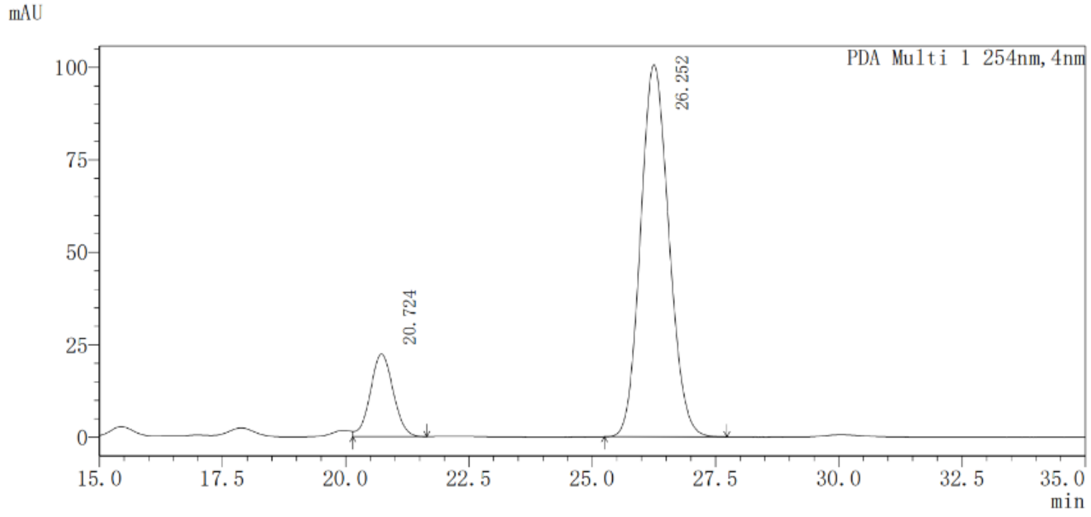

<Peak Results>

PDA Ch1 254nm

Index	Time/min	Height/mAU	Quantity/Area	Area %/%
1	20.724	22345	720838	15.186
2	26.252	100594	4025873	84.814

Supplementary Fig. 156. HPLC chromatograms of compound **3aa.**

HPLC (ChiralPak IC, 2% *i*-PrOH in hexanes, 1.0 mL/min, 254 nm)

<Chromatogram>

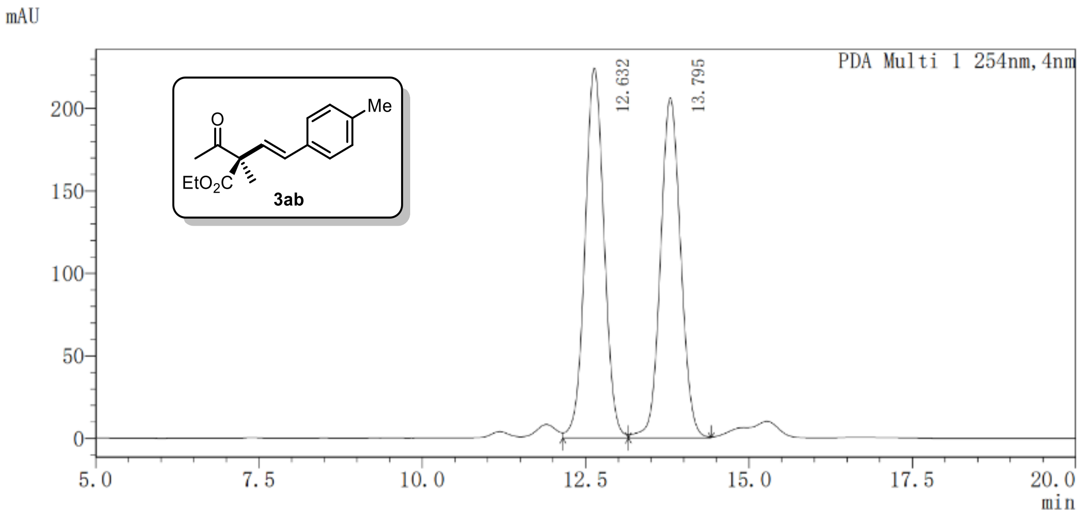

<Peak Results>

PDA Ch1 254nm

Index	Time/min	Height/mAU	Quantity/Area	Area %/%
1	12.632	224140	4546198	50.629
2	13.795	205932	4433256	49.371

<Chromatogram>

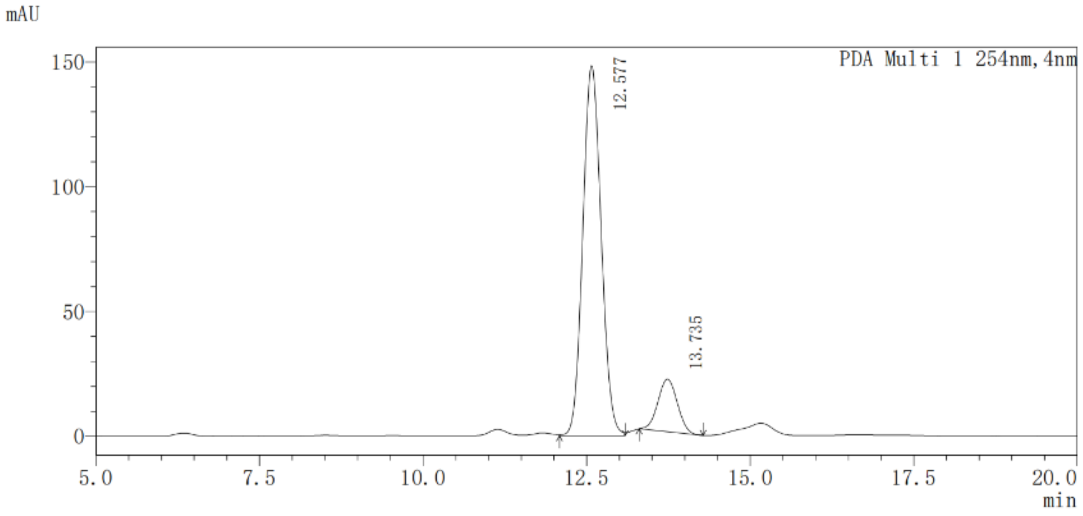

<Peak Results>

PDA Ch1 254nm

Index	Time/min	Height/mAU	Quantity/Area	Area %/%
1	12.577	148116	2895808	86.963
2	13.735	21044	434110	13.037

Supplementary Fig. 157. HPLC chromatograms of compound **3ab.**

HPLC (ChiralPak IC, 2% *i*-PrOH in hexanes, 1.0 mL/min, 254 nm)

<Chromatogram>

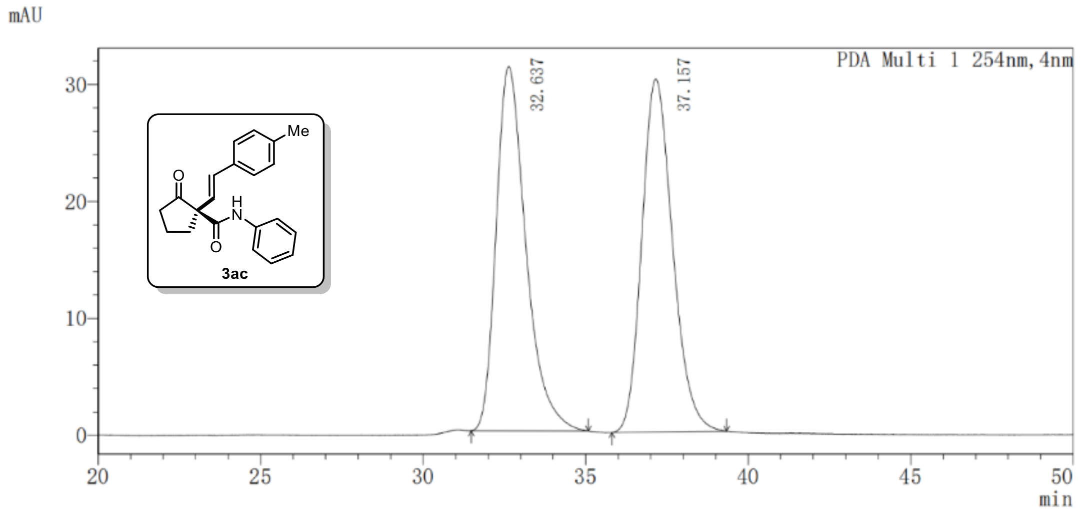

<Peak Results>

PDA Ch1 254nm

Index	Time/min	Height/mAU	Quantity/Area	Area %/%
1	32.637	31136	1985204	49.939
2	37.157	30199	1990093	50.061

<Chromatogram>

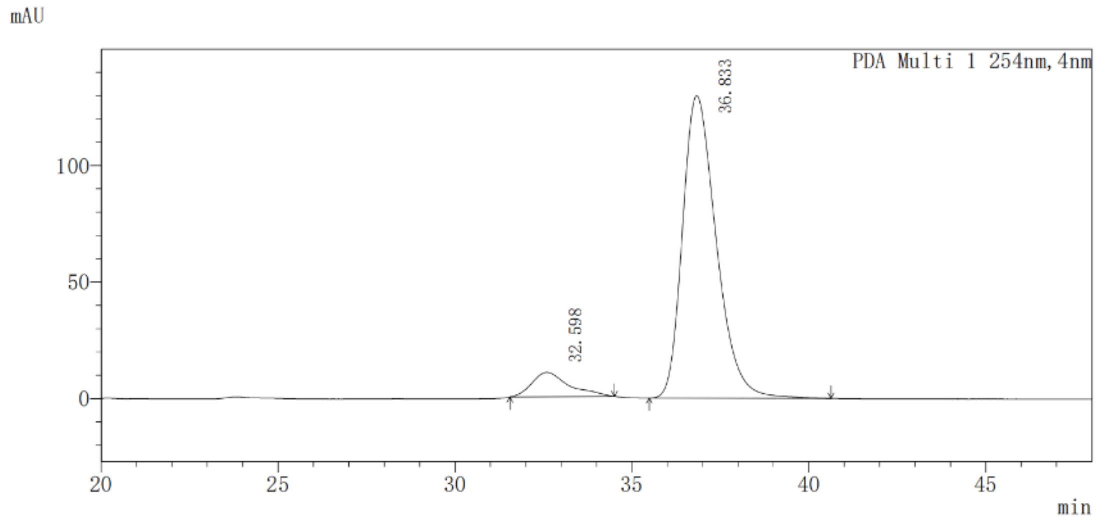

<Peak Results>

PDA Ch1 254nm

Index	Time/min	Height/mAU	Quantity/Area	Area %/%
1	32.598	10518	773913	8.233
2	36.833	129817	8626120	91.767

Supplementary Fig. 158. HPLC chromatograms of compound **3ac.**

HPLC (ChiralPak IC, 5% *i*-PrOH in hexanes, 1.0 mL/min, 262 nm)

<Chromatogram>

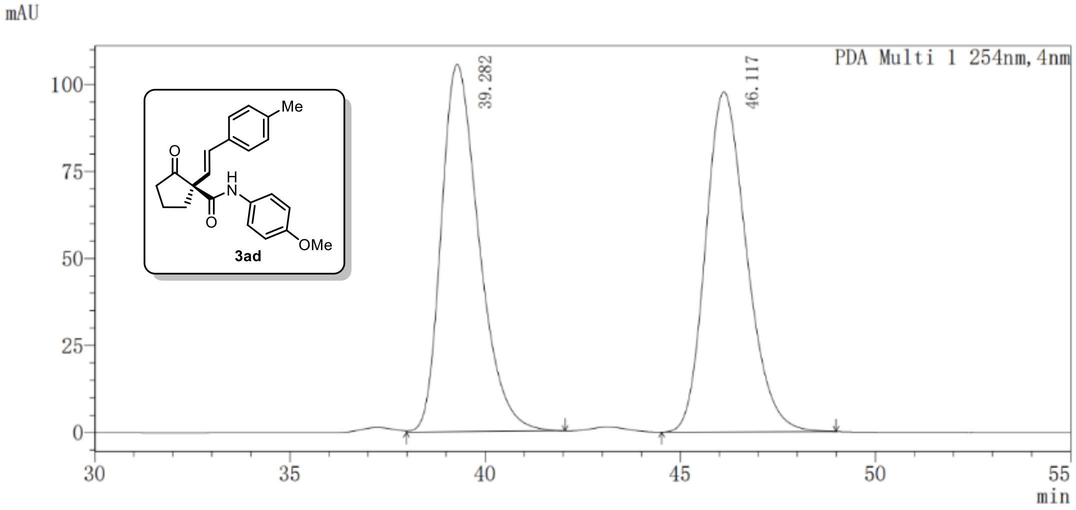

<Peak Results>

PDA Ch1 254nm

Index	Time/min	Height/mAU	Quantity/Area	Area %/%
1	39.282	105541	7103799	49.852
2	46.117	97713	7146019	50.148

<Chromatogram>

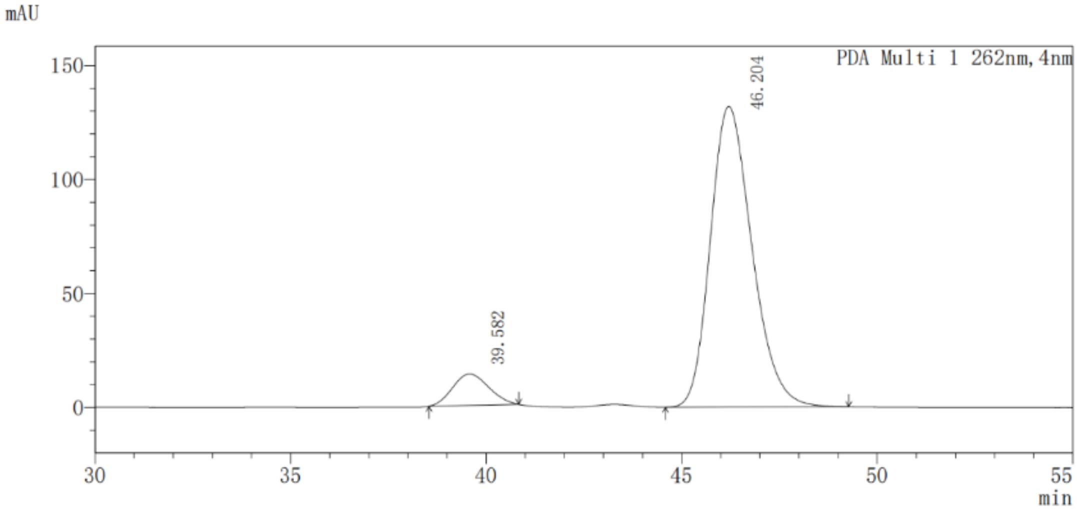

<Peak Results>

PDA Ch1 262nm

Index	Time/min	Height/mAU	Quantity/Area	Area %/%
1	39.582	13762	882799	8.285
2	46.204	131929	9772484	91.715

Supplementary Fig. 159. HPLC chromatograms of compound **3ad.**

HPLC (ChiralPak AD-H, 5% *i*-PrOH in hexanes, 1.0 mL/min, 254 nm)

<Chromatogram>

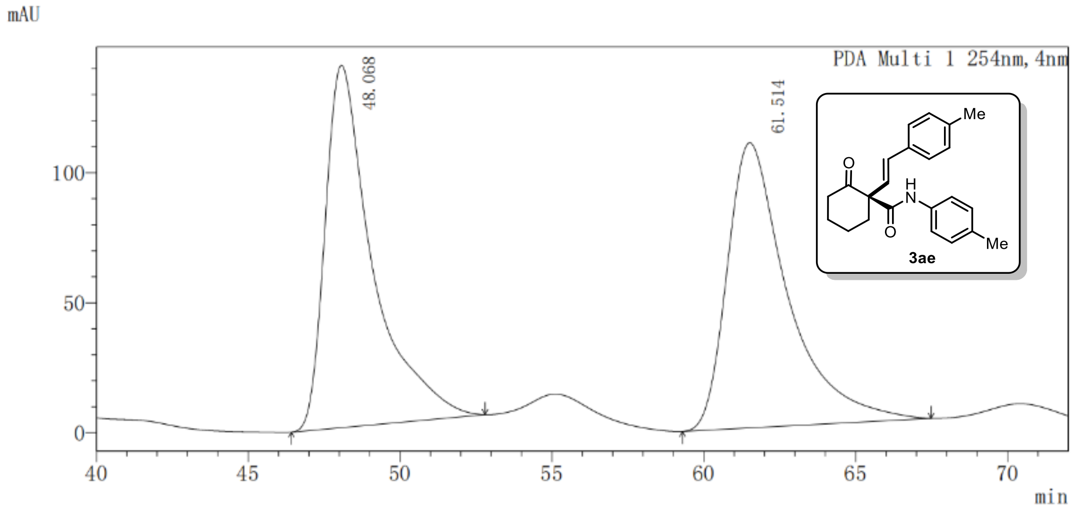

<Peak Results>

PDA Ch1 254nm

Index	Time/min	Height/mAU	Quantity/Area	Area %/%
1	48.068	139282	15165078	49.901
2	61.514	109586	15225389	50.099

<Chromatogram>

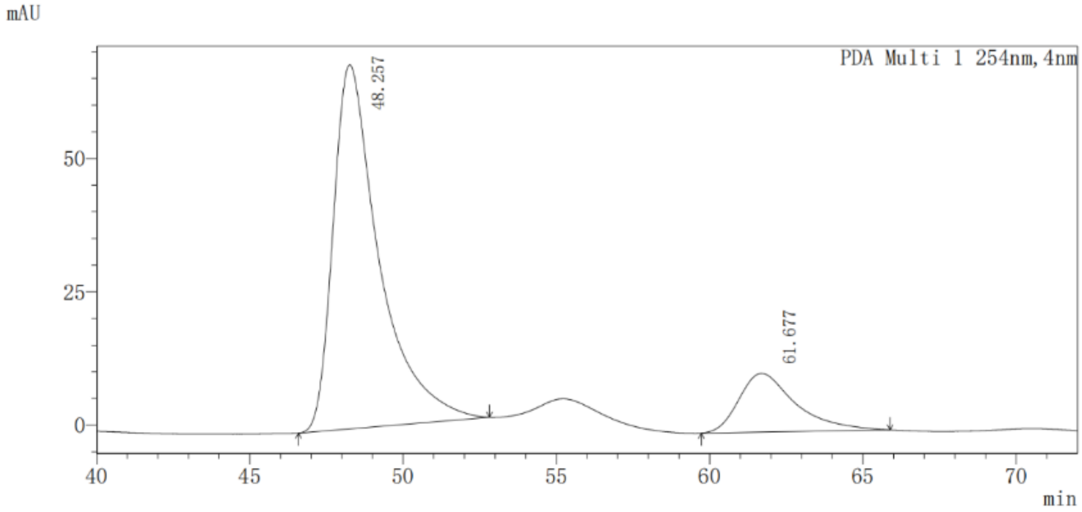

<Peak Results>

PDA Ch1 254nm

Index	Time/min	Height/mAU	Quantity/Area	Area %/%
1	48.257	68264	7141063	83.475
2	61.677	11014	1413634	16.525

Supplementary Fig. 160. HPLC chromatograms of compound **3ae.**

HPLC (ChiralPak OD-H, 10% *i*-PrOH in hexanes, 1.0 mL/min, 254 nm)

<Chromatogram>

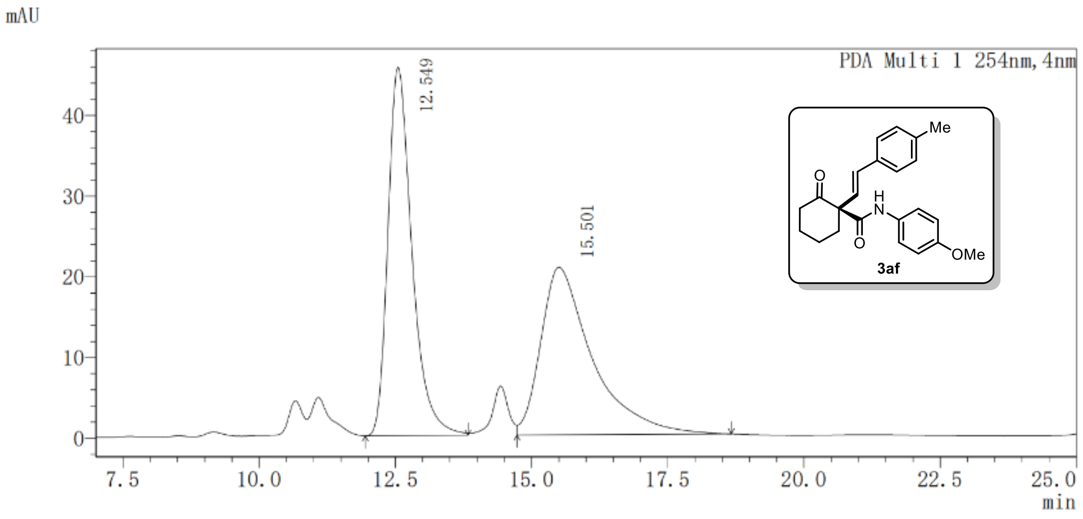

<Peak Results>

PDA Ch1 254nm

Index	Time/min	Height/mAU	Quantity/Area	Area %/%
1	12.549	45641	1401773	50.576
2	15.501	20781	1369867	49.424

<Chromatogram>

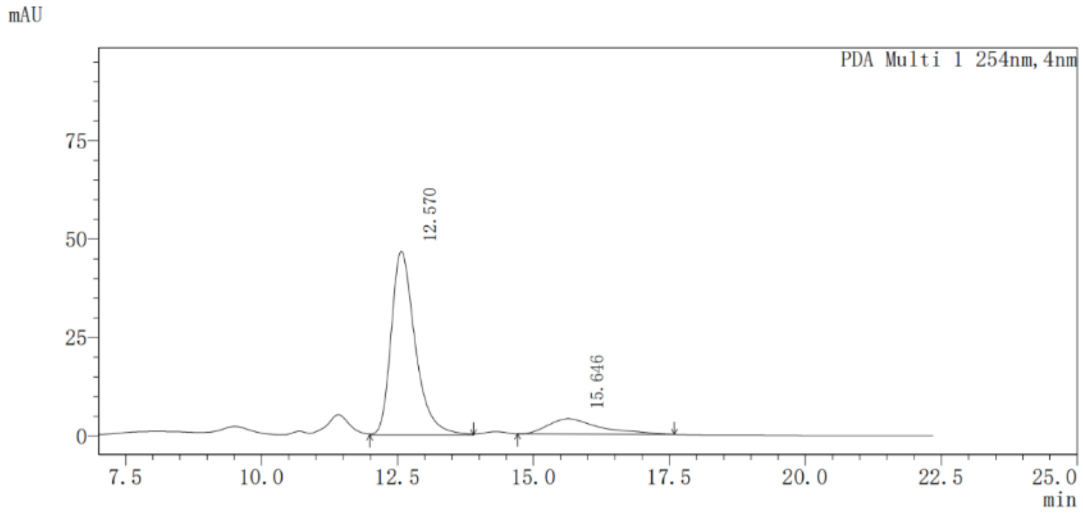

<Peak Results>

PDA Ch1 254nm

Index	Time/min	Height/mAU	Quantity/Area	Area %/%
1	12.570	46618	1433001	84.803
2	15.646	3926	256804	15.197

Supplementary Fig. 161. HPLC chromatograms of compound **3af.**

HPLC (ChiralPak OD-H*2, 2% *i*-PrOH in hexanes, 0.5 mL/min, 254 nm)

<Chromatogram>

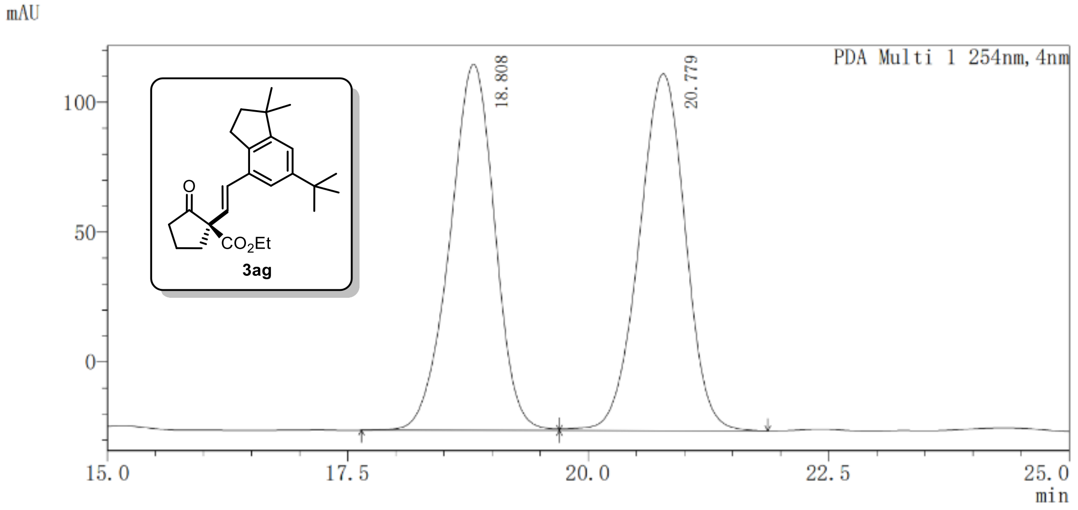

<Peak Results>

PDA Ch1 254nm

Index	Time/min	Height/mAU	Quantity/Area	Area %/%
1	18.808	140973	4588843	49.968
2	20.779	137429	4594807	50.032

<Chromatogram>

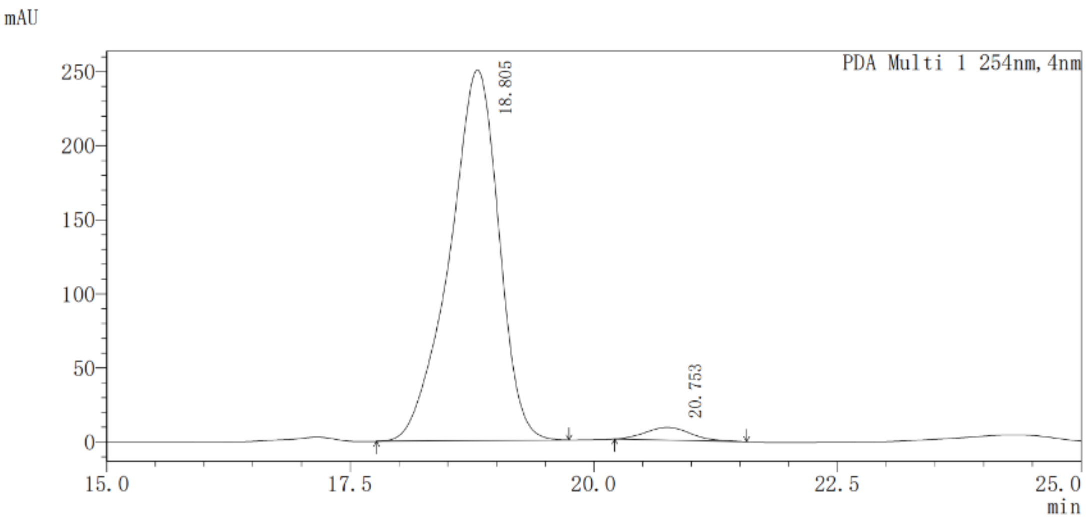

<Peak Results>

PDA Ch1 254nm

Index	Time/min	Height/mAU	Quantity/Area	Area %/%
1	18.805	250332	8810852	96.907
2	20.753	8580	281193	3.093

Supplementary Fig. 162. HPLC chromatograms of compound **3ag.**

HPLC (ChiralPak IE, 2% *i*-PrOH in hexanes, 1.0 mL/min, 254 nm)

<色谱图>

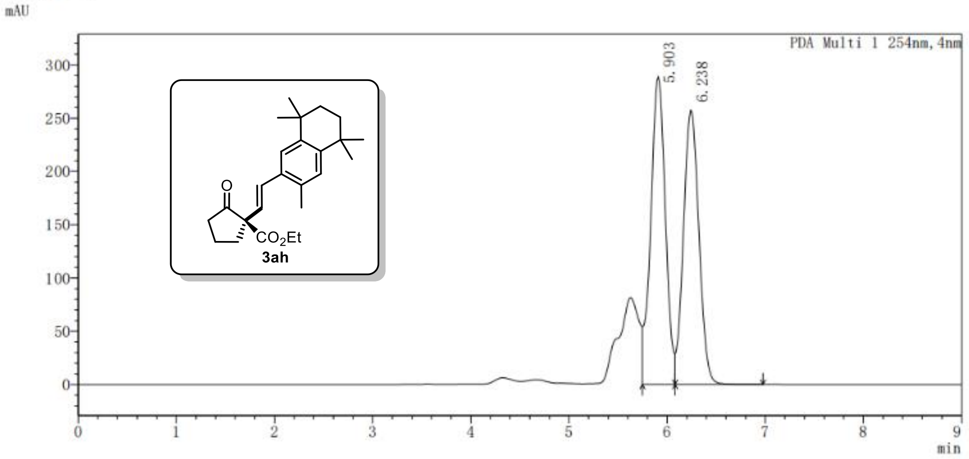

PDA Ch1 254nm

峰号	保留时间	峰宽(高度 50%)	高度	面积	面积%
1	5.903	0.160	288613	2981876	51.408
2	6.238	0.172	257168	2818525	48.592

<色谱图>

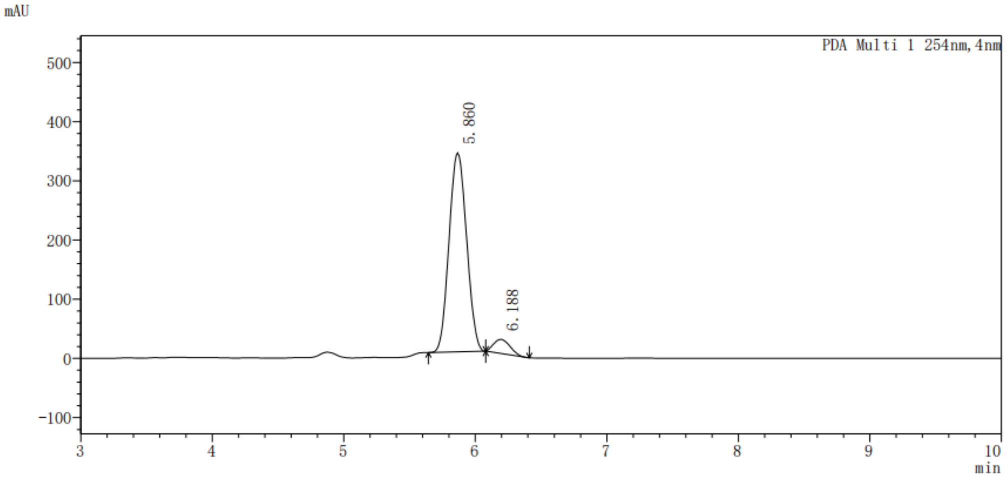

PDA Ch1 254nm

峰号	保留时间	峰宽(高度 50%)	高度	面积	面积%
1	5.860	0.147	336359	3109045	93.935
2	6.188	0.144	23338	200743	6.065

Supplementary Fig. 163. HPLC chromatograms of compound **3ah.**

HPLC (ChiralPak AD-H*2, 5% *i*-PrOH in hexanes, 0.8 mL/min, 254 nm)

<Chromatogram>

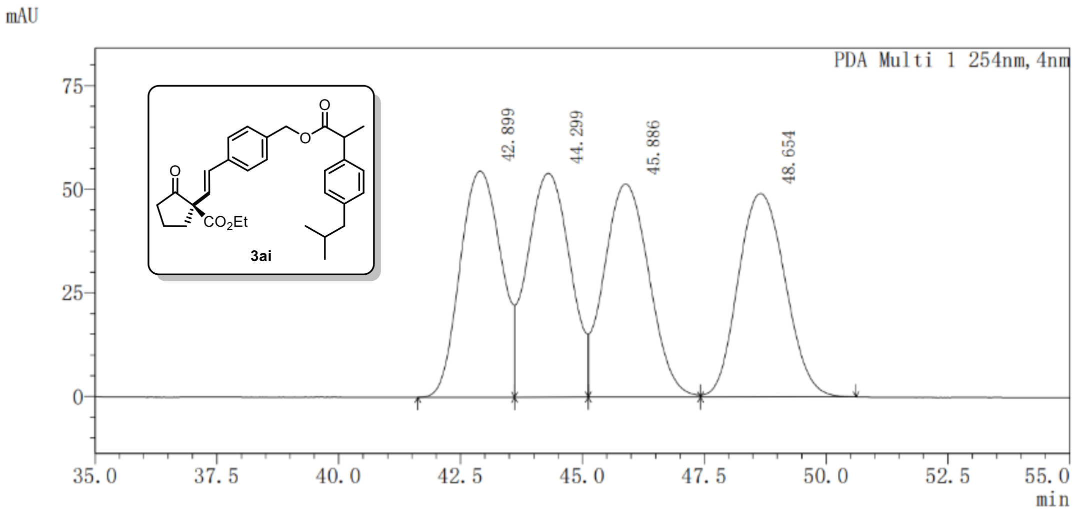

<Peak Results>

PDA Ch1 254nm

Index	Time/min	Height/mAU	Quantity/Area	Area %/%
1	42.899	54568	3205038	24.328
2	44.299	54017	3334997	25.314
3	45.886	51425	3324144	25.232
4	48.654	49122	3310363	25.127

<Chromatogram>

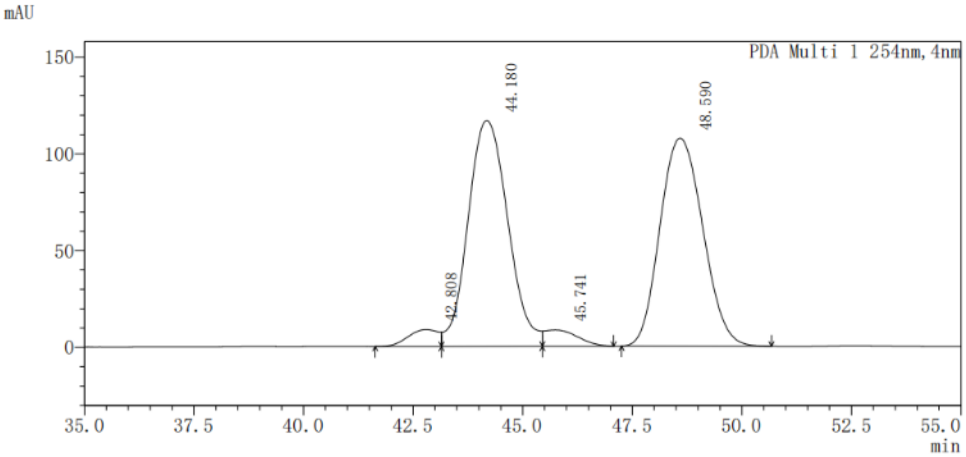

<Peak Results>

PDA Ch1 254nm

Index	Time/min	Height/mAU	Quantity/Area	Area %/%
1	42.808	8709	419777	2.696
2	44.180	116805	7395738	47.506
3	45.741	8388	436868	2.806
4	48.590	107492	7315551	46.991

Supplementary Fig. 164. HPLC chromatograms of compound **3ai.**

HPLC (ChiralPak IA*2, 15% *i*-PrOH in hexanes, 0.8 mL/min, 254 nm)

<Chromatogram>

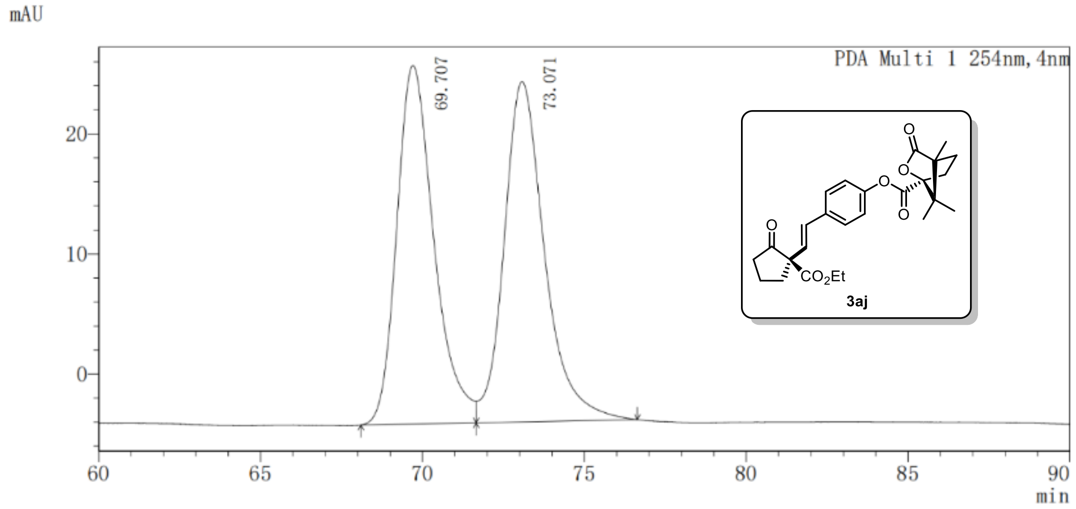

<Peak Results>

PDA Ch1 254nm

Index	Time/min	Height/mAU	Quantity/Area	Area %/%
1	69.707	29872	2384282	49.124
2	73.071	28352	2469280	50.876

<Chromatogram>

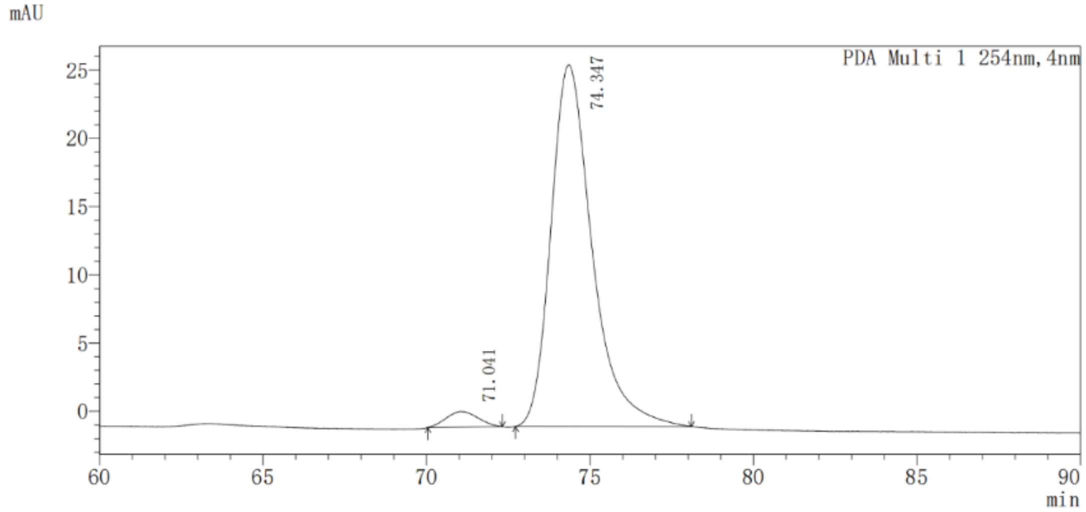

<Peak Results>

PDA Ch1 254nm

Index	Time/min	Height/mAU	Quantity/Area	Area %/%
1	71.041	1142	77350	3.218
2	74.347	26494	2326049	96.782

Supplementary Fig. 165. HPLC chromatograms of compound **3aj.**

HPLC (ChiralPak OD-H, 10% *i*-PrOH in hexanes, 1.0 mL/min, 260 nm)

<Chromatogram>

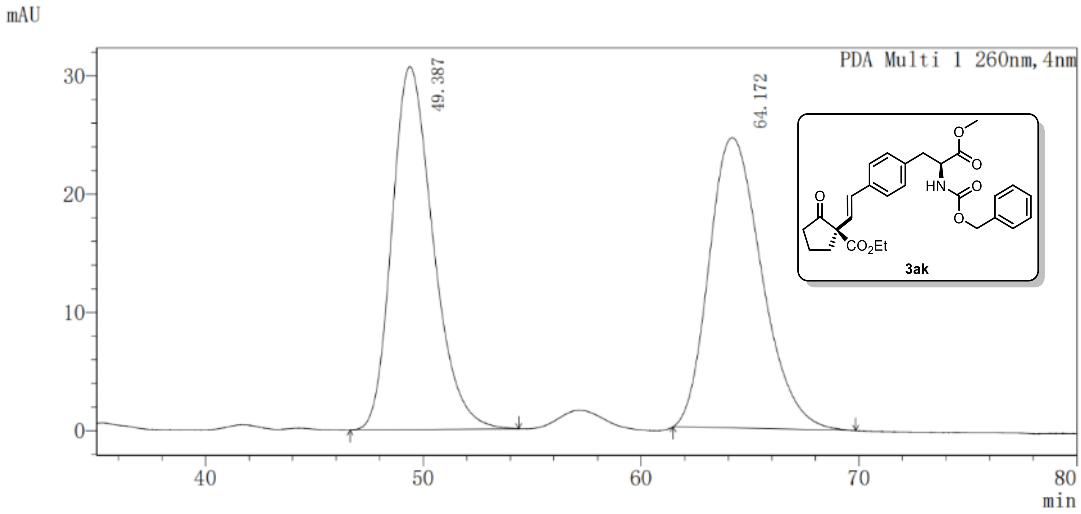

<Peak Results>

PDA Ch1 260nm

Index	Time/min	Height/mAU	Quantity/Area	Area %/%
1	49.387	30686	4031946	49.327
2	64.172	24558	4141909	50.673

<Chromatogram>

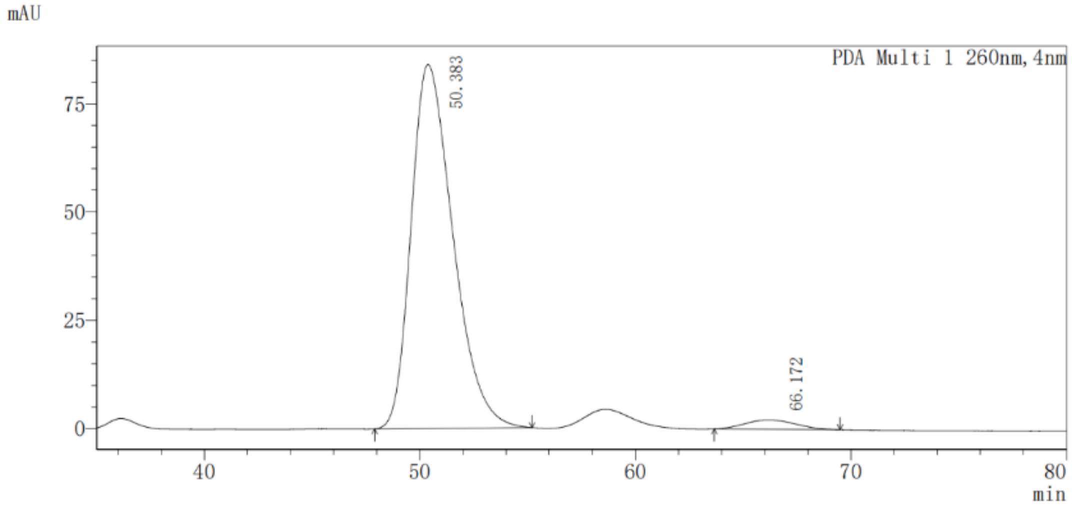

<Peak Results>

PDA Ch1 260nm

Index	Time/min	Height/mAU	Quantity/Area	Area %/%
1	50.383	83963	11350811	97.038
2	66.172	2117	346497	2.962

Supplementary Fig. 166. HPLC chromatograms of compound **3ak.**

HPLC (ChiralPak OD-H*2, 2% *i*-PrOH in hexanes, 0.8 mL/min, 254 nm)

<Chromatogram>

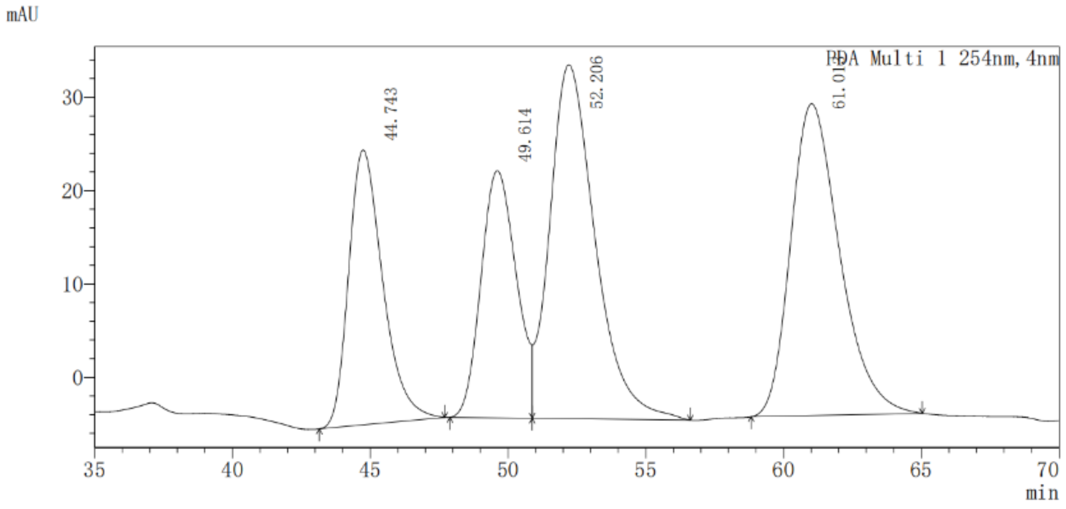

<Peak Results>

PDA Ch1 254nm

Index	Time/min	Height/mAU	Quantity/Area	Area %/%
1	44.743	29444	2533822	18.951
2	49.614	26468	2358772	17.641
3	52.206	37902	4348349	32.522
4	61.013	33409	4129731	30.886

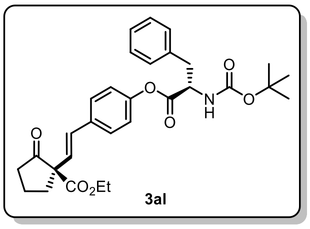

<Chromatogram>

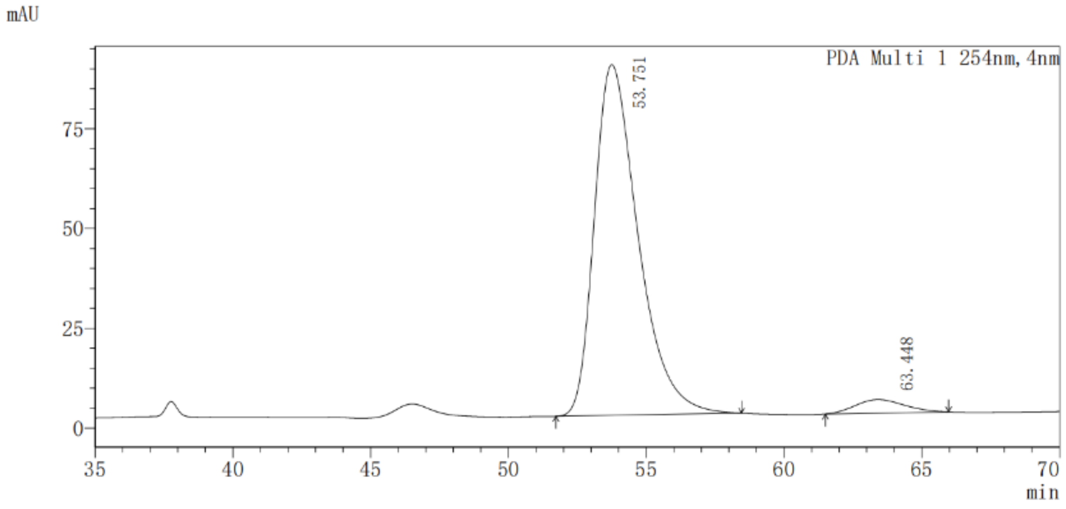

<Peak Results>

PDA Ch1 254nm

Index	Time/min	Height/mAU	Quantity/Area	Area %/%
1	53.751	87844	9716540	95.856
2	63.448	3382	420059	4.144

Supplementary Fig. 167. HPLC chromatograms of compound **3al.**

HPLC (ChiralPak IC, 5% *i*-PrOH in hexanes, 1.0 mL/min, 254 nm)

<Chromatogram>

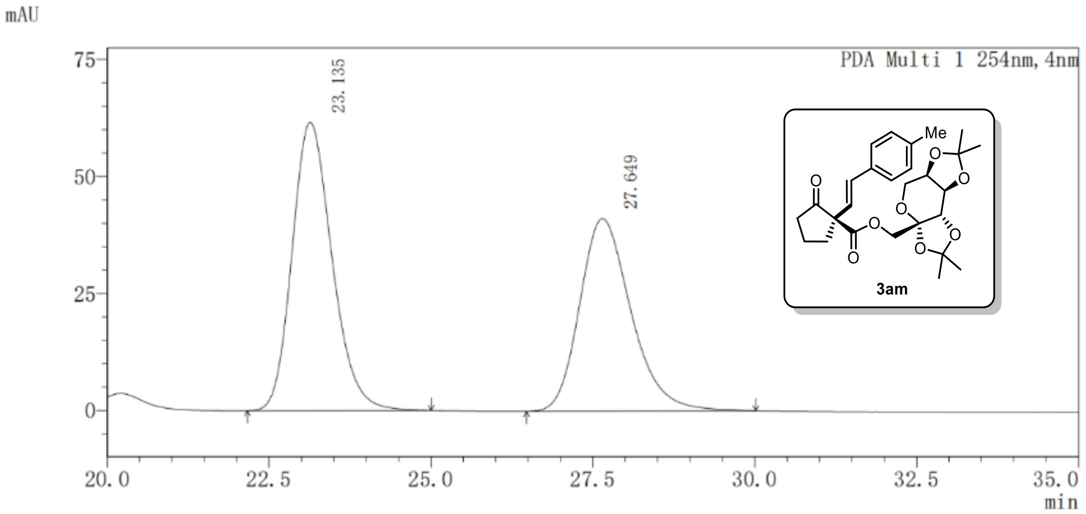

<Peak Results>

PDA Ch1 254nm

Index	Time/min	Height/mAU	Quantity/Area	Area %/%
1	23. 135	61617	2644692	53. 789
2	27. 649	41141	2272099	46. 211

<Chromatogram>

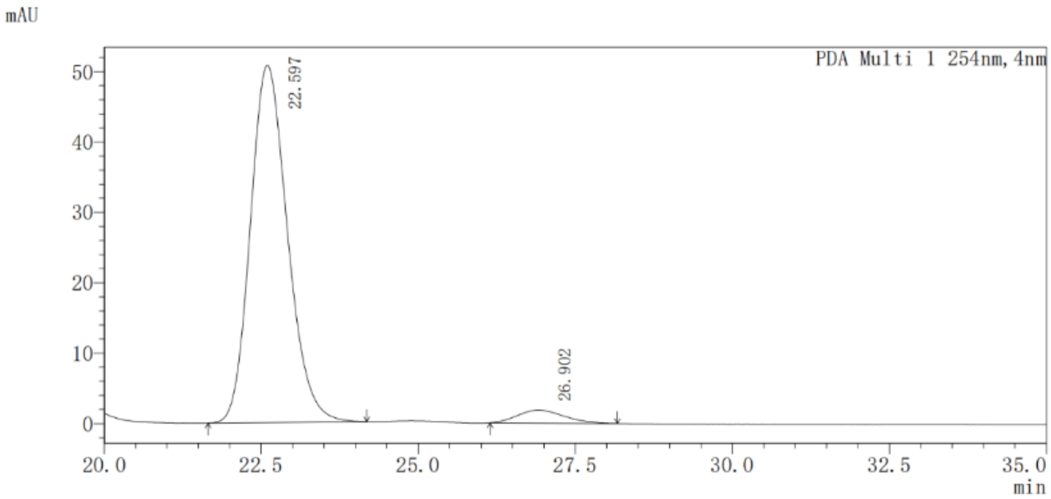

<Peak Results>

PDA Ch1 254nm

Index	Time/min	Height/mAU	Quantity/Area	Area %/%
1	22. 597	50755	2087450	95. 683
2	26. 902	1844	94173	4. 317

Supplementary Fig. 168. HPLC chromatograms of compound **3am.**

HPLC (ChiralPak IC, 2% *i*-PrOH in hexanes, 1.0 mL/min, 254 nm)

<Chromatogram>

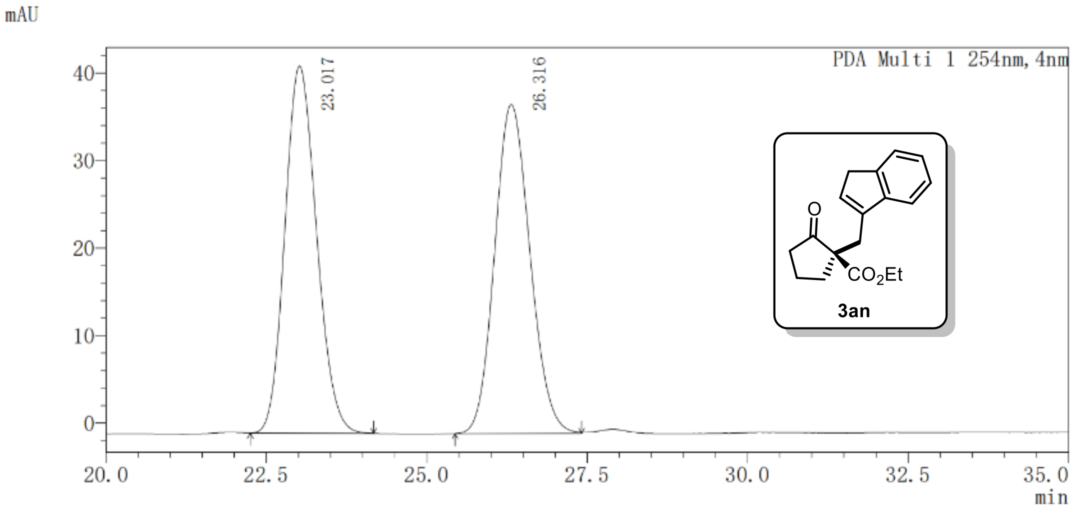

<Peak Results>

PDA Ch1 254nm

Index	Time/min	Height/mAU	Quantity/Area	Area %/%
1	23.017	41968	1443302	50.019
2	26.316	37636	1442232	49.981

<Chromatogram>

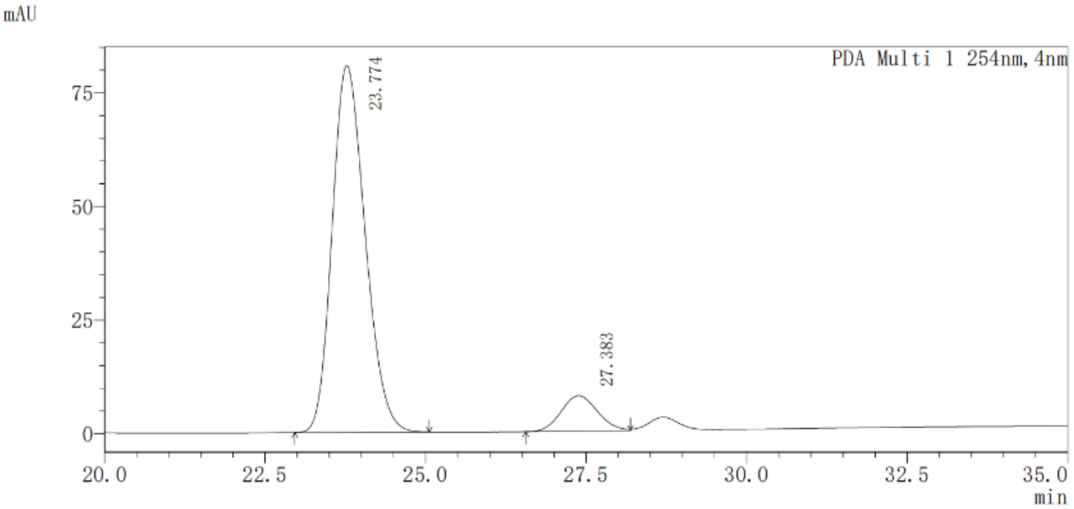

<Peak Results>

PDA Ch1 254nm

Index	Time/min	Height/mAU	Quantity/Area	Area %/%
1	23.774	80723	2880915	90.391
2	27.383	7802	306260	9.609

Supplementary Fig. 169. HPLC chromatograms of compound **3an.**

HPLC (ChiralPak OD-H, 2% *i*-PrOH in hexanes, 1.0 mL/min, 263 nm)

<Chromatogram>

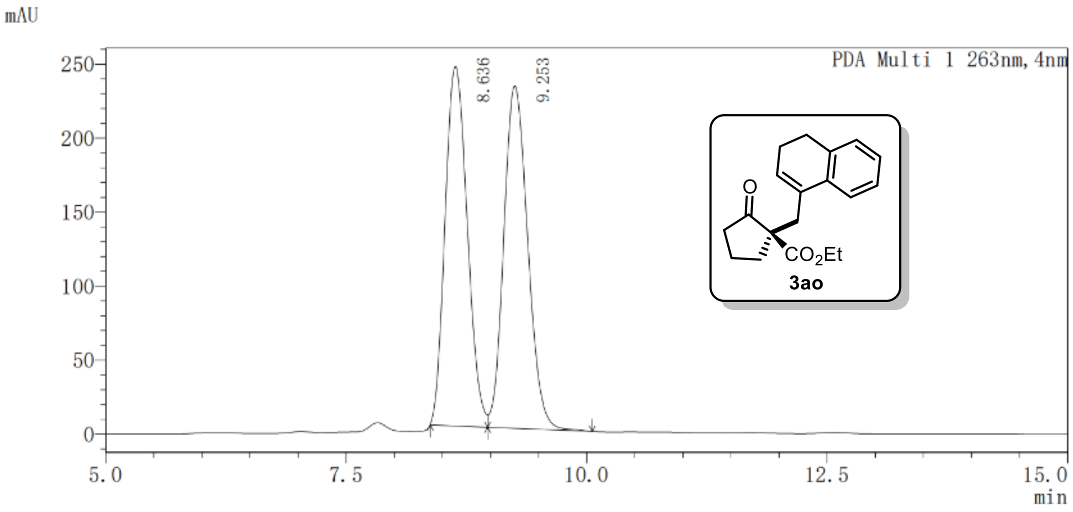

<Peak Results>

PDA Ch1 263nm

Index	Time/min	Height/mAU	Quantity/Area	Area %/%
1	8.636	242888	3898308	49.364
2	9.253	231258	3998813	50.636

<Chromatogram>

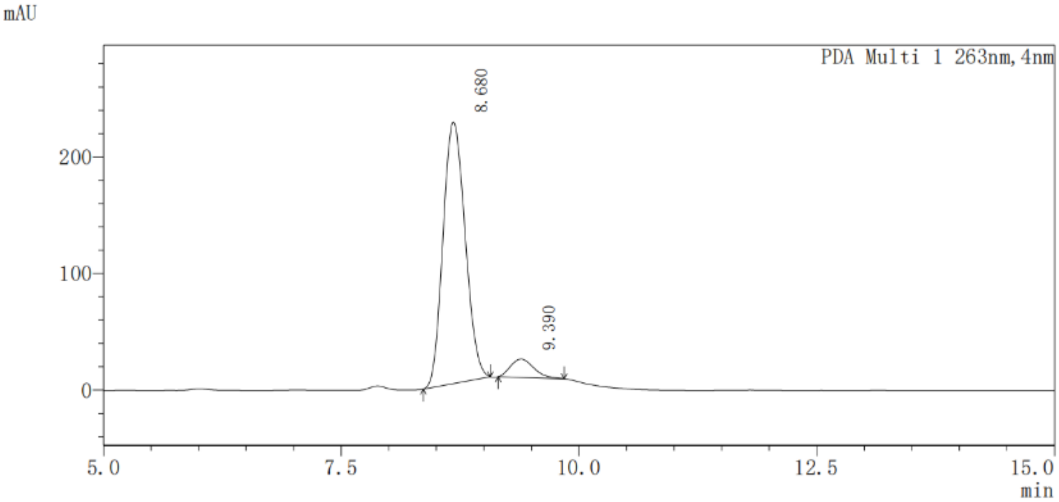

<Peak Results>

PDA Ch1 263nm

Index	Time/min	Height/mAU	Quantity/Area	Area %/%
1	8.680	224369	3632846	93.124
2	9.390	15711	268224	6.876

Supplementary Fig. 170. HPLC chromatograms of compound **3ao.**

HPLC (ChiralPak IC-H, 2% *i*-PrOH in hexanes, 1.0 mL/min, 254 nm)

<Chromatogram>

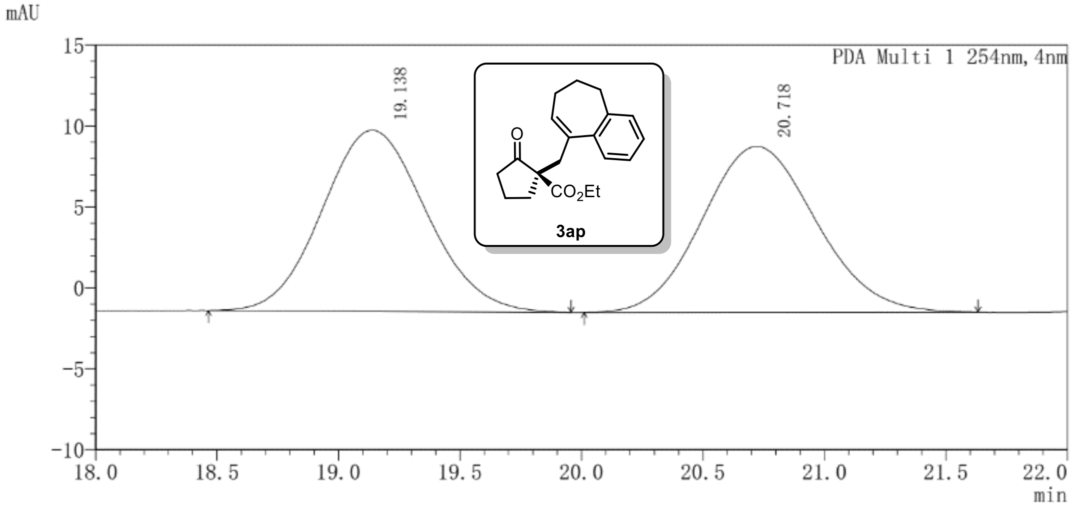

<Peak Results>

PDA Ch1 254nm

Index	Time/min	Height/mAU	Quantity/Area	Area %/%
1	19.138	11191	334255	49.980
2	20.718	10249	334526	50.020

<Chromatogram>

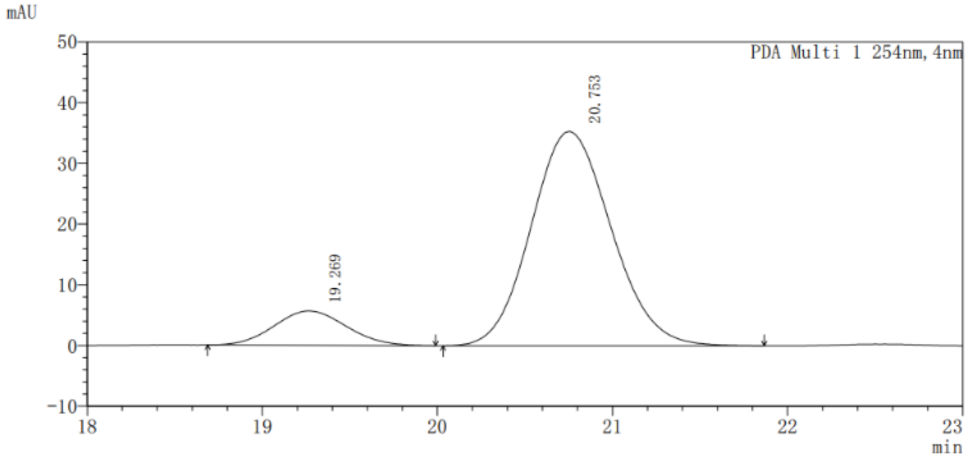

<Peak Results>

PDA Ch1 254nm

Index	Time/min	Height/mAU	Quantity/Area	Area %/%
1	19.269	5654	162358	12.624
2	20.753	35283	1123796	87.376

Supplementary Fig. 171. HPLC chromatograms of compound **3ap.**

HPLC (ChiralPak OJ, 5% *i*-PrOH in hexanes, 1.0 mL/min, 254 nm)

<Chromatogram>

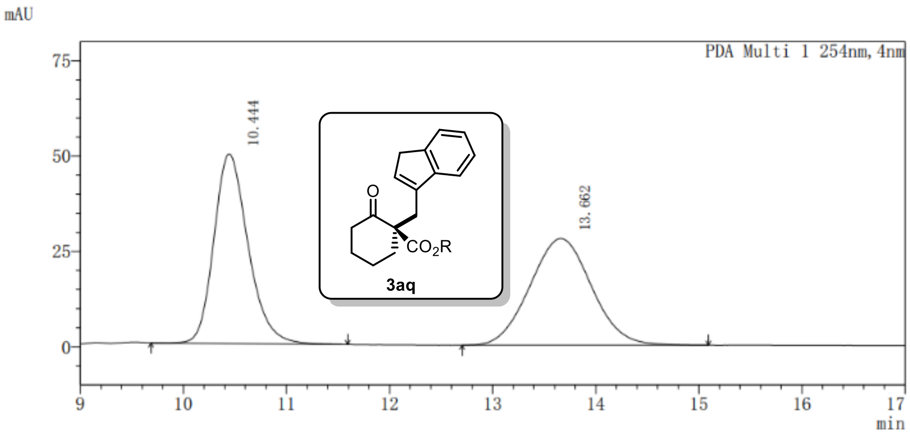

<Peak Results>

PDA Ch1 254nm

Index	Time/min	Height/mAU	Quantity/Area	Area %/%
1	10.444	49741	1148768	49.610
2	13.662	27968	1166827	50.390

<Chromatogram>

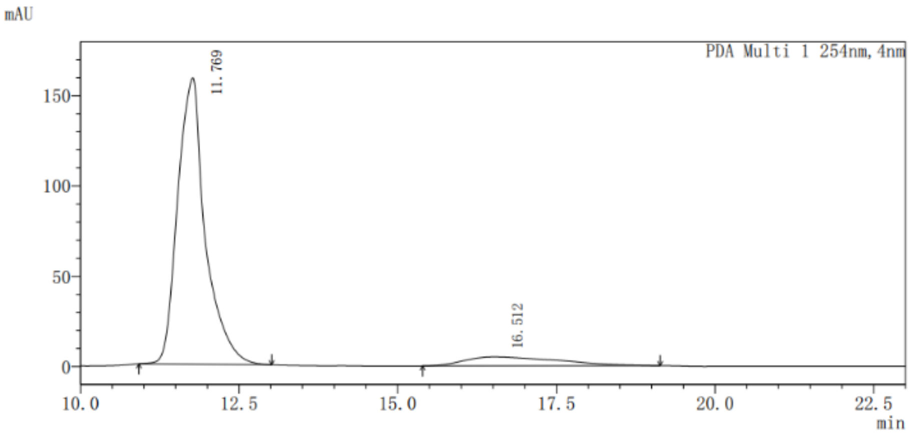

<Peak Results>

PDA Ch1 254nm

Index	Time/min	Height/mAU	Quantity/Area	Area %/%
1	11.769	158642	4796510	90.316
2	16.512	4990	514327	9.684

Supplementary Fig. 172. HPLC chromatograms of compound **3aq.**

HPLC (ChiralPak OJ, 1% *i*-PrOH in hexanes, 1.0 mL/min, 254 nm)

<Chromatogram>

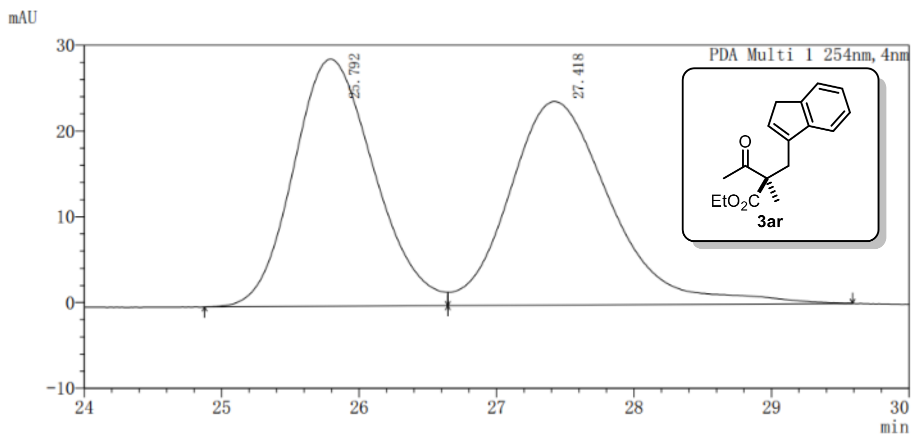

<Peak Results>

PDA Ch1 254nm

Index	Time/min	Height/mAU	Quantity/Area	Area %/%
1	25.792	28807	1183280	49.255
2	27.418	23734	1219097	50.745

<Chromatogram>

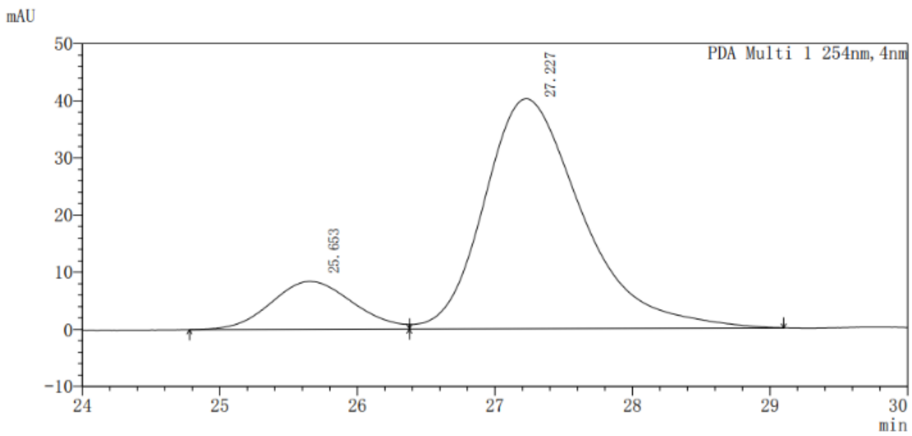

<Peak Results>

PDA Ch1 254nm

Index	Time/min	Height/mAU	Quantity/Area	Area %/%
1	25.653	8364	346414	14.793
2	27.227	40222	1995362	85.207

Supplementary Fig. 173. HPLC chromatograms of compound **3ar.**

HPLC (ChiralPak IC-H, 2% *i*-PrOH in hexanes, 1.0 mL/min, 254 nm)

<Chromatogram>

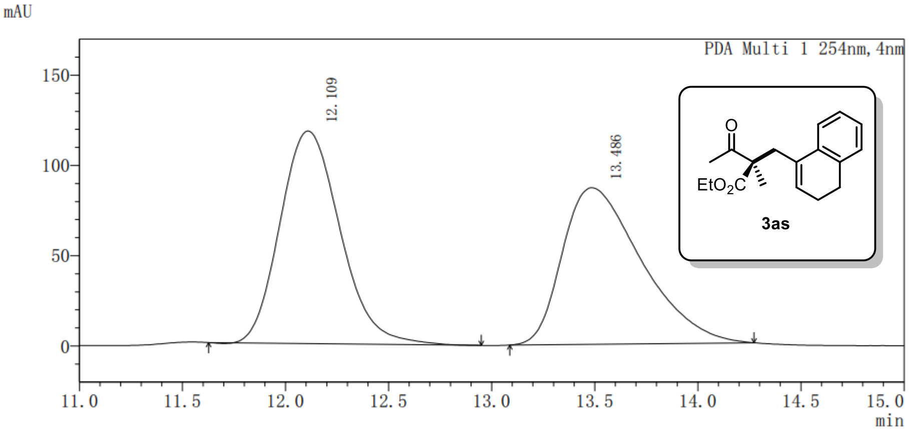

<Peak Results>

PDA Ch1 254nm

Index	Time/min	Height/mAU	Quantity/Area	Area %/%
1	12.109	117907	2391336	50.766
2	13.486	86762	2319216	49.234

<Chromatogram>

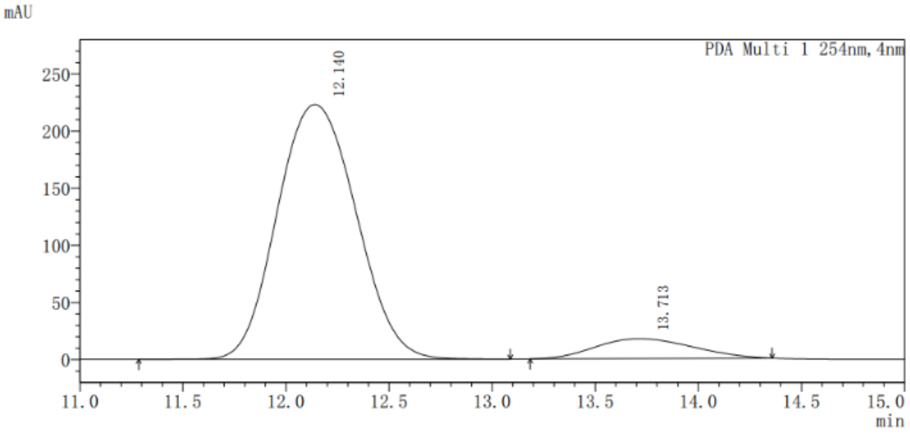

<Peak Results>

PDA Ch1 254nm

Index	Time/min	Height/mAU	Quantity/Area	Area %/%
1	12.140	223073	5949996	91.210
2	13.713	17418	573400	8.790

Supplementary Fig. 174. HPLC chromatograms of compound **3as.**

7. References:

1. Cai, M. et al. Chiral primary amine/ketone cooperative catalysis for asymmetric α-hydroxylation with hydrogen peroxide. *J. Am. Chem. Soc*. **143**, 1078-1087 (2021).
2. Cartwright, K. C., Joseph, E., Comadoll, C. G. & Tunge, J. A. Photoredox/cobalt dual-catalyzed decarboxylative elimination of carboxylic acids: development and mechanistic insight. *Chem. Eur. J*. **26**, 12454-12471 (2020).
3. Zhang, Q., Li, Y., Zhang, L. & Luo, S. Catalytic asymmetric disulfuration by a chiral bulky three component Lewis acid-base. *Angew. Chem. Int. Ed.* **60**, 10971-10976 (2021).
4. Yang, N.-Y., Li, Z.-L., Ye, L., Tan, B. & Liu, X.-Y. Organic base-catalysed solvent-tuned chemoselective carbotrifluoromethylation and oxytrifluoromethylation of unactivated alkenes. *Chem. Commun.* **52**. 9052-9055 (2016).
5. Zhang, L. et al. Chemoselective oxidative c(co)-c(methyl) bond cleavage of methyl ketones to aldehydes catalyzed by cui with molecular oxygen. *Angew. Chem. Int. Ed.* **52**, 11303-11307 (2013).
6. Jang, Y. J. et al. Green-light-driven Fe(III)(btz)$_3$ photocatalysis in the radical cationic [4+2] cycloaddition reaction. *Org. Lett.* **24**, 4479-4484 (2022).
7. Hong, Y., Dong, M.-Y., Li, D.-S. & Deng, H.-P. Photoinduced three-component carboarylation of unactivated alkenes with protic C(sp^3)–H feedstocks. *Org. Lett.* **24**, 7677-7684 (2022).
8. Sun, X., Chen, J. & Ritter, T. Catalytic dehydrogenative decarboxyolefination of carboxylic acids. *Nat. Chem*. *10*, 1229-1233 (**2018)**.
9. Morandi, B., Wickens, Z. K. & Grubbs, R. H. Practical and general palladium-catalyzed synthesis of ketones from internal olefins. *Angew. Chem. Int. Ed*. **52**, 2944-2948 (2013).
10. Zhu, Y. Zhang, L. & Luo, S. Asymmetric *α*-photoalkylation of *β*-ketocarbonyls by primary amine catalysis: facile access to acyclic all-carbon quaternary stereocenters. *J. Am. Chem. Soc.* **136**, 14642-14645 (2014).
11. Jia, Z. & Luo, S. Visible light promoted direct deuteration of alkenes via Co(III)–H mediated H/D exchange *CCS Chem.* **5**, 1069-1076 (2023).
12. Li, Y., Zhang, L. & Luo, S. Bond energies of enamines. *ACS Omega. 7*, 6354-8374 (2022).
13. Gaussian 09, Revision D.01, Frisch, M. J.; Trucks, G. W.; Schlegel, H. B.; Scuseria, G. E.; Robb, M. A.; Cheeseman, J. R.; Scalmani, G.; Barone, V.; Mennucci, B.; Petersson, G. A.; Nakatsuji, H.; Caricato, M.; Li, X.; Hratchian, H. P.; Izmaylov, A. F.; Bloino, J.; Zheng, G.; Sonnenberg, J. L.;

Hada, M.; Ehara, M.; Toyota, K.; Fukuda, R.; Hasegawa, J.; Ishida, M.; Nakajima, T.; Honda, Y.; Kitao, O.; Nakai, H.; Vreven, T.; Montgomery, Jr., J. A.; Peralta, J. E.; Ogliaro, F.; Bearpark, M.; Heyd, J. J.; Brothers, E.; Kudin, K. N.; Staroverov, V. N.; Keith, T.; Kobayashi, R.; Normand, J.; Raghavachari, K.; Rendell, A.; Burant, J. C.; Iyengar, S. S.; Tomasi, J.; Cossi, M.; Rega, N.; Millam, J. M.; Klene, M.; Knox, J. E.; Cross, J. B.; Bakken, V.; Adamo, C.; Jaramillo, J.; Gomperts, R.; Stratmann, R. E.; Yazyev, O.; Austin, A. J.; Cammi, R.; Pomelli, C.; Ochterski, J. W.; Martin, R. L.; Morokuma, K.; Zakrzewski, V. G.; Voth, G. A.; Salvador, P.; Dannenberg, J. J.; Dapprich, S.; Daniels, A. D.; Farkas, O.; Foresman, J. B.; Ortiz, J. V.; Cioslowski, J.; Fox, D. J. Gaussian, Inc., Wallingford CT, 2009.

14. Zhao, Y. & Truhlar, D. G. Density functionals with broad applicability in chemistry. *Acc. Chem. Res.* **41**, 157-167 (2008).
15. Zhao, Y. & Truhlar, D. The M06 suite of density functionals for main group thermochemistry, thermochemical kinetics, noncovalent interactions, excited states, and transition elements: two new functionals and systematic testing of four M06-class functionals and 12 other functionals. *Theor. Chem. Acc.* **120**, 215-241 (2008).
16. Cancès, E., Mennucci, B. & Tomasi, J. A new integral equation formalism for the polarizable continuum model: theoretical background and applications to isotropic and anisotropic dielectrics. *J. Chem. Phys.* **107**, 3032-3041 (1997).
17. Tomasi, J., Mennucci, B. & Cancès, E. The IEF Version of the PCM Solvation Method: An overview of a new method addressed to study molecular solutes at the qm ab initio level. *J. Mol. Struct. (Theochem)*, **464**, 211-226 (1999).
18. Tomasi, J., Mennucci, B. & Cammi, R. Quantum Mechanical Continuum Solvation Models. *Chem. Rev.* **105**, 2999-3093 (2005).
19. Luchini, G., Alegre-Requena, J. V., Funes-Ardoiz, I. & Paton, R. S. GoodVibes: Automated thermochemistry for heterogeneous computational chemistry data. *F1000Research*, *9*, 291 (2020).
20. Ho, J. & Coote, M. L. A universal approach for continuum solvent pK_a calculations: Are we there yet? *Theor. Chem. Acc.* **125**, 3-21 (2010).
